# Southern Tibetan rifting since late Miocene enabled by basal shear of the underthrusting Indian lithosphere

**DOI:** 10.1038/s41467-023-38296-w

**Published:** 2023-05-04

**Authors:** Bingfeng Zhang, Xuewei Bao, Yingkai Wu, Yixian Xu, Wencai Yang

**Affiliations:** Key Laboratory of Geoscience Big Data and Deep Resource of Zhejiang Province, School of Earth Sciences, Zhejiang University, Hangzhou, China

**Keywords:** Seismology, Geophysics

## Abstract

Syncontractional extension is prominent in present-day Tibet, but its origin remains vigorously debated. Several deep-seated geodynamic processes (e.g., Indian underthrusting, horizontal flow, and mantle upwelling) have been linked to Tibetan rifting. Indian underthrusting is a good candidate because it can well explain why surface rifts are more prominent south of the Bangong–Nujiang suture; however, how Indian underthrusting causes extension is not well understood and lacks observational constraints. Seismic anisotropy, measured by exploiting the birefringence effect of shear waves, can be indicative of the deformation styles within the crust. Here, we unveil the dominant convergence-parallel alignment of anisotropic fabrics in the deep crust of the southern Tibetan rifts using seismic recordings collected from our recently deployed and existing seismic stations. This finding suggests that the strong north-directed shearing exerted by the underthrusting Indian plate is key to enabling present-day extension in southern Tibet.

## Abstract (web summary)

This study presents constraints on seismic anisotropy properties in western-central Tibet, revealing pronounced north-directed basal shearing beneath the rifts in the southern plateau that sheds light on the cause of syncontractional extension there.

## Introduction

**Fig. 1:**
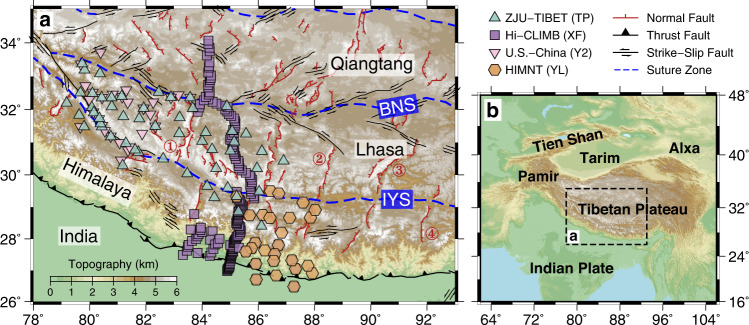
Shaded relief map of the study region. **a** Topographic map of the Tibetan Plateau highlighting the faults and suture zones from Taylor and Yin^3^. The rifts mentioned in the main text are labeled (1: Lunggar; 2: Dinggye–Xainza; 3: Yadong–Gulu; 4: Cona–Woka). See Supplementary Data 1 and references therein for the estimates on rift initiation timing and later stage of rift acceleration timing established by previous geological investigations. The locations of broadband seismic stations used in this study, designated for revealing seismic azimuthal anisotropy in west-central Tibet, are shown by color-filled symbols. **b** Sketch tectonic setting of the Tibetan Plateau and surrounding areas. BNS Bangong–Nujiang suture, IYS Indus–Yarlung suture.

The Tibetan Plateau, the largest compressional orogenic system on Earth, is currently undergoing orogen-wide crustal extension accommodated by a series of north‒south-trending rifts^1–3^ (Fig. 1). However, to date, whether and how these large-scale extensional structures manifested on the surface relate to tectonic processes at depth are still unclear and remain intensely debated.

Geological data indicate that rift extension is a widespread feature within the high plateau and that the majority of rifts initiated in the Miocene (Fig. 1; Supplementary Data 1 and references therein). Despite these general behaviors, rifting in southern Tibet (south of the Karakorum–Jiali fault zone, KJFZ) is much more developed than farther north, as suggested by the regularly spaced, >100-km-long morphology of the southern Tibetan rifts and the smaller, more scattered rifts in the north. The cause of such a difference may be attributed to the addition of north-directed basal shear forces beneath the southern plateau (imposed by the northwards-indenting Indian plate), which is a notion that is based on numerical and analog modeling of surface tectonics^4–6^.

**Fig. 2:**
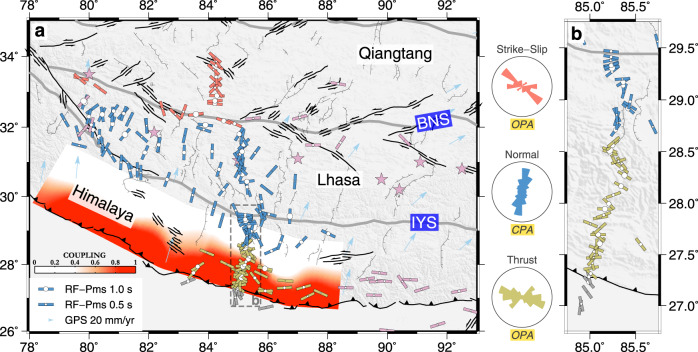
Crustal anisotropy parameters in Tibet. **a** All measurements. **b** A zoomed view of the measurements in the Himalayan terrane along the Hi-CLIMB seismic line. Fast axis and delay time are indicated by orientation of the bar line and size of the circle, respectively. Rose diagrams show distribution of the fast orientations for different tectonic regimes. The most frequent direction of anisotropy is represented by the longest pie slice. The bin size is 20 degrees for each of the plots with the first bin centered at N10°E. See Supplementary Fig. 2 for the number of measurements in different azimuthal bins. Strong CPA characterizes the crust of the southern Tibetan rifts. Measurements inside the Indian plate are marked in gray color. Previous published measurements^28,30, 31^ in Tibet using a similar analysis technique are shown in purple for comparison. We also present in this figure the previously inferred Indian crustal front from receiver-function imaging (purple stars)^7–11^ and the degree of interseismic coupling on the Main Himalayan thrust as indicated by shades of red^51^, which allow for comparison with the northern and southern limits of the southern Tibetan rifts, respectively. Light blue arrows denote GPS velocities relative to the stable Eurasia^70^. BNS Bangong–Nujiang suture, IYS Indus–Yarlung suture, CPA Convergence-Parallel Anisotropy, GPS Global positioning system, OPA Orogen-Parallel Anisotropy.

The basal-shear model requires that the Indian plate underthrusts Tibet rather than subducts steeply into the mantle (an essential requirement for subhorizontal shearing) and that the extent of the underthrusting Indian plate coincides with that of the southern Tibetan rifts. Mechanical coupling between the Indian and Tibetan crust is also a basic prerequisite for the upwards transport of shear stresses^4^. Geophysical imaging of subsurface structures provides the means to verify some of these predictions. Although numerous tomographic seismic wavespeed images disagree as to how far the high-velocity Indian lithosphere has underthrusted northwards beneath Tibet, receiver-function (RF) imaging along several north‒south-trending passive-seismic profiles^7–11^ has indicated a general consistency between the northern limits of the underplated Indian crust (purple stars in Fig. 2) and the southern Tibetan rifts. This is important observational evidence for establishing the relation between Indian underthrusting and Tibetan extension but nonetheless provides no information on how underthrusting causes extension. Some researchers have suggested that rifting is mainly facilitated by mass balance accompanying lower crustal thickening^12^ rather than basal shear induced by northwards underthrusting.

Furthermore, the Tibetan crust is hot^13^ and contains local or extensive weak zones characterized by slow seismic velocity^14,15^, high conductivity^16^, and strong attenuation^17^. These observations have led to the conjecture that ductile channel flow exists in the middle–lower crust of the plateau^18^, which would induce the viscous buckling of the brittle upper crust and consequently the east‒west extension at shallow depths^19^. However, because several important attributes of the hypothesized crustal flow remain controversial, such as the spatial extent of its occurrence^20^ and mode of flow (coherent eastwards flow^15^ or disordered flow^21^), the association between crustal flow and surface extension is still open for discussion. In addition, eastwards asthenospheric flow has been suggested below central Tibet due to the squeezing of the underthrusting Indian and Asian lithospheres, and this flow may be capable of creating paired general–shear deformation in the overlying upper crust and consequently the development of extensional structures, such as V-shaped conjugate strike-slip faults^22^.

In a third scenario, synorogenic extension across Tibet is attributed to the excess gravitational potential energy (GPE) arising from crustal overthickening and/or the convective instability of the lower mantle lithosphere and its replacement by the hot asthenosphere^1,23^. Slab tears in the underthrusting Indian lithosphere are also regarded as potential channels for localized asthenospheric upwelling, producing additional GPE that contribute to surface extension^24^. Such a claim is supported by the spatial distribution and isotopic signatures of the Gangdese volcano-magmatic rocks, implying diachronous subduction^25^ and various geophysical observations (e.g., LAB offsets^26^ and lithospheric low-velocity anomalies^14,24^), indicating slab gaps. Nevertheless, neither of these asthenospheric upwelling scenarios alone or together can explain the north‒south contrast in rifting patterns.

**Fig. 3:**
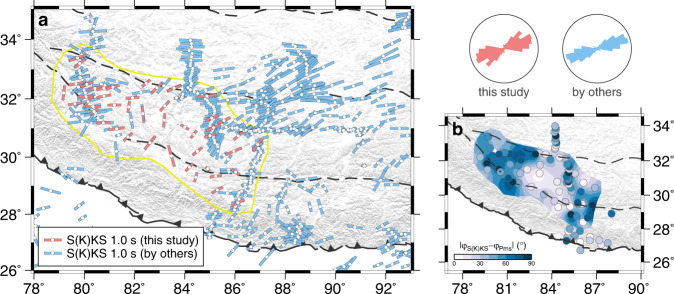
S(K)KS splitting measurements in Tibet. **a** Map view of the station-averaged S(K)KS measurements obtained in this study (red bars) and previous studies (blue bars)^28, 40^. Fast axis and delay time are shown by orientation and length of bar line, respectively. Rose diagram to the top right demonstrates the good correspondence between fast axes from this (red) and previous (blue) studies. Their construction is similar to the ones in Fig. 2, but with a bin size of 10 degrees and the first bin centered on 5 degrees (N5°E). Source data are individual S(K)KS measurements of the ZJU-Tibet array and station-averaged values of the previous ones. A cluster of anomalous fast axes is shown at (~83°E, ~31°N) and neighboring areas. **b** Map view of the differences between the Pms and S(K)KS fast axes. The background, with its extent outlined in Fig. 3a, is constructed using the Kriging gridding method. For stations in the central part of the ZJU-Tibet array, where anomalous S(K)KS fast axes dominate, the Pms and S(K)KS fast axes have similar orientations.

Information on the anisotropic properties of the solid Earth is essential for deciphering strain patterns at depth, which provides important constraints on the dominant tectonic processes that are prevalent in the region. Much progress has been made over the past several decades through the establishment of S(K)KS (i.e., core-refracted SKS and SKKS phases) splitting patterns across the Tibetan Plateau^27–30^ (Fig. 3). Since the S(K)KS data reflect accumulated anisotropy between the Earth’s core and the surface with poor depth resolution, tighter constraints on crustal seismic anisotropy are most often obtained by RF analysis. The crust in Tibet is considered highly anisotropic, as shown by earlier RF studies at some discrete locations exploiting the birefringence effect or azimuthal dependence of the Moho P-to-S converted phases^28,30,31^ (Pms). Anisotropy is not uniformly distributed but rather concentrated in several layers, such as above the Himalayan decollement^32^, near the surface and in the middle–lower crust^33,34^, which is a conjecture that is based on the waveform modeling of RFs.

In this work, to evaluate the abovementioned hypotheses and deepen our understanding of the mechanisms that drive active rifting in Tibet, we comprehensively characterize the nature of seismic azimuthal anisotropy within the crust of western–central Tibet using the Pms moveout fitting technique (see “Methods”). The depth of anisotropy is constrained based on the comparison with local S-wave splitting observations rather than the results of waveform modeling since the latter approach is likely subject to strong tradeoffs among model parameters and therefore may be highly nonunique^35^. Seismic data are collected from 53 newly deployed broadband seismographs operating from September 2018 to June 2021 (ZJU-Tibet) and previous seismic experiments, including Hi-CLIMB^8^, HIMNT^32^, and the U.S.‒China West Tibet array^36^ (Fig. 1). Compared to the dominant body- and surface-wave tomographic images in the region that show an averaged crustal anisotropy structure over hundreds of kilometers^37,38^, the dense array data used herein and the steep incidence of the Pms ray paths enable a higher-resolution illumination of crustal anisotropy in western–central Tibet than previously available. Consequently, the association between surface geological features (e.g., rifts) and underlying tectonic processes can now be explored from the relevant deformation fabrics at depth. Our observations delineate a basal shear zone beneath the southern plateau that closely correlates with the distribution of the rifts there, highlighting the key role of Indian underthrusting in the development of southern Tibetan rifts.

## Results and discussion

### Seismic anisotropy observations in western–central Tibet

Well-constrained crustal azimuthal anisotropy measurements at 171 stations are given in the form of fast orientation (${\varphi }_{Pms}$) and delay time ($\delta {t}_{Pms}$) (Fig. 2; Supplementary Data 2), which is an order of magnitude greater than the published results in western–central Tibet^30^. Explicit polarity changes are observed on fast- and slow-component waveforms at azimuths that correspond to the estimated ${\varphi }_{Pms}$ and its perpendicular direction (Supplementary Data 3), as in the synthetic case of a one-layered anisotropic crust (Supplementary Fig. 1), indicating the reliability of the observations.

Statistically speaking, the ${\varphi }_{Pms}$ measurements show explicit north‒south variations that correlate with the changes in tectonic regime within Tibet (Fig. 2; Supplementary Fig. 2). Convergence-parallel anisotropy (CPA, NNE‒SSW) seems to characterize most of the Lhasa terrane and the Tethys Himalaya (i.e., southern Tibetan rift zone), whereas orogen-parallel anisotropy (OPA, ESE‒WNW) is dominant in areas to the north and south that feature conjugate strike-slip faulting (with the occurrence of some minor normal faulting) and thrust faulting, respectively. The outliers may be related to the localized heterogeneous structures that may generate irregular Pms moveout (e.g., lateral variabilities in crustal velocity and thickness), the Moho transition zone and crustal reverberations that may complicate the shape and identification of Pms phases, and the interference of noise plus the incomplete azimuthal coverage. The average $\delta {t}_{Pms}$ for all measurements is 0.65 s, implying the highly anisotropic nature of the western–central Tibetan crust. The measurements generally show a more scattered pattern rather than an abrupt change in the transition zone of different regimes (Supplementary Fig. 2), which is expected, as relatively high strain is required to fully obscure the preexisting anisotropic fabrics^39^.

In Fig. 3, we compile our and previous S(K)KS splitting measurements in Tibet^27–30,40^, which are generally consistent and highlight a cluster of anomalous ${\varphi }_{S(K)KS}$ values in the center of the ZJU-Tibet array (centered at ~83°E, ~31°N; Supplementary Fig. 3). Interestingly, this cluster roughly corresponds to low shear-wave velocity zones in the uppermost mantle^14,41^ (Supplementary Fig. 4) and the parallelism between ${\varphi }_{S(K)KS}$ and ${\varphi }_{Pms}$ (Fig. 3b), suggesting that crustal anisotropy is the major source of S(K)KS splitting in this area. The contribution of the upper mantle is minor, likely because the ascending low-velocity asthenospheric flow may have resulted in weak mantle azimuthal anisotropy. This is in contrast to other parts of the western–central Lhasa terrane with thick mantle lithosphere, where both the crust and the mantle play important roles in forming the S(K)KS anisotropy.

### Formation mechanisms of the observed crustal anisotropy

The shape-preferred orientation of fluid-saturated cracks^42^ and the crystallographic preferred orientation (CPO) of anisotropic minerals (e.g., mica^43^ and amphibole^44^) are the two fundamental causes of crustal seismic anisotropy. The amounts of upper-crustal anisotropy in the study area (~0.12 s) are constrained by local S-wave splitting (Supplementary Fig. 5). This value, when compared to the Pms delay times, demonstrates that the main source of the observed Pms anisotropy should reside in the middle–lower crustal depths.

As cracks are mostly closed due to the higher pressure at such depths, the aforementioned large-scale north‒south variations in fast orientations should reflect the contrasting deep crustal processes producing the distinct CPO patterns. Taking amphibole as an example, which is an abundant constituent mineral in deep-crust xenoliths from southern Tibet and displays strong CPOs^45^, experimental studies^46^ have established that the alignment of its crystals caused by simple shear and thereby the orientation of high anisotropy are subparallel to the shear direction under high-temperature conditions, which is applicable to the Tibetan crust. Thus, the predominant CPA beneath the southern plateau provides direct observational evidence for the strong subhorizontal shearing between the northwards-underthrusting Indian crust and the overlying Tibetan crust. Such regional-scale resistive shearing caused by the indentation of the continental lithosphere is conceptually analogous to that found in compressional mountain belts worldwide (e.g., Taiwan^47^ and Tien Shan^48^) but affects a significantly larger area in comparison (~300 km wide).

Notably, the available Pms observations in the eastern KJFZ and south of the Yadong rift^28,31^ show predominantly E‒W orientations (i.e., OPA) (Fig. 2), similar to their counterparts in western–central Tibet. The Moho doublet signatures are found as far north as ~30.5°N at 90°E and ~31°N at 92°E, covering most or the entire surface trace of the Dinggye–Xainza, Yadong–Gulu, and Cona–Woka rifts^7,9^ (Fig. 2), implying that the northwards basal shear also takes effect under the southern Tibetan rifts to the east of the study area. If this is the case, the resulting transition of crustal anisotropy from ${\varphi }_{S(K)KS}$-parallel to ${\varphi }_{S(K)KS}$-normal orientations may have a broad implication for the southwards reduction in S(K)KS splitting times south of the Bangong–Nujiang suture that was previously observed at ~90°E^28,29^.

Furthermore, the large Pms delay time that we observe confirms that a larger portion of the crust is involved in the shearing than previously thought^8^ (~32–40 km if we assume that an ~13%^44^ anisotropic layer with 40–50% amphibole by vol. is responsible). The shear zone involves a 15-km-thick underthrusting Indian lower crust, as shown by the active- and passive-source seismic data^8,49^ and some of the Tibetan middle–lower crust above. Because the underthrusting Indian crust has undergone a longer history of shearing in its northern part than in its southern part, a higher degree of shear deformation is achieved in the north; the opposite is true for the overlying Tibetan middle–lower crust, which may explain why there is no obvious northwards increase in the Pms delay time. Thus, the Tibetan crust is well coupled to the underlying Indian lithosphere, even with the presence of crustal low-velocity zones^14,15^, which allows basal shear stresses to be conveyed upwards. Such mechanical coupling at mid-crustal depths is also key to reproducing surface features, such as the limited distortion of southern Tibetan palaeolake shorelines^50^ and the north‒south contrast in the tectonic regime^4^. The hypothesized large-scale eastwards flow of the weak lower crust^19^, on the other hand, is unlikely to develop in the western–central Lhasa terrane since the dominant CPA there is practically orthogonal to flow-parallel fast orientations predicted by this scenario.

The CPA is not manifested further south, where the simple shear of the plate motions is taken up by the brittle part of the Main Himalayan Thrust (MHT). This segment of the MHT appears to be nearly fully locked (highly coupled), as shown by geodetic data^51^ (Fig. 2), and its lateral extent correlates with the background seismicity. This suggests that most shear stresses emplaced on this segment of the MHT decollement are elastically absorbed in interseismic periods and released during large Himalayan earthquakes (e.g., the 2015 Mw 7.8 Gorkha–Nepal earthquake), which leads to an insignificant amount of anelastic strain accumulation over geological time scales and consequently few minerals aligned in the north‒south direction. These crustal minerals are instead aligned parallel to the strike of the Himalaya arc, as evidenced by the dominant OPA revealed in this area, reflecting pure shear deformation within the Himalayan orogenic prism under north‒south compression^52^. Fossil anisotropy retained in the underthrusting Indian crust also plays an important role, as the part of northern India that is away from the deformational front of the India–Eurasia collision^53^ shows predominantly ESE‒WNW-oriented crustal anisotropy with an ~0.4 s average delay time^31^. The pure-shear shortening scenario applies to northern Tibet as well, and when combined with the block motion along strike‐slip faults, causes OPA similar to that at the Himalayan collisional front.

### A conceptual model for southern Tibetan rifting

**Fig. 4:**
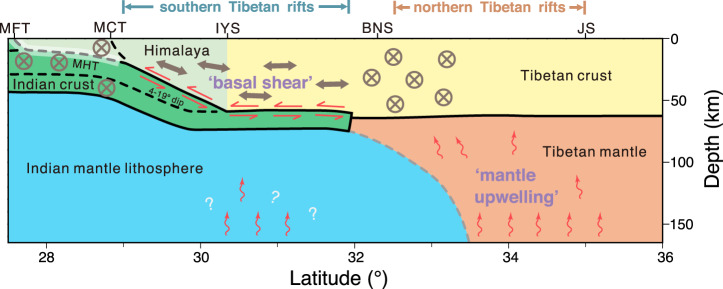
Cartoon illustrating how deep-earth dynamics relate to Tibetan rifting. The north‒south variations of fast orientations unveiled in this study are indicated by double-arrow (convergence-parallel anisotropy) and circled-X (orogen-parallel anisotropy) symbols. All other crustal architectures shown have been geophysically imaged^8, 49^. Basal shear (double-harpoon symbols) imposed by northwards-advancing Indian crust along the MHT (white-shaded, locked portion of the MHT not included) has dramatically modified the state of stress within the southern Tibetan crust, leading to both convergence-parallel anisotropy and active rifting observed there. Rift development in the northern Tibet, on the other hand, is linked to mantle upwelling processes beneath (wavy-arrow symbols), through increases in local gravitational potential energy and thermal weakening of remaining Tibetan lithosphere. BNS Bangong–Nujiang suture, IYS Indus–Yarlung suture, JS Jinsha suture, MFT Main Frontal Thrust, MCT Main Central Thrust, MHT Main Himalayan Thrust.

Our observations demonstrate that basal shear imposed by the underthrusting Indian plate is a prevalent tectonic forcing currently acting in southern Tibet beneath the north‒south-trending rifts (Fig. 4). The strong north-directed shearing in combination with topographically induced stresses is capable of balancing the north‒south compression from plate convergence and thereby rotates the maximum and minimum principal stress axes to the vertical and east‒west directions, respectively^4^. This stress state configuration is fundamental to initiating normal faulting, according to Anderson’s fault theory; without substantial north‒south extensional stresses caused by basal shear, strike-slip deformation dominates the southern plateau.

The underthrusting of the Indian slab affects the modification of the regional stress state alongside the tip of the Indian lower crust, which is an inference that coincides with the northwards propagation of accelerated extension, deduced to be at the Lunggar rift by thermochronological data^12^. We note that the outcome of the exerted basal shear is rift acceleration, not rift initiation, as the Indian plate underlying present-day southern Tibet started to underthrust at/before the time of rift initiation. The Indian slab would lie ~180–360 km south of its current location at 17–12 Ma given a geodetically determined convergence rate of 15–21 mm/yr^51,54^. In a popular view, the onset of north-trending rifts is thought to be initiated by the GPE increase accompanying the Indian slab breakoff ^55^ or the convective removal of the Lhasa lithosphere^23,56^. If that is the case, because the subsequent Indian underthrusting would fill the gaps left by the descending lithosphere and restore the GPE condition to that previously, the pronounced basal shearing is key to sustaining and strengthening the southern Tibetan rifting.

Present-day northern Tibet features both low upper mantle P and S velocities^14,24,41^ and inefficient Sn propagation^57^ (Supplementary Fig. 4), implying that much of the lithospheric mantle has been removed and asthenospheric upwelling has subsequently occurred. However, the rifts there are more scattered and less prominent than in the south; such a contrast indicates that basal shear imposed by the underthrusting Indian lithosphere has a better promotive effect on rift development than the convective removal of mantle lithosphere.

Furthermore, the underthrusting Indian slab is far from homogeneous. The local-scale low-velocity anomalies^14,24,41^ (Supplementary Fig. 6), the offset of the lithosphere–asthenosphere boundary^26^, and the presence of Moho disruption^58^ all favor the existence of multiple asthenospheric upwelling channels through the Indian slab, which is facilitated by slab tearing^25^ or convective removal^23^. This would in principle further enhance the southern Tibetan rifting locally. To verify this speculation, further studies are needed to assert the still-ambiguous upwelling locales and to quantify the extent to which they helped to promote modern extension. However, noting that mantle upwelling in southern Tibet is only a locally active process, we reinforce the idea that basal shear is the principal contributor to southern Tibetan rifting.

## Methods

### Crustal anisotropy from Pms moveout fitting

To gain insights into the poorly-understood geodynamic processes underlying western–central Tibet, we operated a two-phase portable seismic array (ZJU-Tibet) in the Lhasa and Tethyan Himalaya terranes from September 2018 to June 2021 with an average interstation spacing of ~50 km (Fig. 1). Specifically, 28 stations were installed west of 83°E during the first year (Phase I), 25 of which were then shifted eastwards during the following two years (Phase II). Each site was equipped with a Nanometrics Trillium Horizon broadband seismometer and a Centaur-3 digitizer with a sampling rate of 50 Hz^56^. To improve the horizontal resolution in the research region, we also add data recorded by previous seismological campaigns, including Hi-CLIMB^8^ (177 stations), HIMNT^32^ (26 stations), and the U.S.‒China West Tibet array^36^ (29 stations).

Well-recorded P-wave seismograms (defined by signal-to-noise ratio, SNR > 15) from large earthquakes with epicentral distances of 30–90° are utilized for the calculation of RFs (Supplementary Fig. 7). We adopt a lower cut-off body-wave magnitude of 5.5 to select events, which is reduced to 5.0 if back azimuth is within the range of 0–30° or 150–360° to gain better azimuthal coverage of the analyzed records. SNR is defined as the ratio of the peak-to-peak amplitude of the signal envelope (−2 to 7 s relative to P arrival) to the root-mean-square (RMS) amplitude of the noise window (−10 to −2 s) on the vertical-component waveforms, which are filtered in the frequency range of 0.02–1 Hz. We further rotate the two horizontal components into radial (R) and transverse (T) coordinates and construct P-wave RFs in the frequency domain^59^ with a Gaussian coefficient of 1.5 and a water level value of 0.1. All RFs with clear Pms phases are selected and moveout-corrected^60^ to a reference ray parameter of 0.06 s/km, and then averaged in 10° azimuthal bins to mitigate the effect of uneven earthquake distribution. It’s noteworthy that only radial RFs are used in the study. The transverse RFs, on the other hand, are susceptibly contaminated by unwanted seismic energy as is suggested by the unreasonably high peak amplitudes within the Pms windows (~65% that of the radial component), taking the value of ~24% (~44%) for a 50-km-thick anisotropic crust with 4% (8%) azimuthal anisotropy in synthetic cases as reference.

For shear waves passing through an anisotropic medium, the partitioning of energy to fast- and slow-propagating waves depends on the relation between initial polarization direction and symmetry axis of the medium. This phenomenon leads to the four-lobed ($\cos 2\theta$) azimuthal dependence of Pms signals on RFs if an anisotropic crust with a horizontal symmetry axis is considered^61,62^. On this basis, crustal azimuthal anisotropy beneath seismic stations, as indicated by ${\varphi }_{Pms}$ and $\delta {t}_{Pms}$, can be obtained through the least-squares harmonic fitting of Pms arrival times at different back azimuth^48,61,62^. Note that systematic azimuthal variations of Pms moveout can also result from a plunging symmetry axis of anisotropy or a dipping Moho geometry. Although such a pattern is two-lobed ($\cos \theta$), it may interfere with the search of $\cos 2\theta$ harmonics when dealing with real data. On this account, the harmonic fitting is conducted twice using (1) only the $\cos 2\theta$ function and (2) the combination of $\cos \theta$ and $\cos 2\theta$ functions, respectively:

1 $$t={t}_{0}-\frac{\delta {t}_{Pms}}{2}\,\cos [2(\theta -{\varphi }_{Pms})]$$

2 $$t={t}_{0}+\frac{{a}_{1}}{2}\,\cos (\theta -{\varphi }_{1})-\frac{\delta {t}_{Pms}}{2}\,\cos [2(\theta -{\varphi }_{Pms})]$$

where ${t}_{0}$ represents the Pms arrival time in isotropic case, $\delta {t}_{Pms}$ reflects the magnitude of crustal anisotropy and is equivalent to the maximum delay time between fast and slow shear waves, ${\varphi }_{Pms}$ is the fast orientation measured clockwise from the north, ${a}_{1}$ and ${\varphi }_{1}$ are amplitude and phase terms of the two-lobed variation, and $\theta$ is the back azimuth of the incoming ray. The parameters associated with crustal anisotropy are obtained through the grid search for a combination of ${t}_{0}$, ${\varphi }_{Pms}$, $\delta {t}_{Pms}$ (as well as two-lobed terms ${\varphi }_{1}$ and ${a}_{1}$ in the case of Eq. 2) that produces the smallest RMS residual between the observed and predicted Pms arrivals ($RM{S}_{fit}$). The search ranges are as follows: ${\varphi }_{Pms}$, 0–180° with a step of 2°; $\delta {t}_{Pms}$, 0–1.5 s with a step of 0.05 s; ${\varphi }_{1}$, 0–360° with a step of 2°; ${a}_{1}$, 0–1.5 s with a step of 0.05 s; ${t}_{0}$, a range limited by the minimum and maximum arrival times with a step of 0.1 s. Standard deviations (${\sigma }_{Pms}^{\varphi }$ and ${\sigma }_{Pms}^{\delta t}$) are estimated based on bootstrap resampling.

Since these two fitting schemes give similar results in most synthetic test cases and the latter performs better for the scenario that involves a weakly anisotropic crust^48^, the $\cos 2\theta$ estimates from the latter are presented as the final results for crustal anisotropy. For reliable results, several requirements also need to be satisfied: (1) the number of azimuthal bins ≥12 and the maximum azimuthal gap <180°; (2) $\delta {t}_{Pms} \, < \, 1.5$ s and ${a}_{1} \, < \, 1.5$ s (i.e., the maximum time shift); (3) the consistency between the results given by the two fitting schemes (${d}_{Pms}^{\varphi }\le 25$° and ${d}_{Pms}^{\delta t}\le 0.3$ s, respectively); (4) small uncertainties of the obtained crustal anisotropy (${\sigma }_{Pms}^{\varphi }/90+{\sigma }_{Pms}^{\delta t}\le 0.4$, as defined in Kong et al.^63^); (5) small moveout residual for the best fitting curve ($RM{{S}_{fit}}^{2}\le 0.3$ s^2^ for the former scheme and $RM{{S}_{fit}}^{2}\le 0.2$ s^2^ for the latter). All 171 Pms moveout fitting measurements are listed in Supplementary Data 2 and presented in Supplementary Data 4. See Supplementary Figs. 1 and 8 for a demonstration at station TP-XIZ.

### Upper-crustal anisotropy from local S-wave splitting

To estimate the contribution of upper-crustal deformation to the Pms observations, we also perform shear-wave splitting analyses for local earthquakes recorded by ZJU-Tibet stations using the MFAST package^64^. Events with local magnitudes greater than 1.0, focal depths within the range of 10–30 km, epicentral distances less than 100 km, and clear P and S arrivals on the horizontal components are selected and processed in MFAST as follows.

First, out of the 14 predefined bandpass filters tested, the one that gives the highest value of the product of SNR and filter bandwidth is chosen for each event independently. Noise and signal windows are taken as −3.05 to −0.05 s and 0.05 to 3.05 s relative to the manually picked S arrival, respectively; SNR is obtained by averaging the ratios of RMS (signal) and RMS (noise) for the north- and east-component waveforms. Then, a series of analysis windows are defined according to the dominant frequency of the S-wave signal, and for each window, the minimum eigenvalue method^65^ is used to calculate splitting parameters. The most stable solution is determined through cluster analysis^66^. That is, the cluster with the minimum total variance is chosen as the best cluster, and within that cluster, the measurement with the minimum variance is taken as the final measurement for the processed event.

Finally, we identify high-quality (A or B) splitting measurements according to a set of criteria that includes cluster grade (Acl for Quality A or Bcl for Quality B as defined in Savage, et al.^64^), SNR (>4 or >3), standard deviation of fast orientation (<10° or <25°), maximum of the eigenvalue of corrected covariance matrix (>5), delay time (<0.8 s), backazimuthal separation from the fast orientation and its perpendicular (>20°), and linearization of the initially elliptical particle motion. After quality assessment, we obtain a total of 35 pairs of Quality A or B measurements (Supplementary Data 5 and 6). See Supplementary Fig. 9 for an illustration of the procedure using a sample event, and Supplementary Fig. 5 for a general comparison with Pms moveout fitting measurements that target the anisotropy of the whole crust.

### Integrated anisotropy of crust and upper mantle from S(K)KS splitting

The core-refracted SKS and SKKS seismograms of the ZJU-Tibet data are processed for shear-wave splitting analyses. Earthquakes used have magnitudes greater than 5.6 (reduced to 5.5 for events deeper than 100 km) and belong to the epicentral distance range of 85–180° for SKS phases and 90–180° for SKKS. Clear S(K)KS phases with high SNR on the radial component (>4 as defined in Liu and Gao^67^) and meanwhile not interfered by other core-refracted/reflected phases (e.g., ScS, SKIKS) are identified and allowed for further analysis.

The selected waveforms are then bandpass-filtered (4–25 s) and processed using the SplitRacer software^68^, in which the transverse-component minimization technique^65^ is implemented to calculate splitting parameters (${\varphi }_{S(K)KS}$ and $\delta {t}_{S(K)KS}$). The start and end of the S(K)KS window are manually adjusted to focus on the S(K)KS energy and to reduce the standard deviation of the measurement. We repeat the processing for 50 slightly shifted S(K)KS windows. The 95% confidence level is estimated using the mean energy grid over all time windows and the modified inverse F-test of Walsh et al.^69^. The final pair of splitting parameters corresponds to the minimum value of the 95% confidence level.

The measurements are ranked into three categories (good, average, and poor). A good or average measurement should have (1) a clear phase onset, (2) good removal of energy (85% or 50%) on the transverse component after application of inverse splitting, which is also indicated by the linearization of initially elliptical particle motion, (3) a small 95% confidence level, (4) transverse component approximately proportional to the time derivative of the radial component, and (5) none or only minor scattering of splitting parameters for different time windows. The rest are designated to the group of poor and are not used. A total of 591 well-defined S(K)KS splitting parameters are finally obtained, including 139 good and 452 average ones (Supplementary Data 7 and 8). An example of good splitting observation is presented in Supplementary Fig. 10.

## Supplementary information

Supplementary Information

Description of Additional Supplementary Files

Supplementary Data 1

Supplementary Data 2

Supplementary Data 3

Supplementary Data 4

Supplementary Data 5

Supplementary Data 6

Supplementary Data 7

Supplementary Data 8

## Data Availability

Seismic data from Hi-CLIMB (10.7914/SN/XF_2002), HIMNT (10.7914/SN/YL_2001), and the U.S.‒China West Tibet (10.7914/SN/Y2_2007) experiments are archived and provided by the Incorporated Research Institutions for Seismology (IRIS) Data Management Center. The waveform data for P-wave receiver functions, local S, and S(K)KS waves obtained in this study can be downloaded from 10.5281/zenodo.7641796. All seismic anisotropy measurements are provided in the Supplementary Materials. Relief data shown in the figures are from https://topex.ucsd.edu/WWW_html/srtm30_plus.html.

## Code availability

The MFAST code is available at https://mfast-package.geo.vuw.ac.nz/. The SplitRacer package is freely accessible at https://www.geophysik.uni-frankfurt.de/64002762/Software. The MATLAB scripts used in the harmonic fitting of Pms moveout on receiver functions can be downloaded from 10.5281/zenodo.7641812. The GMT code used to create the figures is available at http://gmt.soest.hawaii.edu/.
