# Supplementary material for: Southern Tibetan rifting since late Miocene enabled by basal shear of the underthrusting Indian lithosphere
Source: Nat Commun. 2023 May 4;14:2565. doi: 10.1038/s41467-023-38296-w (PMC10160080; doi:10.1038/s41467-023-38296-w)
Supplement: Supplementary file 1 — Supplementary Information [file 41467_2023_38296_MOESM1_ESM.pdf]

Supplementary Materials

Southern Tibetan rifting since late Miocene enabled by basal shear of the underthrusting Indian lithosphere

Bingfeng Zhang, Xuewei Bao*, Yingkai Wu, Yixian Xu, Wencai Yang

This PDF file includes:

Supplementary Figures 1 to 10

Legends for Supplementary Data 1-8

Other Supplementary Materials for this manuscript include:

Supplementary Data 1-8

Inventory of Supplementary Materials:

- **Supplementary Figure 1.** Fast- and slow-component receiver functions after correction of crustal anisotropy.
- **Supplementary Figure 2.** Statistical graphs showing crustal azimuthal anisotropy parameters.
- **Supplementary Figure 3.** Map view of individual S(K)KS measurements obtained in this study.
- **Supplementary Figure 4.** Comparison between shear-velocity structures in Tibet constrained by adjoint tomography (EARA2014[1]) and surface-wave tomography (Bao2015[2]).
- **Supplementary Figure 5.** Statistical graphs showing local S-wave splitting parameters.
- **Supplementary Figure 6.** Shear-velocity structures in Tibet from EARA2014 model[1].

- **Supplementary Figure 7.** Azimuthal distribution of earthquakes and receiver functions used in the Pms moveout fitting.
- **Supplementary Figure 8.** An example of Pms moveout fitting analysis for station TP-XIZ.
- **Supplementary Figure 9.** Local S-wave splitting measurement for event 2021:113:13:40 recorded at station CUM categorized as Quality A.
- **Supplementary Figure 10.** An example of S(K)KS splitting analysis for event 2019-12-04-20:10:03 recorded at station WUM categorized as Quality good.
- **Supplementary Data 1.** Summary of initiation and acceleration timing of E-W extension across Tibet, modified from Bian, et al. [3]. **(separate file)**
- **Supplementary Data 2.** List of Pms moveout fitting measurements at individual stations. **(separate file)**
- **Supplementary Data 3.** Similar to Supplementary Fig. 1b but for 171 Pms moveout fitting measurements that pass all selection criteria. **(separate file)**
- **Supplementary Data 4.** Similar to Supplementary Fig. 8 but for 243 Pms moveout fitting measurements that have sufficient azimuthal coverage. **(separate file)**
- **Supplementary Data 5.** List of local S-wave splitting measurements at individual stations in the categories of A and B. **(separate file)**
- **Supplementary Data 6.** Similar to Supplementary Fig. 9 but for 35 local S-wave splitting measurements in the categories of A and B. **(separate file)**
- **Supplementary Data 7.** List of S(K)KS splitting measurements at individual stations in the categories of good and average. **(separate file)**
- **Supplementary Data 8.** Similar to Supplementary Fig. 10 but for 591 S(K)KS splitting measurements in the categories of good and average. **(separate file)**
- **Supplementary References**

Supplementary Figures

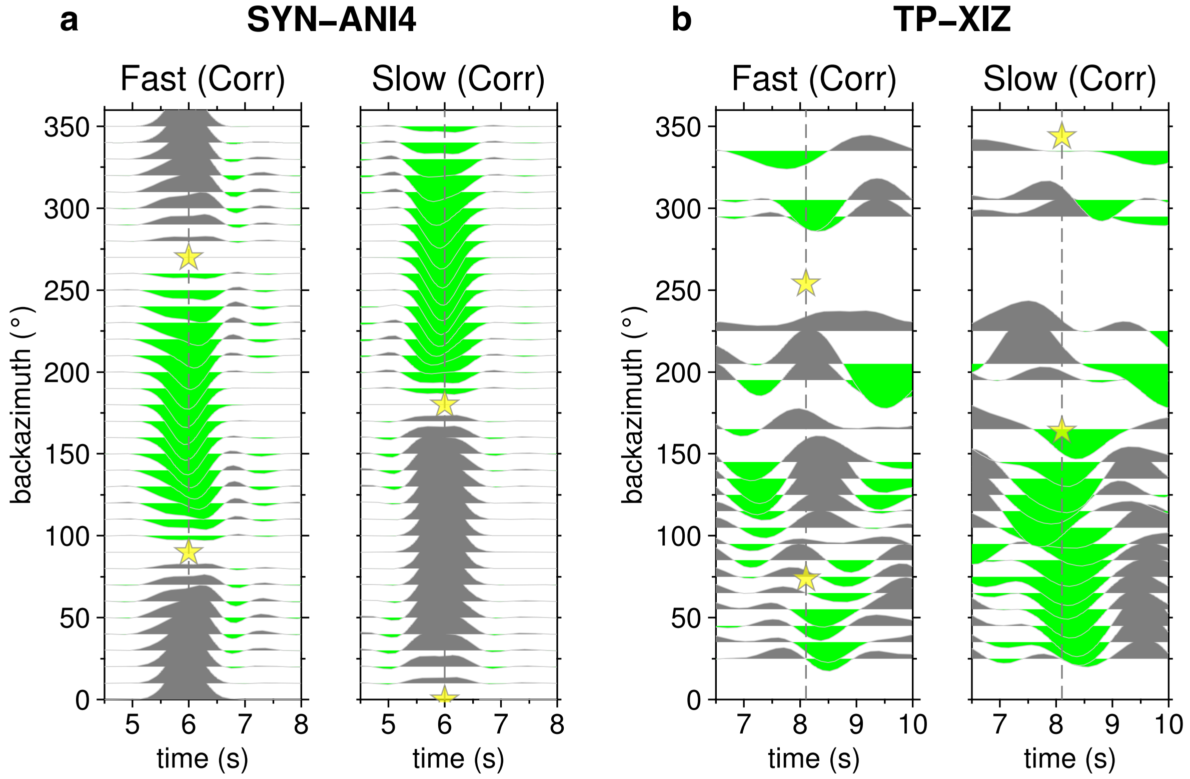

Supplementary Figure 1 | Fast- and slow-component receiver functions after correction of crustal anisotropy. (a) A synthetic case: one-layered anisotropic (4%) crust with 0° fast orientation. Receiver functions are plotted as a function of back azimuth with the positive amplitudes shown in gray and negative in green. Pms moveout fitting measurement is used in the correction of anisotropy. Gray dashed line shows the estimated Pms arrival time in the isotropic case. Yellow stars mark the estimated fast orientation and its perpendicular. Polarity changes are observed at these back azimuths. **(b)** A real data example for station TP-XIZ. Similar polarity change is found at azimuths that correspond to the fast orientation and its perpendicular, indicating the reliability of the observation. Corr = Correction.

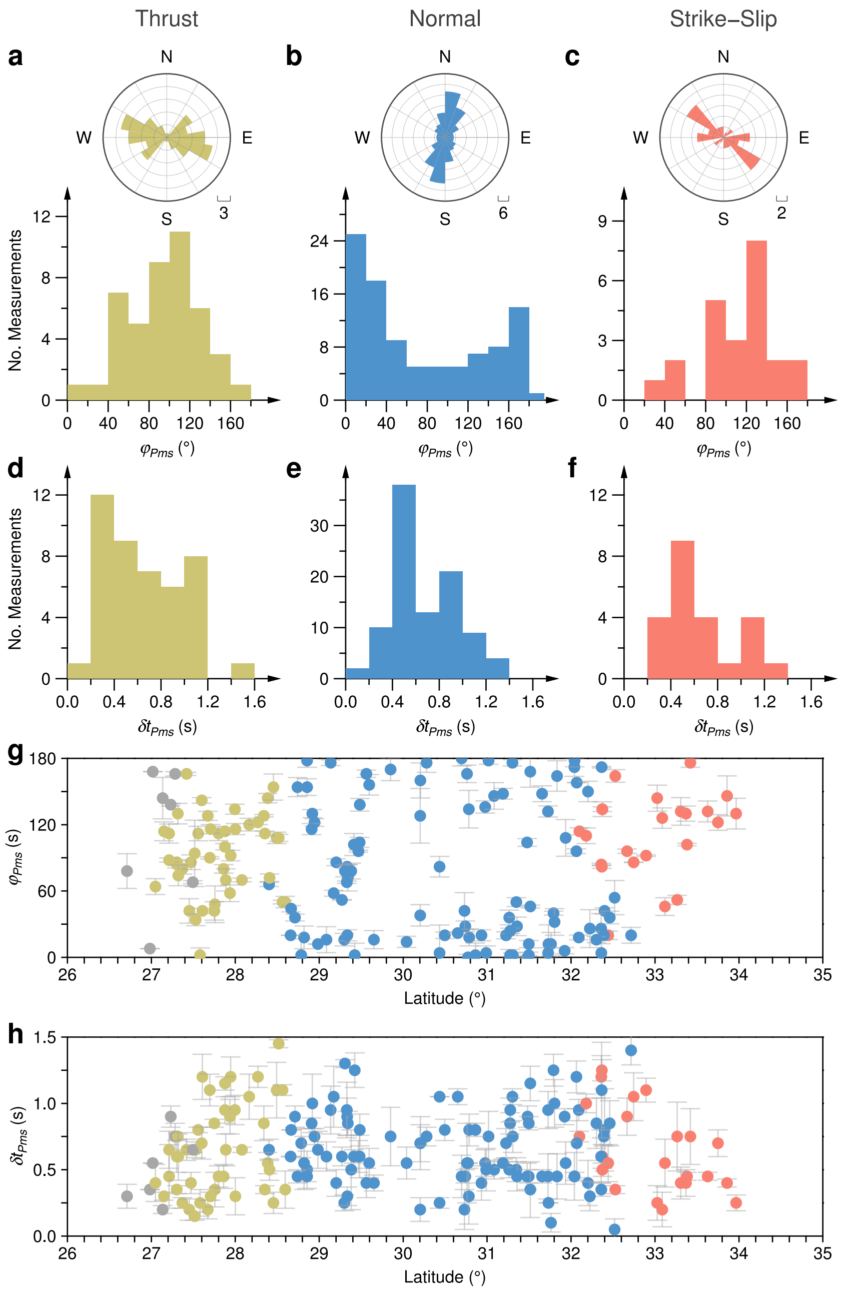

Supplementary Figure 2 | Statistical graphs showing crustal azimuthal anisotropy parameters. (a-c) Rose diagrams and histograms for estimated fast orientations in three tectonic regimes. The setup of azimuthal bins is as in Fig. 2. **(d-f)** Histograms for estimated delay times. **(g-h)** Fast orientations and delay times plotted along longitudinal profile. Measurement uncertainties are marked by gray bars.

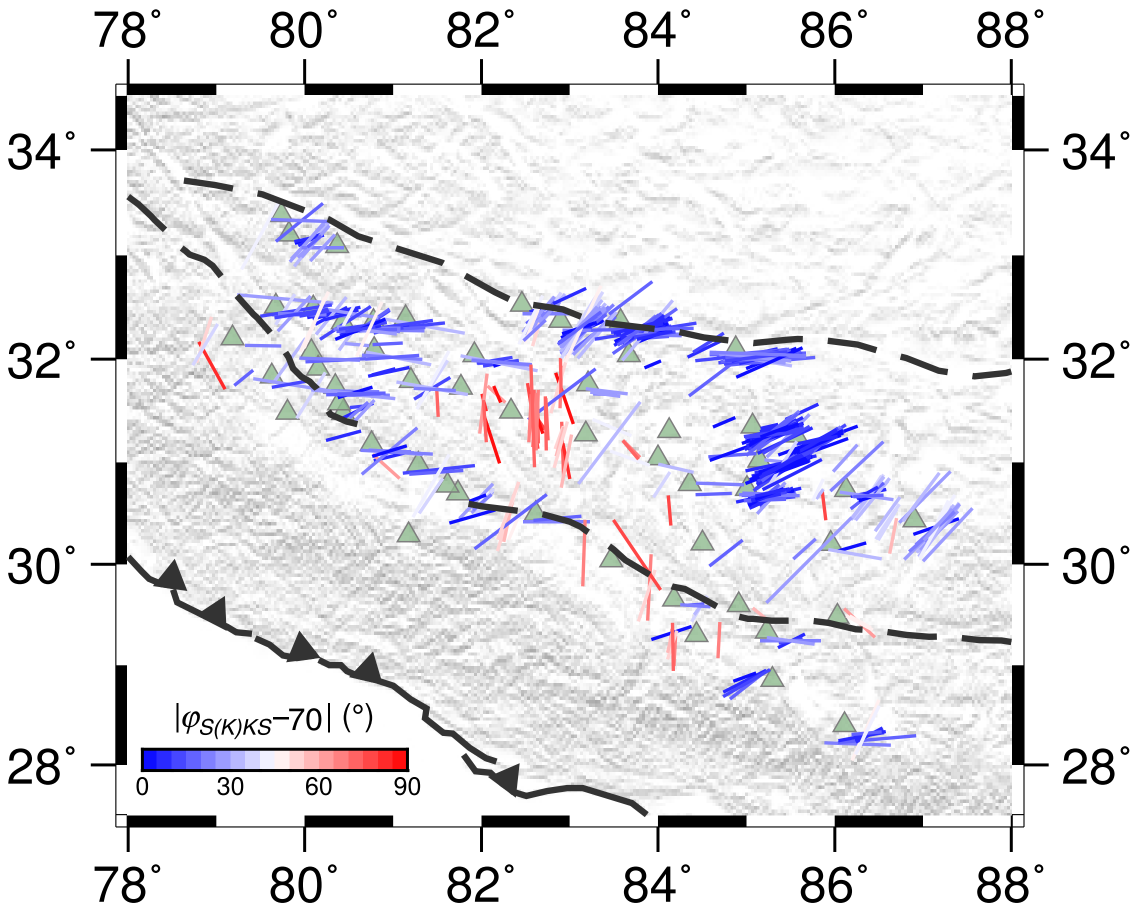

Supplementary Figure 3 | Map view of individual S(K)KS measurements obtained in this study. Measurements are projected to the piercing points at 140 km depth based on iasp91 Earth model. The fast axis and the amount of splitting are shown by the orientation and length of the bar line, respectively. The colors indicate the differences between the individual fast axis estimates and the general pattern in Tibet (N70°E). Green triangles show locations of ZJU-Tibet stations. A cluster of anomalous fast axes is observed in the central part of the ZJU-Tibet array.

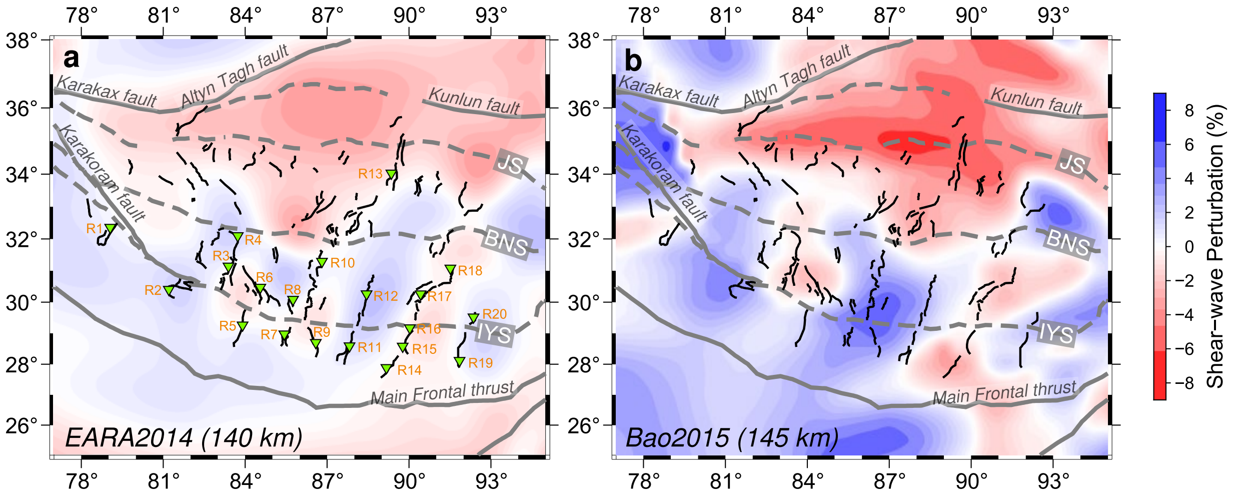

Supplementary Figure 4 | Comparison between shear-velocity structures in Tibet constrained by adjoint tomography (EARA2014[1]) and surface-wave tomography (Bao2015[2]). In both models, low-velocity anomalies feature a large area of northern Tibet indicating active mantle upwelling; high-velocity anomalies dominate the southern plateau representing the underthrusting Indian slab. The Indian slab is heterogenous as shown by the occurrences of several small-scale low-velocity anomalies therein; however, the locations of these anomalies haven't been agreed on. Black solid lines: all active rifts; gray dashed lines: major sutures; gray solid lines: major strike-slip/thrust faults. Tibetan rifts listed in Supplementary Data 1 are marked as triangles. R1: Leo Pargil; R2: Gurla Mandhata; R3: South Lunggar; R4: North Lunggar; R5: Thakkhola; R6: Lopukangri; R7: Gyirong; R8: Dajiamang Tso; R9: Kung Co; R10: Tangra Yum Co; R11: Dinggye; R12: Xainza; R13: Shuanghu; R14: Yadong; R15: Gyanze; R16: Ringbung; R17: Nyainqentanghla; R18: Gulu; R19: Cona; R20: Woka. BNS = Bangong–Nujiang suture, IYS = Indus–Yarlung suture, JS = Jinsha suture.

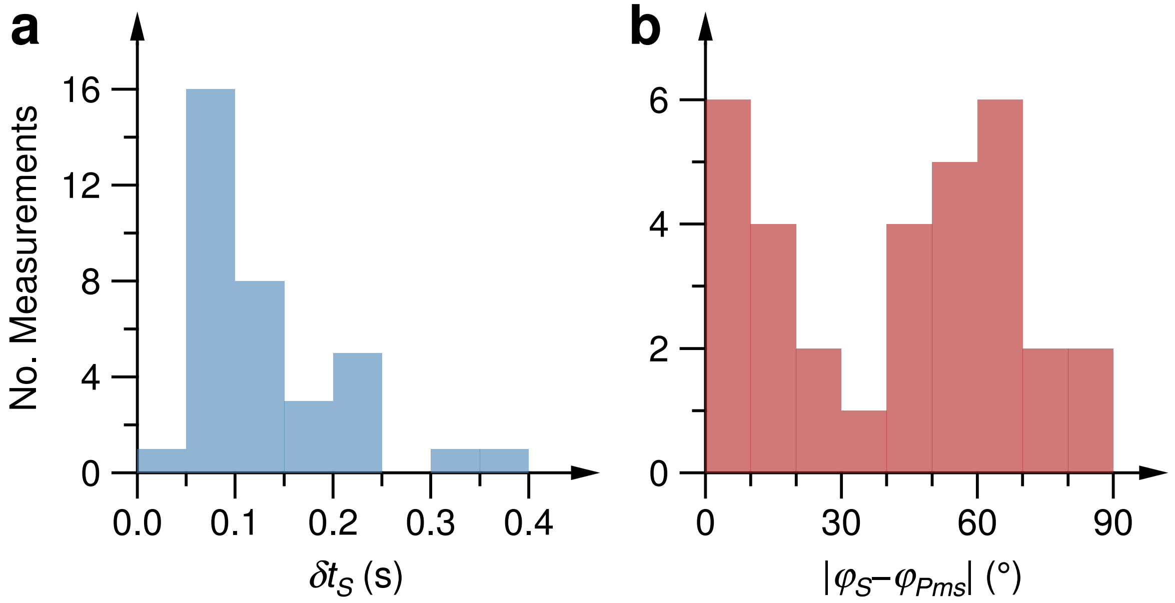

Supplementary Figure 5 | Statistical graphs showing local S-wave splitting parameters. (a) Histogram for estimated delay times. **(b)** Histogram for the differences between the individual fast orientation estimates and that of the Pms measurement at the same station.

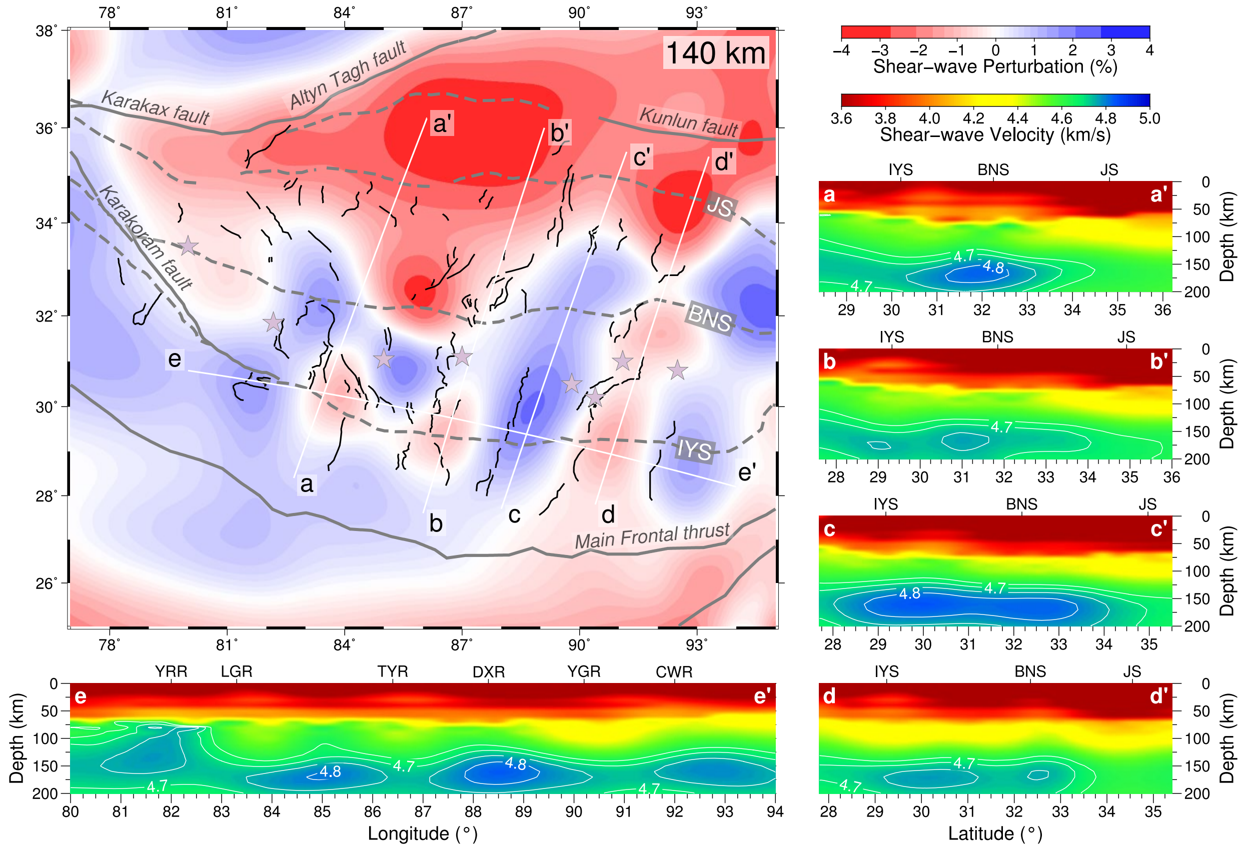

Supplementary Figure 6 | Shear-velocity structures in Tibet from EARA2014 model[1]. Left: Horizontal slice of shear-velocity perturbations at 140 km depth, similar to Supplementary Fig. 4a. As in Fig. 2, purple stars denote the previously inferred Indian crustal front. **Right and bottom:** Vertical slices of the shear-velocity model along four NNE-SSW and one ESE-WNW trending profiles shown in the left figure. Note the low-velocity vertical channels through the high-velocity Indian slab shown along profile ee'. BNS = Bangong–Nujiang suture, IYS = Indus–Yarlung suture, JS = Jinsha suture. YRR = Yari rift, LGR = Lunggar rift, TYR = Tangra Yum Co rift, DXR = Dinggye–Xainza rift, YGR = Yadong–Gulu rift, CWR = Cona–Woka rift.

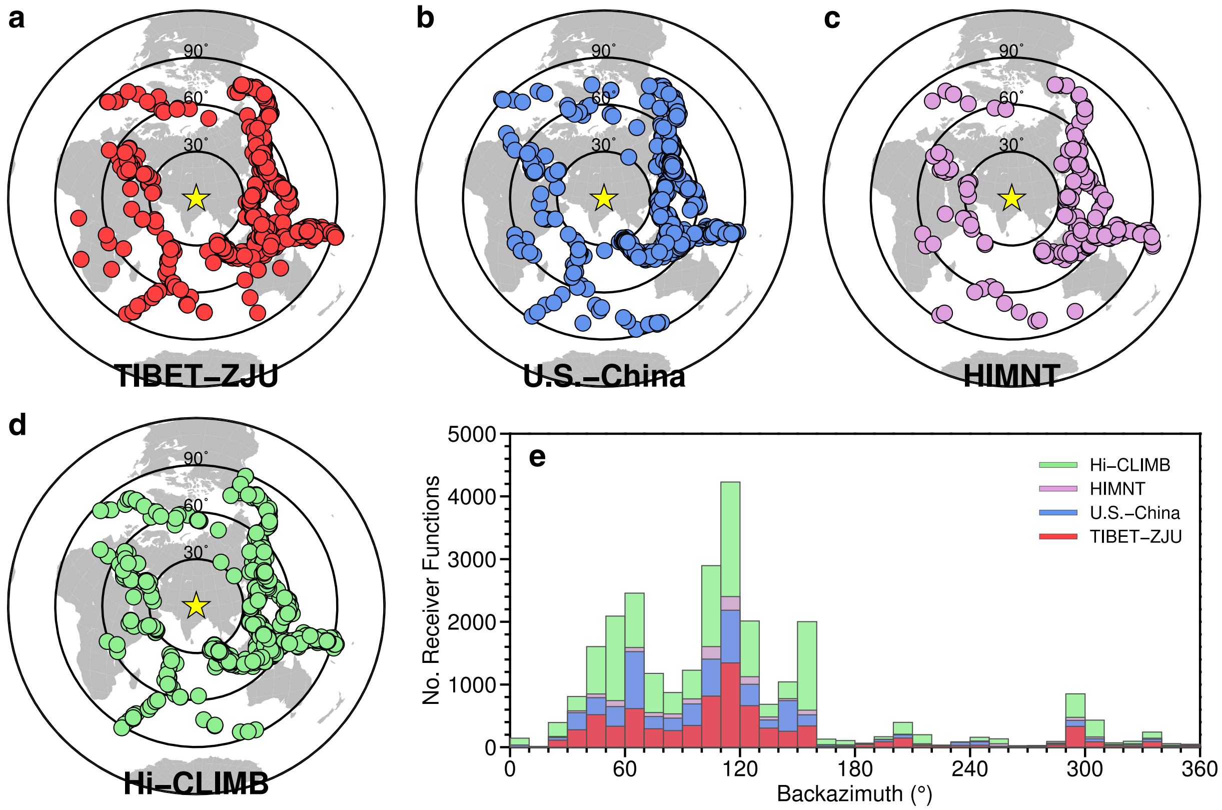

Supplementary Figure 7 | Azimuthal distribution of earthquakes and receiver functions used in the Pms moveout fitting. (a-d) Spatial distribution of earthquakes used in receiver-function study for different seismic experiments. **(e)** Histogram showing the backazimuthal distribution of the receiver functions.

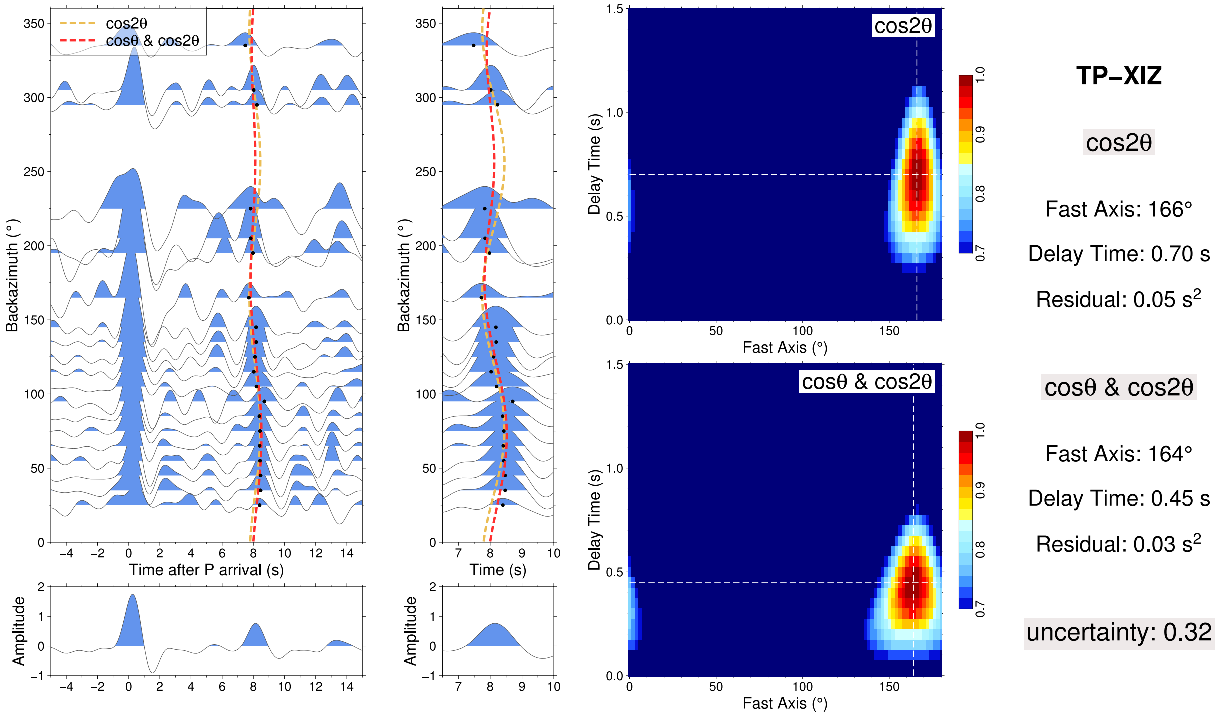

Supplementary Figure 8 | An example of Pms moveout fitting analysis for station TP-XIZ. Left: radial receiver functions plotted as a function of back azimuth and stacked receiver function. Arrival times of the Pms phases and the best-fit harmonic curves are indicated by black dots and dashed lines, respectively. **Right:** energy maps showing the optimal pair of parameters given by two harmonic fitting schemes.

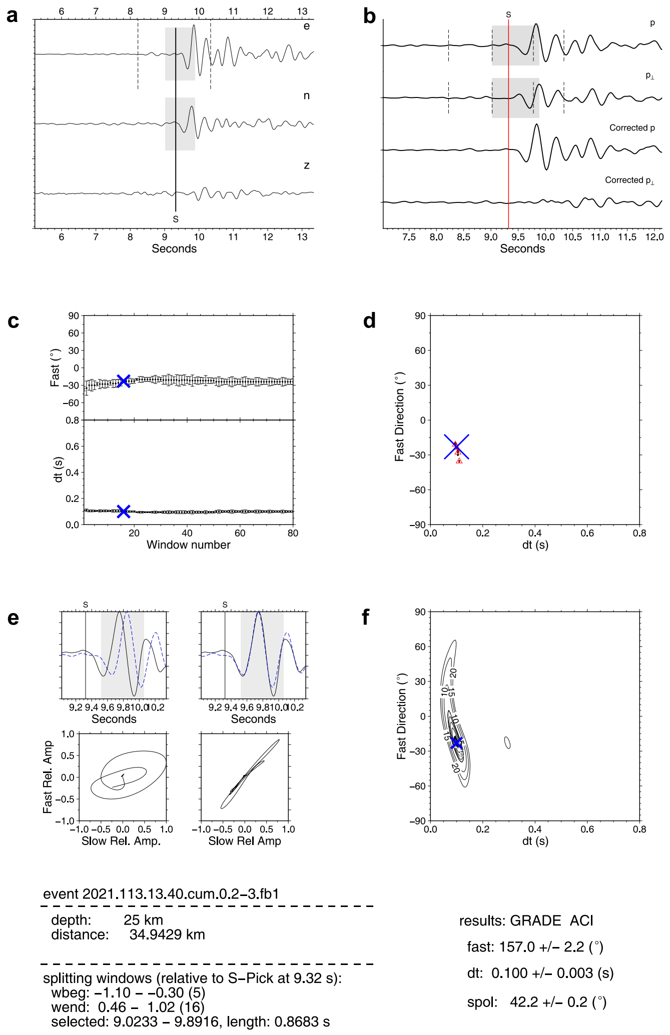

Supplementary Figure 9 | Local S-wave splitting measurement for event 2021:113:13:40 recorded at station CUM categorized as Quality A. (a) Filtered three-component waveforms. **(b)** Original and anisotropy-corrected radial (p) and transverse ($p_{\perp}$) waveforms. In panels (a) and (b), solid lines mark the S arrival; dashed lines show the range of start and end times of the analysis windows used in the processing; grey shading boxes indicate the window used for the final measurement. **(c-d)** Splitting parameters determined for each analysis window. The solutions are shown as black dots with the best one marked by blue crosses. Identified clusters are denoted by red triangles. **(e)** Original (left) and anisotropy-corrected (right) waveforms in the fast-slow coordinate system, as well as the corresponding particle motions. **(f)** Contour plot of the smallest eigenvalue of the covariance matrix for the final measurement.

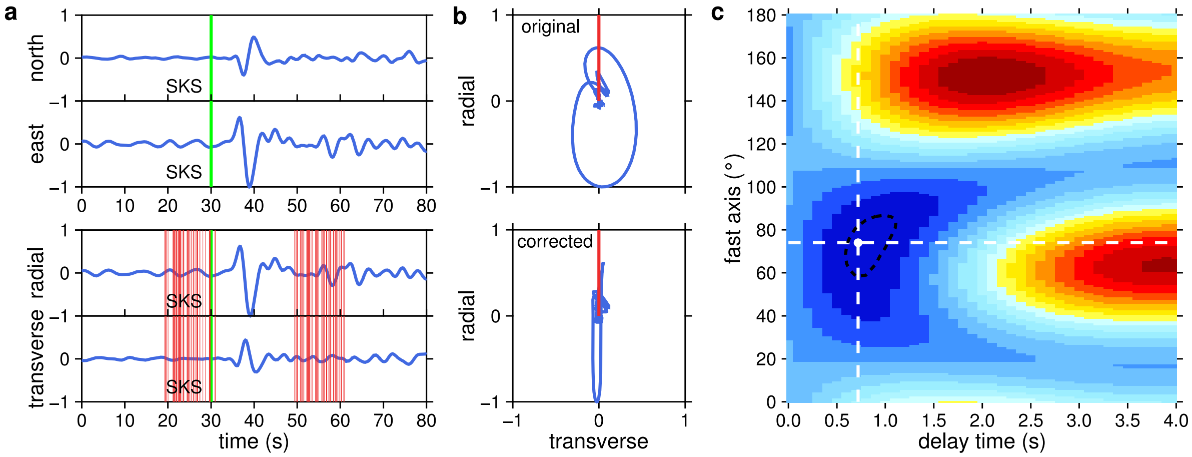

Supplementary Figure 10 | An example of S(K)KS splitting analysis for event 2019-12-04-20:10:03 recorded at station WUM categorized as Quality good. (a) Seismogram components in north, east, radial and transverse directions. Green and red solid lines show theoretical phase arrival and 50 different time windows used for the analysis, respectively. **(b)** Original and corrected particle motion patterns. **(c)** Energy grid for the corrected transverse component as a function of candidate fast axes and delay times. The white dot marks the optimal splitting parameters corresponding to the minimum energy. 95% confidence level are indicated by the black dashed contour line.

Supplementary References

1 Chen, M. *et al.* Lithospheric foundering and underthrusting imaged beneath Tibet. *Nature Communications* **8**, 15659, doi:10.1038/ncomms15659 (2017).
2 Bao, X., Song, X. & Li, J. High-resolution lithospheric structure beneath Mainland China from ambient noise and earthquake surface-wave tomography. *Earth and Planetary Science Letters* **417**, 132-141, doi:10.1016/j.epsl.2015.02.024 (2015).
3 Bian, S. *et al.* Along-strike variation in the initiation timing of the north-trending rifts in southern Tibet as revealed from the Yadong-Gulu rift. *Tectonics* **41**, e2021TC007091, doi:10.1029/2021tc007091 (2022).
4 Langille, J. M., Jessup, M. J., Cottle, J. M., Lederer, G. & Ahmad, T. Timing of metamorphism, melting and exhumation of the Leo Pargil dome, northwest India. *Journal of Metamorphic Geology* **30**, 769-791, doi:10.1111/j.1525-1314.2012.00998.x (2012).
5 Thiede, R. C. *et al.* Dome formation and extension in the Tethyan Himalaya, Leo Pargil, northwest India. *GSA Bulletin* **118**, 635-650, doi:10.1130/b25872.1 (2006).
6 Hintersberger, E., Thiede, R. C., Strecker, M. R. & Hacker, B. R. East-west extension in the NW Indian Himalaya. *GSA Bulletin* **122**, 1499-1515, doi:10.1130/b26589.1 (2010).
7 Murphy, M. A. & Copeland, P. Transtensional deformation in the central Himalaya and its role in accommodating growth of the Himalayan orogen. *Tectonics* **24**, TC4012, doi:10.1029/2004tc001659 (2005).
8 McCallister, A. T., Taylor, M. H., Murphy, M. A., Styron, R. H. & Stockli, D. F. Thermochronologic constraints on the late Cenozoic exhumation history of the Gurla Mandhata metamorphic core complex, Southwestern Tibet. *Tectonics* **33**, 27-52, doi:10.1002/2013tc003302 (2014).
9 Murphy, M. A. *et al.* Structural evolution of the Gurla Mandhata detachment system, southwest Tibet: Implications for the eastward extent of the Karakoram fault system. *GSA Bulletin* **114**, 428-447, doi:10.1130/0016-7606(2002)114<0428:seotgm>2.0.co;2 (2002).
10 Saylor, J. E. *et al.* The late Miocene through present paleoelevation history of southwestern Tibet. *American Journal of Science* **309**, 1-42, doi:10.2475/01.2009.01 (2009).
11 Saylor, J., DeCelles, P. & Quade, J. Climate-driven environmental change in the Zhada basin, southwestern Tibetan Plateau. *Geosphere* **6**, 74-92, doi:10.1130/ges00507.1 (2010).
12 Nagy, C., Godin, L., Antolín, B., Cottle, J. & Archibald, D. Mid-Miocene initiation of orogen-parallel extension, NW Nepal Himalaya. *Lithosphere* **7**, 483-502, doi:10.1130/l425.1 (2015).
13 Styron, R. H. *et al.* Miocene initiation and acceleration of extension in the South Lunggar rift, western Tibet: Evolution of an active detachment system from structural mapping and (U-Th)/He thermochronology. *Tectonics* **32**, 880-907, doi:10.1002/tect.20053 (2013).
14 Kapp, P., Taylor, M., Stockli, D. & Ding, L. Development of active low-angle normal fault systems during orogenic collapse: Insight from Tibet. *Geology* **36**, 7-10, doi:10.1130/g24054a.1 (2008).

15 Sundell, K. E. *et al.* Evidence for constriction and Pliocene acceleration of east-west extension in the North Lunggar rift region of west central Tibet. *Tectonics* **32**, 1454-1479, doi:10.1002/tect.20086 (2013).
16 Styron, R., Taylor, M. & Sundell, K. Accelerated extension of Tibet linked to the northward underthrusting of Indian crust. *Nature Geoscience* **8**, 131-134, doi:10.1038/ngeo2336 (2015).
17 Woodruff, W. H., Horton, B. K., Kapp, P. & Stockli, D. F. Late Cenozoic evolution of the Lunggar extensional basin, Tibet: Implications for basin growth and exhumation in hinterland plateaus. *GSA Bulletin* **125**, 343-358, doi:10.1130/b30664.1 (2013).
18 Larson, K. P., Kellett, D. A., Cottle, J. M., Camacho, A. & Brubacher, A. D. Mid-Miocene initiation of E-W extension and recoupling of the Himalaya. *Terra Nova* **32**, 151-158, doi:10.1111/ter.12443 (2019).
19 Coleman, M. & Hodges, K. Evidence for Tibetan plateau uplift before 14 Myr ago from a new minimum age for east–west extension. *Nature* **374**, 49-52, doi:10.1038/374049a0 (1995).
20 Garzione, C. N., Dettman, D. L., Quade, J., DeCelles, P. G. & Butler, R. F. High times on the Tibetan Plateau: Paleoelevation of the Thakkhola graben, Nepal. *Geology* **28**, 339-342, doi:10.1130/0091-7613(2000)28<339:htottp>2.0.co;2 (2000).
21 Garzione, C. N., DeCelles, P. G., Hodkinson, D. G., Ojha, T. P. & Upreti, B. N. East-west extension and Miocene environmental change in the southern Tibetan plateau: Thakkhola graben, central Nepal. *GSA Bulletin* **115**, 3-20, doi:10.1130/0016-7606(2003)115<0003:eweame>2.0.co;2 (2003).
22 Brubacher, A. D., Larson, K. P., Cottle, J. M., Matthews, W. & Camacho, A. Progressive development of E-W extension across the Tibetan plateau: A case study of the Thakkhola graben, west-central Nepal. *International Geology Review* **63**, 1900-1919, doi:10.1080/00206814.2020.1808860 (2021).
23 Sanchez, V. *et al.* in *American Geophysical Union Fall Meeting* (San Francisco, USA, 2010).
24 Sanchez, V. I., Murphy, M. A., Robinson, A. C., Lapen, T. J. & Heizler, M. T. Tectonic evolution of the India–Asia suture zone since Middle Eocene time, Lopukangri area, south-central Tibet. *Journal of Asian Earth Sciences* **62**, 205-220, doi:10.1016/j.jseaes.2012.09.004 (2013).
25 Murphy, M. A., Sanchez, V. & Taylor, M. H. Syncollisional extension along the India–Asia suture zone, south-central Tibet: Implications for crustal deformation of Tibet. *Earth and Planetary Science Letters* **290**, 233-243, doi:10.1016/j.epsl.2009.11.046 (2010).
26 Laskowski, A. K., Kapp, P., Ding, L., Campbell, C. & Liu, X. Tectonic evolution of the Yarlung suture zone, Lopu Range region, southern Tibet. *Tectonics* **36**, 108-136, doi:10.1002/2016tc004334 (2017).
27 Shen, T. *et al.* Controls on Cenozoic exhumation of the Tethyan Himalaya from fission-track thermochronology and detrital zircon U-Pb geochronology in the Gyirong basin area, southern Tibet. *Tectonics* **35**, 1713-1734, doi:10.1002/2016tc004149 (2016).
28 Burke, W. B. *et al.* Record of crustal thickening and synconvergent extension from the Dajiamang Tso rift, southern Tibet. *Geosciences* **11**, 209, doi:10.3390/geosciences11050209 (2021).

29 Lee, J. *et al.* Middle to late Miocene extremely rapid exhumation and thermal reequilibration in the Kung Co rift, southern Tibet. *Tectonics* **30**, TC2007, doi:10.1029/2010tc002745 (2011).

30 Maheo, G. *et al.* Post 4 Ma initiation of normal faulting in southern Tibet. Constraints from the Kung Co half graben. *Earth and Planetary Science Letters* **256**, 233-243, doi:10.1016/j.epsl.2007.01.029 (2007).

31 Mitsuishi, M., Wallis, S. R., Aoya, M., Lee, J. & Wang, Y. E-W extension at 19 Ma in the Kung Co area, S. Tibet: Evidence for contemporaneous E-W and N-S extension in the Himalayan orogen. *Earth and Planetary Science Letters* **325-326**, 10-20, doi:10.1016/j.epsl.2011.11.013 (2012).

32 Wolff, R. *et al.* High-angle normal faulting at the Tangra Yumco graben (southern Tibet) since ~15 Ma. *The Journal of Geology* **127**, 15-36, doi:10.1086/700406 (2019).

33 Dewane, T. J. *et al.* in *American Geophysical Union Fall Meeting* (San Francisco, USA, 2006).

34 Wolff, R. *et al.* Rift propagation in south Tibet controlled by under-thrusting of India: a case study of the Tangra Yumco graben (south Tibet). *Journal of the Geological Society* **180**, jgs2022-2090, doi:10.1144/jgs2022-090 (2023).

35 Zhang, J. & Guo, L. Structure and geochronology of the southern Xainza-Dinggye rift and its relationship to the south Tibetan detachment system. *Journal of Asian Earth Sciences* **29**, 722-736, doi:10.1016/j.jseaes.2006.05.003 (2007).

36 Kali, E. *et al.* Exhumation history of the deepest central Himalayan rocks, Ama Drime range: Key pressure-temperature-deformation-time constraints on orogenic models. *Tectonics* **29**, TC2014, doi:10.1029/2009tc002551 (2010).

37 Hager, C., Stockli, D. F., Dewane, T. J., Gehrels, G. & Ding, L. in *EGU General Assembly 2009* (Vienna, Austria, 2009).

38 Blisniuk, P. M. *et al.* Normal faulting in central Tibet since at least 13.5 Myr ago. *Nature* **412**, 628-632, doi:10.1038/35088045 (2001).

39 Yin, A. *et al.* Significant late Neogene east-west extension in northern Tibet. *Geology* **27**, 787-790, doi:10.1130/0091-7613(1999)027<0787:slnewe>2.3.co;2 (1999).

40 Edwards, M. A. & Harrison, T. M. When did the roof collapse? Late Miocene north-south extension in the high Himalaya revealed by Th-Pb monazite dating of the Khula Kangri granite. *Geology* **25**, 543-546, doi:10.1130/0091-7613(1997)025<0543:WDTRCL>2.3.CO;2 (1997).

41 Ratschbacher, L. *et al.* Rifting and strike–slip shear in central Tibet and the geometry, age and kinematics of upper crustal extension in Tibet. *Geological Society, London, Special Publications* **353**, 127-163, doi:10.1144/sp353.8 (2011).

42 Xu, Z. *et al.* Orogen-parallel ductile extension and extrusion of the Greater Himalaya in the late Oligocene and Miocene. *Tectonics* **32**, 191-215, doi:10.1002/tect.20021 (2013).

43 Dong, H. *et al.* Timing of slip across the South Tibetan detachment system and Yadong–Gulu graben, Eastern Himalaya. *Journal of the Geological Society* **178**, jgs2019-2197, doi:10.1144/jgs2019-197 (2020).

44 Cooper, F. J., Hodges, K. V., Parrish, R. R., Roberts, N. M. W. & Horstwood, M. S. A. Synchronous N-S and E-W extension at the Tibet-to-Himalaya transition in NW Bhutan. *Tectonics* **34**, 1375-1395, doi:10.1002/2014tc003712 (2015).

45 Ha, G., Wu, Z., He, L. & Wang, S. Late Cenozoic sedimentary evolution of Pagri-Duoqing Co graben, southern end of Yadong-Gulu rift, southern Tibet. *Acta Geologica Sinica - English Edition* **92**, 972-987, doi:10.1111/1755-6724.13586 (2018).
46 Wang, S. *et al.* Illite K-Ar and (U-Th)/He low-temperature thermochronology reveal onset timing of Yadong-Gulu rift in southern Tibetan Plateau. *Frontiers in Earth Science* **10**, 993796, doi:10.3389/feart.2022.993796 (2022).
47 Harrison, T. M., Copeland, P., Kidd, W. S. F. & Lovera, O. M. Activation of the Nyainqentanghla Shear Zone: Implications for uplift of the southern Tibetan Plateau. *Tectonics* **14**, 658-676, doi:10.1029/95tc00608 (1995).
48 Kapp, J. L. D. A. *et al.* Nyainqentanglha Shan: A window into the tectonic, thermal, and geochemical evolution of the Lhasa block, southern Tibet. *Journal of Geophysical Research* **110**, B08413, doi:10.1029/2004jb003330 (2005).
49 Wu, Z., Hu, d., Liu, Q., Xia, H. & Yan, X. The formation and evolution of tectonic landform of Damxung area in central Tibetan Plateau. *Acta Geoscientica Sinica* **23**, 423-428 (2002).
50 Stockli, D. F. *et al.* in *Proceedings of the Geological Society of America* (Denver, USA, 2002).
51 Bian, S. *et al.* Late Pliocene onset of the Cona rift, eastern Himalaya, confirms eastward propagation of extension in Himalayan-Tibetan orogen. *Earth and Planetary Science Letters* **544**, 116383, doi:10.1016/j.epsl.2020.116383 (2020).
52 Cao, H. *et al.* Miocene Sn polymetallic mineralization in the Tethyan Himalaya, southeastern Tibet: A case study of the Cuonadong deposit. *Ore Geology Reviews* **119**, 103403, doi:10.1016/j.oregeorev.2020.103403 (2020).
53 Shen, T. *et al.* Impacts of late Miocene normal faulting on Yarlung Tsangpo River evolution, southeastern Tibet. *GSA Bulletin* **134**, 3142-3154, doi:10.1130/b36210.1 (2022).
