# Supplementary material for: Southern Tibetan rifting since late Miocene enabled by basal shear of the underthrusting Indian lithosphere
Source: Nat Commun. 2023 May 4;14:2565. doi: 10.1038/s41467-023-38296-w (PMC10160080; doi:10.1038/s41467-023-38296-w)
Supplement: Supplementary file 2 — Description of Additional Supplementary Files [file 41467_2023_38296_MOESM2_ESM.pdf]

Description of Additional Supplementary Files

File Name: Supplementary Data 1
Description: Summary of initiation and acceleration timing of E-W extension across Tibet, modified from Bian, et al.[3].

File Name: Supplementary Data 2
Description: List of Pms moveout fitting measurements at individual stations.

File Name: Supplementary Data 3
Description: Similar to Supplementary Fig. 1b but for 171 Pms moveout fitting measurements that pass all selection criteria.

File Name: Supplementary Data 4
Description: Similar to Supplementary Fig. 8 but for 243 Pms moveout fitting measurements that have sufficient azimuthal coverage.

File Name: Supplementary Data 5
Description: List of local S-wave splitting measurements at individual stations in the categories of A and B.

File Name: Supplementary Data 6
Description: Similar to Supplementary Fig. 9 but for 35 local S-wave splitting measurements in the categories of A and B.

File Name: Supplementary Data 7
Description: List of S(K)KS splitting measurements at individual stations in the categories of good and average.

File Name: Supplementary Data 8
Description: Similar to Supplementary Fig. 10 but for 591 S(K)KS splitting measurements in the categories of good and average.
