# Supplementary figures and images for: Southern Tibetan rifting since late Miocene enabled by basal shear of the underthrusting Indian lithosphere (part 2 of 4)
Source: Nat Commun. 2023 May 4;14:2565. doi: 10.1038/s41467-023-38296-w (PMC10160080; doi:10.1038/s41467-023-38296-w)

# XF-H1210

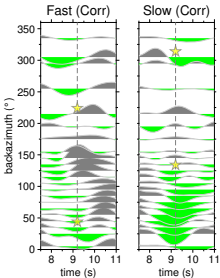

Supplement: Supplementary file 5 — Supplementary Data 3 [file 41467_2023_38296_MOESM5_ESM.zip › XF-H1210.pdf]

## XF-H1220

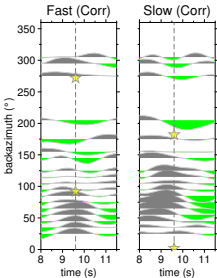

Supplement: Supplementary file 5 — Supplementary Data 3 [file 41467_2023_38296_MOESM5_ESM.zip › XF-H1220.pdf]

## XF-H1230

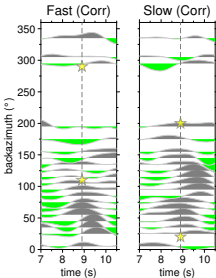

Supplement: Supplementary file 5 — Supplementary Data 3 [file 41467_2023_38296_MOESM5_ESM.zip › XF-H1230.pdf]

# XF-H1250

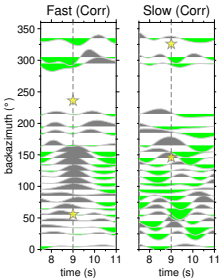

Supplement: Supplementary file 5 — Supplementary Data 3 [file 41467_2023_38296_MOESM5_ESM.zip › XF-H1250.pdf]

# XF-H1270

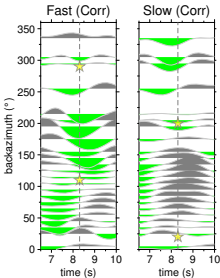

Supplement: Supplementary file 5 — Supplementary Data 3 [file 41467_2023_38296_MOESM5_ESM.zip › XF-H1270.pdf]

# XF-H1280

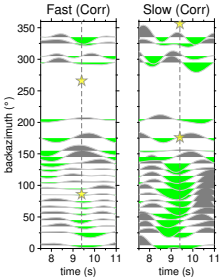

Supplement: Supplementary file 5 — Supplementary Data 3 [file 41467_2023_38296_MOESM5_ESM.zip › XF-H1280.pdf]

# XF-H1310

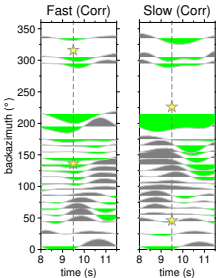

Supplement: Supplementary file 5 — Supplementary Data 3 [file 41467_2023_38296_MOESM5_ESM.zip › XF-H1310.pdf]

# XF-H1330

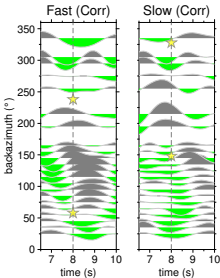

Supplement: Supplementary file 5 — Supplementary Data 3 [file 41467_2023_38296_MOESM5_ESM.zip › XF-H1330.pdf]

# XF-H1340

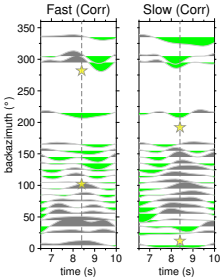

Supplement: Supplementary file 5 — Supplementary Data 3 [file 41467_2023_38296_MOESM5_ESM.zip › XF-H1340.pdf]

## XF-H1350

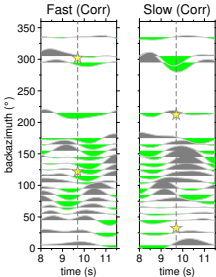

Supplement: Supplementary file 5 — Supplementary Data 3 [file 41467_2023_38296_MOESM5_ESM.zip › XF-H1350.pdf]

## XF-H1405

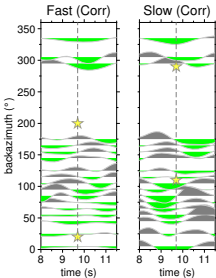

Supplement: Supplementary file 5 — Supplementary Data 3 [file 41467_2023_38296_MOESM5_ESM.zip › XF-H1405.pdf]

## XF-H1420

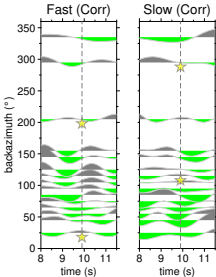

Supplement: Supplementary file 5 — Supplementary Data 3 [file 41467_2023_38296_MOESM5_ESM.zip › XF-H1420.pdf]

# XF-H1422

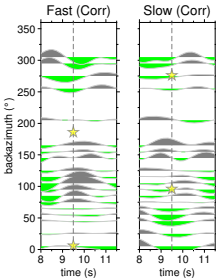

Supplement: Supplementary file 5 — Supplementary Data 3 [file 41467_2023_38296_MOESM5_ESM.zip › XF-H1422.pdf]

## XF-H1470

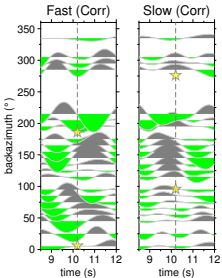

Supplement: Supplementary file 5 — Supplementary Data 3 [file 41467_2023_38296_MOESM5_ESM.zip › XF-H1470.pdf]

# XF-H1480

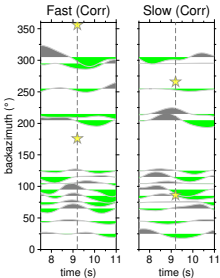

Supplement: Supplementary file 5 — Supplementary Data 3 [file 41467_2023_38296_MOESM5_ESM.zip › XF-H1480.pdf]

# XF-H1500

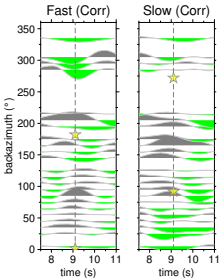

Supplement: Supplementary file 5 — Supplementary Data 3 [file 41467_2023_38296_MOESM5_ESM.zip › XF-H1500.pdf]

## XF-H1520

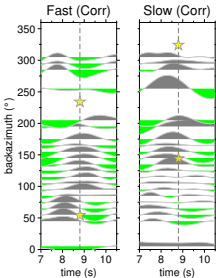

Supplement: Supplementary file 5 — Supplementary Data 3 [file 41467_2023_38296_MOESM5_ESM.zip › XF-H1520.pdf]

# XF-H1530

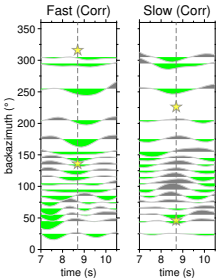

Supplement: Supplementary file 5 — Supplementary Data 3 [file 41467_2023_38296_MOESM5_ESM.zip › XF-H1530.pdf]

# XF-H1550

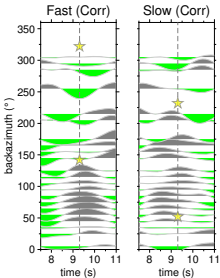

Supplement: Supplementary file 5 — Supplementary Data 3 [file 41467_2023_38296_MOESM5_ESM.zip › XF-H1550.pdf]

# XF-H1560

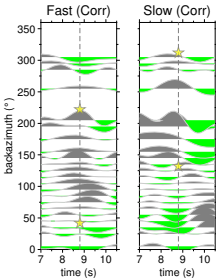

Supplement: Supplementary file 5 — Supplementary Data 3 [file 41467_2023_38296_MOESM5_ESM.zip › XF-H1560.pdf]

## XF-H1570

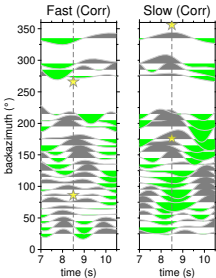

Supplement: Supplementary file 5 — Supplementary Data 3 [file 41467_2023_38296_MOESM5_ESM.zip › XF-H1570.pdf]

# XF-H1590

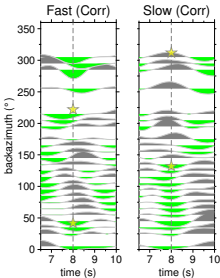

Supplement: Supplementary file 5 — Supplementary Data 3 [file 41467_2023_38296_MOESM5_ESM.zip › XF-H1590.pdf]

## XF-H1600

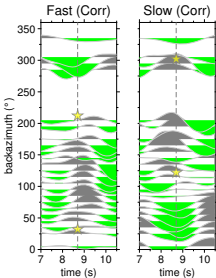

Supplement: Supplementary file 5 — Supplementary Data 3 [file 41467_2023_38296_MOESM5_ESM.zip › XF-H1600.pdf]

# XF-H1610

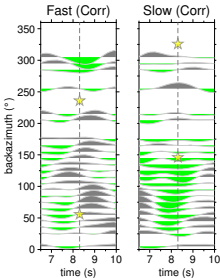

Supplement: Supplementary file 5 — Supplementary Data 3 [file 41467_2023_38296_MOESM5_ESM.zip › XF-H1610.pdf]

## XF-H1620

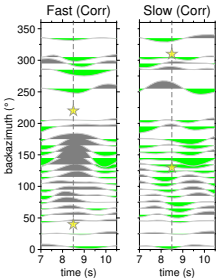

Supplement: Supplementary file 5 — Supplementary Data 3 [file 41467_2023_38296_MOESM5_ESM.zip › XF-H1620.pdf]

## XF-NBUNG

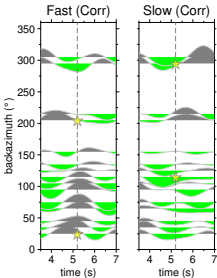

Supplement: Supplementary file 5 — Supplementary Data 3 [file 41467_2023_38296_MOESM5_ESM.zip › XF-NBUNG.pdf]

## XF-NG060

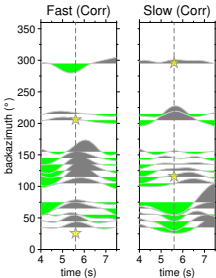

Supplement: Supplementary file 5 — Supplementary Data 3 [file 41467_2023_38296_MOESM5_ESM.zip › XF-NG060.pdf]

## XF-NP010

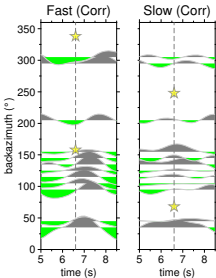

Supplement: Supplementary file 5 — Supplementary Data 3 [file 41467_2023_38296_MOESM5_ESM.zip › XF-NP010.pdf]

## XF-NP030

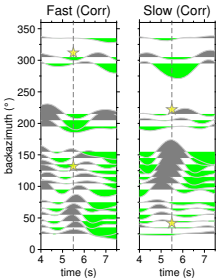

Supplement: Supplementary file 5 — Supplementary Data 3 [file 41467_2023_38296_MOESM5_ESM.zip › XF-NP030.pdf]

## XF-NP050

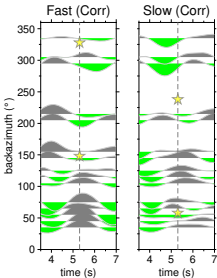

Supplement: Supplementary file 5 — Supplementary Data 3 [file 41467_2023_38296_MOESM5_ESM.zip › XF-NP050.pdf]

## XF-NP085

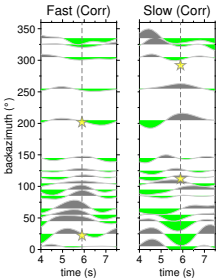

Supplement: Supplementary file 5 — Supplementary Data 3 [file 41467_2023_38296_MOESM5_ESM.zip › XF-NP085.pdf]

## XF-NRUMJ

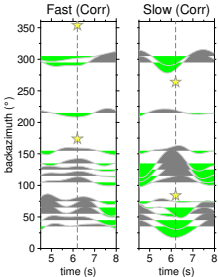

Supplement: Supplementary file 5 — Supplementary Data 3 [file 41467_2023_38296_MOESM5_ESM.zip › XF-NRUMJ.pdf]

## XF-NSIND

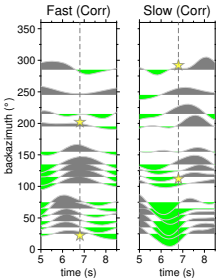

Supplement: Supplementary file 5 — Supplementary Data 3 [file 41467_2023_38296_MOESM5_ESM.zip › XF-NSIND.pdf]

## Y2-GARY

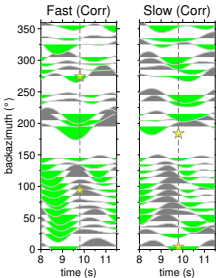

Supplement: Supplementary file 5 — Supplementary Data 3 [file 41467_2023_38296_MOESM5_ESM.zip › Y2-GARY.pdf]

## Y2-GUGE

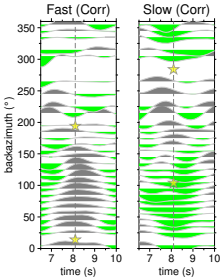

Supplement: Supplementary file 5 — Supplementary Data 3 [file 41467_2023_38296_MOESM5_ESM.zip › Y2-GUGE.pdf]

## Y2-MONS

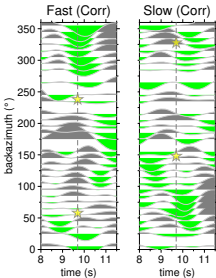

Supplement: Supplementary file 5 — Supplementary Data 3 [file 41467_2023_38296_MOESM5_ESM.zip › Y2-MONS.pdf]

## Y2-NOMA

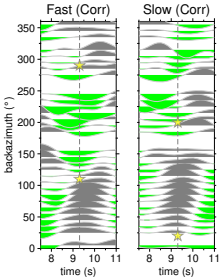

Supplement: Supplementary file 5 — Supplementary Data 3 [file 41467_2023_38296_MOESM5_ESM.zip › Y2-NOMA.pdf]

## Y2-NPUK

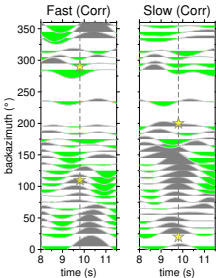

Supplement: Supplementary file 5 — Supplementary Data 3 [file 41467_2023_38296_MOESM5_ESM.zip › Y2-NPUK.pdf]

## Y2-RUTK

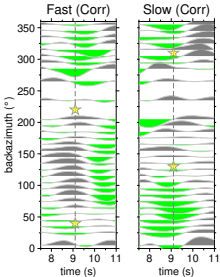

Supplement: Supplementary file 5 — Supplementary Data 3 [file 41467_2023_38296_MOESM5_ESM.zip › Y2-RUTK.pdf]

## Y2-SQAH

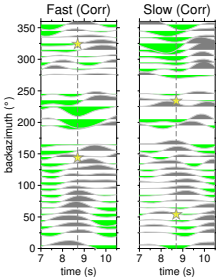

Supplement: Supplementary file 5 — Supplementary Data 3 [file 41467_2023_38296_MOESM5_ESM.zip › Y2-SQAH.pdf]

## Y2-WT01

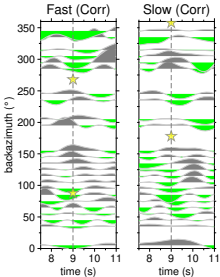

Supplement: Supplementary file 5 — Supplementary Data 3 [file 41467_2023_38296_MOESM5_ESM.zip › Y2-WT01.pdf]

## Y2-WT02

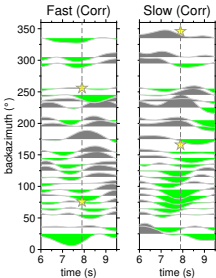

Supplement: Supplementary file 5 — Supplementary Data 3 [file 41467_2023_38296_MOESM5_ESM.zip › Y2-WT02.pdf]

## Y2-WT03

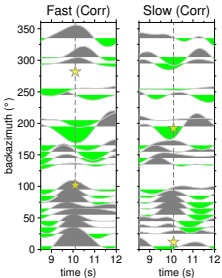

Supplement: Supplementary file 5 — Supplementary Data 3 [file 41467_2023_38296_MOESM5_ESM.zip › Y2-WT03.pdf]

## Y2-WT07

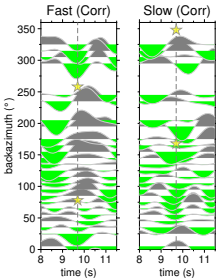

Supplement: Supplementary file 5 — Supplementary Data 3 [file 41467_2023_38296_MOESM5_ESM.zip › Y2-WT07.pdf]

## Y2-WT08

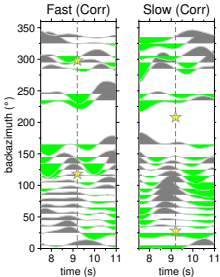

Supplement: Supplementary file 5 — Supplementary Data 3 [file 41467_2023_38296_MOESM5_ESM.zip › Y2-WT08.pdf]

## Y2-WT09

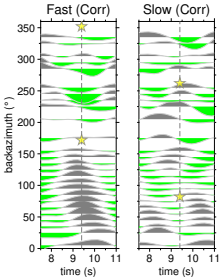

Supplement: Supplementary file 5 — Supplementary Data 3 [file 41467_2023_38296_MOESM5_ESM.zip › Y2-WT09.pdf]

## Y2-WT11

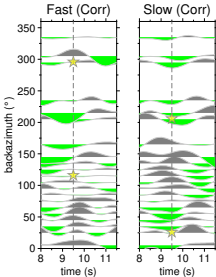

Supplement: Supplementary file 5 — Supplementary Data 3 [file 41467_2023_38296_MOESM5_ESM.zip › Y2-WT11.pdf]

## Y2-WT12

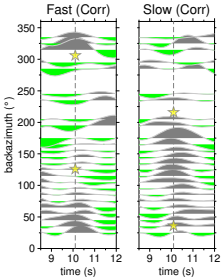

Supplement: Supplementary file 5 — Supplementary Data 3 [file 41467_2023_38296_MOESM5_ESM.zip › Y2-WT12.pdf]

## Y2-WT13

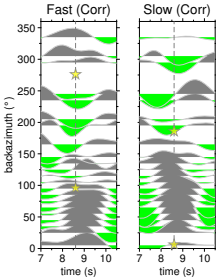

Supplement: Supplementary file 5 — Supplementary Data 3 [file 41467_2023_38296_MOESM5_ESM.zip › Y2-WT13.pdf]

## Y2-WT14

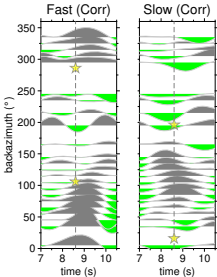

Supplement: Supplementary file 5 — Supplementary Data 3 [file 41467_2023_38296_MOESM5_ESM.zip › Y2-WT14.pdf]

## Y2-WT15

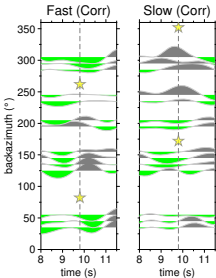

Supplement: Supplementary file 5 — Supplementary Data 3 [file 41467_2023_38296_MOESM5_ESM.zip › Y2-WT15.pdf]

## Y2-WT16

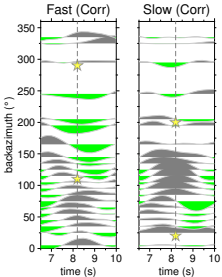

Supplement: Supplementary file 5 — Supplementary Data 3 [file 41467_2023_38296_MOESM5_ESM.zip › Y2-WT16.pdf]

## Y2-WT19

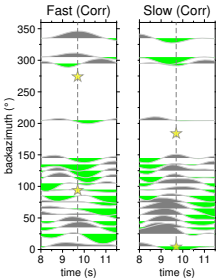

Supplement: Supplementary file 5 — Supplementary Data 3 [file 41467_2023_38296_MOESM5_ESM.zip › Y2-WT19.pdf]

## Y2-WT20

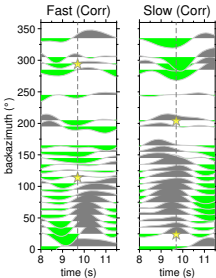

Supplement: Supplementary file 5 — Supplementary Data 3 [file 41467_2023_38296_MOESM5_ESM.zip › Y2-WT20.pdf]

## Y2-ZMBA

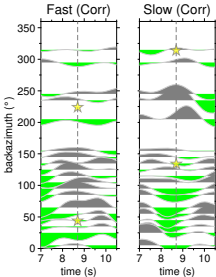

Supplement: Supplementary file 5 — Supplementary Data 3 [file 41467_2023_38296_MOESM5_ESM.zip › Y2-ZMBA.pdf]

## YL-BUNG

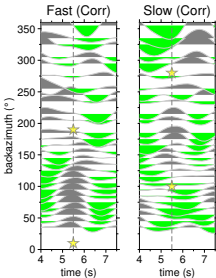

Supplement: Supplementary file 5 — Supplementary Data 3 [file 41467_2023_38296_MOESM5_ESM.zip › YL-BUNG.pdf]

# YL-DINX

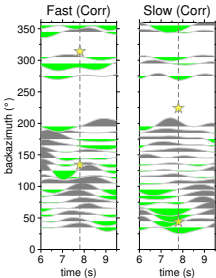

Supplement: Supplementary file 5 — Supplementary Data 3 [file 41467_2023_38296_MOESM5_ESM.zip › YL-DINX.pdf]

# YL-HILE

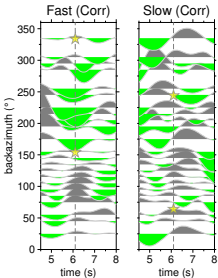

Supplement: Supplementary file 5 — Supplementary Data 3 [file 41467_2023_38296_MOESM5_ESM.zip › YL-HILE.pdf]

# YL-JANA

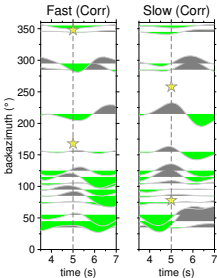

Supplement: Supplementary file 5 — Supplementary Data 3 [file 41467_2023_38296_MOESM5_ESM.zip › YL-JANA.pdf]

# YL-NAIL

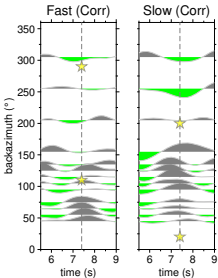

Supplement: Supplementary file 5 — Supplementary Data 3 [file 41467_2023_38296_MOESM5_ESM.zip › YL-NAIL.pdf]

## YL-PHAP

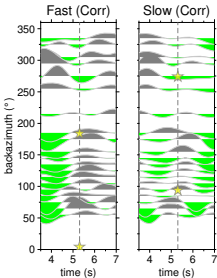

Supplement: Supplementary file 5 — Supplementary Data 3 [file 41467_2023_38296_MOESM5_ESM.zip › YL-PHAP.pdf]

## YL-PHID

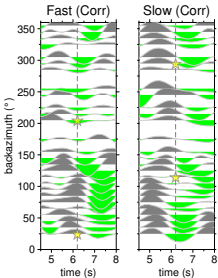

Supplement: Supplementary file 5 — Supplementary Data 3 [file 41467_2023_38296_MOESM5_ESM.zip › YL-PHID.pdf]

# YL-RUMJ

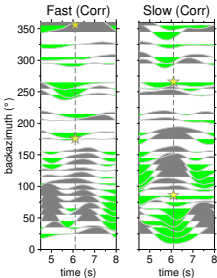

Supplement: Supplementary file 5 — Supplementary Data 3 [file 41467_2023_38296_MOESM5_ESM.zip › YL-RUMJ.pdf]

## YL-SAGA

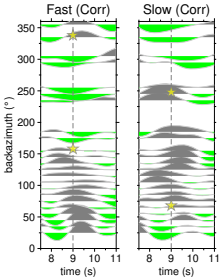

Supplement: Supplementary file 5 — Supplementary Data 3 [file 41467_2023_38296_MOESM5_ESM.zip › YL-SAGA.pdf]

# YL-SAJA

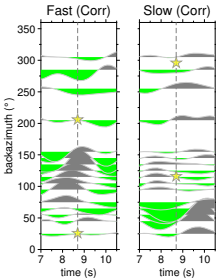

Supplement: Supplementary file 5 — Supplementary Data 3 [file 41467_2023_38296_MOESM5_ESM.zip › YL-SAJA.pdf]

## YL-SIND

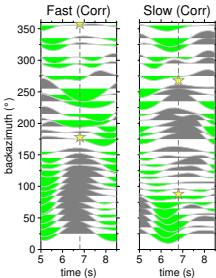

Supplement: Supplementary file 5 — Supplementary Data 3 [file 41467_2023_38296_MOESM5_ESM.zip › YL-SIND.pdf]

## YL-SSAN

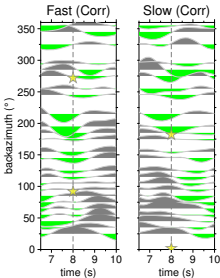

Supplement: Supplementary file 5 — Supplementary Data 3 [file 41467_2023_38296_MOESM5_ESM.zip › YL-SSAN.pdf]

# YL-SUKT

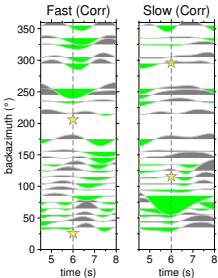

Supplement: Supplementary file 5 — Supplementary Data 3 [file 41467_2023_38296_MOESM5_ESM.zip › YL-SUKT.pdf]

# YL-THAK

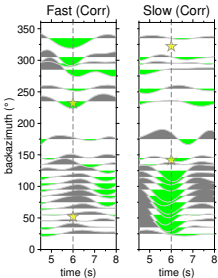

Supplement: Supplementary file 5 — Supplementary Data 3 [file 41467_2023_38296_MOESM5_ESM.zip › YL-THAK.pdf]

# YL-TUML

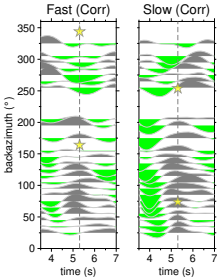

Supplement: Supplementary file 5 — Supplementary Data 3 [file 41467_2023_38296_MOESM5_ESM.zip › YL-TUML.pdf]

# YL-XIXI

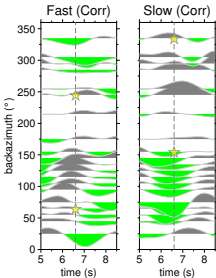

Supplement: Supplementary file 5 — Supplementary Data 3 [file 41467_2023_38296_MOESM5_ESM.zip › YL-XIXI.pdf]

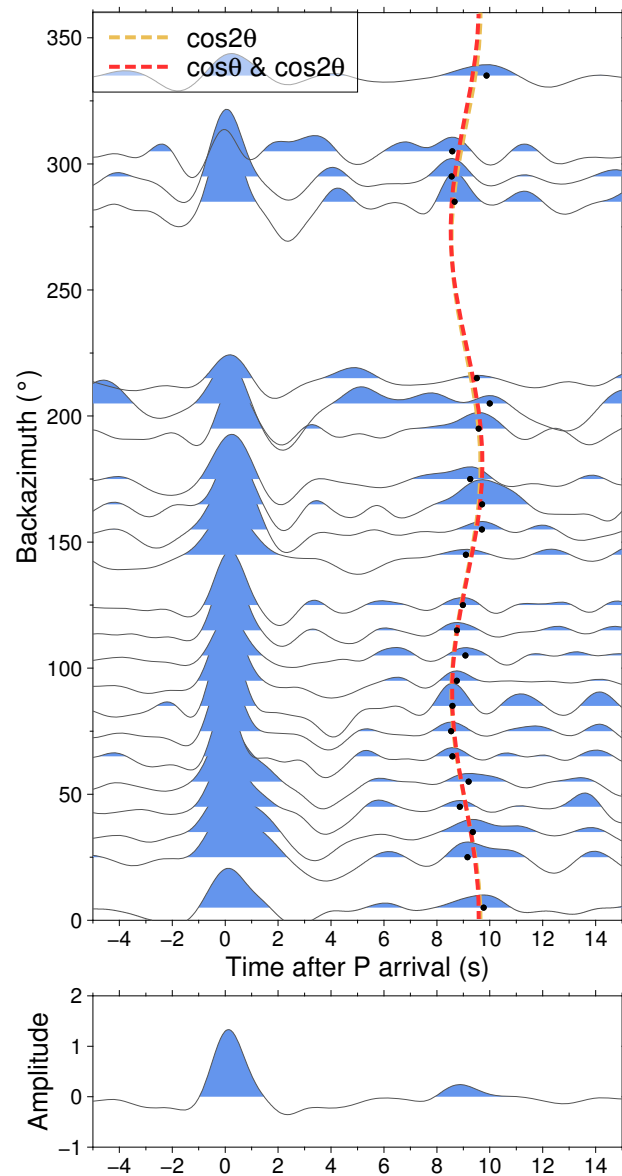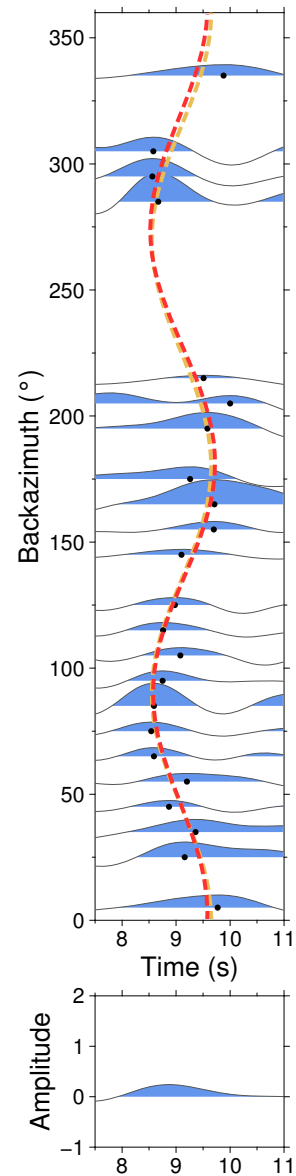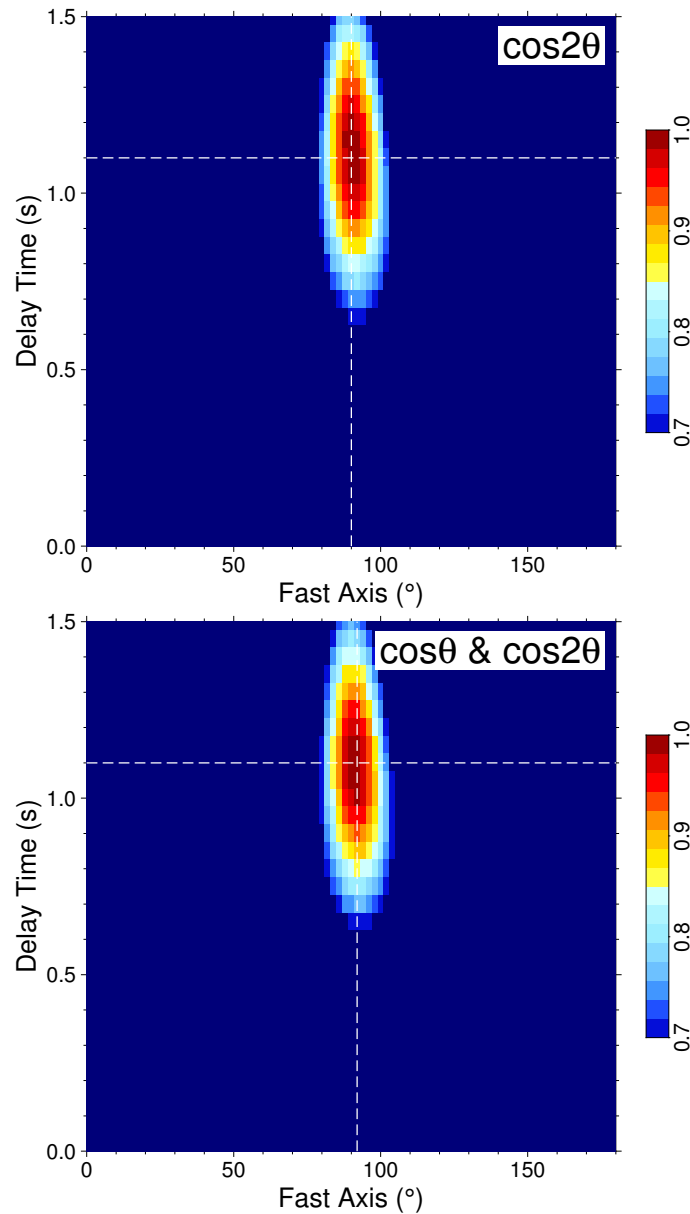

Supplement: Supplementary file 6 — Supplementary Data 4 [file 41467_2023_38296_MOESM6_ESM.zip › strike-slip_XF-H1500.pdf]

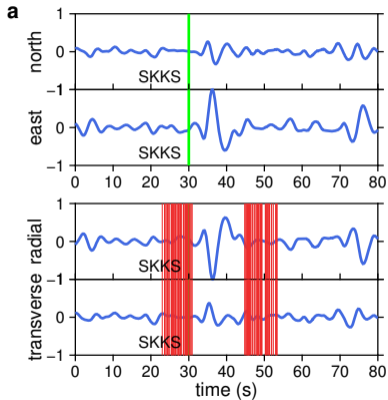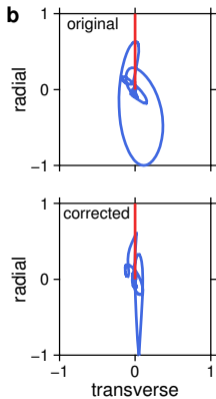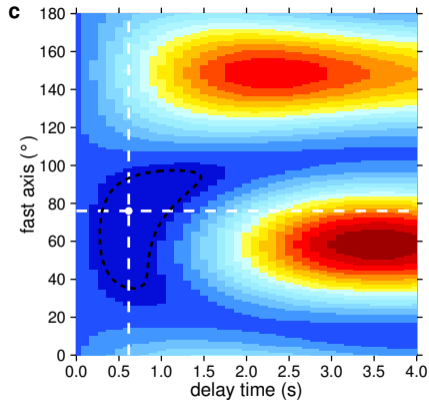

Supplement: Supplementary file 10 — Supplementary Data 8 [file 41467_2023_38296_MOESM10_ESM.zip › TP_BAG_01-Nov-2018_22_19_51_SKKS_average.pdf]

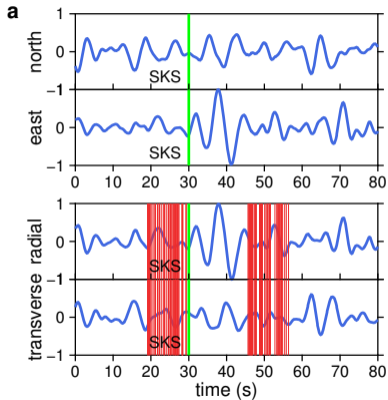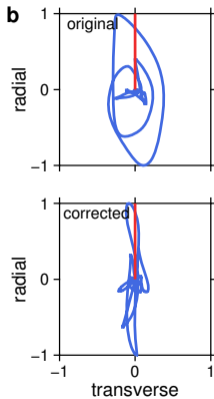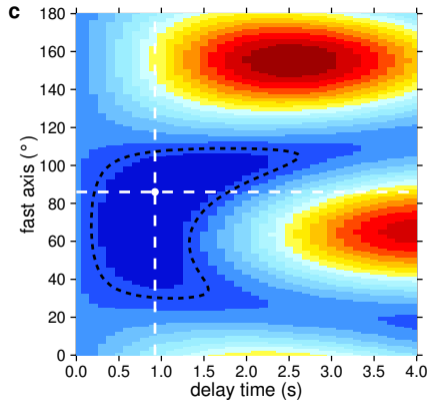

Supplement: Supplementary file 10 — Supplementary Data 8 [file 41467_2023_38296_MOESM10_ESM.zip › TP_BAG_26-Jun-2019_18_06_30_SKS_average.pdf]

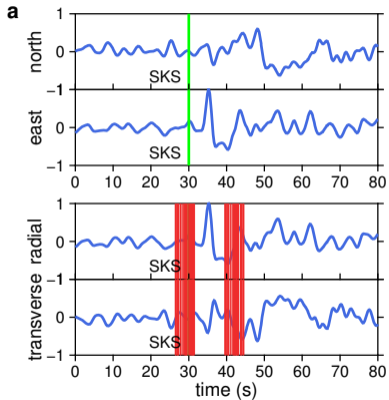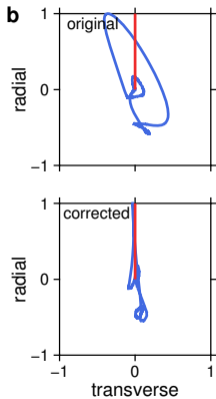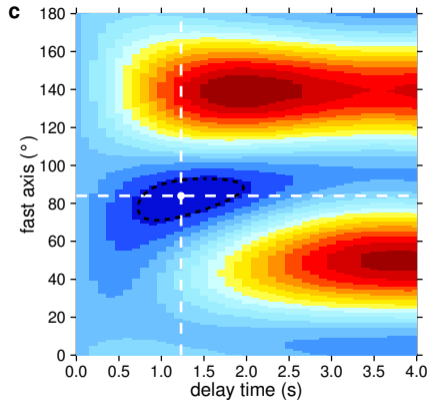

Supplement: Supplementary file 10 — Supplementary Data 8 [file 41467_2023_38296_MOESM10_ESM.zip › TP_BAG_30-May-2019_15_38_01_SKS_average.pdf]

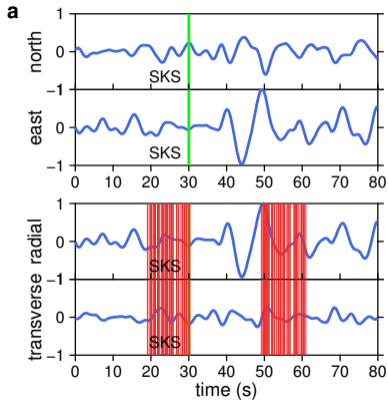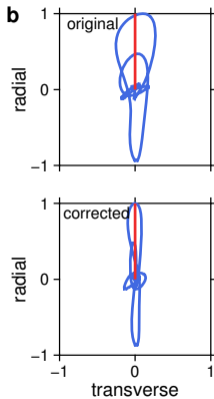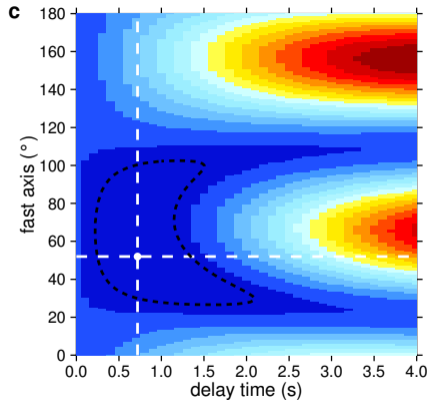

Supplement: Supplementary file 10 — Supplementary Data 8 [file 41467_2023_38296_MOESM10_ESM.zip › TP_BUD_10-Feb-2021_18_36_46_SKS_average.pdf]

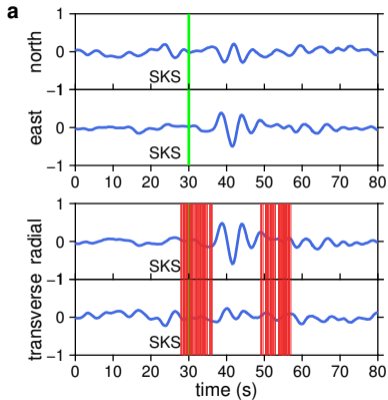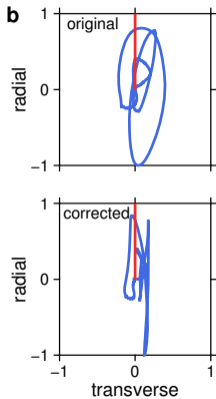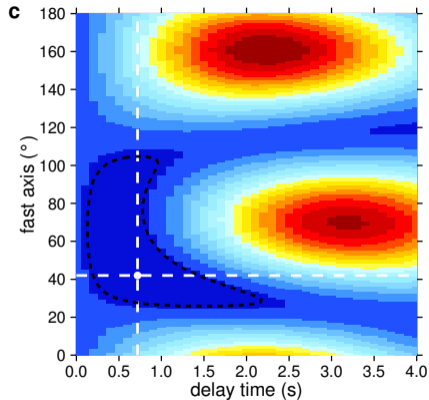

Supplement: Supplementary file 10 — Supplementary Data 8 [file 41467_2023_38296_MOESM10_ESM.zip › TP_CAK_02-Jul-2019_04_07_26_SKS_average.pdf]

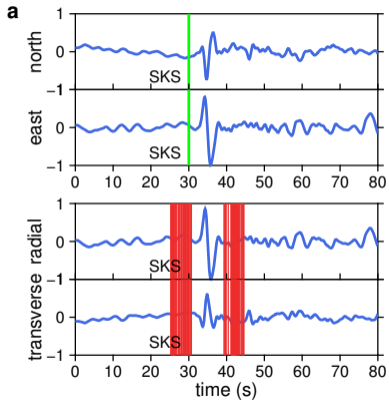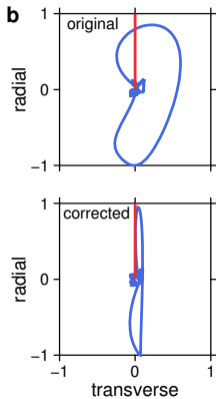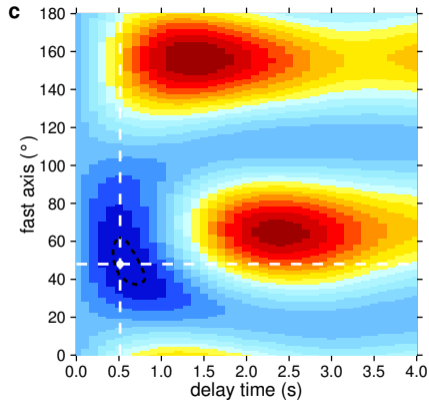

Supplement: Supplementary file 10 — Supplementary Data 8 [file 41467_2023_38296_MOESM10_ESM.zip › TP_CAK_06-Aug-2019_22_14_14_SKS_good.pdf]

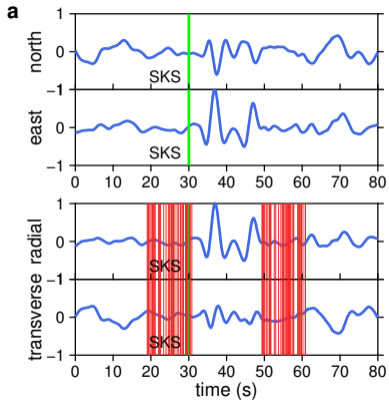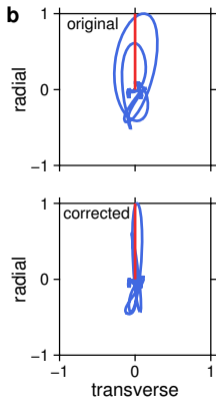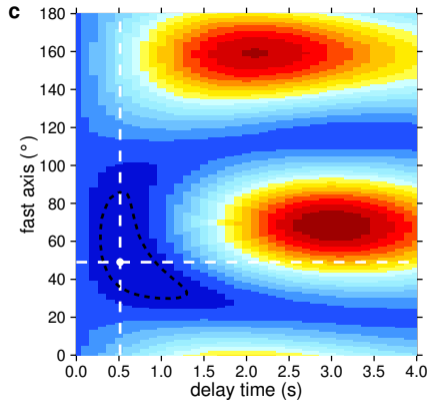

Supplement: Supplementary file 10 — Supplementary Data 8 [file 41467_2023_38296_MOESM10_ESM.zip › TP_CAK_06-Dec-2018_23_26_59_SKS_average.pdf]

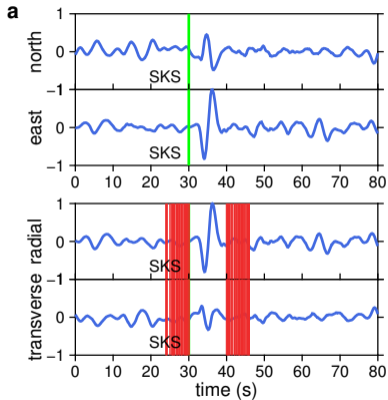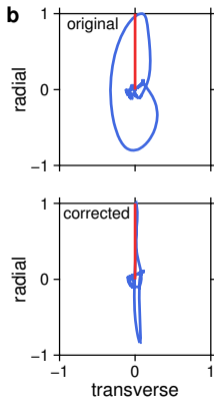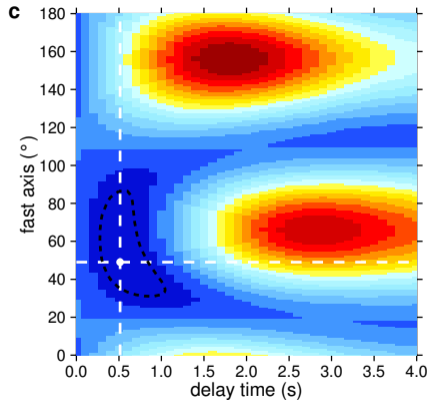

Supplement: Supplementary file 10 — Supplementary Data 8 [file 41467_2023_38296_MOESM10_ESM.zip › TP_CAK_06-Jul-2019_11_08_16_SKS_average.pdf]

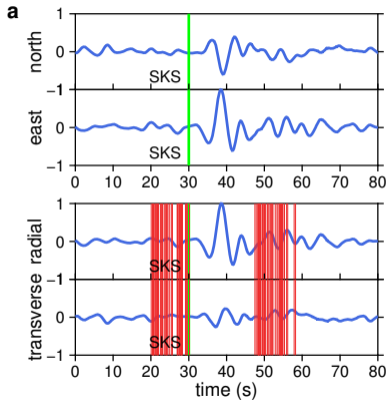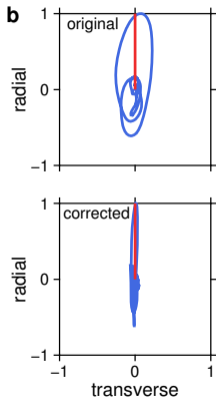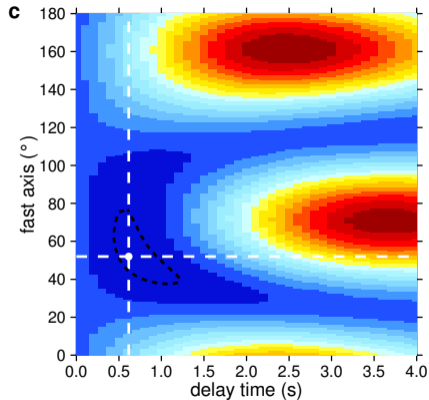

Supplement: Supplementary file 10 — Supplementary Data 8 [file 41467_2023_38296_MOESM10_ESM.zip › TP_CAK_06-Mar-2019_20_19_59_SKS_good.pdf]

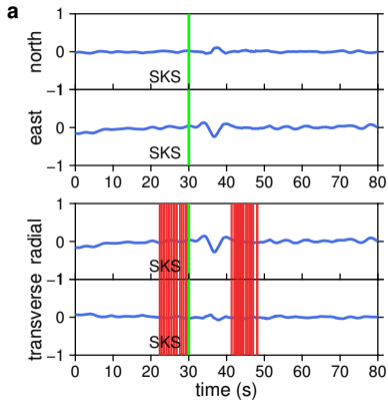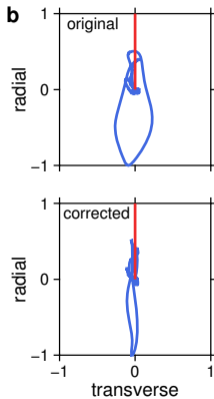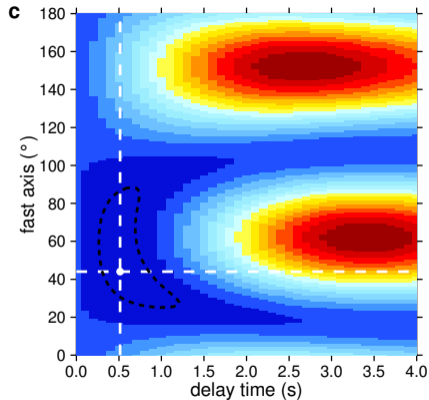

Supplement: Supplementary file 10 — Supplementary Data 8 [file 41467_2023_38296_MOESM10_ESM.zip › TP_CAK_07-Aug-2019_05_32_40_SKS_average.pdf]

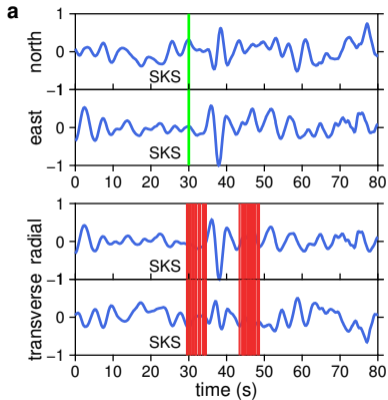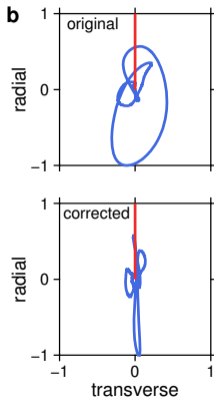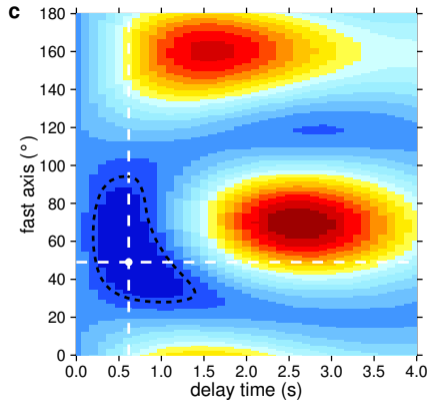

Supplement: Supplementary file 10 — Supplementary Data 8 [file 41467_2023_38296_MOESM10_ESM.zip › TP_CAK_07-Oct-2018_17_42_21_SKS_average.pdf]

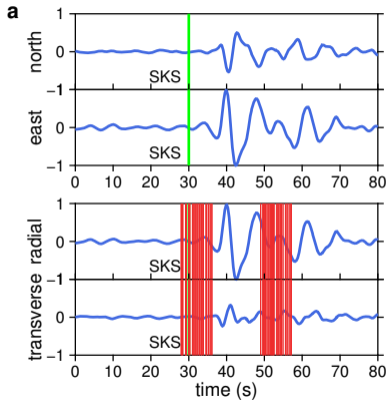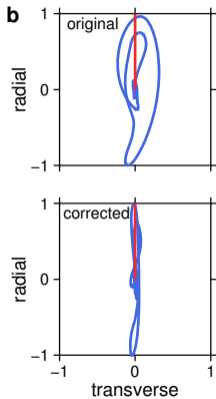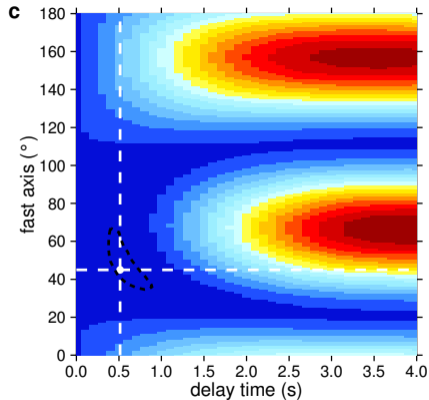

Supplement: Supplementary file 10 — Supplementary Data 8 [file 41467_2023_38296_MOESM10_ESM.zip › TP_CAK_16-Oct-2018_00_28_13_SKS_good.pdf]

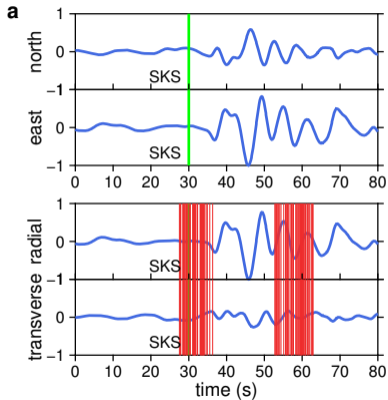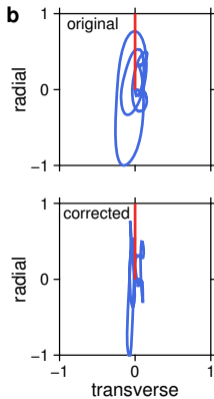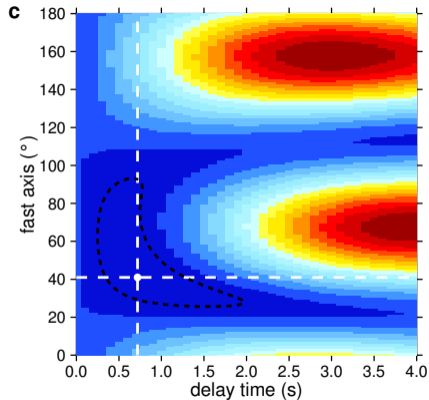

Supplement: Supplementary file 10 — Supplementary Data 8 [file 41467_2023_38296_MOESM10_ESM.zip › TP_CAK_16-Oct-2018_01_03_43_SKS_average.pdf]

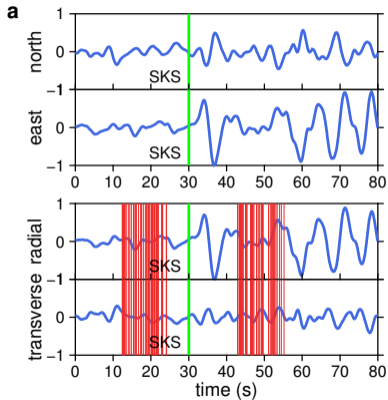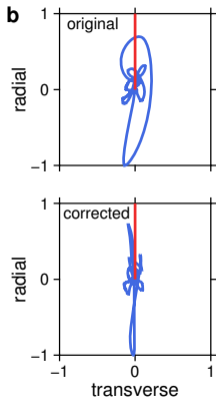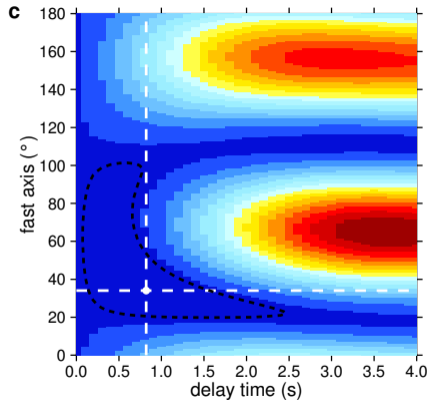

Supplement: Supplementary file 10 — Supplementary Data 8 [file 41467_2023_38296_MOESM10_ESM.zip › TP_CAK_18-Jan-2019_13_18_31_SKS_average.pdf]

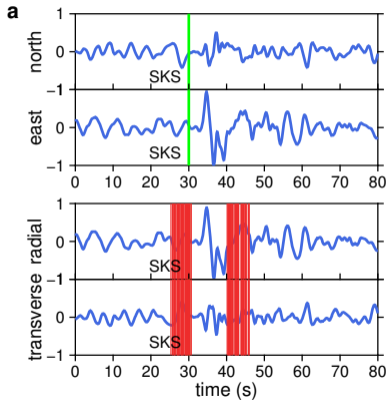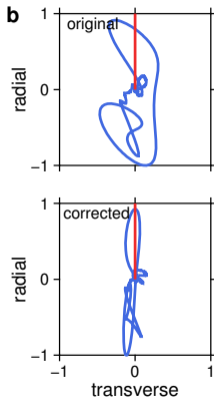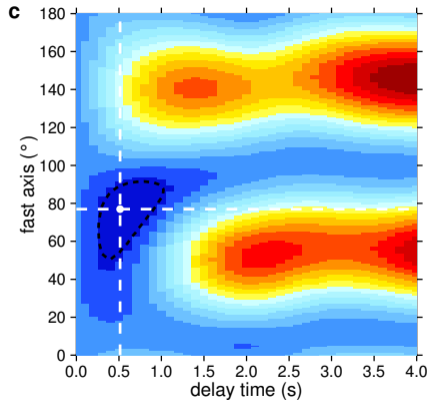

Supplement: Supplementary file 10 — Supplementary Data 8 [file 41467_2023_38296_MOESM10_ESM.zip › TP_CAK_18-Nov-2018_20_25_46_SKS_average.pdf]

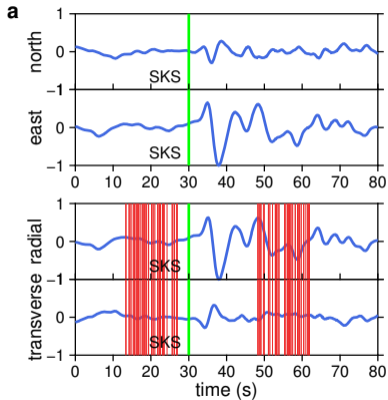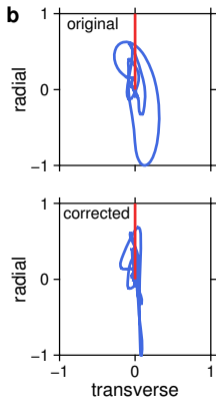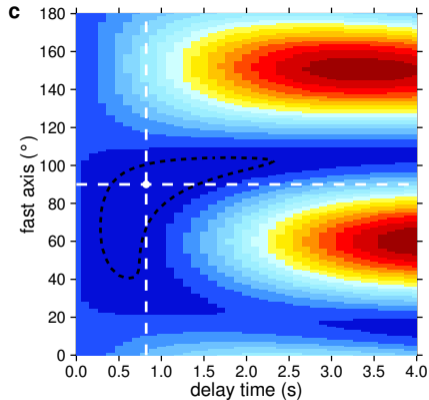

Supplement: Supplementary file 10 — Supplementary Data 8 [file 41467_2023_38296_MOESM10_ESM.zip › TP_CAK_19-May-2019_14_27_11_SKS_average.pdf]

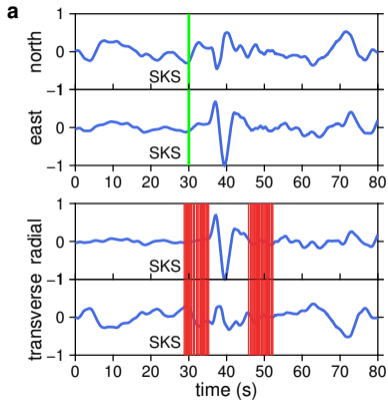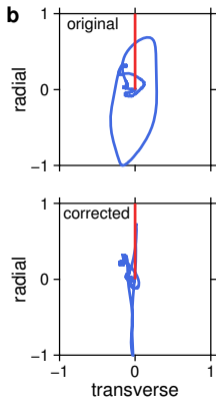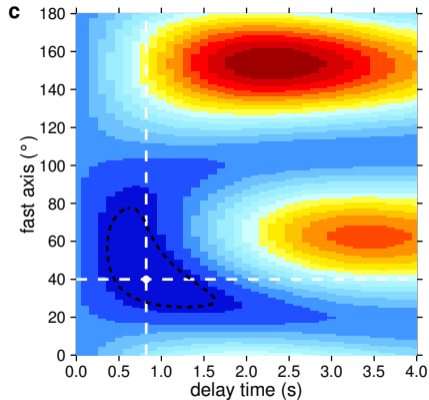

Supplement: Supplementary file 10 — Supplementary Data 8 [file 41467_2023_38296_MOESM10_ESM.zip › TP_CAK_20-Mar-2019_15_24_00_SKS_average.pdf]

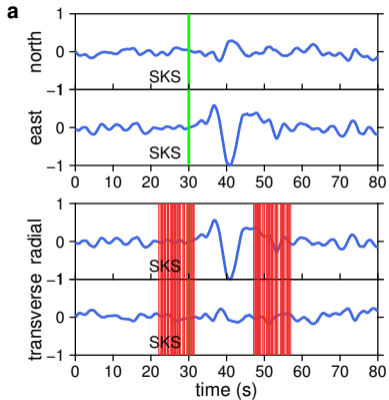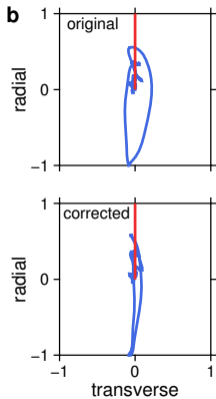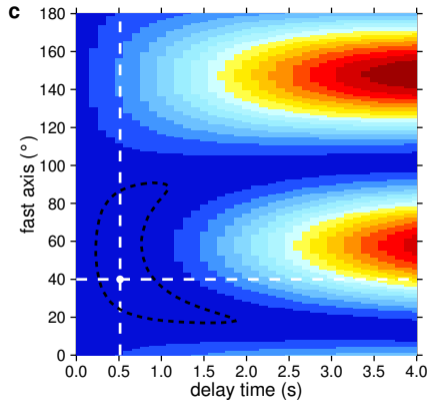

Supplement: Supplementary file 10 — Supplementary Data 8 [file 41467_2023_38296_MOESM10_ESM.zip › TP_CAK_30-Sep-2018_10_52_23_SKS_average.pdf]

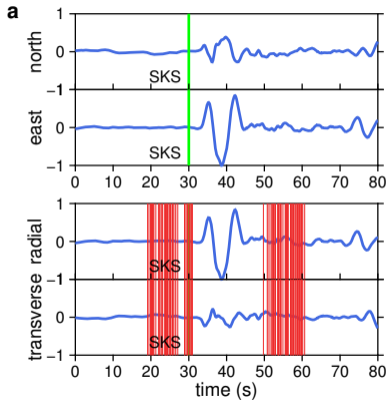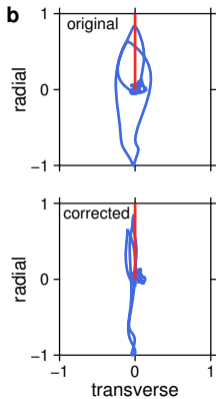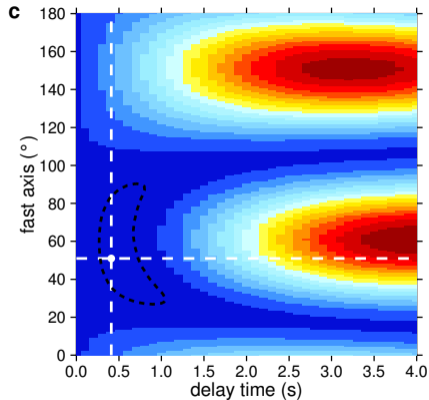

Supplement: Supplementary file 10 — Supplementary Data 8 [file 41467_2023_38296_MOESM10_ESM.zip › TP_CAK_31-Jul-2019_15_02_33_SKS_average.pdf]

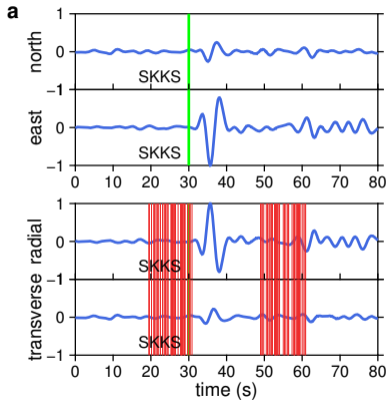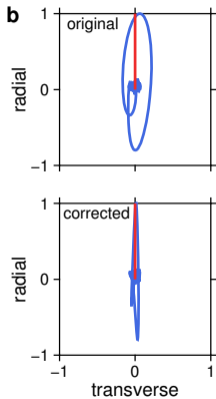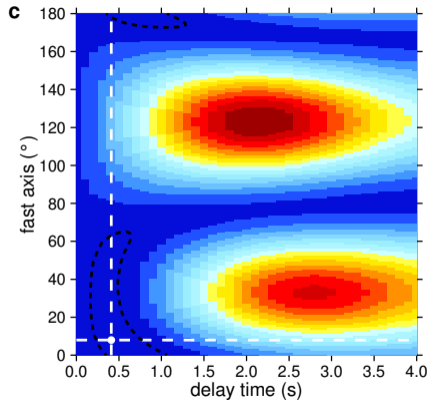

Supplement: Supplementary file 10 — Supplementary Data 8 [file 41467_2023_38296_MOESM10_ESM.zip › TP_CHG_04-Nov-2019_21_53_25_SKKS_average.pdf]

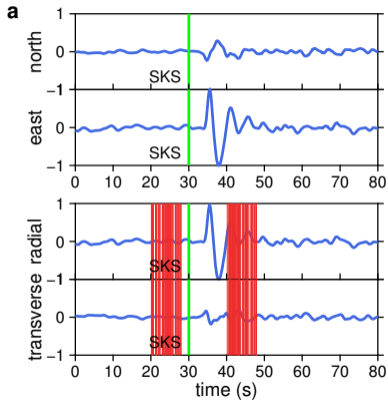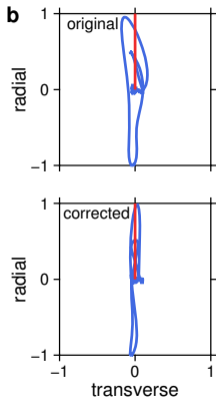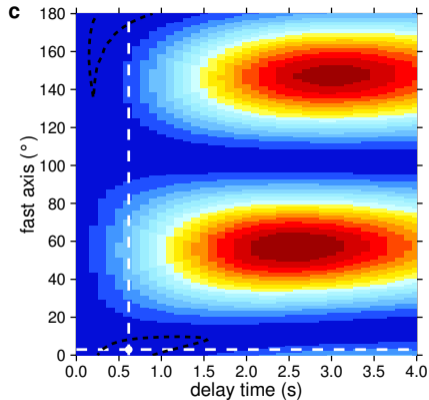

Supplement: Supplementary file 10 — Supplementary Data 8 [file 41467_2023_38296_MOESM10_ESM.zip › TP_CHG_06-Oct-2020_10_11_45_SKS_average.pdf]

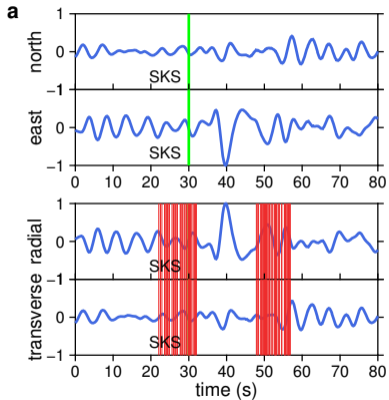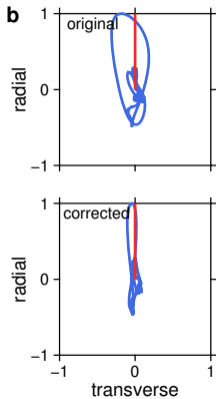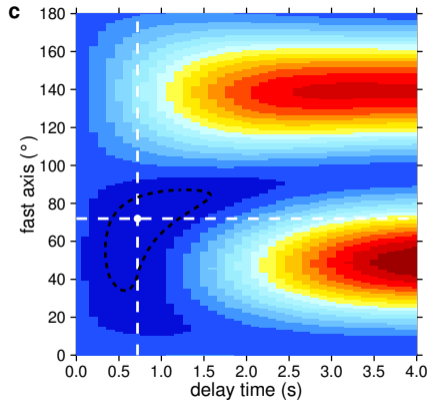

Supplement: Supplementary file 10 — Supplementary Data 8 [file 41467_2023_38296_MOESM10_ESM.zip › TP_CHG_16-Jan-2020_09_58_19_SKS_average.pdf]

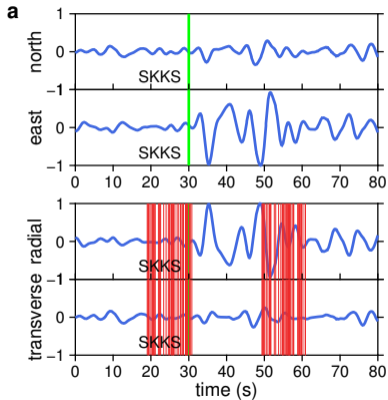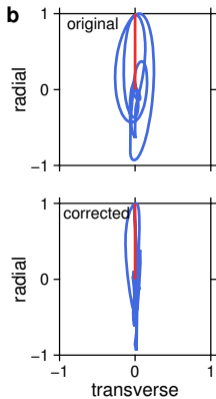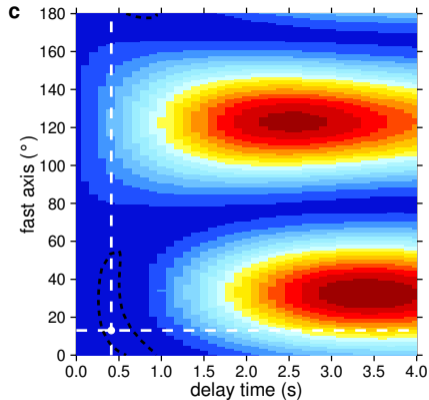

Supplement: Supplementary file 10 — Supplementary Data 8 [file 41467_2023_38296_MOESM10_ESM.zip › TP_CHG_19-Jan-2021_02_46_20_SKKS_average.pdf]

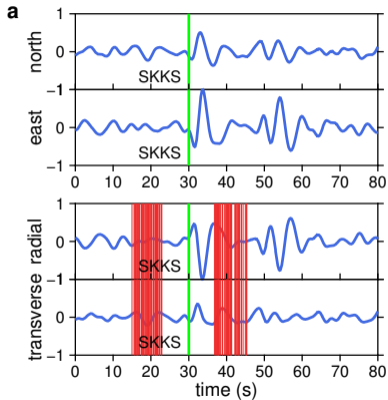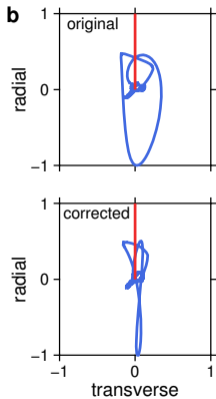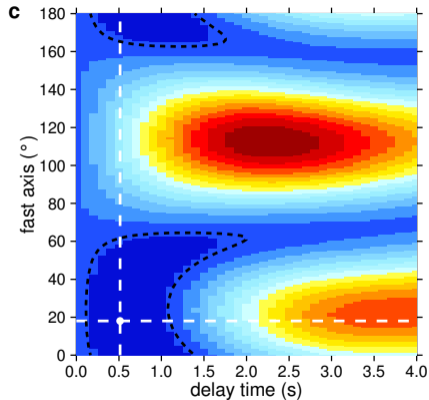

Supplement: Supplementary file 10 — Supplementary Data 8 [file 41467_2023_38296_MOESM10_ESM.zip › TP_CHG_22-Nov-2020_00_54_54_SKKS_average.pdf]

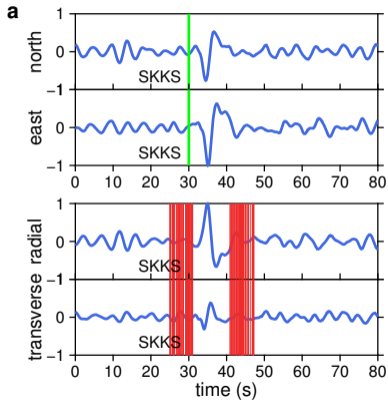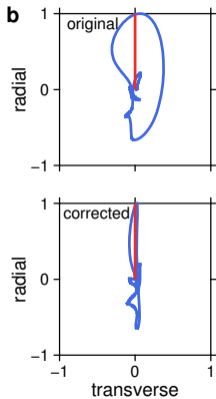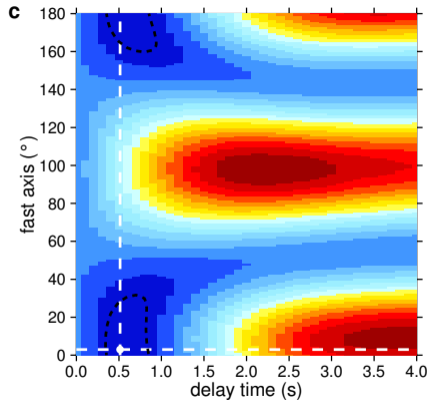

Supplement: Supplementary file 10 — Supplementary Data 8 [file 41467_2023_38296_MOESM10_ESM.zip › TP_CHG_26-Sep-2019_16_36_18_SKKS_good.pdf]

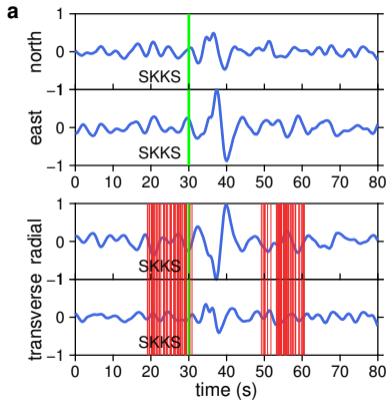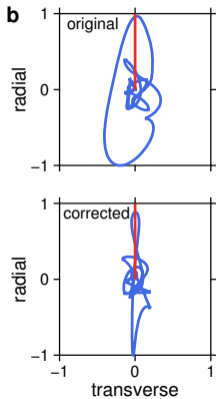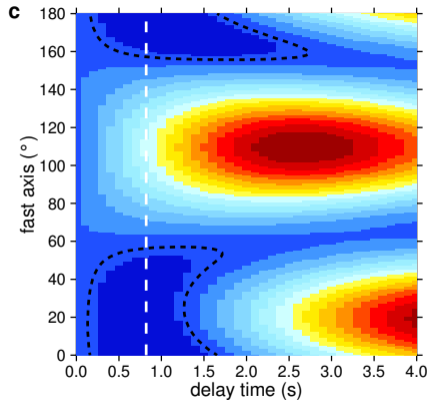

Supplement: Supplementary file 10 — Supplementary Data 8 [file 41467_2023_38296_MOESM10_ESM.zip › TP_CHG_29-Apr-2021_02_17_16_SKKS_average.pdf]

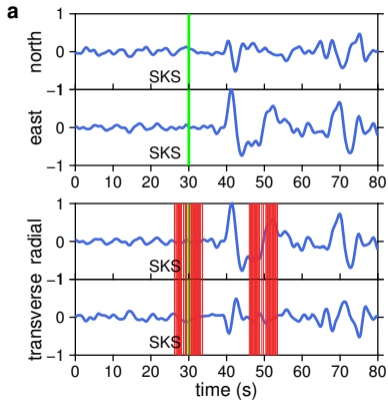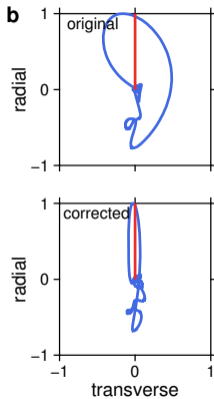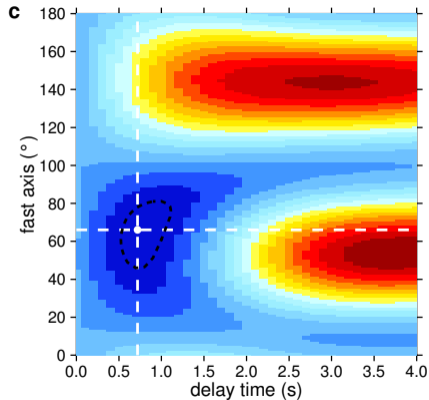

Supplement: Supplementary file 10 — Supplementary Data 8 [file 41467_2023_38296_MOESM10_ESM.zip › TP_CIS_01-Oct-2020_01_13_41_SKS_good.pdf]

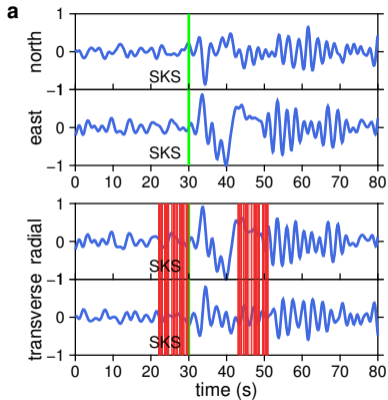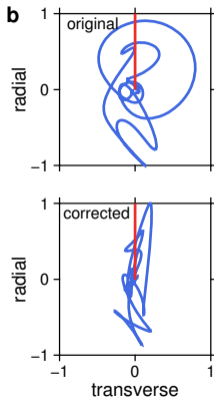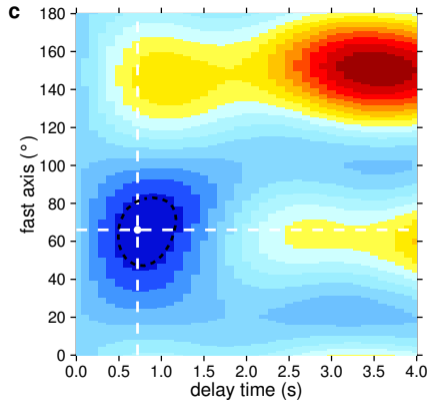

Supplement: Supplementary file 10 — Supplementary Data 8 [file 41467_2023_38296_MOESM10_ESM.zip › TP_CIS_01-Sep-2019_15_54_20_SKS_average.pdf]
