# Supplementary figures and images for: Southern Tibetan rifting since late Miocene enabled by basal shear of the underthrusting Indian lithosphere (part 1 of 4)
Source: Nat Commun. 2023 May 4;14:2565. doi: 10.1038/s41467-023-38296-w (PMC10160080; doi:10.1038/s41467-023-38296-w)

## TP-BAE

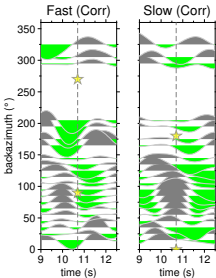

Supplement: Supplementary file 5 — Supplementary Data 3 [file 41467_2023_38296_MOESM5_ESM.zip › TP-BAE.pdf]

## TP-BAG

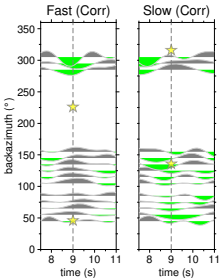

Supplement: Supplementary file 5 — Supplementary Data 3 [file 41467_2023_38296_MOESM5_ESM.zip › TP-BAG.pdf]

## TP-BUD

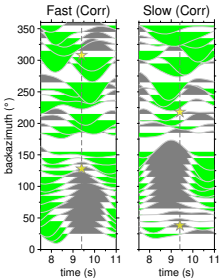

Supplement: Supplementary file 5 — Supplementary Data 3 [file 41467_2023_38296_MOESM5_ESM.zip › TP-BUD.pdf]

## TP-CAK

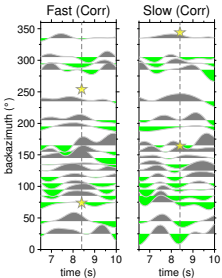

Supplement: Supplementary file 5 — Supplementary Data 3 [file 41467_2023_38296_MOESM5_ESM.zip › TP-CAK.pdf]

## TP-CHG

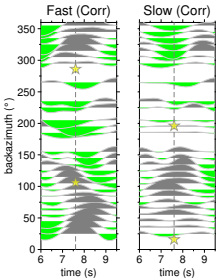

Supplement: Supplementary file 5 — Supplementary Data 3 [file 41467_2023_38296_MOESM5_ESM.zip › TP-CHG.pdf]

## TP-CIS

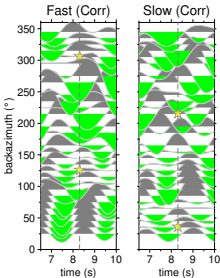

Supplement: Supplementary file 5 — Supplementary Data 3 [file 41467_2023_38296_MOESM5_ESM.zip › TP-CIS.pdf]

## TP-CIW

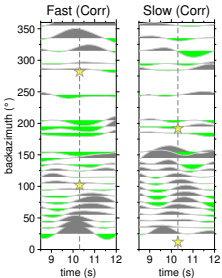

Supplement: Supplementary file 5 — Supplementary Data 3 [file 41467_2023_38296_MOESM5_ESM.zip › TP-CIW.pdf]

## TP-CUM

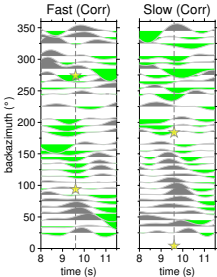

Supplement: Supplementary file 5 — Supplementary Data 3 [file 41467_2023_38296_MOESM5_ESM.zip › TP-CUM.pdf]

## TP-CUQ

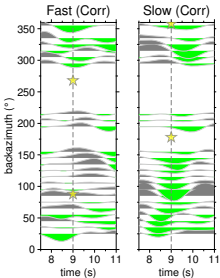

Supplement: Supplementary file 5 — Supplementary Data 3 [file 41467_2023_38296_MOESM5_ESM.zip › TP-CUQ.pdf]

## TP-DAX

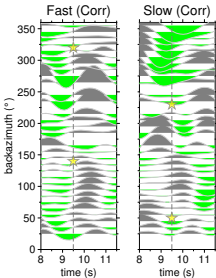

Supplement: Supplementary file 5 — Supplementary Data 3 [file 41467_2023_38296_MOESM5_ESM.zip › TP-DAX.pdf]

## TP-DOC

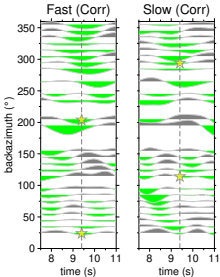

Supplement: Supplementary file 5 — Supplementary Data 3 [file 41467_2023_38296_MOESM5_ESM.zip › TP-DOC.pdf]

## TP-GEJ

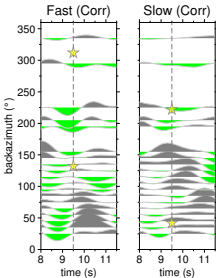

Supplement: Supplementary file 5 — Supplementary Data 3 [file 41467_2023_38296_MOESM5_ESM.zip › TP-GEJ.pdf]

## TP-GOZ

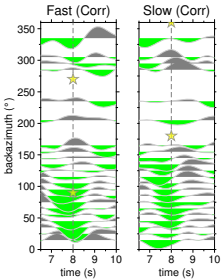

Supplement: Supplementary file 5 — Supplementary Data 3 [file 41467_2023_38296_MOESM5_ESM.zip › TP-GOZ.pdf]

## TP-HUE

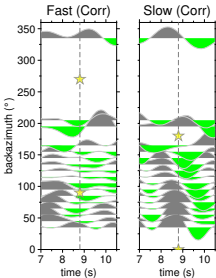

Supplement: Supplementary file 5 — Supplementary Data 3 [file 41467_2023_38296_MOESM5_ESM.zip › TP-HUE.pdf]

## TP-JIG

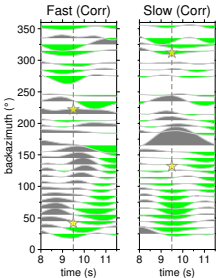

Supplement: Supplementary file 5 — Supplementary Data 3 [file 41467_2023_38296_MOESM5_ESM.zip › TP-JIG.pdf]

## TP-JIL

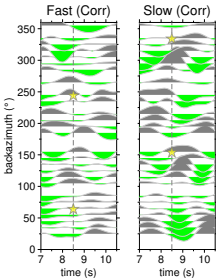

Supplement: Supplementary file 5 — Supplementary Data 3 [file 41467_2023_38296_MOESM5_ESM.zip › TP-JIL.pdf]

## TP-JIM

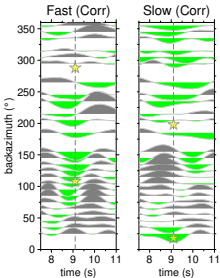

Supplement: Supplementary file 5 — Supplementary Data 3 [file 41467_2023_38296_MOESM5_ESM.zip › TP-JIM.pdf]

## TP-JIR

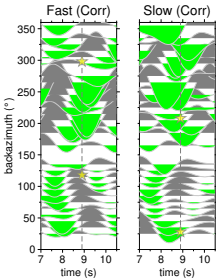

Supplement: Supplementary file 5 — Supplementary Data 3 [file 41467_2023_38296_MOESM5_ESM.zip › TP-JIR.pdf]

## TP-KUS

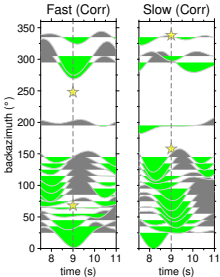

Supplement: Supplementary file 5 — Supplementary Data 3 [file 41467_2023_38296_MOESM5_ESM.zip › TP-KUS.pdf]

## TP-MAL

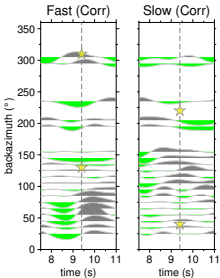

Supplement: Supplementary file 5 — Supplementary Data 3 [file 41467_2023_38296_MOESM5_ESM.zip › TP-MAL.pdf]

## TP-MAY

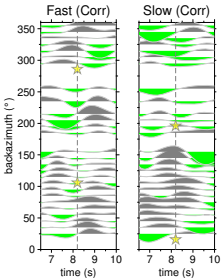

Supplement: Supplementary file 5 — Supplementary Data 3 [file 41467_2023_38296_MOESM5_ESM.zip › TP-MAY.pdf]

## TP-MYM

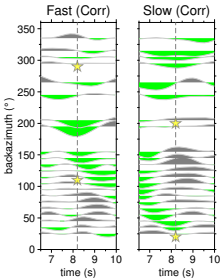

Supplement: Supplementary file 5 — Supplementary Data 3 [file 41467_2023_38296_MOESM5_ESM.zip › TP-MYM.pdf]

## TP-NIG

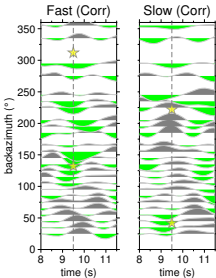

Supplement: Supplementary file 5 — Supplementary Data 3 [file 41467_2023_38296_MOESM5_ESM.zip › TP-NIG.pdf]

## TP-PAJ

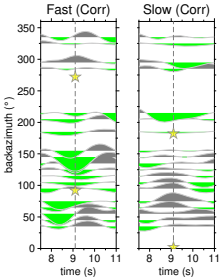

Supplement: Supplementary file 5 — Supplementary Data 3 [file 41467_2023_38296_MOESM5_ESM.zip › TP-PAJ.pdf]

## TP-PAY

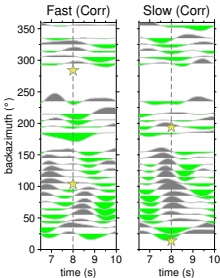

Supplement: Supplementary file 5 — Supplementary Data 3 [file 41467_2023_38296_MOESM5_ESM.zip › TP-PAY.pdf]

## TP-QIR

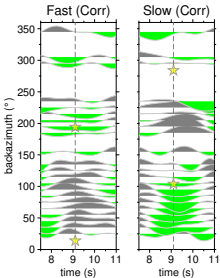

Supplement: Supplementary file 5 — Supplementary Data 3 [file 41467_2023_38296_MOESM5_ESM.zip › TP-QIR.pdf]

## TP-QUS

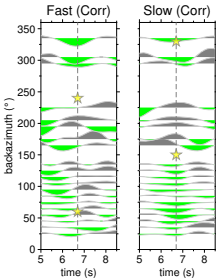

Supplement: Supplementary file 5 — Supplementary Data 3 [file 41467_2023_38296_MOESM5_ESM.zip › TP-QUS.pdf]

## TP-REB

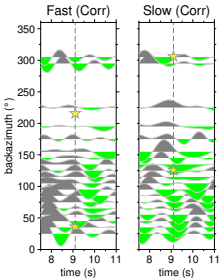

Supplement: Supplementary file 5 — Supplementary Data 3 [file 41467_2023_38296_MOESM5_ESM.zip › TP-REB.pdf]

## TP-RIT

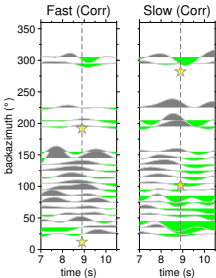

Supplement: Supplementary file 5 — Supplementary Data 3 [file 41467_2023_38296_MOESM5_ESM.zip › TP-RIT.pdf]

## TP-RUJ

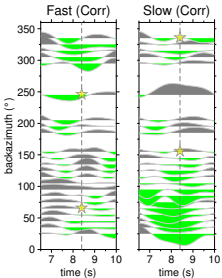

Supplement: Supplementary file 5 — Supplementary Data 3 [file 41467_2023_38296_MOESM5_ESM.zip › TP-RUJ.pdf]

## TP-RUS

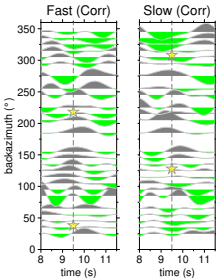

Supplement: Supplementary file 5 — Supplementary Data 3 [file 41467_2023_38296_MOESM5_ESM.zip › TP-RUS.pdf]

## TP-SAG

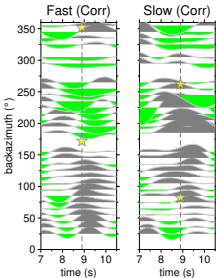

Supplement: Supplementary file 5 — Supplementary Data 3 [file 41467_2023_38296_MOESM5_ESM.zip › TP-SAG.pdf]

## TP-SHZ

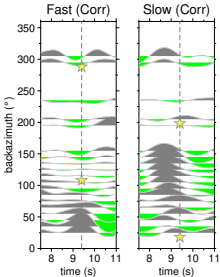

Supplement: Supplementary file 5 — Supplementary Data 3 [file 41467_2023_38296_MOESM5_ESM.zip › TP-SHZ.pdf]

## TP-WEB

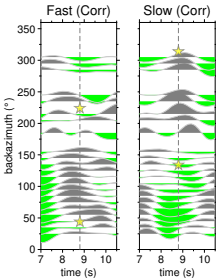

Supplement: Supplementary file 5 — Supplementary Data 3 [file 41467_2023_38296_MOESM5_ESM.zip › TP-WEB.pdf]

## TP-WUM

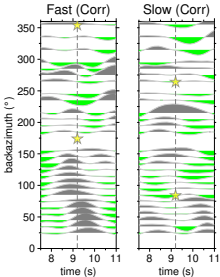

Supplement: Supplementary file 5 — Supplementary Data 3 [file 41467_2023_38296_MOESM5_ESM.zip › TP-WUM.pdf]

## TP-XIB

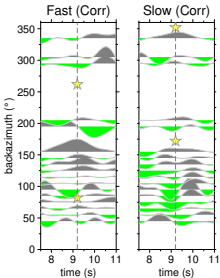

Supplement: Supplementary file 5 — Supplementary Data 3 [file 41467_2023_38296_MOESM5_ESM.zip › TP-XIB.pdf]

## TP-XIS

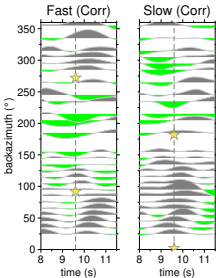

Supplement: Supplementary file 5 — Supplementary Data 3 [file 41467_2023_38296_MOESM5_ESM.zip › TP-XIS.pdf]

## TP-XIZ

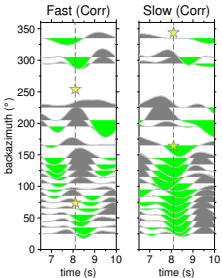

Supplement: Supplementary file 5 — Supplementary Data 3 [file 41467_2023_38296_MOESM5_ESM.zip › TP-XIZ.pdf]

## TP-XZZ

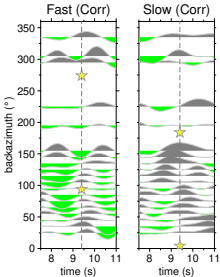

Supplement: Supplementary file 5 — Supplementary Data 3 [file 41467_2023_38296_MOESM5_ESM.zip › TP-XZZ.pdf]

## TP-YAL

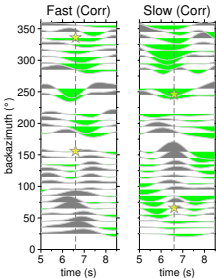

Supplement: Supplementary file 5 — Supplementary Data 3 [file 41467_2023_38296_MOESM5_ESM.zip › TP-YAL.pdf]

## TP-YAR

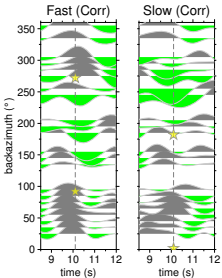

Supplement: Supplementary file 5 — Supplementary Data 3 [file 41467_2023_38296_MOESM5_ESM.zip › TP-YAR.pdf]

## TP-ZUZ

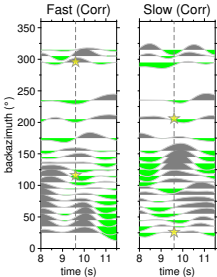

Supplement: Supplementary file 5 — Supplementary Data 3 [file 41467_2023_38296_MOESM5_ESM.zip › TP-ZUZ.pdf]

# XF-H0010

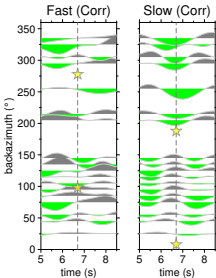

Supplement: Supplementary file 5 — Supplementary Data 3 [file 41467_2023_38296_MOESM5_ESM.zip › XF-H0010.pdf]

# XF-H0020

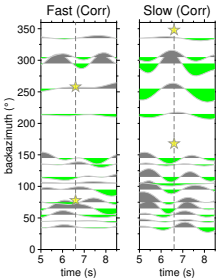

Supplement: Supplementary file 5 — Supplementary Data 3 [file 41467_2023_38296_MOESM5_ESM.zip › XF-H0020.pdf]

# XF-H0070

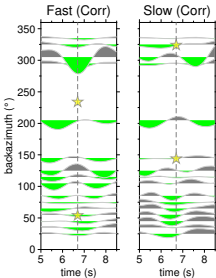

Supplement: Supplementary file 5 — Supplementary Data 3 [file 41467_2023_38296_MOESM5_ESM.zip › XF-H0070.pdf]

## XF-H0100

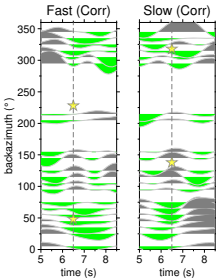

Supplement: Supplementary file 5 — Supplementary Data 3 [file 41467_2023_38296_MOESM5_ESM.zip › XF-H0100.pdf]

## XF-H0120

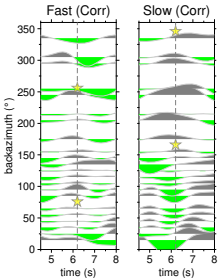

Supplement: Supplementary file 5 — Supplementary Data 3 [file 41467_2023_38296_MOESM5_ESM.zip › XF-H0120.pdf]

# XF-H0130

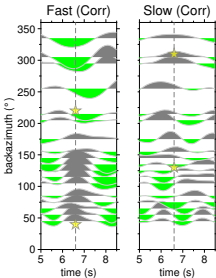

Supplement: Supplementary file 5 — Supplementary Data 3 [file 41467_2023_38296_MOESM5_ESM.zip › XF-H0130.pdf]

# XF-H0150

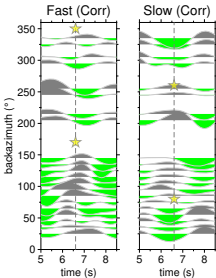

Supplement: Supplementary file 5 — Supplementary Data 3 [file 41467_2023_38296_MOESM5_ESM.zip › XF-H0150.pdf]

# XF-H0170

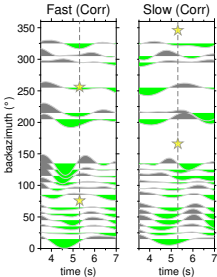

Supplement: Supplementary file 5 — Supplementary Data 3 [file 41467_2023_38296_MOESM5_ESM.zip › XF-H0170.pdf]

# XF-H0180

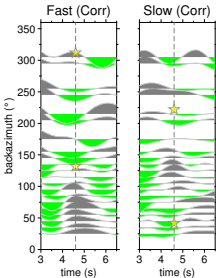

Supplement: Supplementary file 5 — Supplementary Data 3 [file 41467_2023_38296_MOESM5_ESM.zip › XF-H0180.pdf]

## XF-H0190

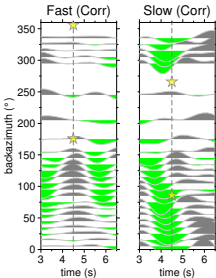

Supplement: Supplementary file 5 — Supplementary Data 3 [file 41467_2023_38296_MOESM5_ESM.zip › XF-H0190.pdf]

## XF-H0210

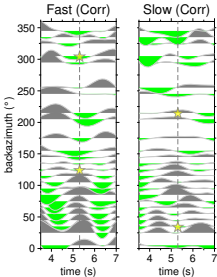

Supplement: Supplementary file 5 — Supplementary Data 3 [file 41467_2023_38296_MOESM5_ESM.zip › XF-H0210.pdf]

## XF-H0220

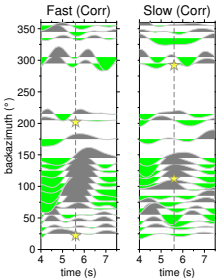

Supplement: Supplementary file 5 — Supplementary Data 3 [file 41467_2023_38296_MOESM5_ESM.zip › XF-H0220.pdf]

## XF-H0230

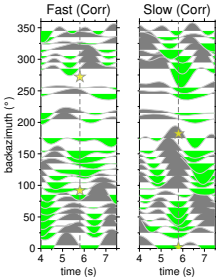

Supplement: Supplementary file 5 — Supplementary Data 3 [file 41467_2023_38296_MOESM5_ESM.zip › XF-H0230.pdf]

## XF-H0240

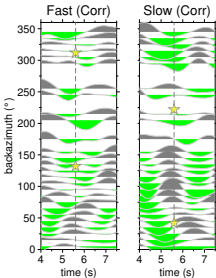

Supplement: Supplementary file 5 — Supplementary Data 3 [file 41467_2023_38296_MOESM5_ESM.zip › XF-H0240.pdf]

## XF-H0260

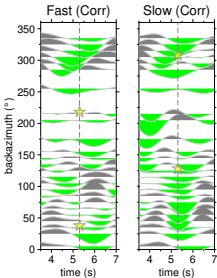

Supplement: Supplementary file 5 — Supplementary Data 3 [file 41467_2023_38296_MOESM5_ESM.zip › XF-H0260.pdf]

## XF-H0270

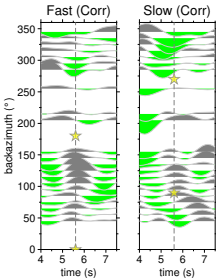

Supplement: Supplementary file 5 — Supplementary Data 3 [file 41467_2023_38296_MOESM5_ESM.zip › XF-H0270.pdf]

# XF-H0290

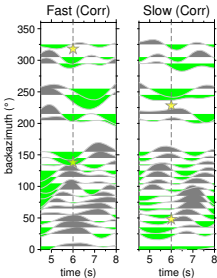

Supplement: Supplementary file 5 — Supplementary Data 3 [file 41467_2023_38296_MOESM5_ESM.zip › XF-H0290.pdf]

# XF-H0310

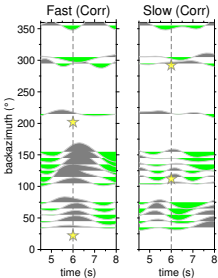

Supplement: Supplementary file 5 — Supplementary Data 3 [file 41467_2023_38296_MOESM5_ESM.zip › XF-H0310.pdf]

## XF-H0330

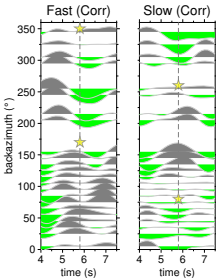

Supplement: Supplementary file 5 — Supplementary Data 3 [file 41467_2023_38296_MOESM5_ESM.zip › XF-H0330.pdf]

## XF-H0340

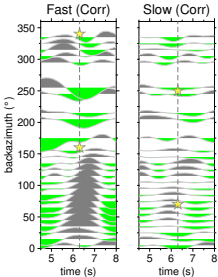

Supplement: Supplementary file 5 — Supplementary Data 3 [file 41467_2023_38296_MOESM5_ESM.zip › XF-H0340.pdf]

## XF-H0360

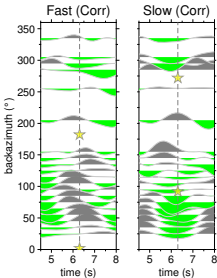

Supplement: Supplementary file 5 — Supplementary Data 3 [file 41467_2023_38296_MOESM5_ESM.zip › XF-H0360.pdf]

## XF-H0380

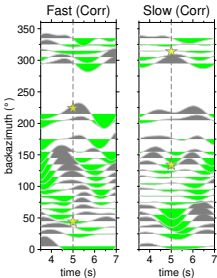

Supplement: Supplementary file 5 — Supplementary Data 3 [file 41467_2023_38296_MOESM5_ESM.zip › XF-H0380.pdf]

# XF-H0410

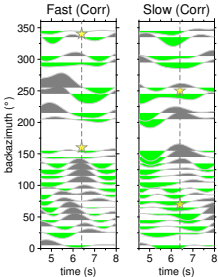

Supplement: Supplementary file 5 — Supplementary Data 3 [file 41467_2023_38296_MOESM5_ESM.zip › XF-H0410.pdf]

## XF-H0440

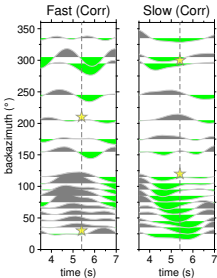

Supplement: Supplementary file 5 — Supplementary Data 3 [file 41467_2023_38296_MOESM5_ESM.zip › XF-H0440.pdf]

## XF-H0480

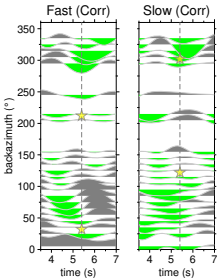

Supplement: Supplementary file 5 — Supplementary Data 3 [file 41467_2023_38296_MOESM5_ESM.zip › XF-H0480.pdf]

## XF-H0500

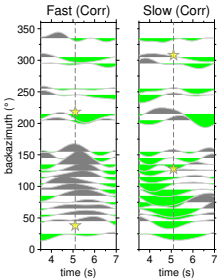

Supplement: Supplementary file 5 — Supplementary Data 3 [file 41467_2023_38296_MOESM5_ESM.zip › XF-H0500.pdf]

## XF-H0510

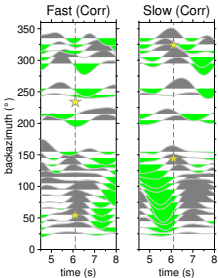

Supplement: Supplementary file 5 — Supplementary Data 3 [file 41467_2023_38296_MOESM5_ESM.zip › XF-H0510.pdf]

# XF-H0520

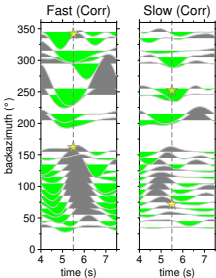

Supplement: Supplementary file 5 — Supplementary Data 3 [file 41467_2023_38296_MOESM5_ESM.zip › XF-H0520.pdf]

# XF-H0530

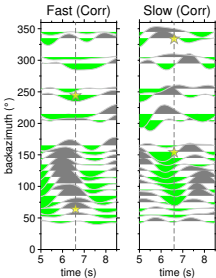

Supplement: Supplementary file 5 — Supplementary Data 3 [file 41467_2023_38296_MOESM5_ESM.zip › XF-H0530.pdf]

## XF-H0540

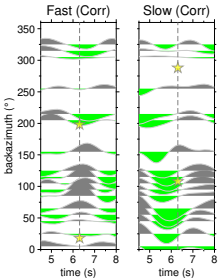

Supplement: Supplementary file 5 — Supplementary Data 3 [file 41467_2023_38296_MOESM5_ESM.zip › XF-H0540.pdf]

# XF-H0550

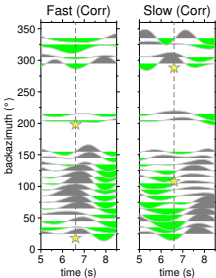

Supplement: Supplementary file 5 — Supplementary Data 3 [file 41467_2023_38296_MOESM5_ESM.zip › XF-H0550.pdf]

## XF-H0560

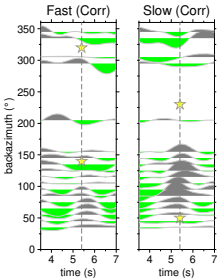

Supplement: Supplementary file 5 — Supplementary Data 3 [file 41467_2023_38296_MOESM5_ESM.zip › XF-H0560.pdf]

## XF-H0570

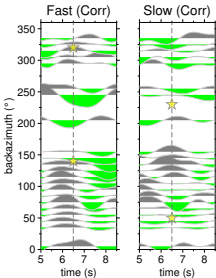

Supplement: Supplementary file 5 — Supplementary Data 3 [file 41467_2023_38296_MOESM5_ESM.zip › XF-H0570.pdf]

## XF-H0600

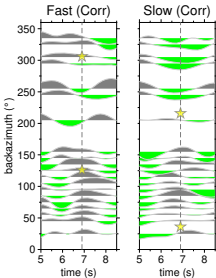

Supplement: Supplementary file 5 — Supplementary Data 3 [file 41467_2023_38296_MOESM5_ESM.zip › XF-H0600.pdf]

## XF-H0620

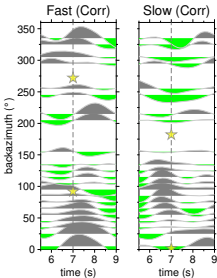

Supplement: Supplementary file 5 — Supplementary Data 3 [file 41467_2023_38296_MOESM5_ESM.zip › XF-H0620.pdf]

## XF-H0630

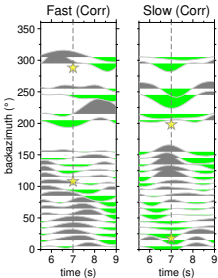

Supplement: Supplementary file 5 — Supplementary Data 3 [file 41467_2023_38296_MOESM5_ESM.zip › XF-H0630.pdf]

# XF-H0641

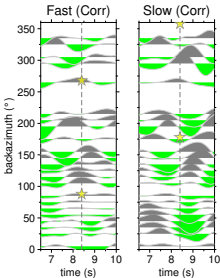

Supplement: Supplementary file 5 — Supplementary Data 3 [file 41467_2023_38296_MOESM5_ESM.zip › XF-H0641.pdf]

# XF-H0660

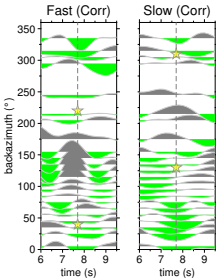

Supplement: Supplementary file 5 — Supplementary Data 3 [file 41467_2023_38296_MOESM5_ESM.zip › XF-H0660.pdf]

## XF-H0670

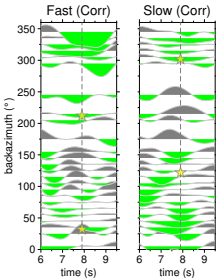

Supplement: Supplementary file 5 — Supplementary Data 3 [file 41467_2023_38296_MOESM5_ESM.zip › XF-H0670.pdf]

## XF-H0680

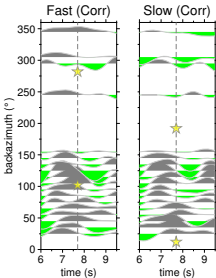

Supplement: Supplementary file 5 — Supplementary Data 3 [file 41467_2023_38296_MOESM5_ESM.zip › XF-H0680.pdf]

# XF-H0710

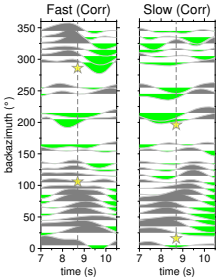

Supplement: Supplementary file 5 — Supplementary Data 3 [file 41467_2023_38296_MOESM5_ESM.zip › XF-H0710.pdf]

# XF-H0720

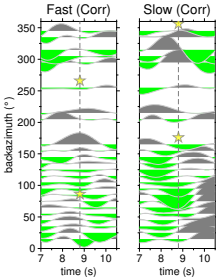

Supplement: Supplementary file 5 — Supplementary Data 3 [file 41467_2023_38296_MOESM5_ESM.zip › XF-H0720.pdf]

# XF-H0730

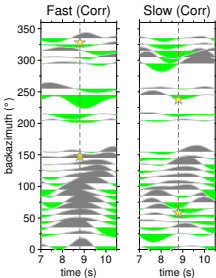

Supplement: Supplementary file 5 — Supplementary Data 3 [file 41467_2023_38296_MOESM5_ESM.zip › XF-H0730.pdf]

# XF-H0740

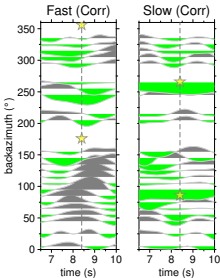

Supplement: Supplementary file 5 — Supplementary Data 3 [file 41467_2023_38296_MOESM5_ESM.zip › XF-H0740.pdf]

# XF-H0760

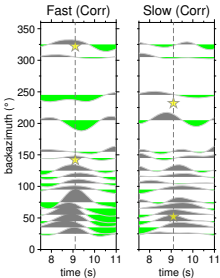

Supplement: Supplementary file 5 — Supplementary Data 3 [file 41467_2023_38296_MOESM5_ESM.zip › XF-H0760.pdf]

## XF-H0770

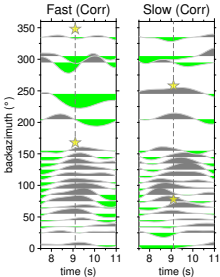

Supplement: Supplementary file 5 — Supplementary Data 3 [file 41467_2023_38296_MOESM5_ESM.zip › XF-H0770.pdf]

## XF-H0780

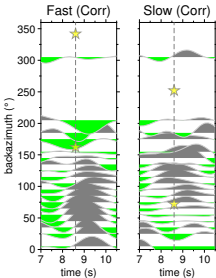

Supplement: Supplementary file 5 — Supplementary Data 3 [file 41467_2023_38296_MOESM5_ESM.zip › XF-H0780.pdf]

## XF-H0790

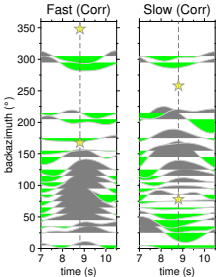

Supplement: Supplementary file 5 — Supplementary Data 3 [file 41467_2023_38296_MOESM5_ESM.zip › XF-H0790.pdf]

# XF-H0800

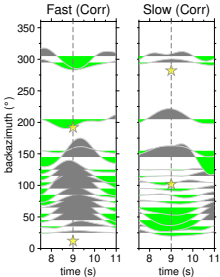

Supplement: Supplementary file 5 — Supplementary Data 3 [file 41467_2023_38296_MOESM5_ESM.zip › XF-H0800.pdf]

# XF-H0810

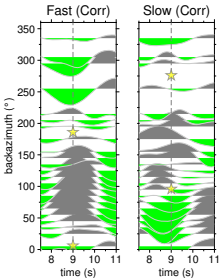

Supplement: Supplementary file 5 — Supplementary Data 3 [file 41467_2023_38296_MOESM5_ESM.zip › XF-H0810.pdf]

# XF-H1010

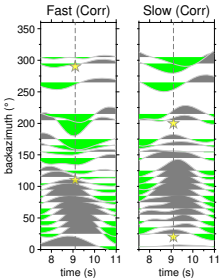

Supplement: Supplementary file 5 — Supplementary Data 3 [file 41467_2023_38296_MOESM5_ESM.zip › XF-H1010.pdf]

## XF-H1030

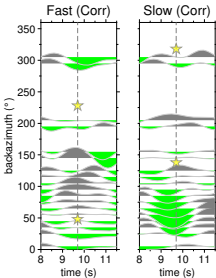

Supplement: Supplementary file 5 — Supplementary Data 3 [file 41467_2023_38296_MOESM5_ESM.zip › XF-H1030.pdf]

## XF-H1040

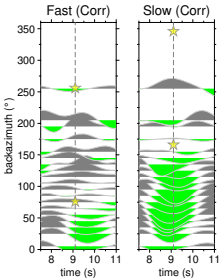

Supplement: Supplementary file 5 — Supplementary Data 3 [file 41467_2023_38296_MOESM5_ESM.zip › XF-H1040.pdf]

# XF-H1080

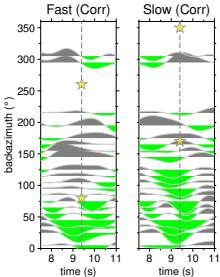

Supplement: Supplementary file 5 — Supplementary Data 3 [file 41467_2023_38296_MOESM5_ESM.zip › XF-H1080.pdf]

# XF-H1130

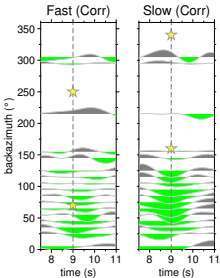

Supplement: Supplementary file 5 — Supplementary Data 3 [file 41467_2023_38296_MOESM5_ESM.zip › XF-H1130.pdf]

# XF-H1140

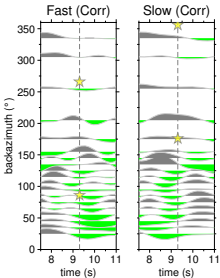

Supplement: Supplementary file 5 — Supplementary Data 3 [file 41467_2023_38296_MOESM5_ESM.zip › XF-H1140.pdf]

## XF-H1160

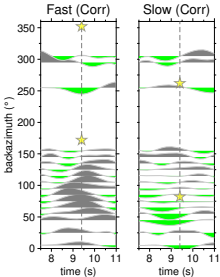

Supplement: Supplementary file 5 — Supplementary Data 3 [file 41467_2023_38296_MOESM5_ESM.zip › XF-H1160.pdf]

## XF-H1190

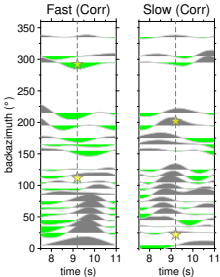

Supplement: Supplementary file 5 — Supplementary Data 3 [file 41467_2023_38296_MOESM5_ESM.zip › XF-H1190.pdf]
