# Supplementary material for: Southern Tibetan rifting since late Miocene enabled by basal shear of the underthrusting Indian lithosphere
Source: Nat Commun. 2023 May 4;14:2565. doi: 10.1038/s41467-023-38296-w (PMC10160080; doi:10.1038/s41467-023-38296-w)
Supplement: Supplementary file 6 — Supplementary Data 4 [file 41467_2023_38296_MOESM6_ESM.zip › normal_TP-ZUZ.pdf]

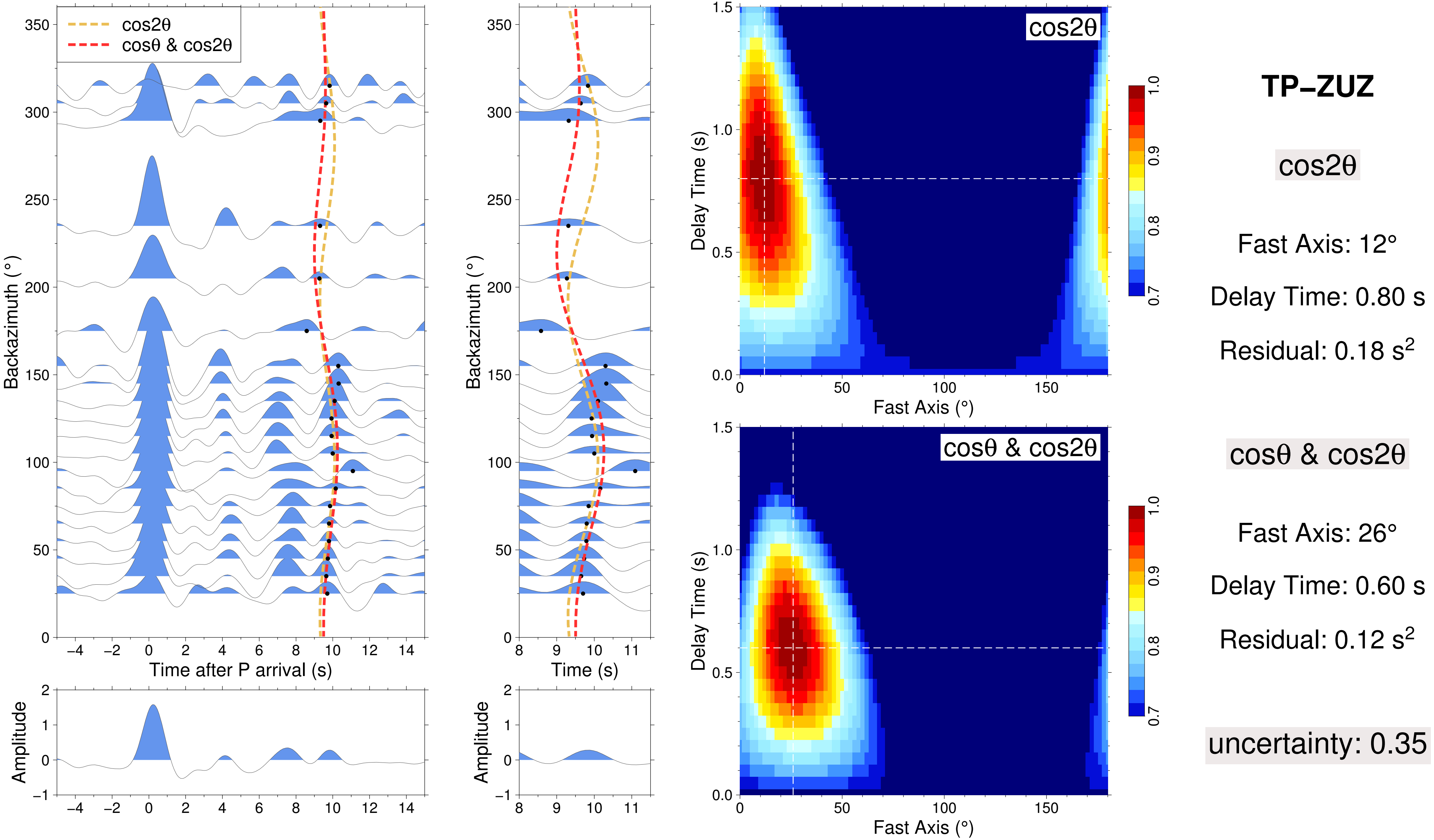
cos2θ
cosθ & cos2θ
Backazimuth (°)
Time after P arrival (s)
Amplitude
Time (s)
Delay Time (s)
Fast Axis (°)
TP−ZUZ
cos2θ
Fast Axis: 12°
Delay Time: 0.80 s
Residual: 0.18 s^2
cosθ & cos2θ
Fast Axis: 26°
Delay Time: 0.60 s
Residual: 0.12 s^2
uncertainty: 0.35
