# Supplementary material for: Southern Tibetan rifting since late Miocene enabled by basal shear of the underthrusting Indian lithosphere
Source: Nat Commun. 2023 May 4;14:2565. doi: 10.1038/s41467-023-38296-w (PMC10160080; doi:10.1038/s41467-023-38296-w)
Supplement: Supplementary file 6 — Supplementary Data 4 [file 41467_2023_38296_MOESM6_ESM.zip › normal_XF-H0800.pdf]

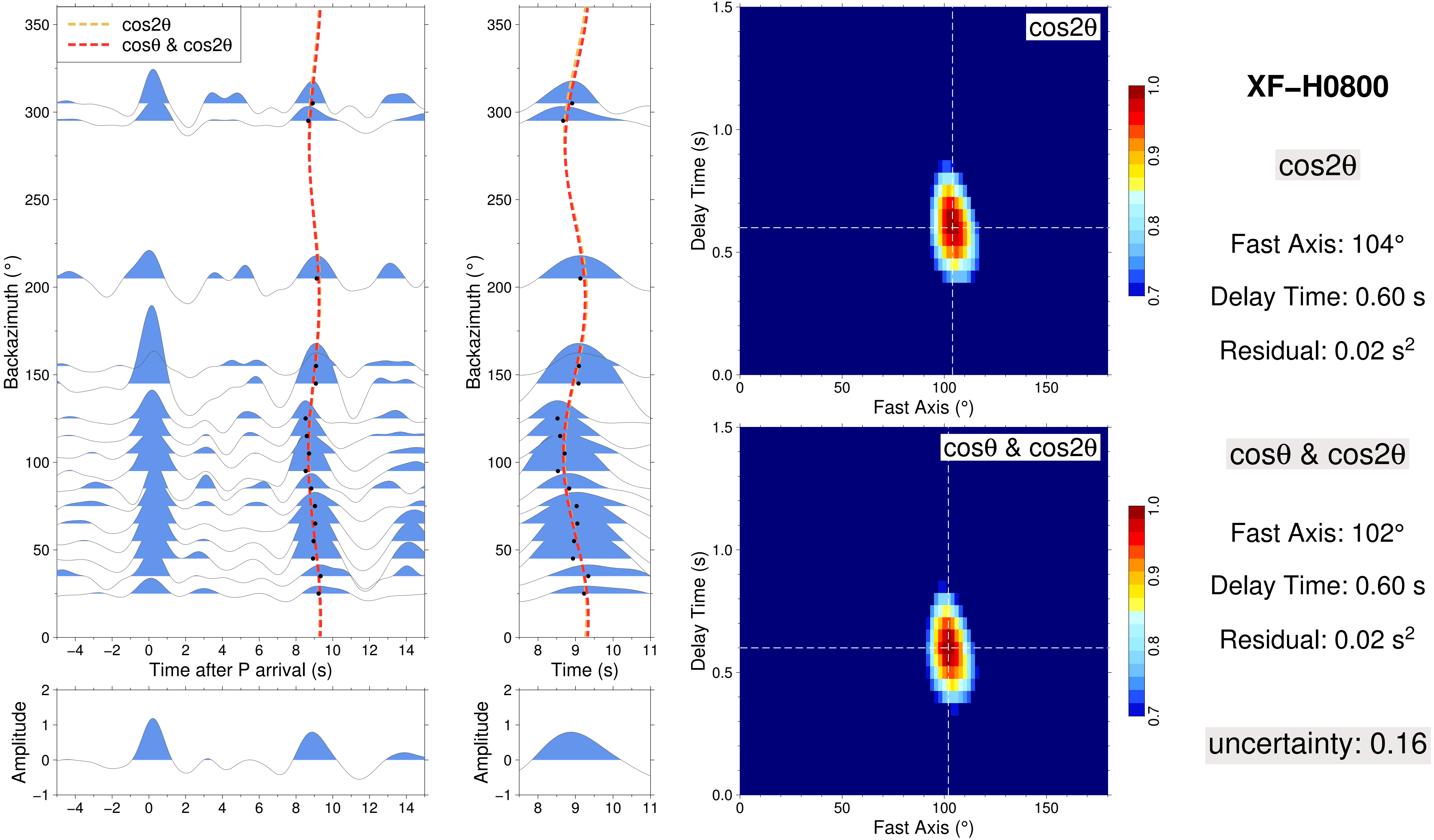
cos2θ
cosθ & cos2θ
Backazimuth (°)
Time after P arrival (s)
Amplitude
Time (s)
Delay Time (s)
Fast Axis (°)
XF–H0800
cos2θ
Fast Axis: 104°
Delay Time: 0.60 s
Residual: 0.02 s²
cosθ & cos2θ
Fast Axis: 102°
Delay Time: 0.60 s
Residual: 0.02 s²
uncertainty: 0.16
