# Supplementary material for: Southern Tibetan rifting since late Miocene enabled by basal shear of the underthrusting Indian lithosphere
Source: Nat Commun. 2023 May 4;14:2565. doi: 10.1038/s41467-023-38296-w (PMC10160080; doi:10.1038/s41467-023-38296-w)
Supplement: Supplementary file 8 — Supplementary Data 6 [file 41467_2023_38296_MOESM8_ESM.zip › event 2019.286.20.56.cum.0.2−3.fb1.pdf]

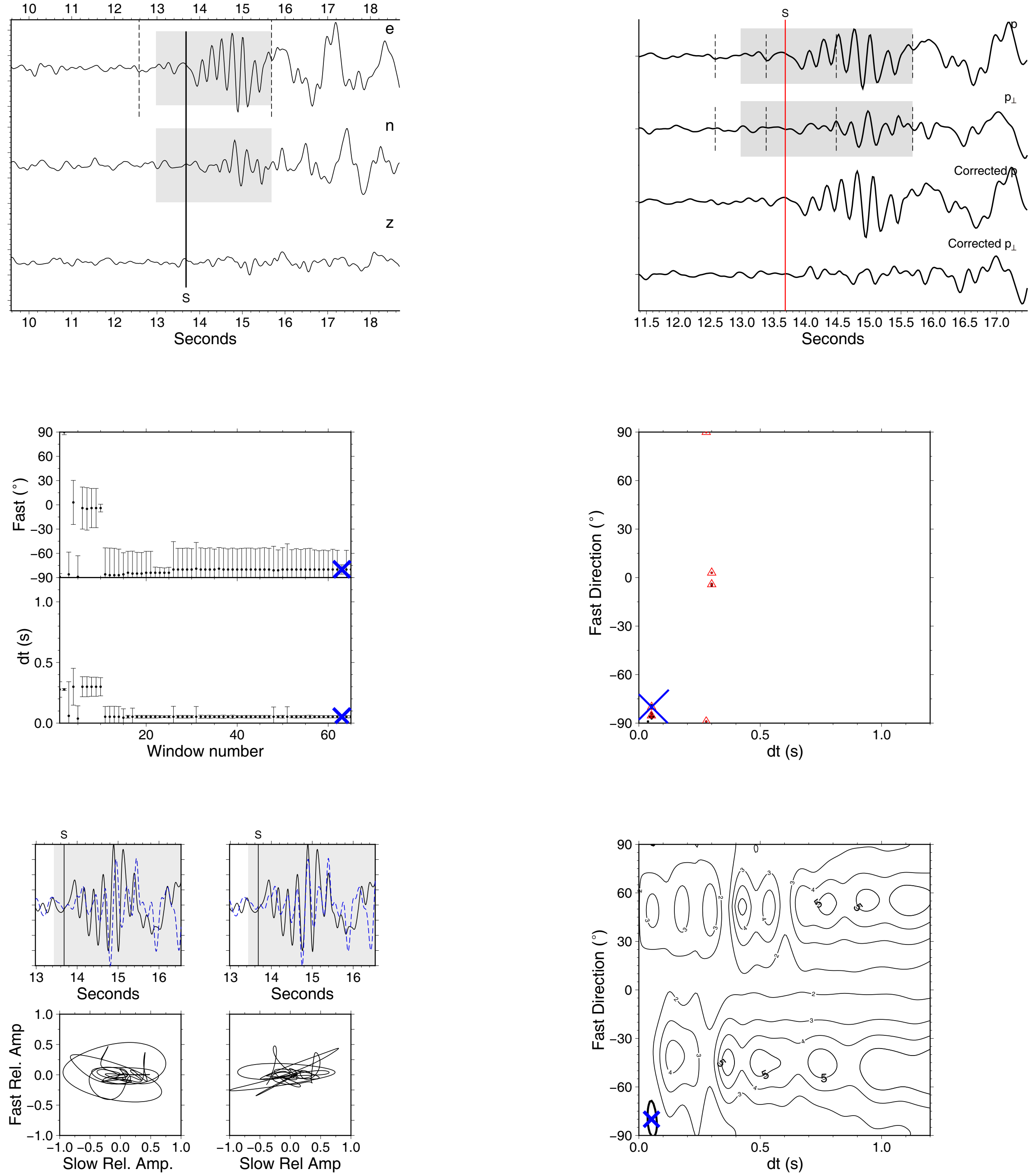

event 2019.286.20.56.cum.0.2−3.fb1

depth: 15 km
distance: 79.9995 km

splitting windows (relative to S−Pick at 13.68 s):
wbeg: −1.10 − −0.30 (5)
wend: 0.80 − 2.00 (16)
selected: 12.981 − 15.682, length: 2.701 s

results: GRADE ACl

fast: 100.0 +/− 5.8 (°)

dt: 0.052 +/− 0.007 (s)

spol: 79.3 +/− 4.8 (°)
