# Supplementary material for: Southern Tibetan rifting since late Miocene enabled by basal shear of the underthrusting Indian lithosphere
Source: Nat Commun. 2023 May 4;14:2565. doi: 10.1038/s41467-023-38296-w (PMC10160080; doi:10.1038/s41467-023-38296-w)
Supplement: Supplementary file 8 — Supplementary Data 6 [file 41467_2023_38296_MOESM8_ESM.zip › event 2020.83.09.59.doc.0.2−3.fb1.pdf]

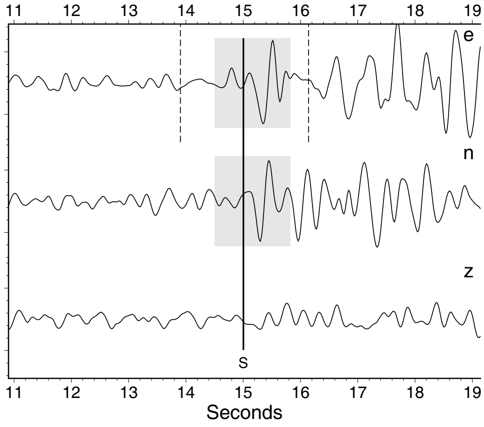

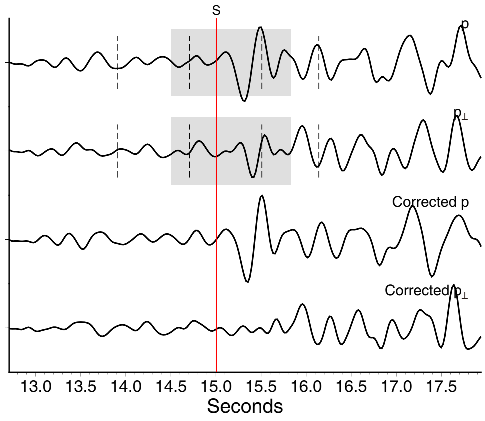

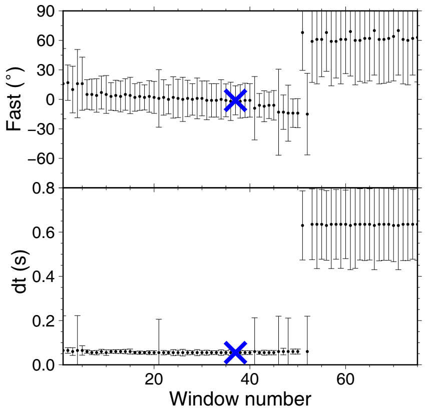

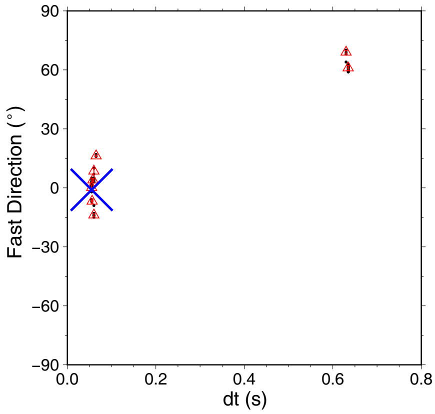

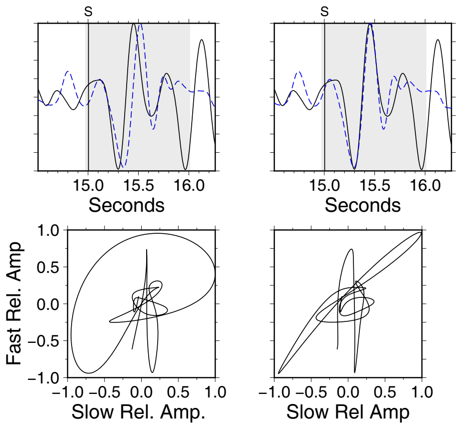

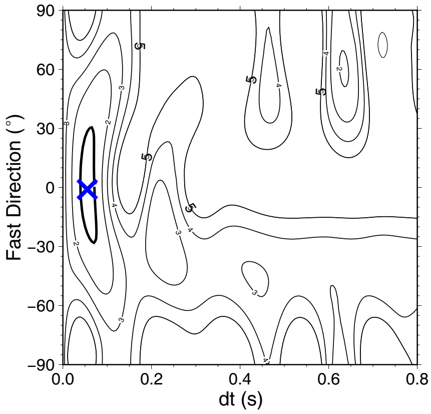

event 2020.83.09.59.doc.0.2−3.fb1

depth: 13 km
distance: 95.4549 km

splitting windows (relative to S−Pick at 15.00 s):
wbeg: −1.10 − −0.30 (5)
wend: 0.51 − 1.14 (15)
selected: 14.504 − 15.826, length: 1.322 s

results: GRADE ACI

fast: 179.0 +/− 14.8 (°)

dt: 0.055 +/− 0.009 (s)

spol: 47.5 +/− 3.0 (°)
