# Supplementary material for: Southern Tibetan rifting since late Miocene enabled by basal shear of the underthrusting Indian lithosphere
Source: Nat Commun. 2023 May 4;14:2565. doi: 10.1038/s41467-023-38296-w (PMC10160080; doi:10.1038/s41467-023-38296-w)
Supplement: Supplementary file 8 — Supplementary Data 6 [file 41467_2023_38296_MOESM8_ESM.zip › event 2021.112.03.08.cum.0.2−3.fb1.pdf]

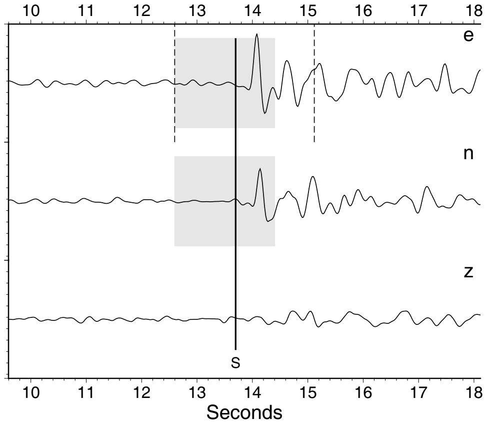

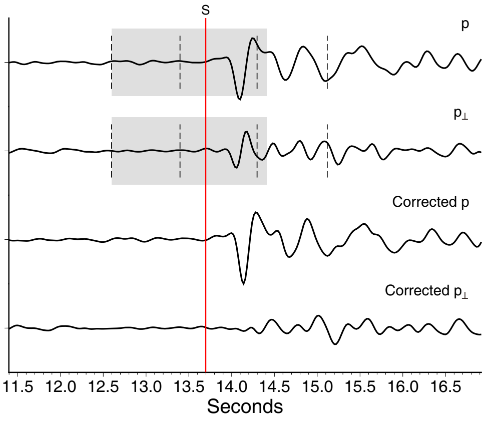

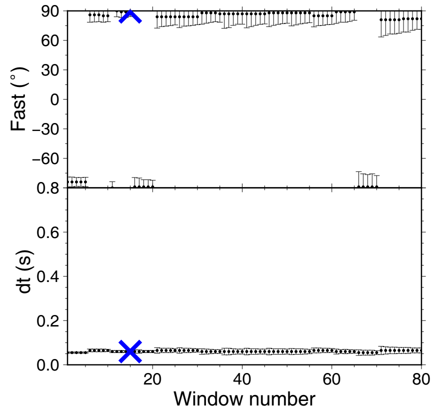

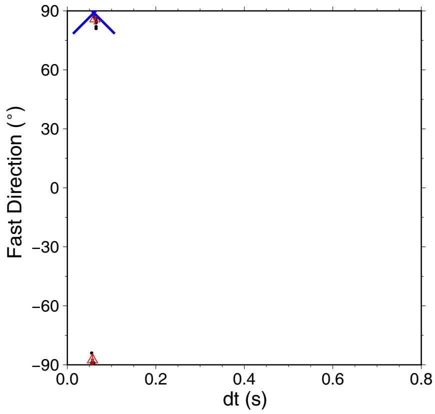

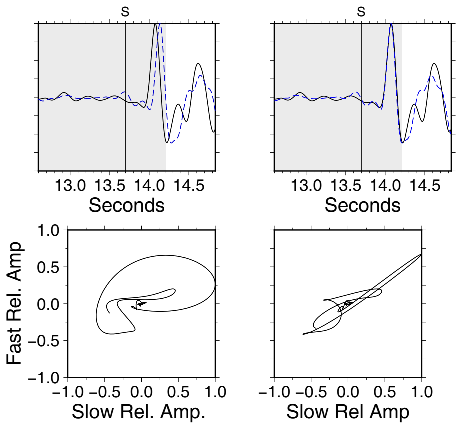

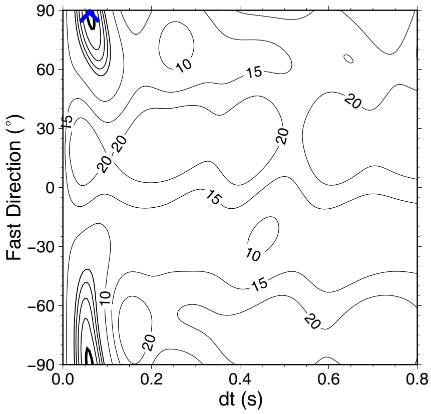

event 2021.112.03.08.cum.0.2–3.fb1

depth: 24 km
distance: 66.5008 km

splitting windows (relative to S–Pick at 13.70 s):
wbeg: −1.10 – −0.30 (5)
wend: 0.60 – 1.42 (16)
selected: 12.597 – 14.407, length: 1.81 s

results: GRADE ACI

fast: 89.0 +/− 4.8 (°)

dt: 0.060 +/− 0.004 (s)

spol: 54.7 +/− 0.8 (°)
