# Supplementary material for: Southern Tibetan rifting since late Miocene enabled by basal shear of the underthrusting Indian lithosphere
Source: Nat Commun. 2023 May 4;14:2565. doi: 10.1038/s41467-023-38296-w (PMC10160080; doi:10.1038/s41467-023-38296-w)
Supplement: Supplementary file 8 — Supplementary Data 6 [file 41467_2023_38296_MOESM8_ESM.zip › event 2021.113.13.40.cum.0.2−3.fb1.pdf]

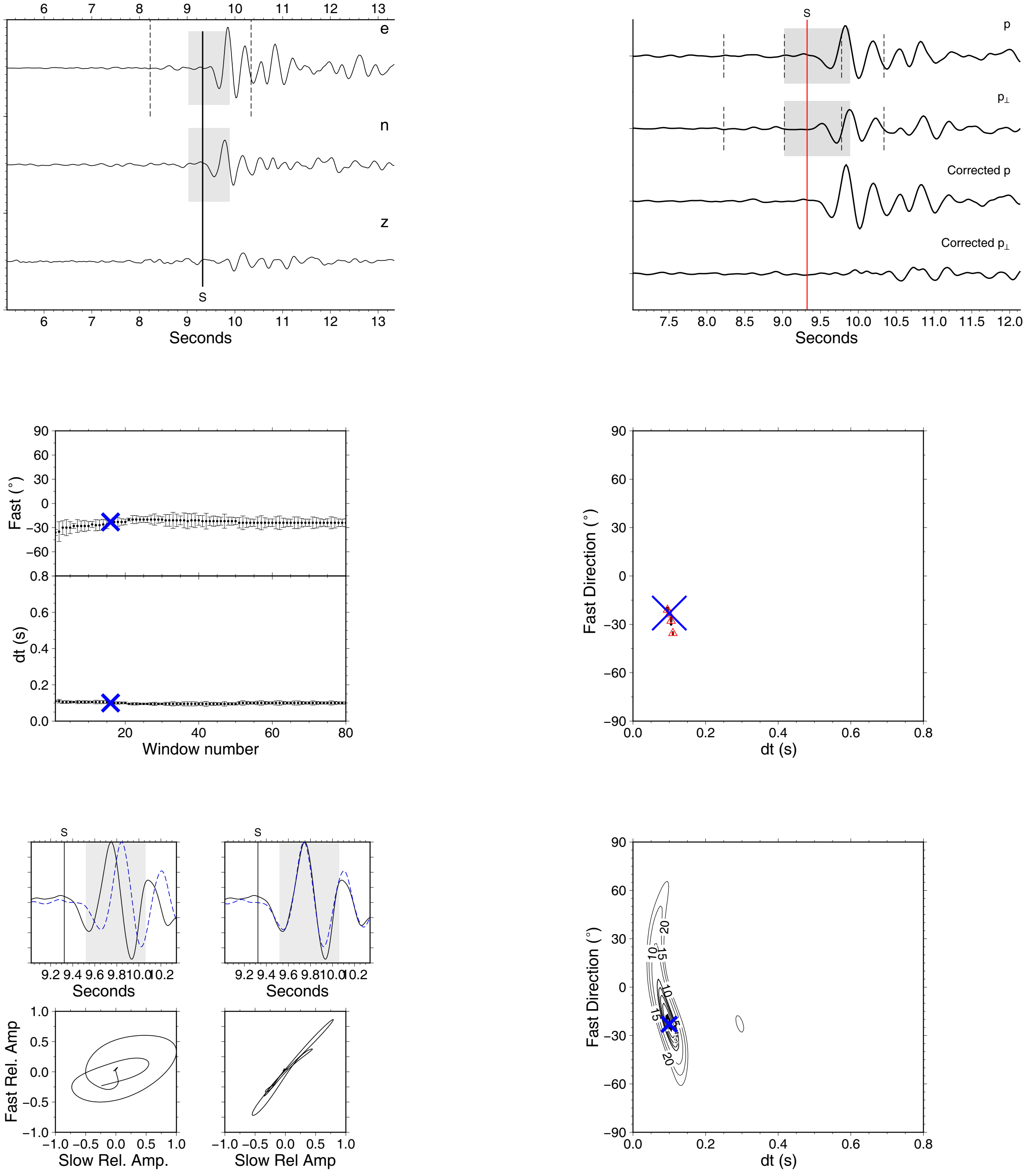

event 2021.113.13.40.cum.0.2–3.fb1

depth: 25 km
distance: 34.9429 km

splitting windows (relative to S–Pick at 9.32 s):
wbeg: –1.10 – –0.30 (5)
wend: 0.46 – 1.02 (16)
selected: 9.0233 – 9.8916, length: 0.8683 s

results: GRADE ACl

fast: 157.0 +/– 2.2 (°)

dt: 0.100 +/– 0.003 (s)

spol: 42.2 +/– 0.2 (°)
