# Supplementary material for: Southern Tibetan rifting since late Miocene enabled by basal shear of the underthrusting Indian lithosphere
Source: Nat Commun. 2023 May 4;14:2565. doi: 10.1038/s41467-023-38296-w (PMC10160080; doi:10.1038/s41467-023-38296-w)
Supplement: Supplementary file 8 — Supplementary Data 6 [file 41467_2023_38296_MOESM8_ESM.zip › event 2021.114.22.25.may.0.2−3.fb1.pdf]

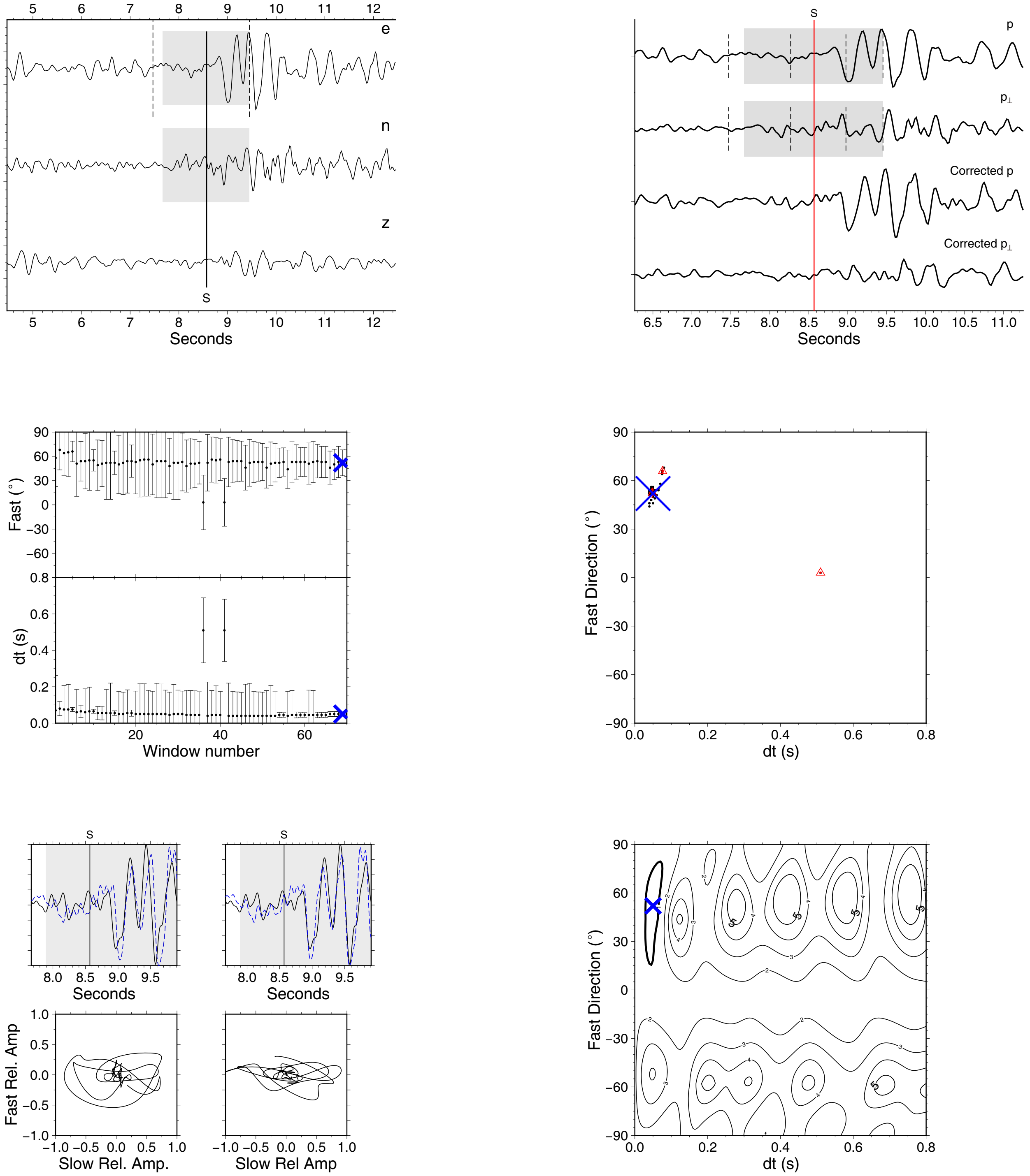

event 2021.114.22.25.may.0.2–3.fb1

depth: 24 km
distance: 39.452 km

splitting windows (relative to S–Pick at 8.57 s):
wbeg: −1.10 – −0.30 (5)
wend: 0.41 – 0.88 (15)
selected: 7.6682 – 9.452, length: 1.7838 s

results: GRADE ACI

fast: 52.0 +/– 16.0 (°)

dt: 0.050 +/– 0.011 (s)

spol: 90.1 +/– 5.5 (°)
