# Supplementary material for: Southern Tibetan rifting since late Miocene enabled by basal shear of the underthrusting Indian lithosphere
Source: Nat Commun. 2023 May 4;14:2565. doi: 10.1038/s41467-023-38296-w (PMC10160080; doi:10.1038/s41467-023-38296-w)
Supplement: Supplementary file 8 — Supplementary Data 6 [file 41467_2023_38296_MOESM8_ESM.zip › event 2021.114.22.25.pay.0.2−3.fb1.pdf]

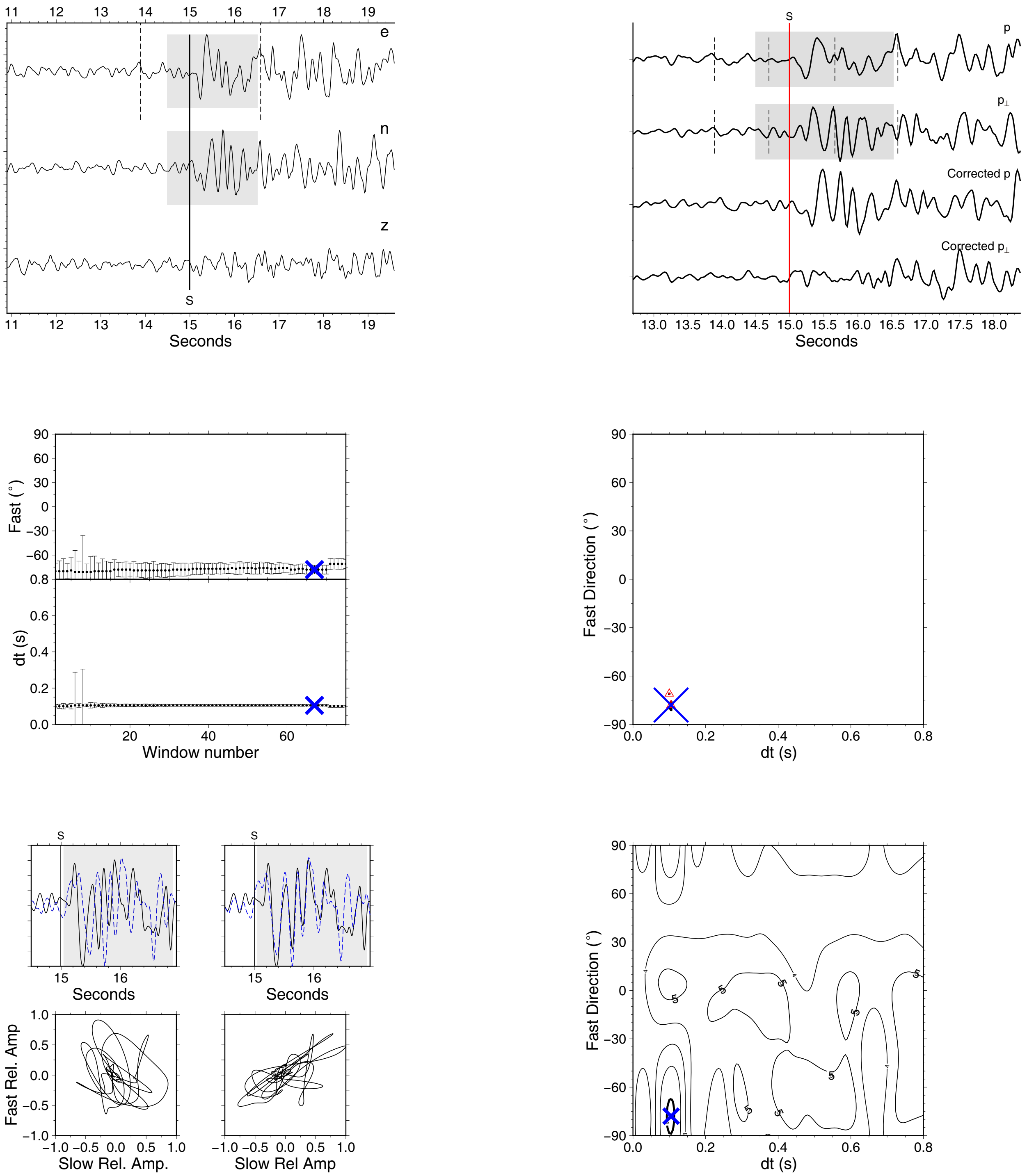

event 2021.114.22.25.pay.0.2-3.fb1

depth: 24 km
distance: 46.7824 km

splitting windows (relative to S−Pick at 14.99 s):
wbeg: −1.10 – −0.30 (5)
wend: 0.67 – 1.60 (15)
selected: 14.493 – 16.522, length: 2.029 s

results: GRADE ACI

fast: 102.0 +/− 5.2 (°)

dt: 0.105 +/− 0.004 (s)

spol: 55.8 +/− 5.4 (°)
