# Supplementary material for: Southern Tibetan rifting since late Miocene enabled by basal shear of the underthrusting Indian lithosphere
Source: Nat Commun. 2023 May 4;14:2565. doi: 10.1038/s41467-023-38296-w (PMC10160080; doi:10.1038/s41467-023-38296-w)
Supplement: Supplementary file 8 — Supplementary Data 6 [file 41467_2023_38296_MOESM8_ESM.zip › event 2021.132.00.07.jir.0.2−3.fb1.pdf]

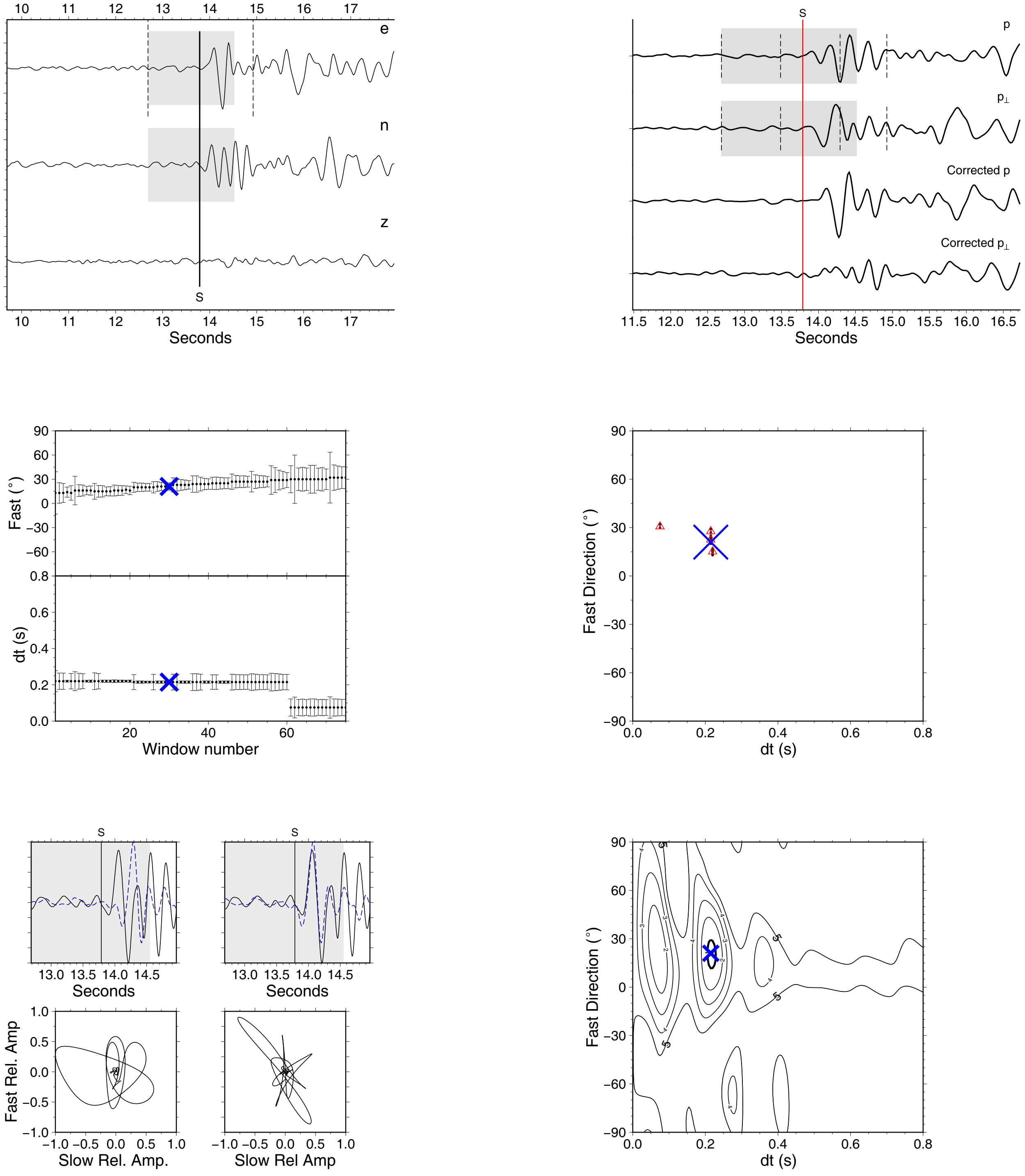

event 2021.132.00.07.jir.0.2–3.fb1

depth: 25 km
distance: 67.3821 km

splitting windows (relative to S–Pick at 13.79 s):
wbeg: –1.10 – –0.30 (5)
wend: 0.51 – 1.14 (15)
selected: 12.687 – 14.519, length: 1.832 s

results: GRADE ACI

fast: 21.0 +/– 4.5 (°)

dt: 0.215 +/– 0.004 (s)

spol: 145.8 +/– 3.6 (°)
