# Supplementary material for: Southern Tibetan rifting since late Miocene enabled by basal shear of the underthrusting Indian lithosphere
Source: Nat Commun. 2023 May 4;14:2565. doi: 10.1038/s41467-023-38296-w (PMC10160080; doi:10.1038/s41467-023-38296-w)
Supplement: Supplementary file 8 — Supplementary Data 6 [file 41467_2023_38296_MOESM8_ESM.zip › event 2021.152.20.02.doc.0.2−3.fb1.pdf]

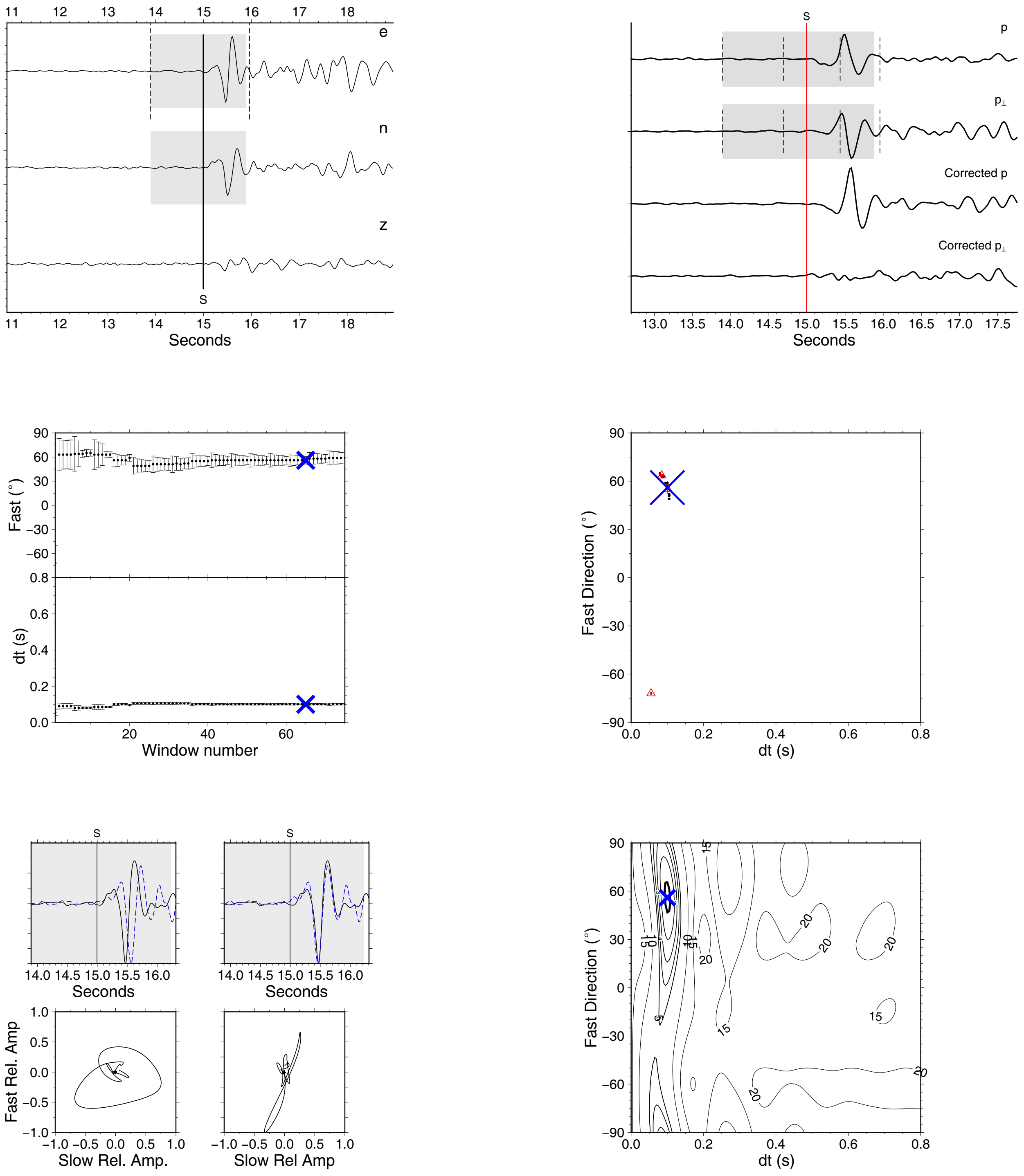

event 2021.152.20.02.doc.0.2−3.fb1

depth: 25 km
distance: 61.9059 km

splitting windows (relative to S−Pick at 14.99 s):
wbeg: −1.10 − −0.30 (5)
wend: 0.44 − 0.96 (15)
selected: 13.895 − 15.882, length: 1.987 s

results: GRADE ACI

fast: 56.0 +/− 4.8 (°)

dt: 0.100 +/− 0.003 (s)

spol: 17.7 +/− 1.3 (°)
