# Supplementary material for: Southern Tibetan rifting since late Miocene enabled by basal shear of the underthrusting Indian lithosphere
Source: Nat Commun. 2023 May 4;14:2565. doi: 10.1038/s41467-023-38296-w (PMC10160080; doi:10.1038/s41467-023-38296-w)
Supplement: Supplementary file 8 — Supplementary Data 6 [file 41467_2023_38296_MOESM8_ESM.zip › event 2021.167.08.19.cis.0.2−3.fb1.pdf]

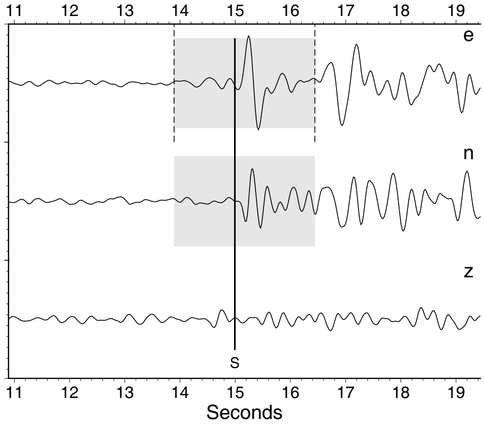

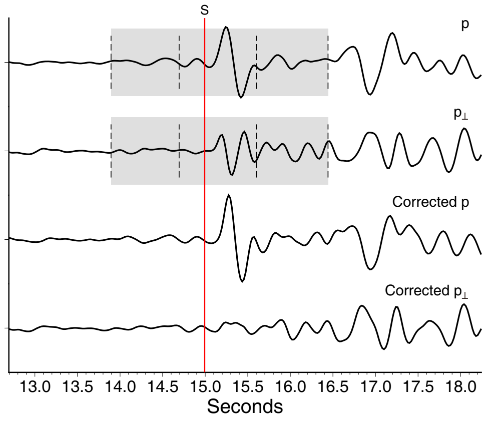

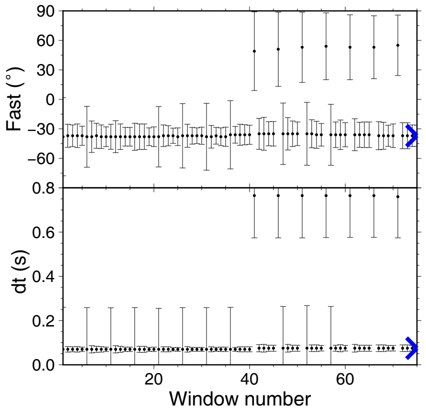

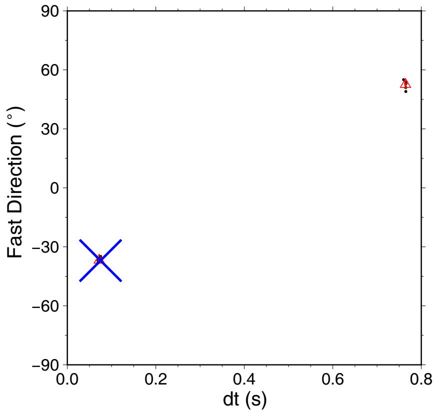

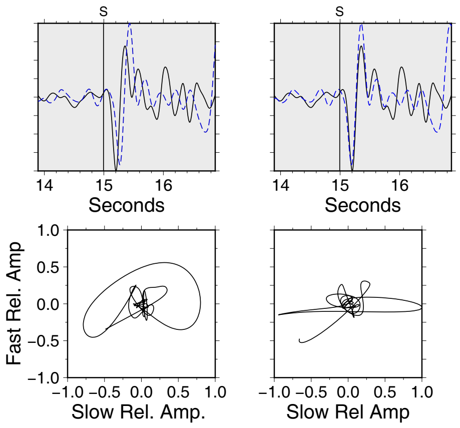

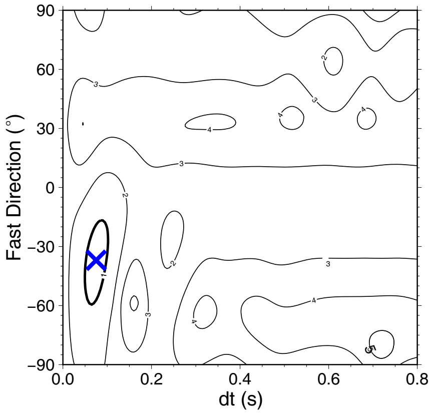

event 2021.167.08.19.cis.0.2−3.fb1

depth: 11 km
distance: 48.9431 km

splitting windows (relative to S−Pick at 14.99 s):
wbeg: −1.10 − −0.30 (5)
wend: 0.61 − 1.45 (15)
selected: 13.894 − 16.443, length: 2.549 s

results: GRADE ACI

fast: 143.0 +/− 10.8 (°)

dt: 0.075 +/− 0.013 (s)

spol: 88.3 +/− 5.8 (°)
