# Supplementary material for: Southern Tibetan rifting since late Miocene enabled by basal shear of the underthrusting Indian lithosphere
Source: Nat Commun. 2023 May 4;14:2565. doi: 10.1038/s41467-023-38296-w (PMC10160080; doi:10.1038/s41467-023-38296-w)
Supplement: Supplementary file 8 — Supplementary Data 6 [file 41467_2023_38296_MOESM8_ESM.zip › event 2021.167.08.19.cuq.0.2−3.fb1.pdf]

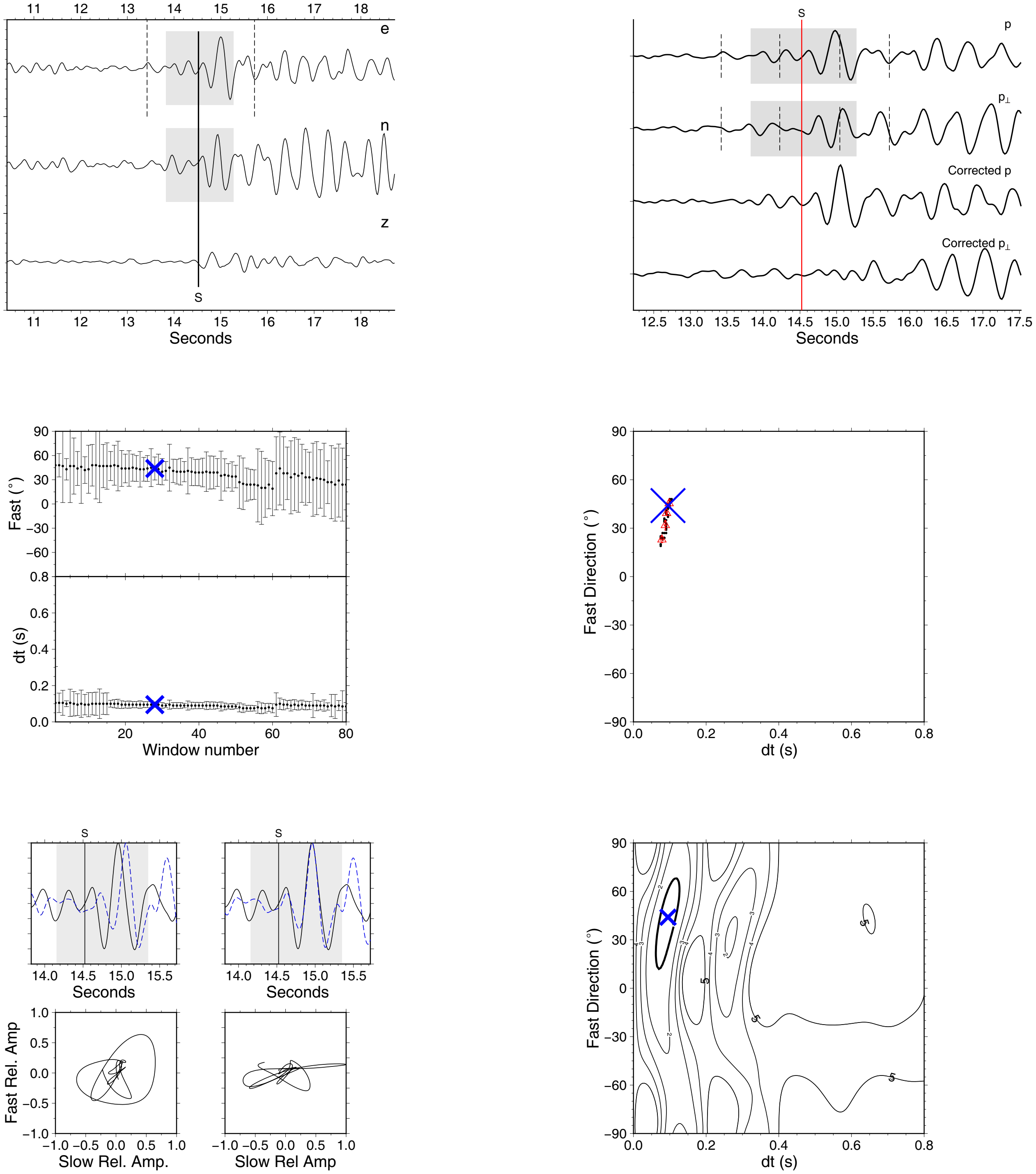

event 2021.167.08.19.cuq.0.2-3.fb1

depth: 11 km
distance: 79.2767 km

splitting windows (relative to S−Pick at 14.52 s):
wbeg: −1.10 − −0.30 (5)
wend: 0.52 − 1.20 (16)
selected: 13.823 − 15.271, length: 1.448 s

results: GRADE ACl

fast: 44.0 +/− 14.2 (°)

dt: 0.095 +/− 0.015 (s)

spol: 76.1 +/− 2.6 (°)
