# Supplementary material for: Southern Tibetan rifting since late Miocene enabled by basal shear of the underthrusting Indian lithosphere
Source: Nat Commun. 2023 May 4;14:2565. doi: 10.1038/s41467-023-38296-w (PMC10160080; doi:10.1038/s41467-023-38296-w)
Supplement: Supplementary file 8 — Supplementary Data 6 [file 41467_2023_38296_MOESM8_ESM.zip › event 2021.168.19.48.rus.0.2−3.fb1.pdf]

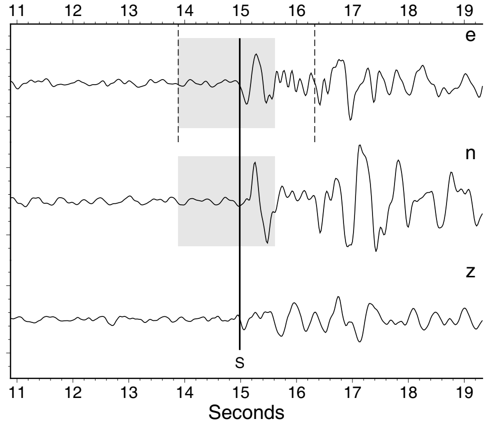

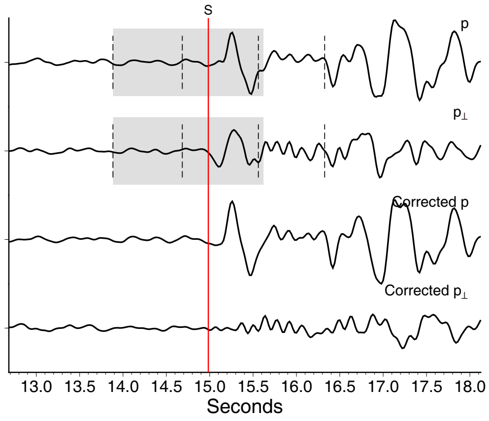

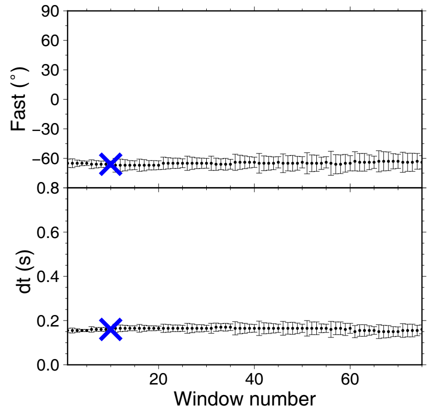

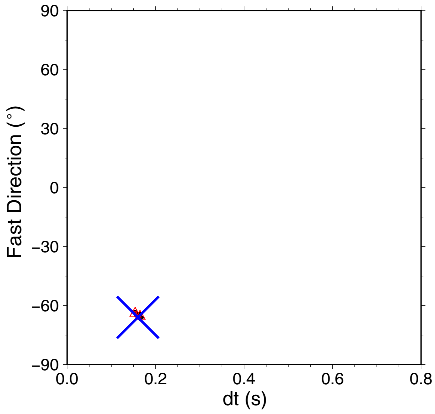

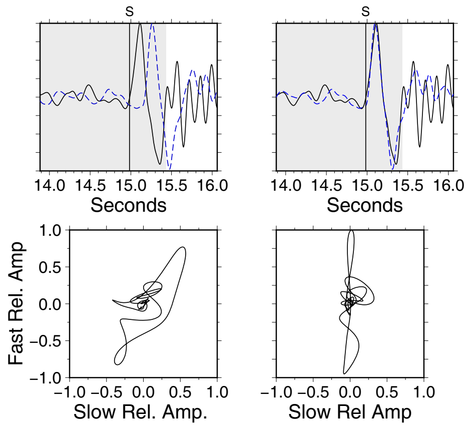

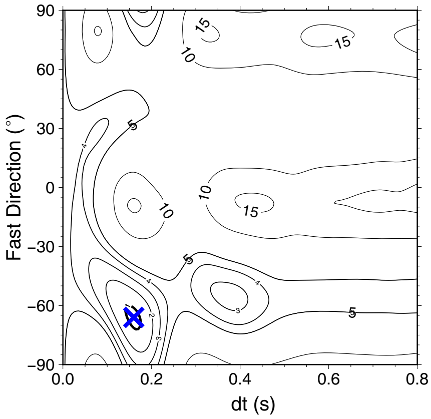

event 2021.168.19.48.rus.0.2−3.fb1

depth: 25 km
distance: 71.1813 km

splitting windows (relative to S−Pick at 14.98 s):
wbeg: −1.10 − −0.30 (5)
wend: 0.58 − 1.34 (15)
selected: 13.884 − 15.616, length: 1.732 s

results: GRADE ACI

fast: 114.0 +/− 3.2 (°)

dt: 0.160 +/− 0.007 (s)

spol: 1.8 +/− 1.3 (°)
