# Supplementary material for: Southern Tibetan rifting since late Miocene enabled by basal shear of the underthrusting Indian lithosphere
Source: Nat Commun. 2023 May 4;14:2565. doi: 10.1038/s41467-023-38296-w (PMC10160080; doi:10.1038/s41467-023-38296-w)
Supplement: Supplementary file 8 — Supplementary Data 6 [file 41467_2023_38296_MOESM8_ESM.zip › event 2021.170.20.01.bud.0.2−3.fb1.pdf]

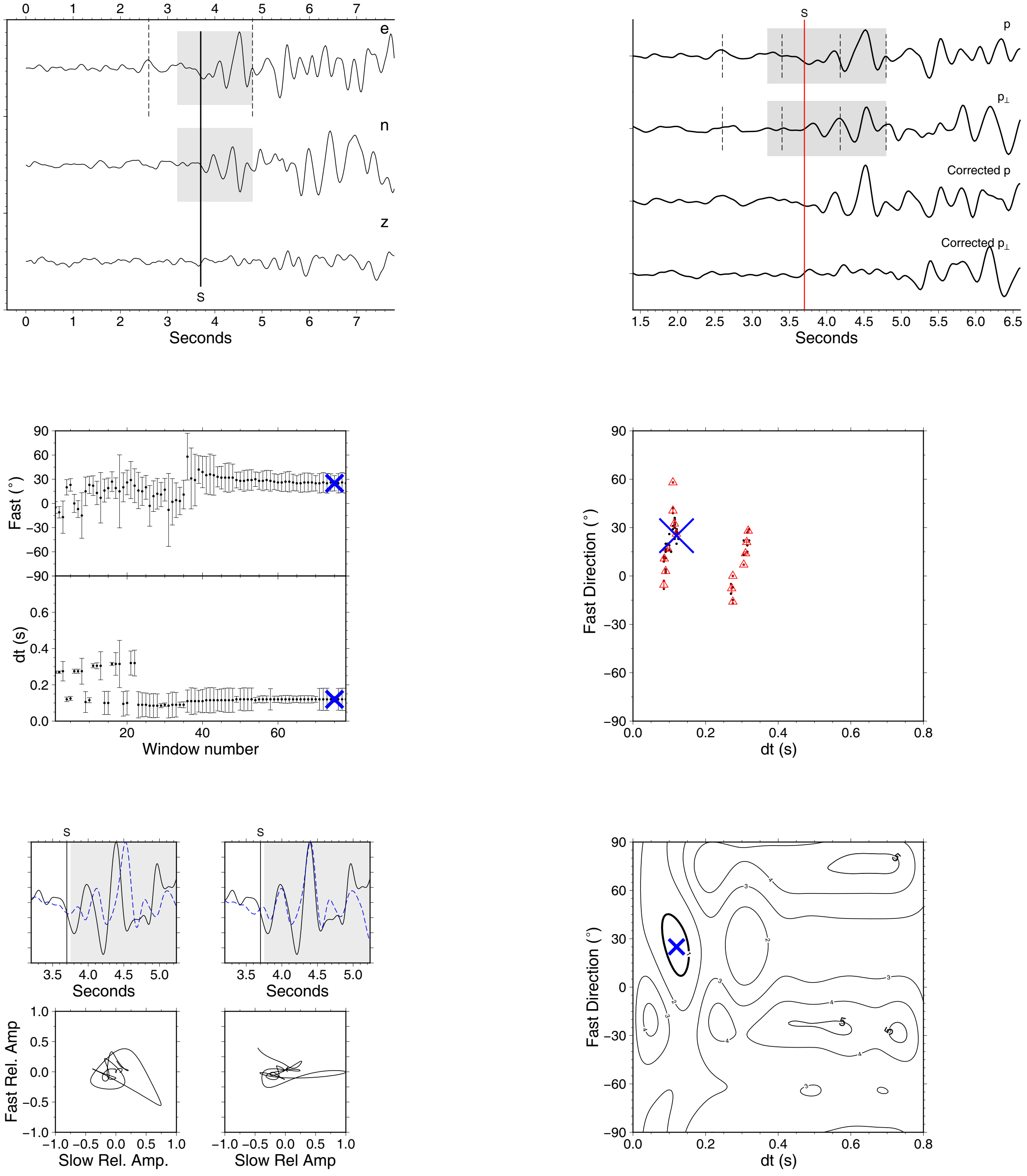

event 2021.170.20.01.bud.0.2−3.fb1

depth: 10 km
distance: 87.4047 km

splitting windows (relative to S−Pick at 3.70 s):
wbeg: −1.10 − −0.30 (5)
wend: 0.48 − 1.10 (16)
selected: 3.2003 − 4.7962, length: 1.5959 s

results: GRADE ACI

fast: 25.0 +/− 9.5 (°)

dt: 0.120 +/− 0.016 (s)

spol: 87.0 +/− 4.0 (°)
