# Supplementary material for: Southern Tibetan rifting since late Miocene enabled by basal shear of the underthrusting Indian lithosphere
Source: Nat Commun. 2023 May 4;14:2565. doi: 10.1038/s41467-023-38296-w (PMC10160080; doi:10.1038/s41467-023-38296-w)
Supplement: Supplementary file 8 — Supplementary Data 6 [file 41467_2023_38296_MOESM8_ESM.zip › event 2021.25.16.50.log.0.2−3.fb1.pdf]

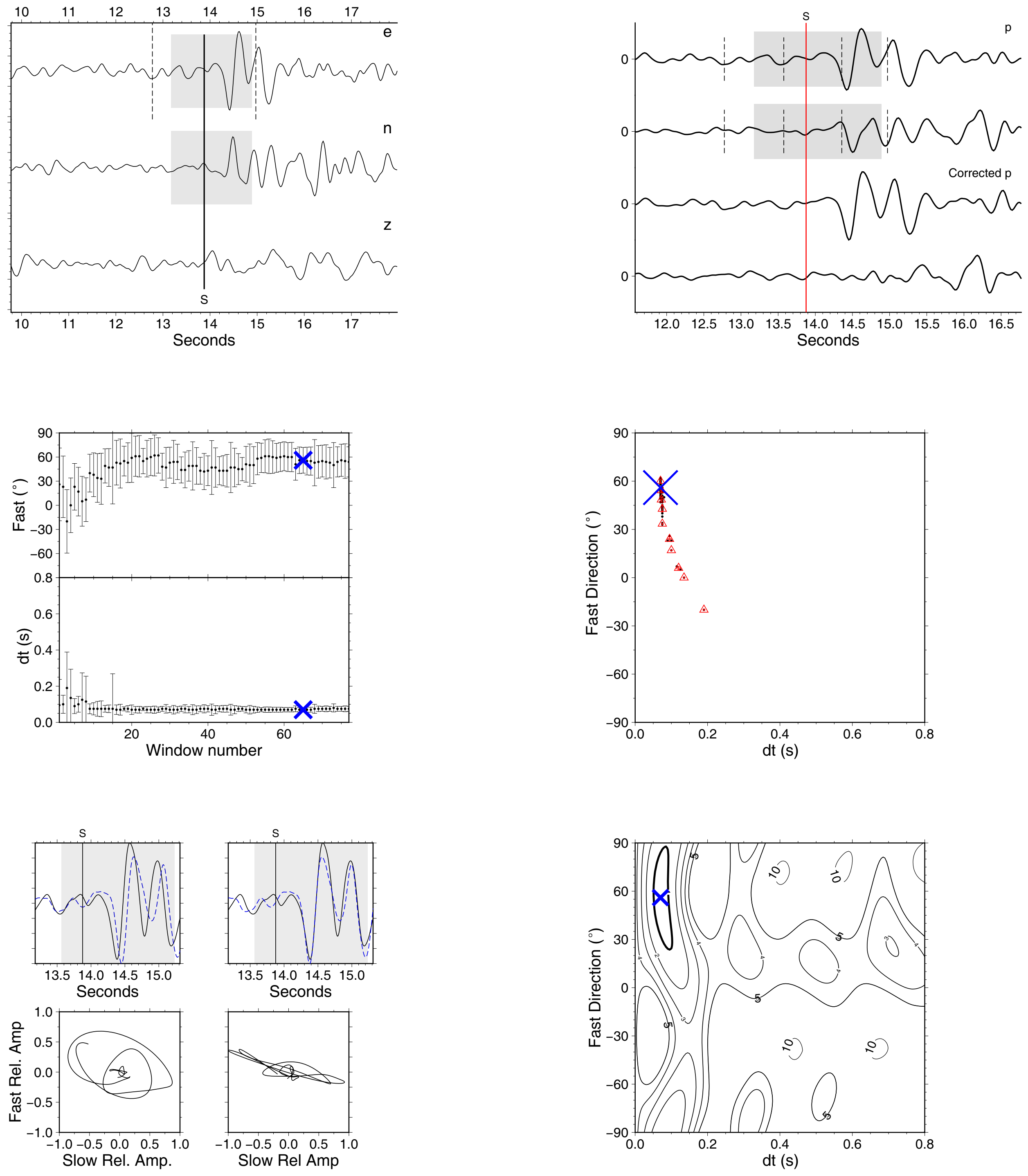

event 2021.25.16.50.log.0.2–3.fb1

---

depth: 25 km
distance: 60.3253 km

---

splitting windows (relative to S–Pick at 13.88 s):
wbeg: −1.10 – −0.30 (5)
wend: 0.48 – 1.10 (16)
selected: 13.176 – 14.89, length: 1.714 s

results: GRADE ACl

fast: 56.0 +/− 16.0 (°)

dt: 0.070 +/− 0.011 (s)

spol: 104.4 +/− 2.0 (°)
