# Supplementary material for: Southern Tibetan rifting since late Miocene enabled by basal shear of the underthrusting Indian lithosphere
Source: Nat Commun. 2023 May 4;14:2565. doi: 10.1038/s41467-023-38296-w (PMC10160080; doi:10.1038/s41467-023-38296-w)
Supplement: Supplementary file 8 — Supplementary Data 6 [file 41467_2023_38296_MOESM8_ESM.zip › event 2021.25.16.50.xis.0.2−3.fb1.pdf]

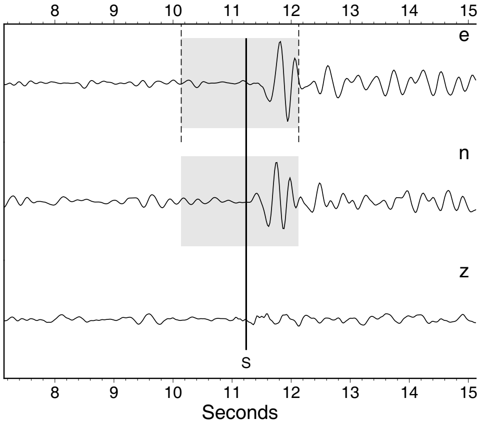

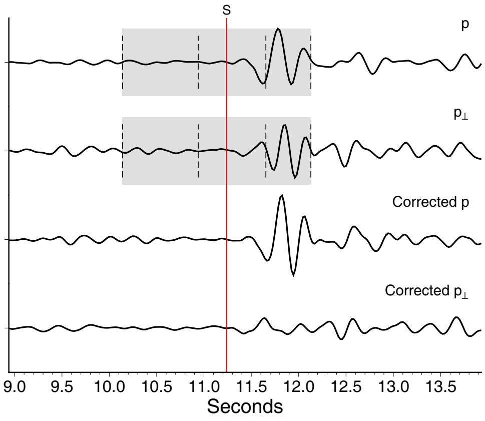

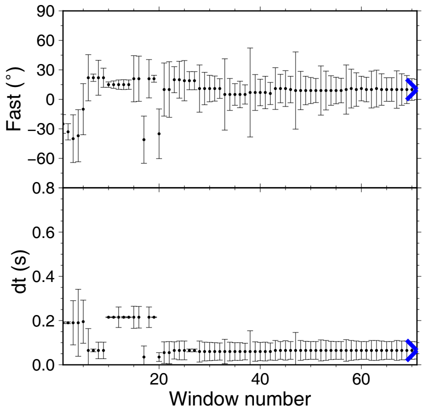

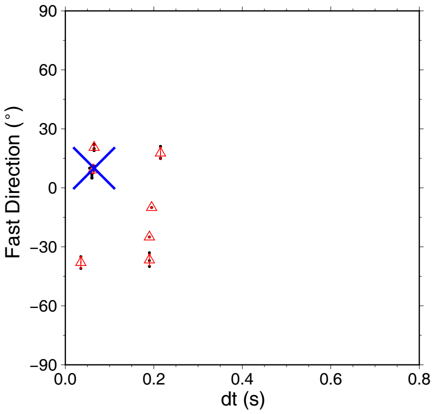

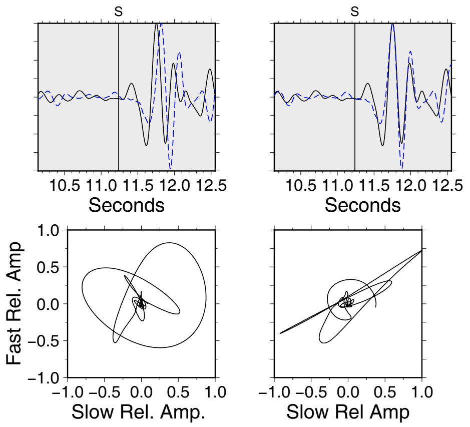

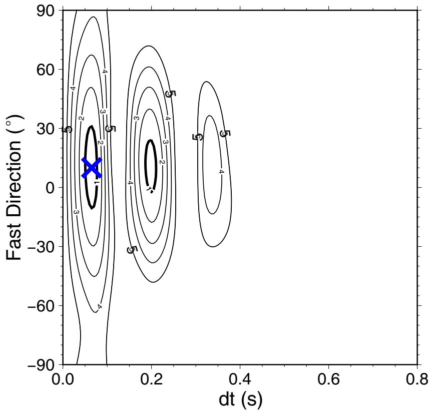

event 2021.25.16.50.xis.0.2−3.fb1

---

depth: 25 km
distance: 36.1588 km

---

splitting windows (relative to S−Pick at 11.24 s):
wbeg: −1.10 − −0.30 (5)
wend: 0.41 − 0.89 (15)
selected: 10.139 − 12.128, length: 1.989 s

results: GRADE BCI

fast: 10.0 +/− 10.2 (°)

dt: 0.065 +/− 0.039 (s)

spol: 56.5 +/− 3.3 (°)
