# Supplementary material for: Southern Tibetan rifting since late Miocene enabled by basal shear of the underthrusting Indian lithosphere
Source: Nat Commun. 2023 May 4;14:2565. doi: 10.1038/s41467-023-38296-w (PMC10160080; doi:10.1038/s41467-023-38296-w)
Supplement: Supplementary file 8 — Supplementary Data 6 [file 41467_2023_38296_MOESM8_ESM.zip › event 2021.25.21.32.qir.0.2−3.fb1.pdf]

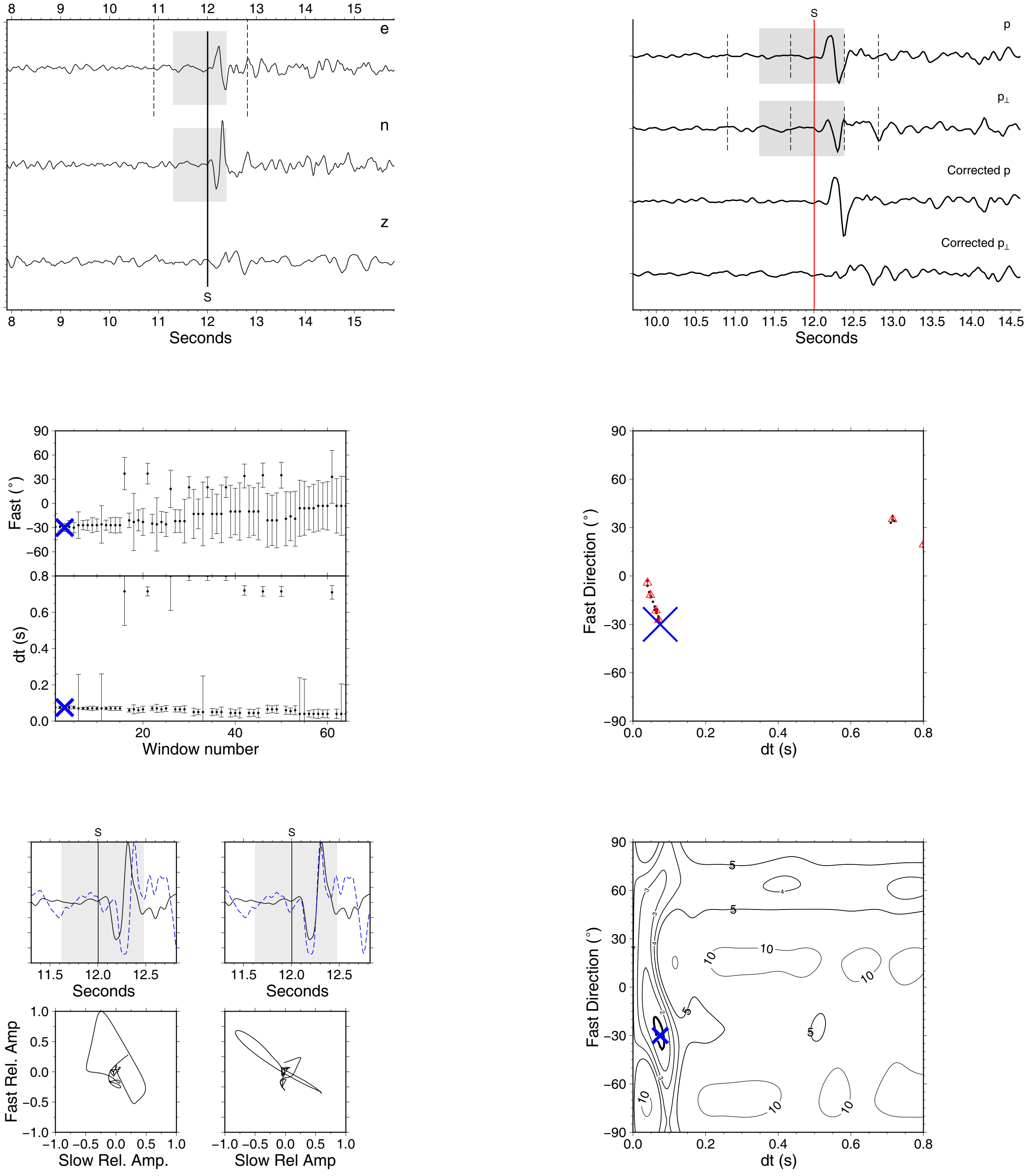

event 2021.25.21.32.qir.0.2−3.fb1

---

depth: 20 km
distance: 36.9469 km

---

splitting windows (relative to S−Pick at 12.00 s):
wbeg: −1.10 − −0.30 (5)
wend: 0.38 − 0.81 (15)
selected: 11.304 − 12.386, length: 1.082 s

results: GRADE ACl

fast: 150.0 +/− 5.0 (°)

dt: 0.075 +/− 0.006 (s)

spol: 126.1 +/− 2.0 (°)
