# Supplementary material for: Southern Tibetan rifting since late Miocene enabled by basal shear of the underthrusting Indian lithosphere
Source: Nat Commun. 2023 May 4;14:2565. doi: 10.1038/s41467-023-38296-w (PMC10160080; doi:10.1038/s41467-023-38296-w)
Supplement: Supplementary file 8 — Supplementary Data 6 [file 41467_2023_38296_MOESM8_ESM.zip › event 2021.40.06.21.qir.0.2−3.fb1.pdf]

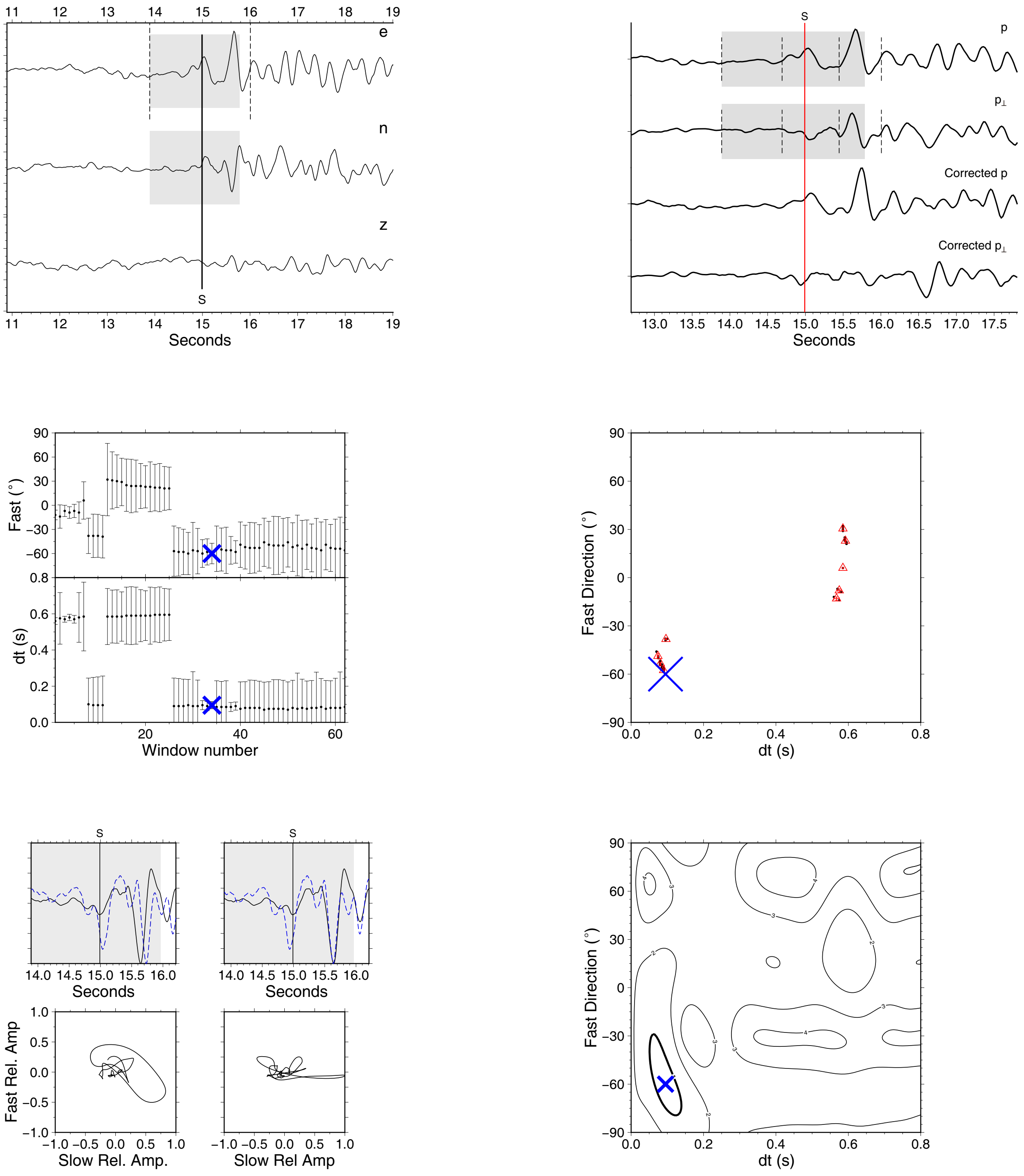

event 2021.40.06.21.qir.0.2−3.fb1

depth: 18 km
distance: 81.0881 km

splitting windows (relative to S−Pick at 14.99 s):
wbeg: −1.10 − −0.30 (5)
wend: 0.46 − 1.02 (16)
selected: 13.891 − 15.783, length: 1.892 s

results: GRADE ACl

fast: 120.0 +/− 12.8 (°)

dt: 0.095 +/− 0.020 (s)

spol: 89.6 +/− 5.5 (°)
