# Supplementary material for: Southern Tibetan rifting since late Miocene enabled by basal shear of the underthrusting Indian lithosphere
Source: Nat Commun. 2023 May 4;14:2565. doi: 10.1038/s41467-023-38296-w (PMC10160080; doi:10.1038/s41467-023-38296-w)
Supplement: Supplementary file 8 — Supplementary Data 6 [file 41467_2023_38296_MOESM8_ESM.zip › event 2021.43.04.53.qir.0.2−3.fb1.pdf]

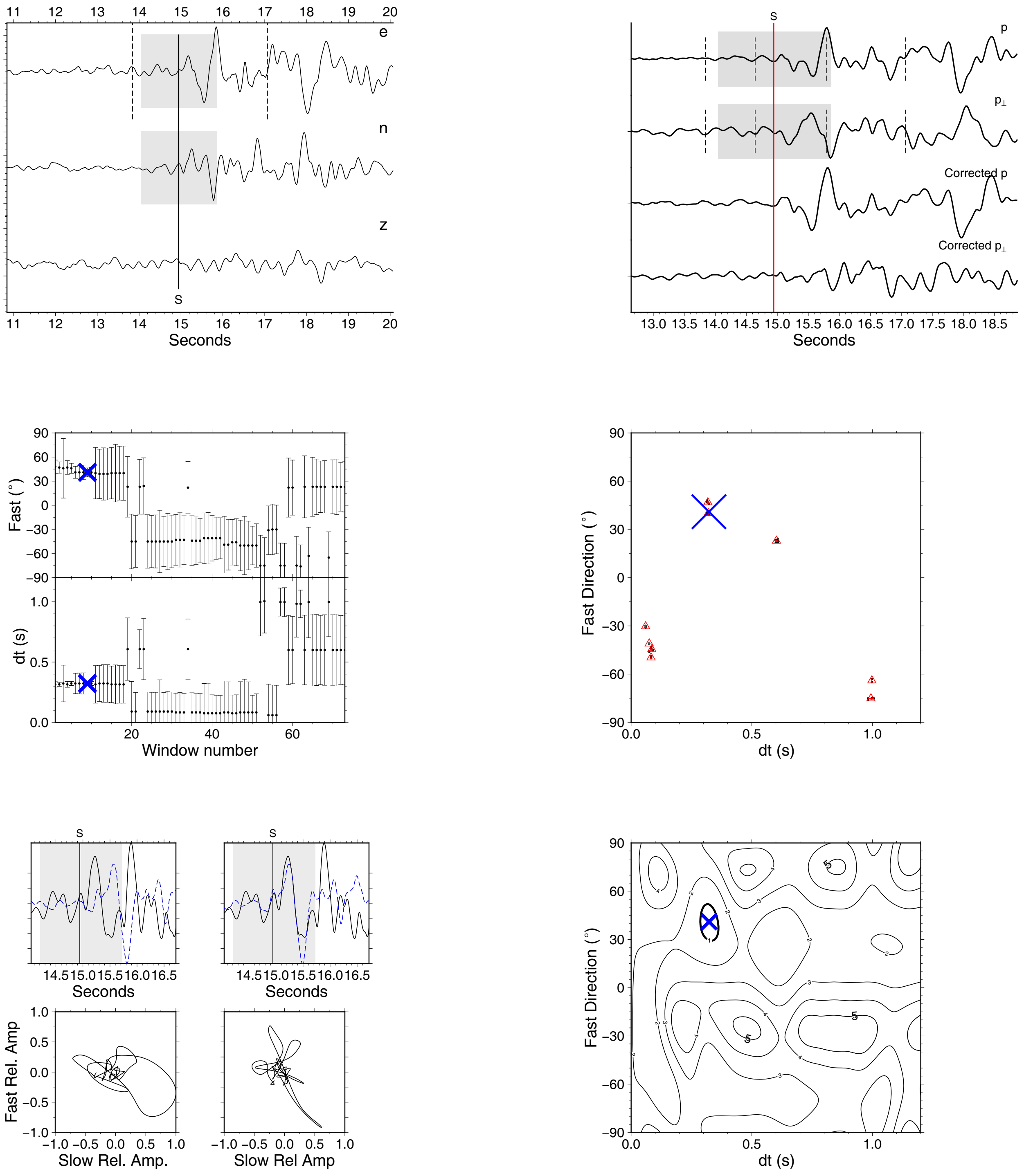

event 2021.43.04.53.qir.0.2-3.fb1

depth: 14 km
distance: 81.8943 km

splitting windows (relative to S−Pick at 14.94 s):
wbeg: −1.10 – −0.30 (5)
wend: 0.85 – 2.13 (17)
selected: 14.042 – 15.869, length: 1.827 s

results: GRADE ACI

fast: 41.0 +/− 5.5 (°)

dt: 0.323 +/− 0.017 (s)

spol: 155.7 +/− 3.9 (°)
