# Supplementary material for: Southern Tibetan rifting since late Miocene enabled by basal shear of the underthrusting Indian lithosphere
Source: Nat Commun. 2023 May 4;14:2565. doi: 10.1038/s41467-023-38296-w (PMC10160080; doi:10.1038/s41467-023-38296-w)
Supplement: Supplementary file 8 — Supplementary Data 6 [file 41467_2023_38296_MOESM8_ESM.zip › event 2021.46.17.00.log.0.2−3.fb1.pdf]

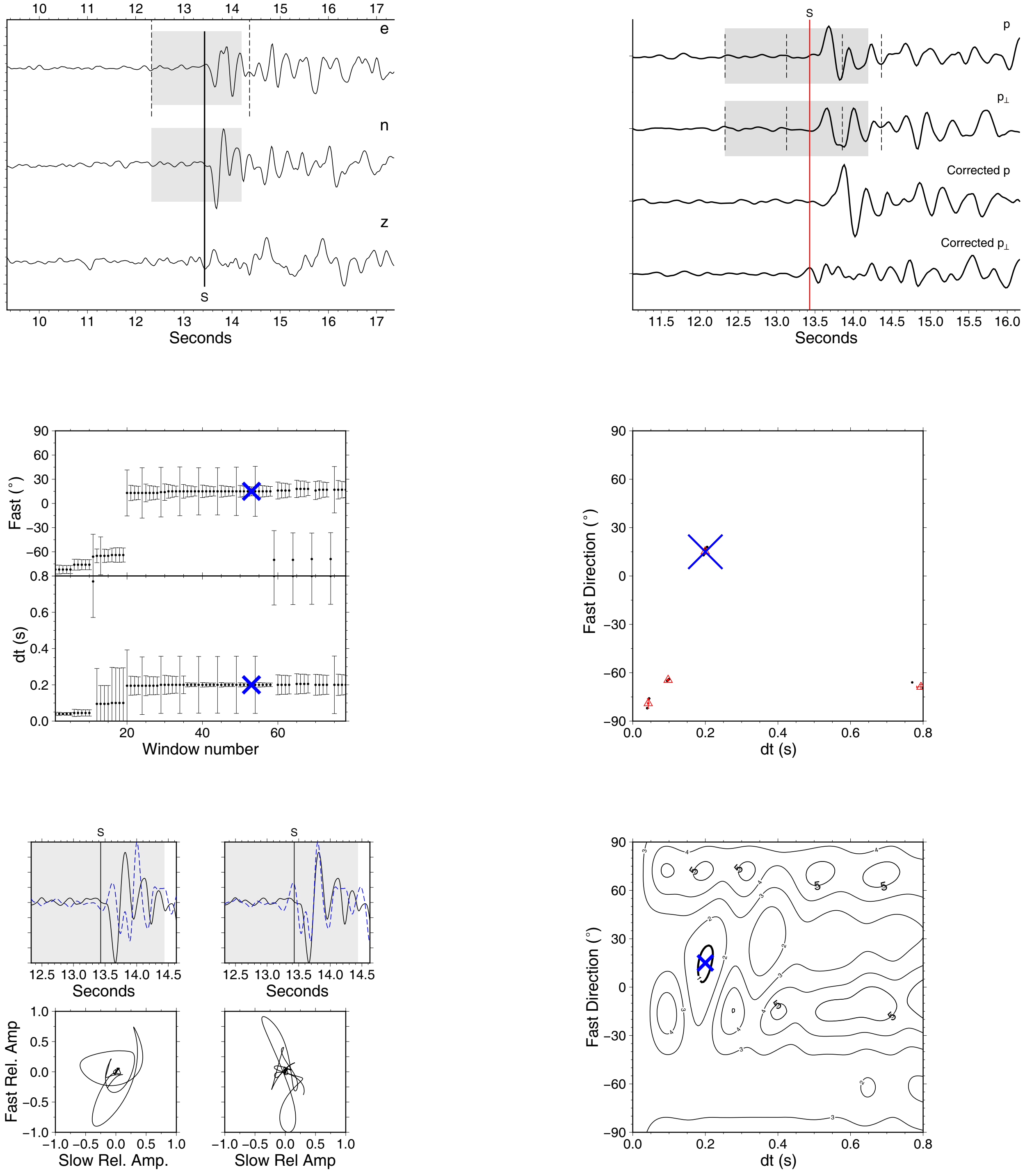

event 2021.46.17.00.log.0.2−3.fb1

depth: 25 km
distance: 51.2499 km

splitting windows (relative to S−Pick at 13.43 s):
wbeg: −1.10 − −0.30 (5)
wend: 0.43 − 0.93 (16)
selected: 12.33 − 14.195, length: 1.865 s

results: GRADE ACI

fast: 15.0 +/− 5.5 (°)

dt: 0.200 +/− 0.010 (s)

spol: 167.6 +/− 4.5 (°)
