# Supplementary material for: Southern Tibetan rifting since late Miocene enabled by basal shear of the underthrusting Indian lithosphere
Source: Nat Commun. 2023 May 4;14:2565. doi: 10.1038/s41467-023-38296-w (PMC10160080; doi:10.1038/s41467-023-38296-w)
Supplement: Supplementary file 8 — Supplementary Data 6 [file 41467_2023_38296_MOESM8_ESM.zip › event 2021.53.16.43.ruj.0.2−3.fb1.pdf]

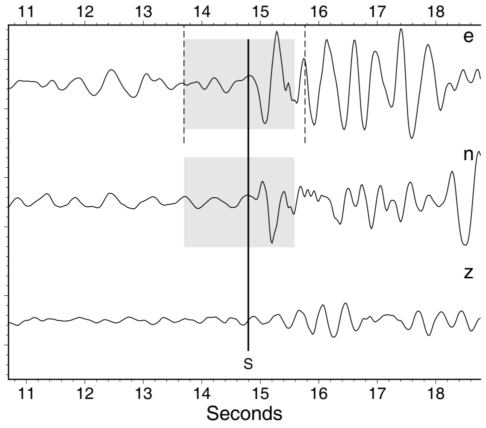

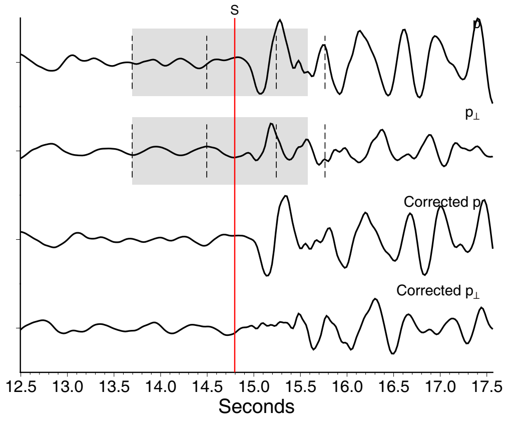

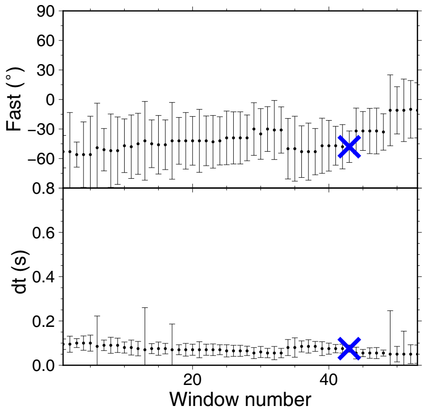

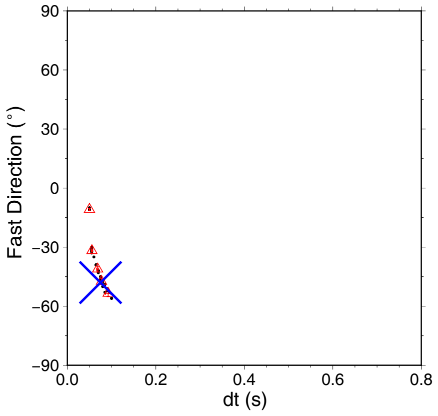

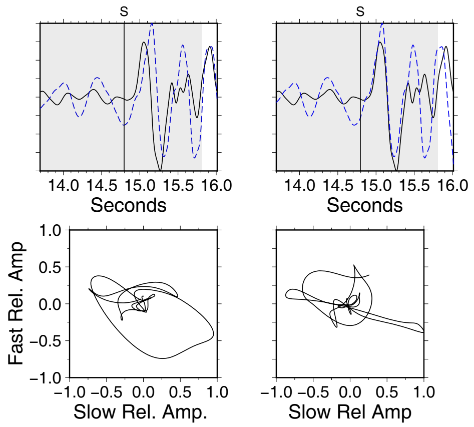

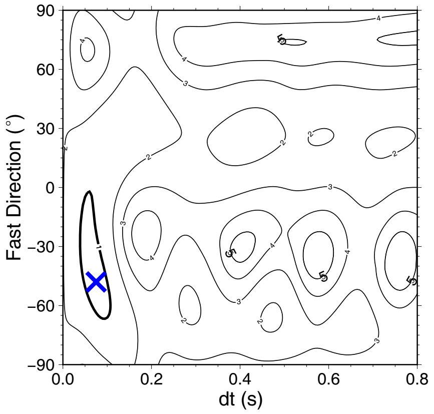

event 2021.53.16.43.ruj.0.2−3.fb1

depth: 25 km
distance: 84.0657 km

splitting windows (relative to S−Pick at 14.79 s):
wbeg: −1.10 − −0.30 (5)
wend: 0.45 − 0.97 (15)
selected: 13.695 − 15.578, length: 1.883 s

results: GRADE ACI

fast: 132.0 +/− 16.0 (°)

dt: 0.075 +/− 0.016 (s)

spol: 103.6 +/− 4.9 (°)
