# Supplementary material for: Southern Tibetan rifting since late Miocene enabled by basal shear of the underthrusting Indian lithosphere
Source: Nat Commun. 2023 May 4;14:2565. doi: 10.1038/s41467-023-38296-w (PMC10160080; doi:10.1038/s41467-023-38296-w)
Supplement: Supplementary file 8 — Supplementary Data 6 [file 41467_2023_38296_MOESM8_ESM.zip › event 2021.53.18.46.web.0.2−3.fb1.pdf]

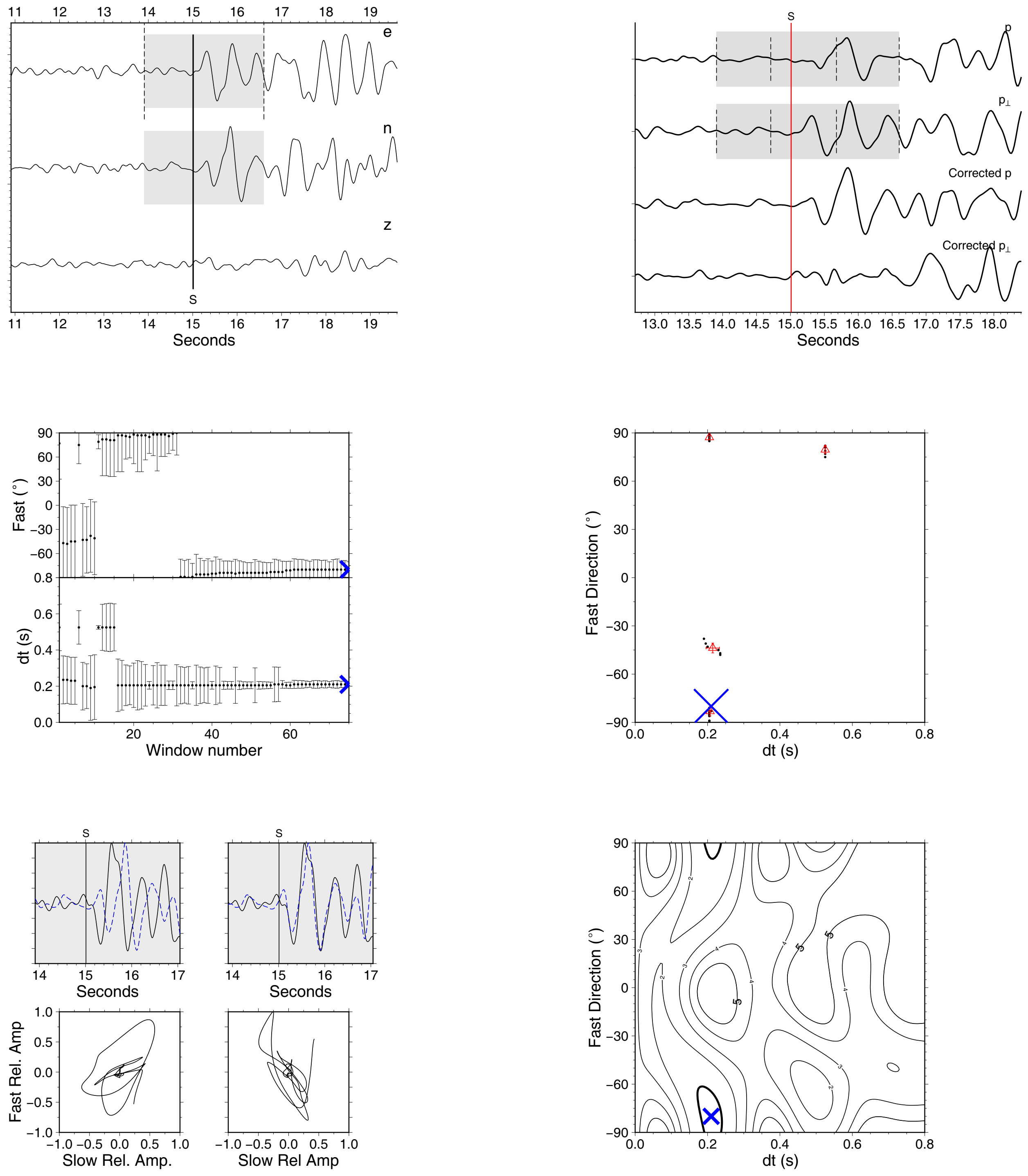

event 2021.53.18.46.web.0.2−3.fb1

depth: 11 km
distance: 97.4614 km

splitting windows (relative to S−Pick at 15.01 s):
wbeg: −1.10 − −0.30 (5)
wend: 0.67 − 1.59 (15)
selected: 13.91 − 16.603, length: 2.693 s

results: GRADE ACI

fast: 100.0 +/− 9.8 (°)

dt: 0.210 +/− 0.015 (s)

spol: 154.8 +/− 3.3 (°)
