# Supplementary material for: Southern Tibetan rifting since late Miocene enabled by basal shear of the underthrusting Indian lithosphere
Source: Nat Commun. 2023 May 4;14:2565. doi: 10.1038/s41467-023-38296-w (PMC10160080; doi:10.1038/s41467-023-38296-w)
Supplement: Supplementary file 8 — Supplementary Data 6 [file 41467_2023_38296_MOESM8_ESM.zip › event 2021.53.18.46.yar.0.2−3.fb1.pdf]

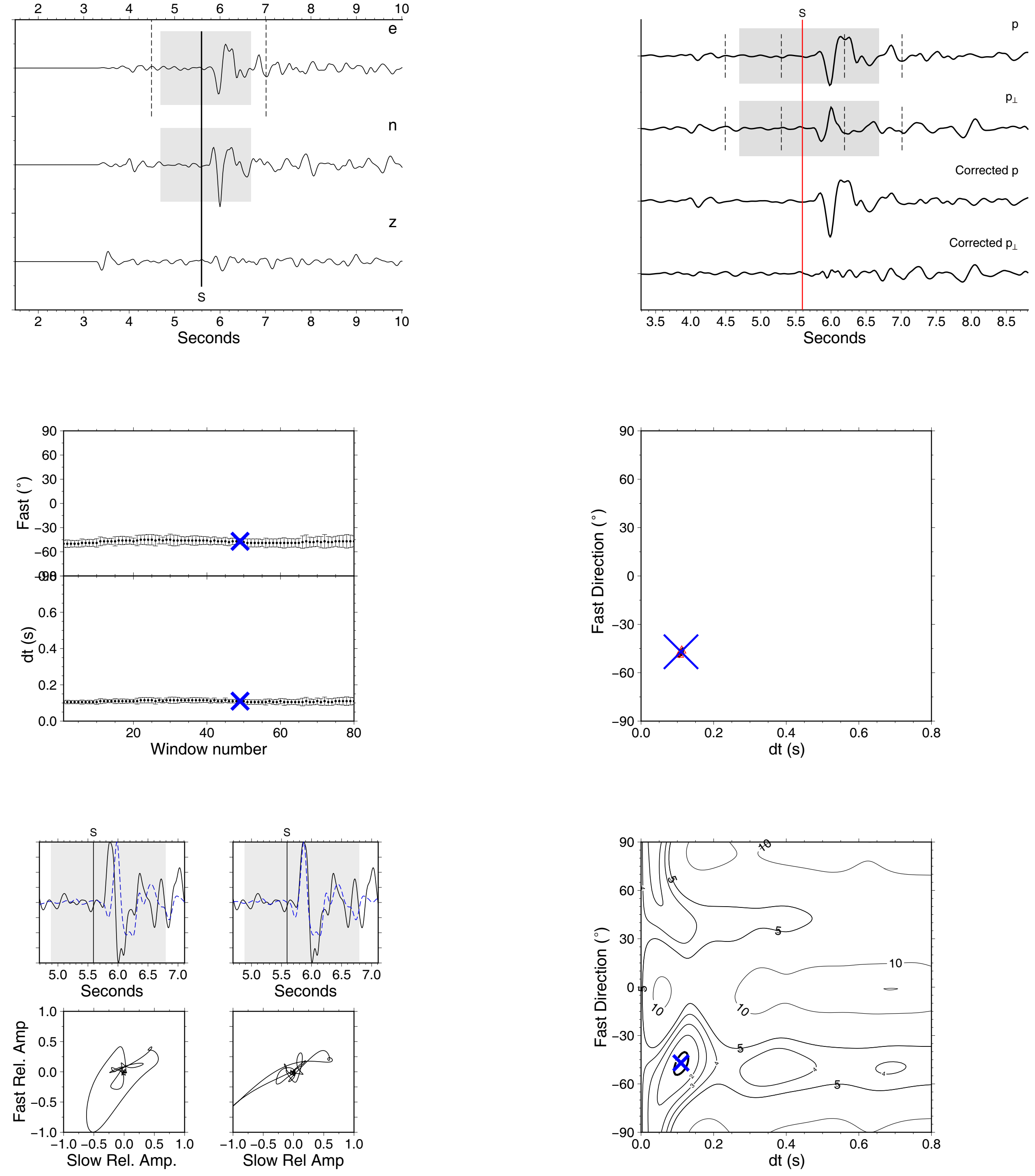

event 2021.53.18.46.yar.0.2–3.fb1

depth: 11 km
distance: 13.4029 km

splitting windows (relative to S–Pick at 5.59 s):
wbeg: −1.10 – −0.30 (5)
wend: 0.60 – 1.42 (16)
selected: 4.692 – 6.6841, length: 1.9921 s

results: GRADE ACl

fast: 133.0 +/– 3.5 (°)

dt: 0.110 +/– 0.009 (s)

spol: 64.0 +/– 1.7 (°)
