# Supplementary material for: Southern Tibetan rifting since late Miocene enabled by basal shear of the underthrusting Indian lithosphere
Source: Nat Commun. 2023 May 4;14:2565. doi: 10.1038/s41467-023-38296-w (PMC10160080; doi:10.1038/s41467-023-38296-w)
Supplement: Supplementary file 8 — Supplementary Data 6 [file 41467_2023_38296_MOESM8_ESM.zip › event 2021.68.19.23.jil.0.2−3.fb1.pdf]

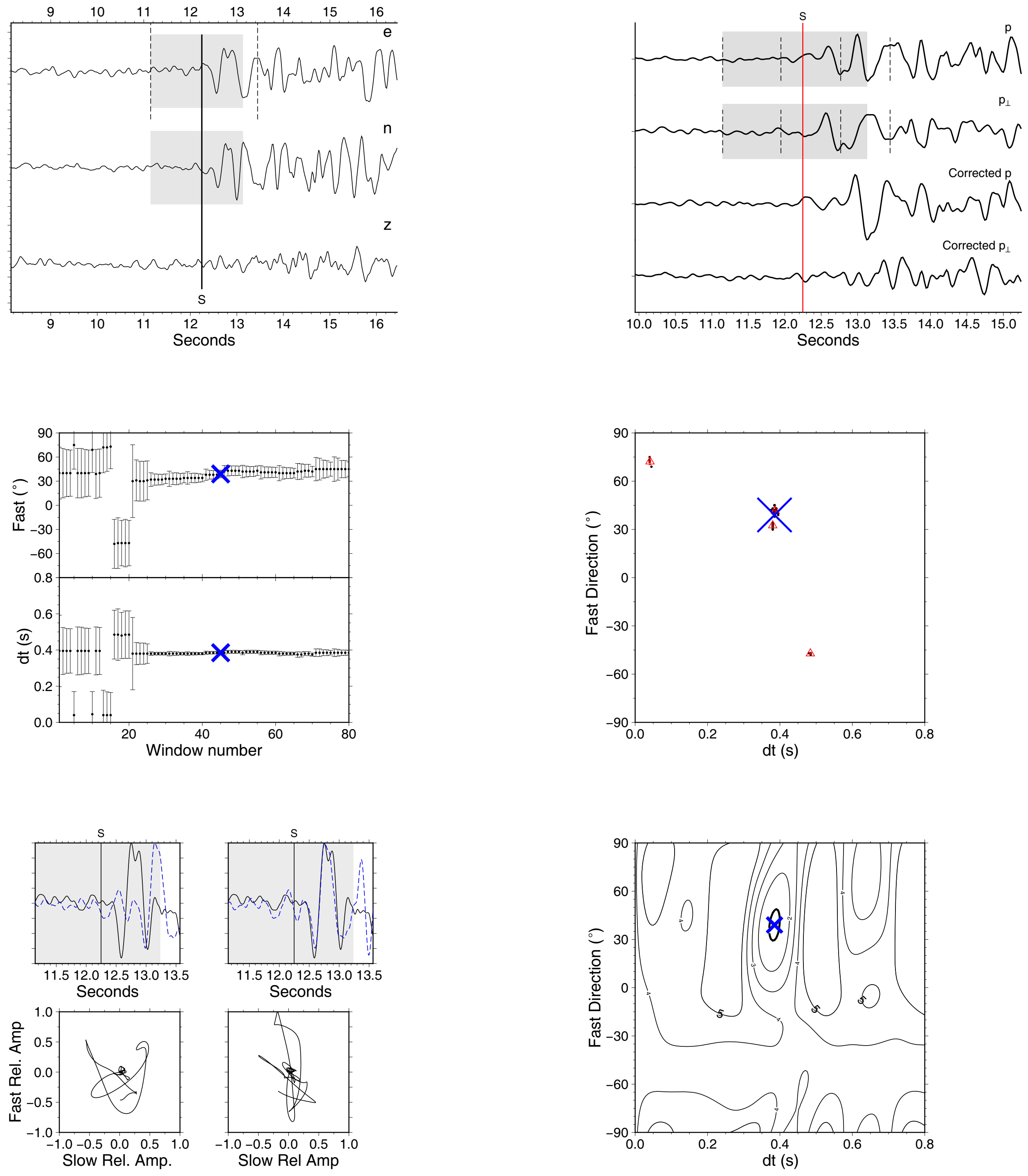

event 2021.68.19.23.jil.0.2−3.fb1

depth: 19 km
distance: 85.1675 km

splitting windows (relative to S−Pick at 12.25 s):
wbeg: −1.10 − −0.30 (5)
wend: 0.52 − 1.20 (16)
selected: 11.148 − 13.13, length: 1.982 s

results: GRADE ACl

fast: 39.0 +/− 4.8 (°)

dt: 0.385 +/− 0.005 (s)

spol: 171.0 +/− 2.8 (°)
