# Supplementary material for: Southern Tibetan rifting since late Miocene enabled by basal shear of the underthrusting Indian lithosphere
Source: Nat Commun. 2023 May 4;14:2565. doi: 10.1038/s41467-023-38296-w (PMC10160080; doi:10.1038/s41467-023-38296-w)
Supplement: Supplementary file 8 — Supplementary Data 6 [file 41467_2023_38296_MOESM8_ESM.zip › event 2021.68.19.23.yal.0.2−3.fb1.pdf]

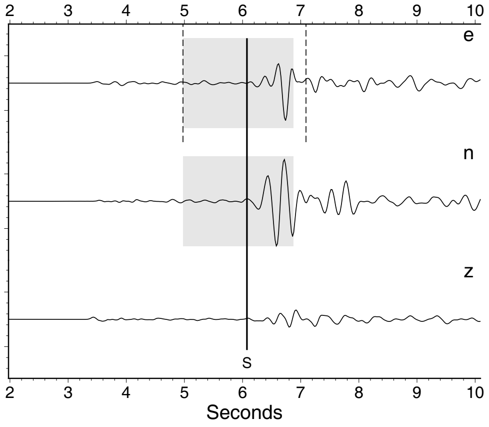

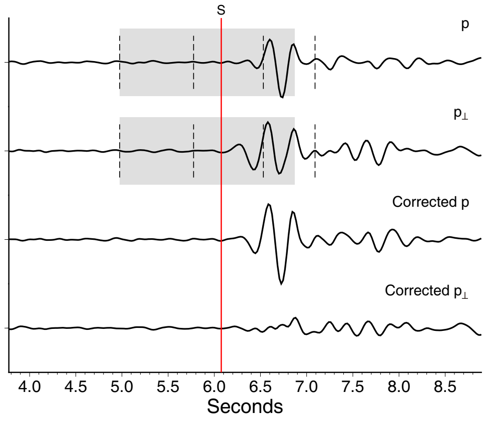

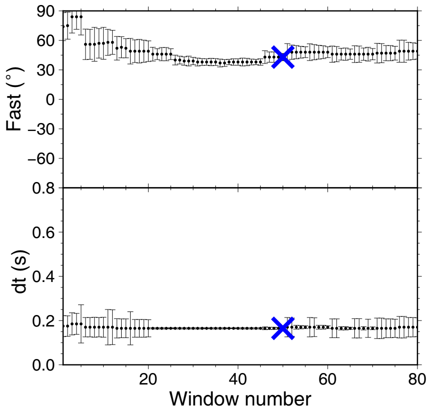

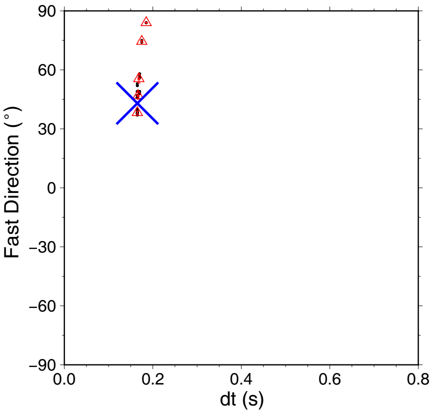

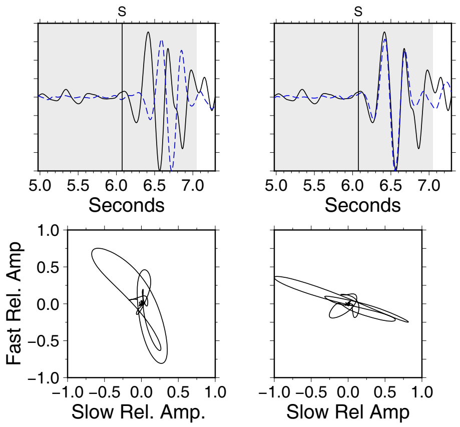

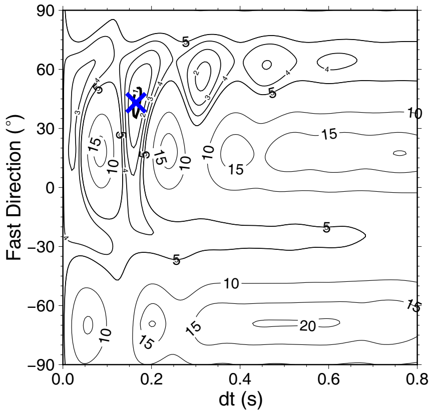

event 2021.68.19.23.yal.0.2–3.fb1

depth: 19 km
distance: 17.0174 km

splitting windows (relative to S–Pick at 6.08 s):
wbeg: –1.10 – –0.30 (5)
wend: 0.46 – 1.02 (16)
selected: 4.9751 – 6.8672, length: 1.8921 s

results: GRADE ACI

fast: 43.0 +/– 3.8 (°)

dt: 0.165 +/– 0.004 (s)

spol: 107.9 +/– 1.0 (°)
