# Supplementary material for: Southern Tibetan rifting since late Miocene enabled by basal shear of the underthrusting Indian lithosphere
Source: Nat Commun. 2023 May 4;14:2565. doi: 10.1038/s41467-023-38296-w (PMC10160080; doi:10.1038/s41467-023-38296-w)
Supplement: Supplementary file 8 — Supplementary Data 6 [file 41467_2023_38296_MOESM8_ESM.zip › event 2021.70.04.10.yal.0.2−3.fb1.pdf]

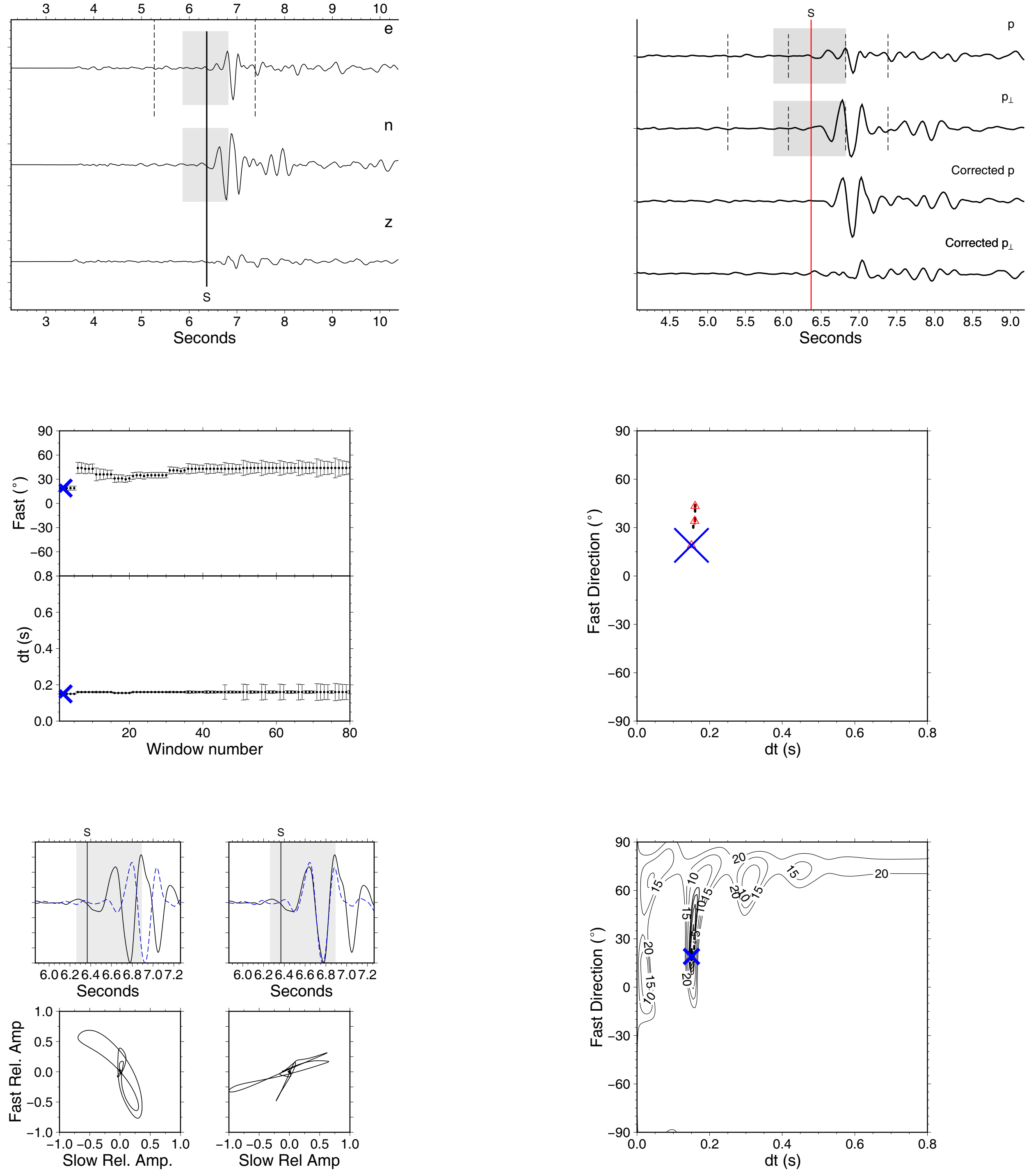

event 2021.70.04.10.yal.0.2−3.fb1

depth: 17 km
distance: 19.0141 km

splitting windows (relative to S−Pick at 6.37 s):
wbeg: −1.10 − −0.30 (5)
wend: 0.46 − 1.02 (16)
selected: 5.8666 − 6.8228, length: 0.9562 s

results: GRADE ACI

fast: 19.0 +/− 2.0 (°)

dt: 0.150 +/− 0.000 (s)

spol: 72.6 +/− 0.6 (°)
