# Supplementary material for: Southern Tibetan rifting since late Miocene enabled by basal shear of the underthrusting Indian lithosphere
Source: Nat Commun. 2023 May 4;14:2565. doi: 10.1038/s41467-023-38296-w (PMC10160080; doi:10.1038/s41467-023-38296-w)
Supplement: Supplementary file 8 — Supplementary Data 6 [file 41467_2023_38296_MOESM8_ESM.zip › event 2021.75.16.15.rus.0.2−3.fb1.pdf]

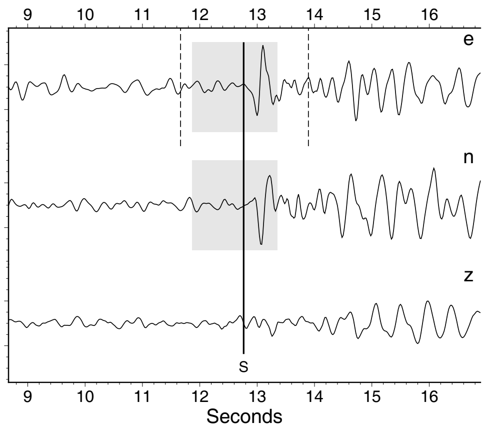

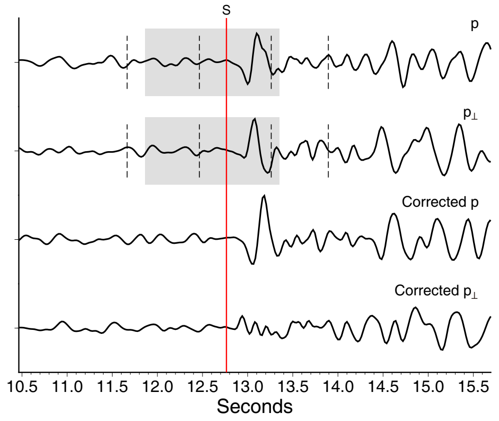

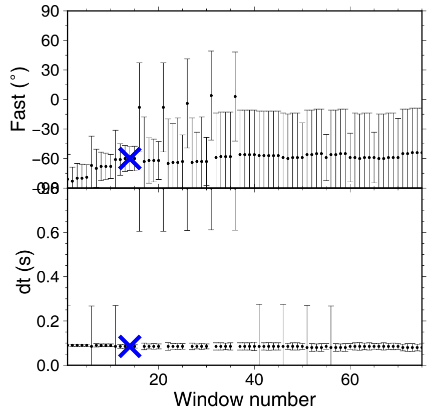

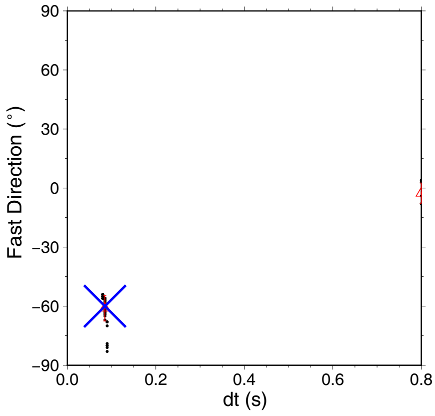

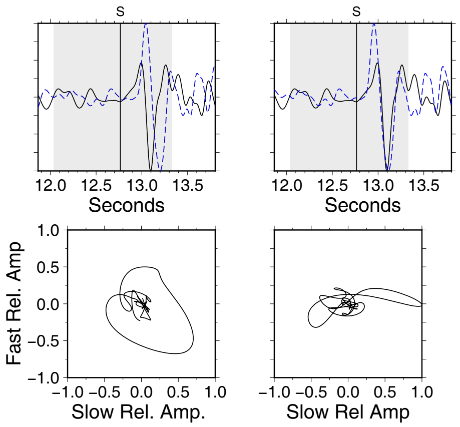

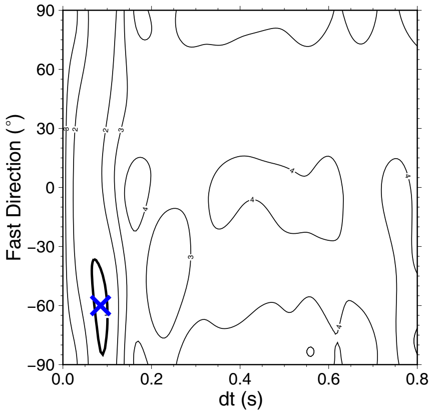

event 2021.75.16.15.rus.0.2–3.fb1

depth: 25 km
distance: 47.1218 km

splitting windows (relative to S–Pick at 12.77 s):
wbeg: −1.10 – −0.30 (5)
wend: 0.50 – 1.13 (15)
selected: 11.865 – 13.352, length: 1.487 s

results: GRADE ACl

fast: 120.0 +/− 12.0 (°)

dt: 0.085 +/− 0.007 (s)

spol: 83.8 +/− 5.7 (°)
