# Supplementary material for: Southern Tibetan rifting since late Miocene enabled by basal shear of the underthrusting Indian lithosphere
Source: Nat Commun. 2023 May 4;14:2565. doi: 10.1038/s41467-023-38296-w (PMC10160080; doi:10.1038/s41467-023-38296-w)
Supplement: Supplementary file 8 — Supplementary Data 6 [file 41467_2023_38296_MOESM8_ESM.zip › event 2021.75.17.42.rus.0.2−3.fb1.pdf]

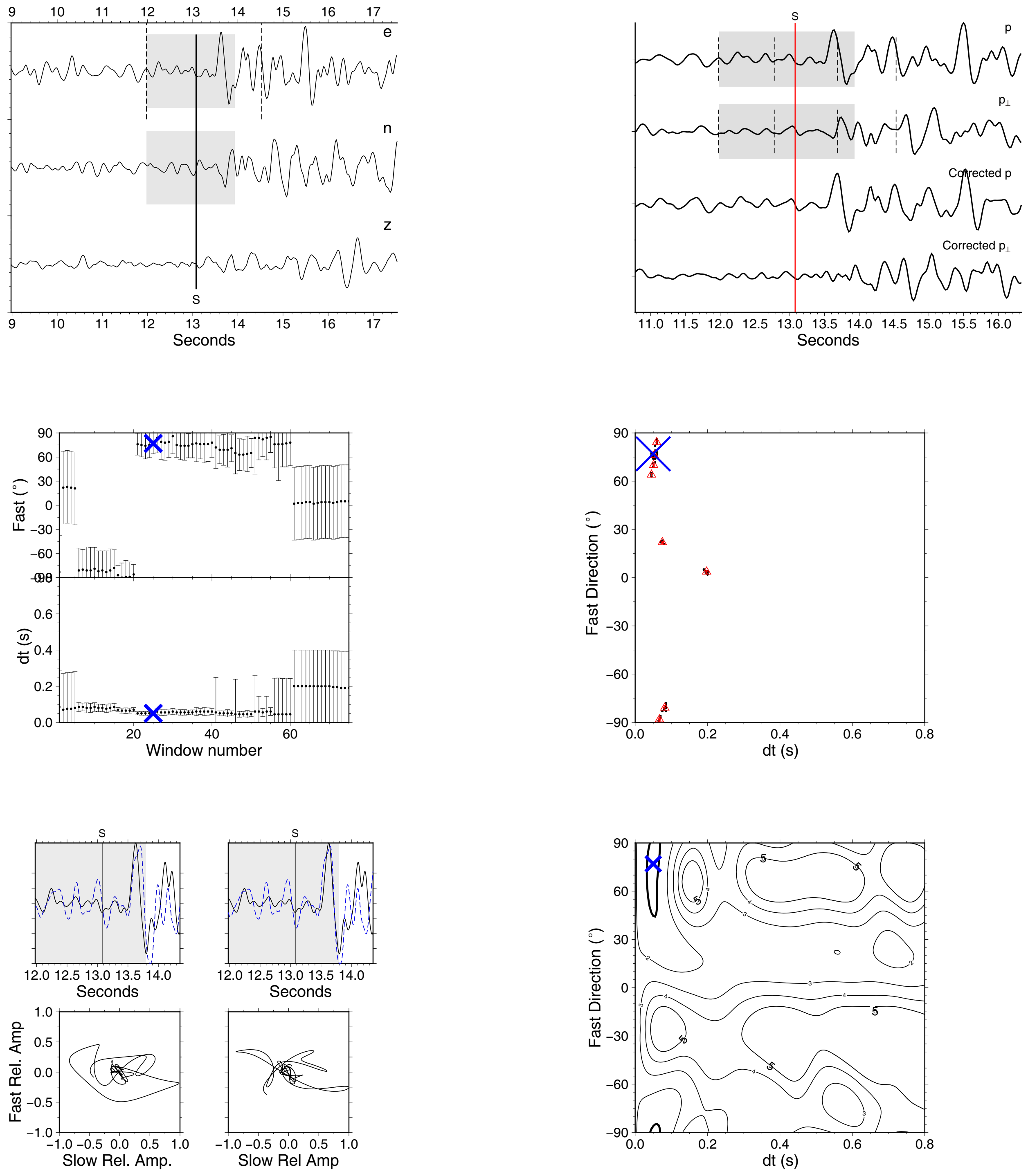

event 2021.75.17.42.rus.0.2−3.fb1

depth: 18 km
distance: 45.7371 km

splitting windows (relative to S−Pick at 13.08 s):
wbeg: −1.10 − −0.30 (5)
wend: 0.61 − 1.45 (15)
selected: 11.977 − 13.928, length: 1.951 s

results: GRADE ACI

fast: 77.0 +/− 13.0 (°)

dt: 0.050 +/− 0.007 (s)

spol: 113.5 +/− 3.4 (°)
