# Supplementary material for: Southern Tibetan rifting since late Miocene enabled by basal shear of the underthrusting Indian lithosphere
Source: Nat Commun. 2023 May 4;14:2565. doi: 10.1038/s41467-023-38296-w (PMC10160080; doi:10.1038/s41467-023-38296-w)
Supplement: Supplementary file 8 — Supplementary Data 6 [file 41467_2023_38296_MOESM8_ESM.zip › event 2021.75.20.02.rus.0.2−3.fb1.pdf]

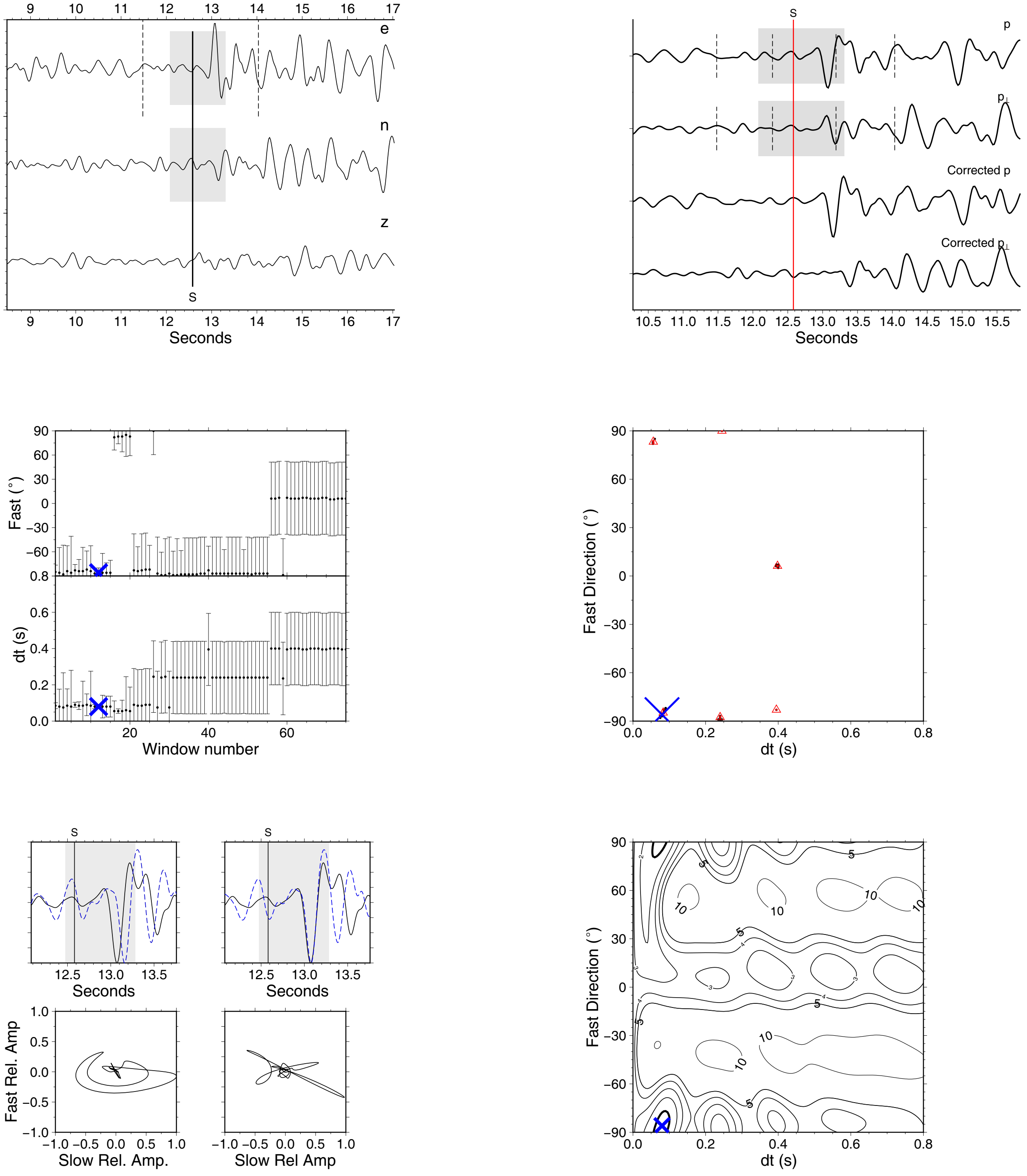

event 2021.75.20.02.rus.0.2–3.fb1

depth: 25 km
distance: 49.9467 km

splitting windows (relative to S–Pick at 12.58 s):
wbeg: −1.10 – −0.30 (5)
wend: 0.61 – 1.45 (15)
selected: 12.081 – 13.312, length: 1.231 s

results: GRADE ACI

fast: 94.0 +/– 6.0 (°)

dt: 0.080 +/– 0.011 (s)

spol: 115.3 +/– 1.5 (°)
