# Supplementary material for: Southern Tibetan rifting since late Miocene enabled by basal shear of the underthrusting Indian lithosphere
Source: Nat Commun. 2023 May 4;14:2565. doi: 10.1038/s41467-023-38296-w (PMC10160080; doi:10.1038/s41467-023-38296-w)
Supplement: Supplementary file 8 — Supplementary Data 6 [file 41467_2023_38296_MOESM8_ESM.zip › event 2021.80.08.30.log.0.2−3.fb1.pdf]

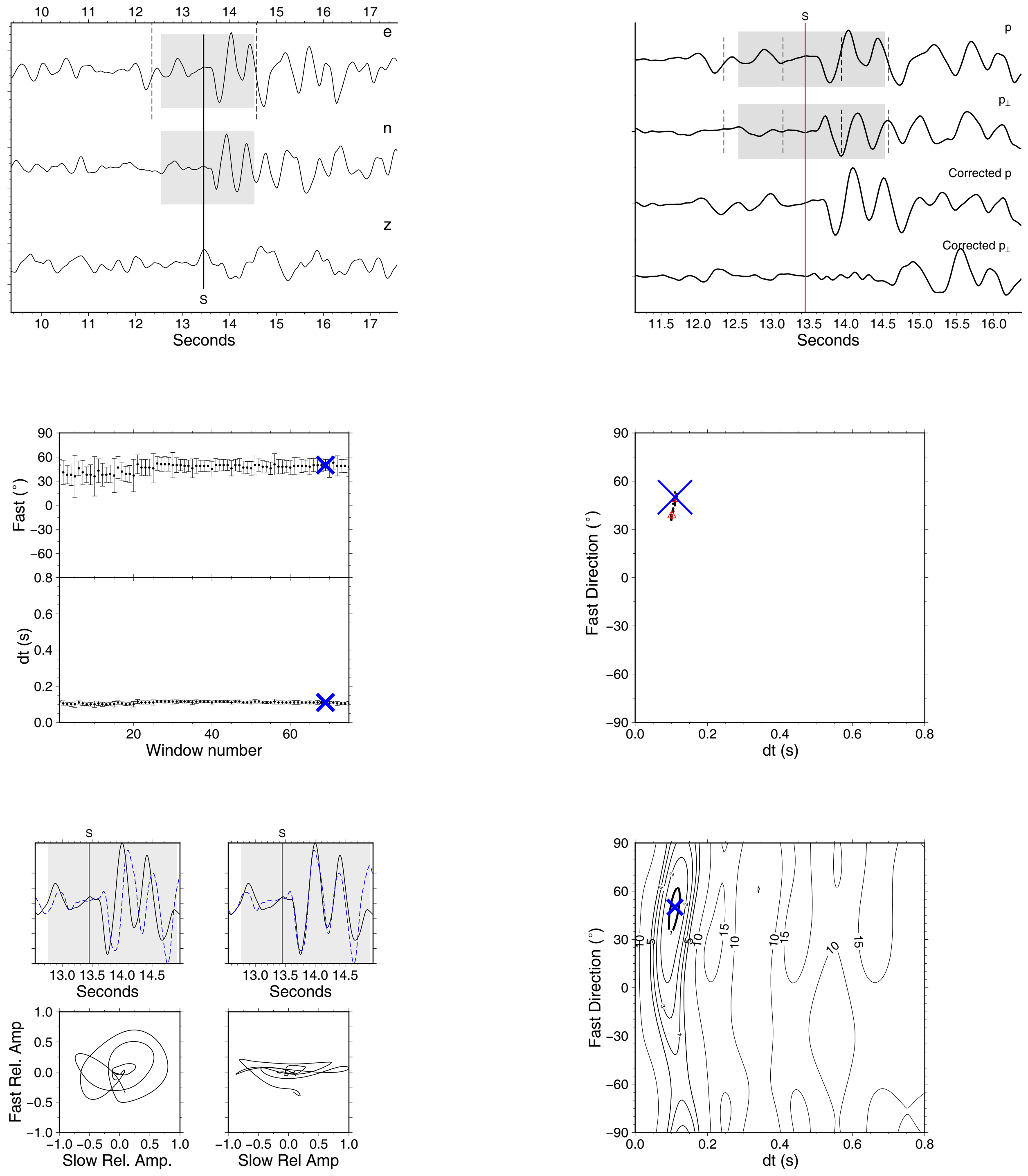

event 2021.80.08.30.log.0.2–3.fb1

depth: 25 km
distance: 58.0683 km

splitting windows (relative to S–Pick at 13.45 s):
wbeg: –1.10 – –0.30 (5)
wend: 0.49 – 1.12 (15)
selected: 12.549 – 14.527, length: 1.978 s

results: GRADE ACl

fast: 50.0 +/– 7.0 (°)

dt: 0.110 +/– 0.006 (s)

spol: 87.2 +/– 1.4 (°)
