# Supplementary material for: Southern Tibetan rifting since late Miocene enabled by basal shear of the underthrusting Indian lithosphere
Source: Nat Commun. 2023 May 4;14:2565. doi: 10.1038/s41467-023-38296-w (PMC10160080; doi:10.1038/s41467-023-38296-w)
Supplement: Supplementary file 8 — Supplementary Data 6 [file 41467_2023_38296_MOESM8_ESM.zip › event 2021.80.08.30.xis.0.2−3.fb1.pdf]

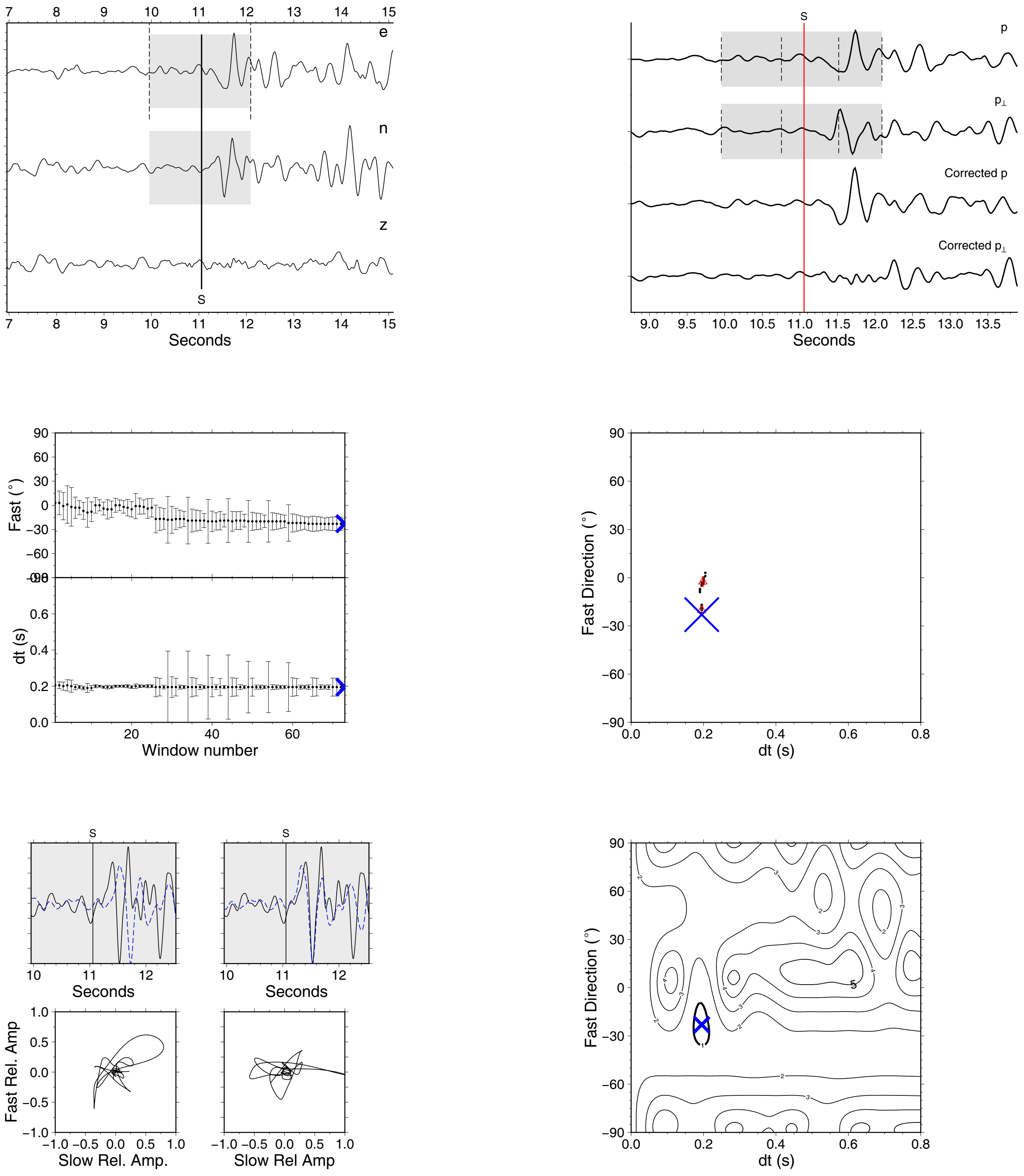

event 2021.80.08.30.xis.0.2−3.fb1

depth: 25 km
distance: 41.4305 km

splitting windows (relative to S−Pick at 11.06 s):
wbeg: −1.10 − −0.30 (5)
wend: 0.46 − 1.04 (15)
selected: 9.9563 − 12.092, length: 2.1357 s

results: GRADE ACl

fast: 157.0 +/− 6.5 (°)

dt: 0.195 +/− 0.010 (s)

spol: 90.9 +/− 4.7 (°)
