# Supplementary material for: Southern Tibetan rifting since late Miocene enabled by basal shear of the underthrusting Indian lithosphere
Source: Nat Commun. 2023 May 4;14:2565. doi: 10.1038/s41467-023-38296-w (PMC10160080; doi:10.1038/s41467-023-38296-w)
Supplement: Supplementary file 8 — Supplementary Data 6 [file 41467_2023_38296_MOESM8_ESM.zip › event 2021.85.22.16.dax.0.2−3.fb1.pdf]

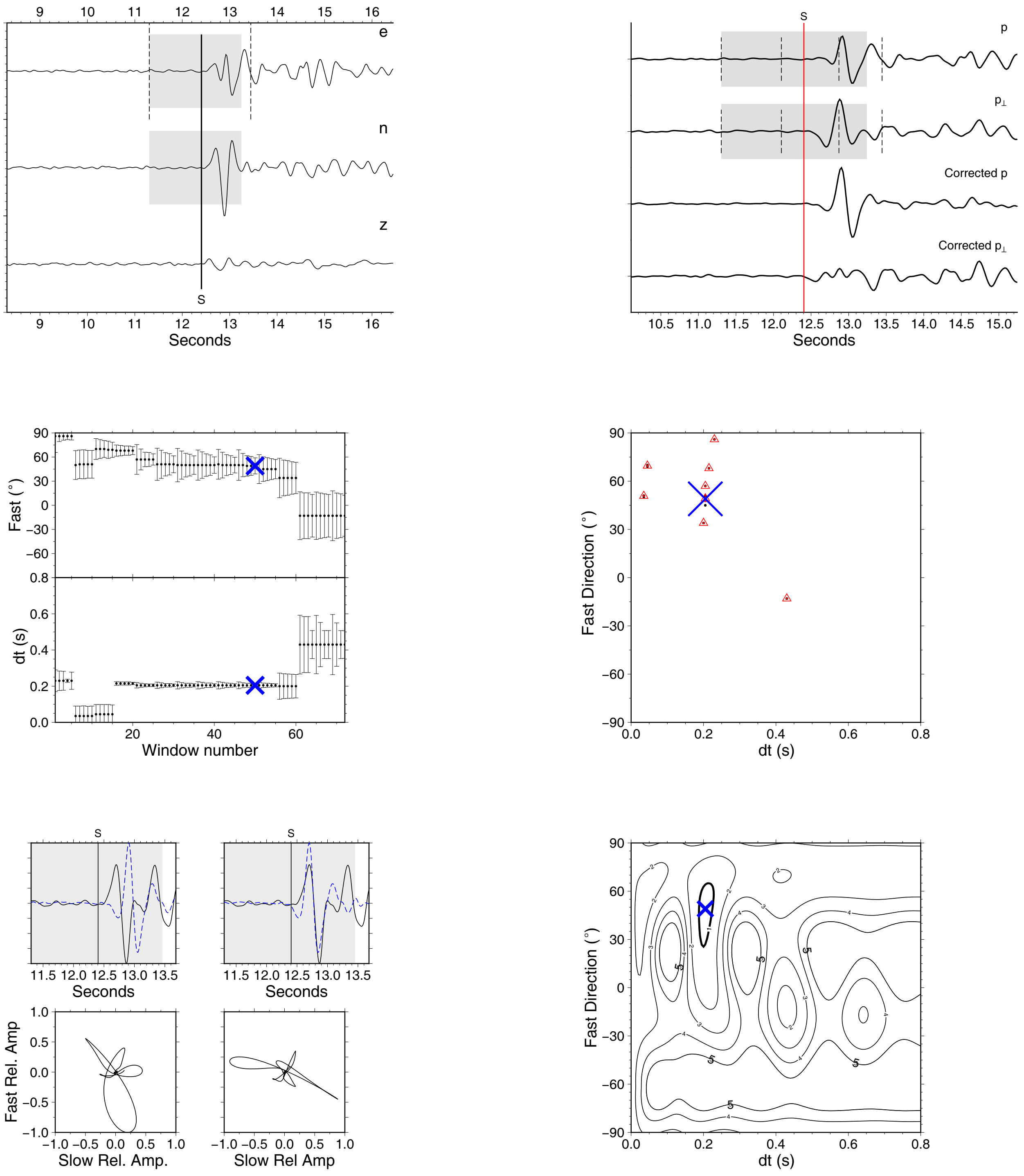

event 2021.85.22.16.dax.0.2−3.fb1

depth: 25 km
distance: 36.9061 km

splitting windows (relative to S−Pick at 12.40 s):
wbeg: −1.10 − −0.30 (5)
wend: 0.47 − 1.04 (15)
selected: 11.304 − 13.24, length: 1.936 s

results: GRADE ACl

fast: 49.0 +/− 10.0 (°)

dt: 0.205 +/− 0.009 (s)

spol: 111.0 +/− 3.0 (°)
