# Supplementary material for: Southern Tibetan rifting since late Miocene enabled by basal shear of the underthrusting Indian lithosphere
Source: Nat Commun. 2023 May 4;14:2565. doi: 10.1038/s41467-023-38296-w (PMC10160080; doi:10.1038/s41467-023-38296-w)
Supplement: Supplementary file 8 — Supplementary Data 6 [file 41467_2023_38296_MOESM8_ESM.zip › event 2021.97.11.36.yal.0.2−3.fb1.pdf]

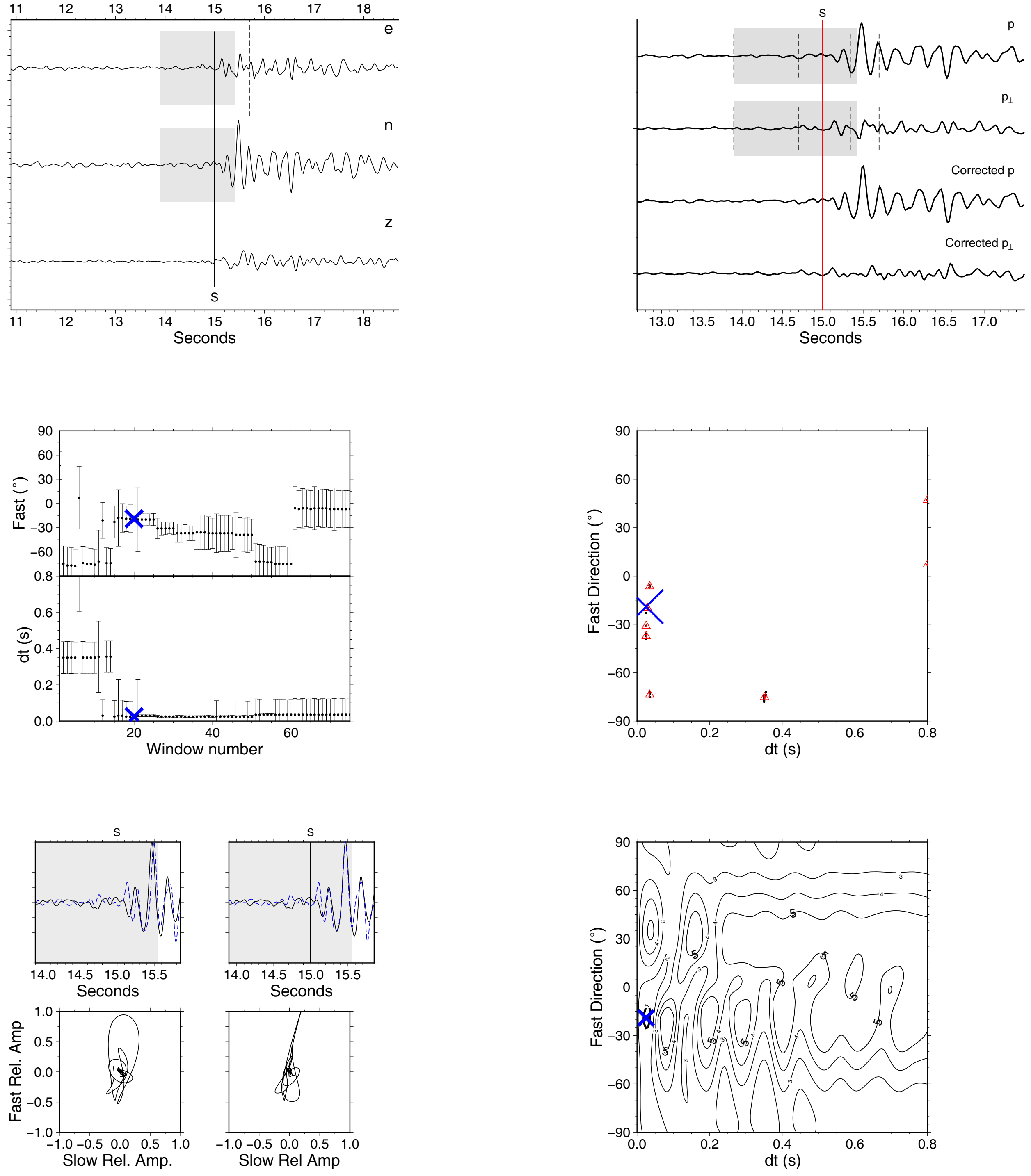

event 2021.97.11.36.yal.0.2−3.fb1

depth: 25 km
distance: 60.6477 km

splitting windows (relative to S−Pick at 15.00 s):
wbeg: −1.10 − −0.30 (5)
wend: 0.34 − 0.70 (15)
selected: 13.896 − 15.417, length: 1.521 s

results: GRADE ACI

fast: 161.0 +/− 3.5 (°)

dt: 0.025 +/− 0.004 (s)

spol: 11.0 +/− 7.9 (°)
