# Supplementary figures and images for: Southern Tibetan rifting since late Miocene enabled by basal shear of the underthrusting Indian lithosphere (part 4 of 4)
Source: Nat Commun. 2023 May 4;14:2565. doi: 10.1038/s41467-023-38296-w (PMC10160080; doi:10.1038/s41467-023-38296-w)

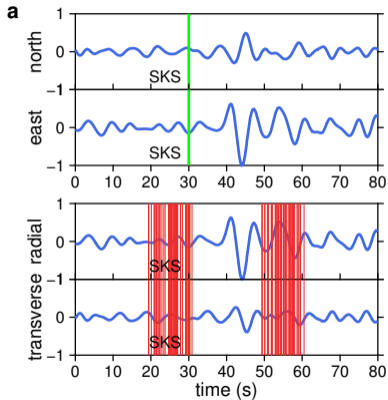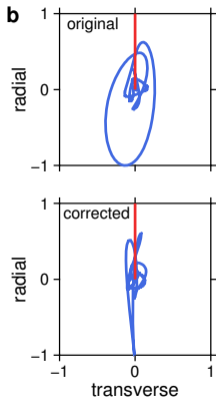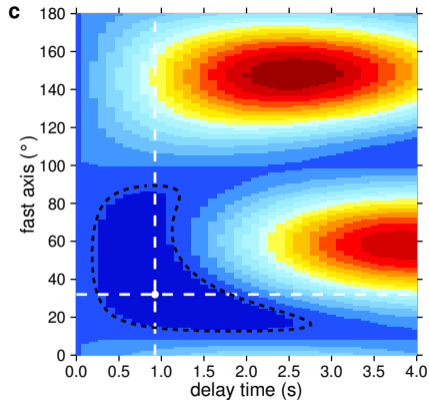

Supplement: Supplementary file 10 — Supplementary Data 8 [file 41467_2023_38296_MOESM10_ESM.zip › TP_CUQ_18-Feb-2021_15_30_52_SKS_average.pdf]

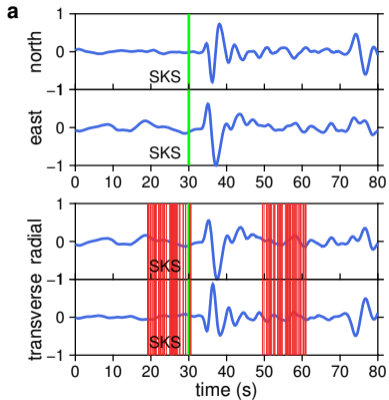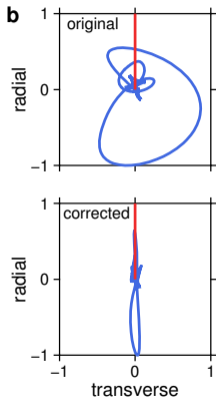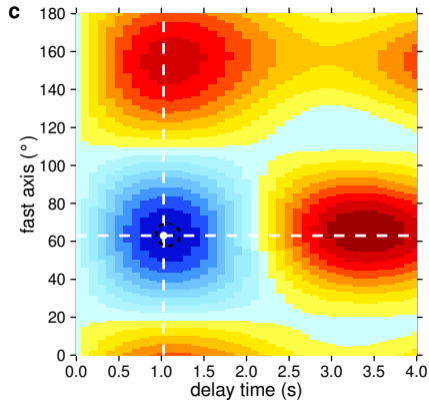

Supplement: Supplementary file 10 — Supplementary Data 8 [file 41467_2023_38296_MOESM10_ESM.zip › TP_CUQ_21-Oct-2019_02_52_29_SKS_good.pdf]

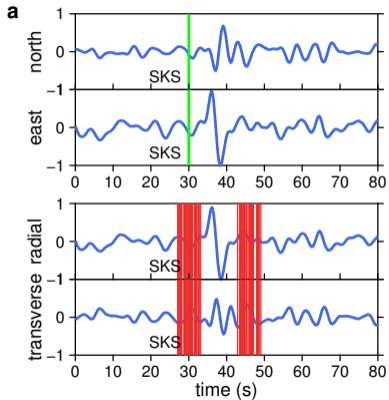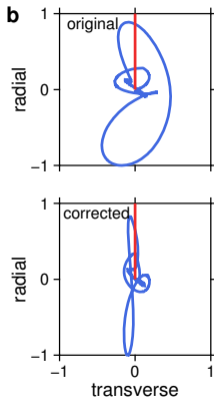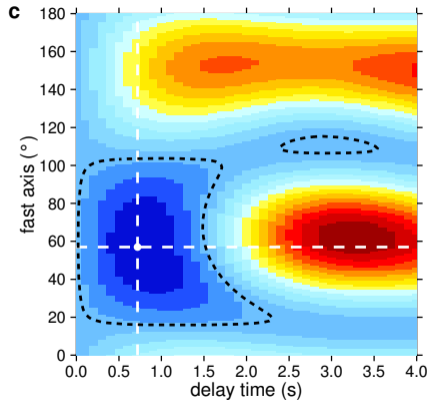

Supplement: Supplementary file 10 — Supplementary Data 8 [file 41467_2023_38296_MOESM10_ESM.zip › TP_CUQ_22-Apr-2020_22_31_25_SKS_average.pdf]

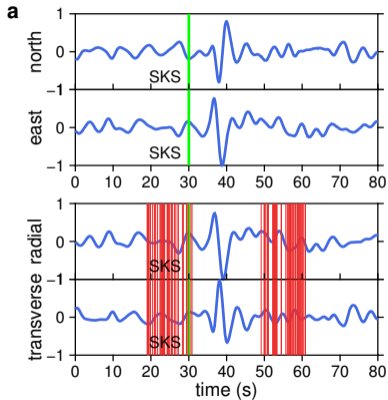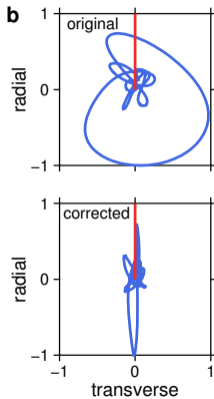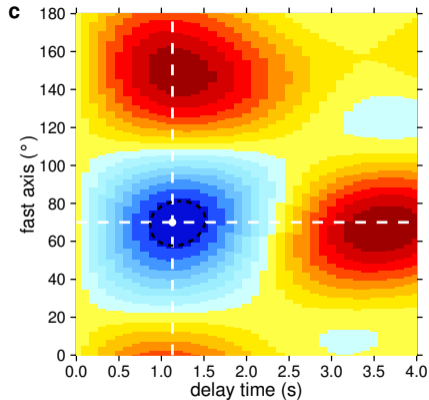

Supplement: Supplementary file 10 — Supplementary Data 8 [file 41467_2023_38296_MOESM10_ESM.zip › TP_CUQ_23-Mar-2020_20_33_39_SKS_good.pdf]

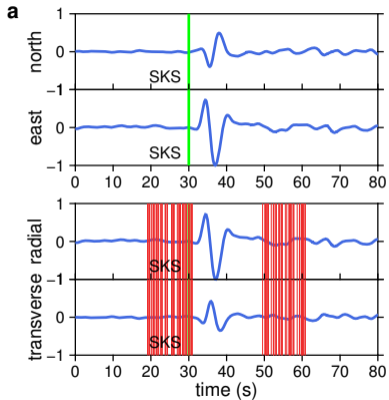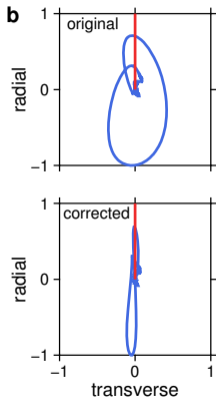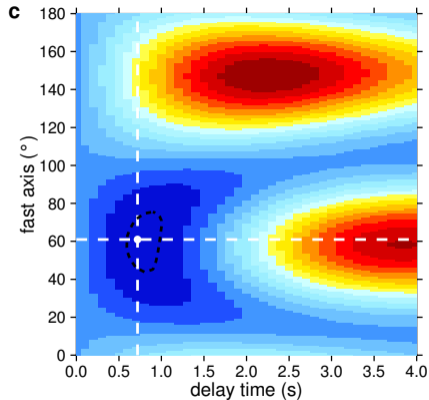

Supplement: Supplementary file 10 — Supplementary Data 8 [file 41467_2023_38296_MOESM10_ESM.zip › TP_CUQ_24-Apr-2021_00_23_35_SKS_good.pdf]

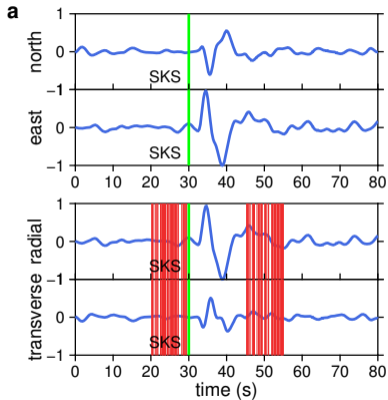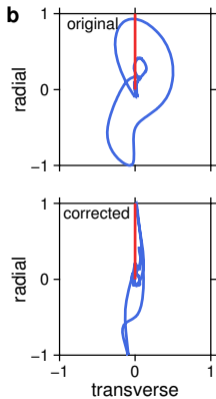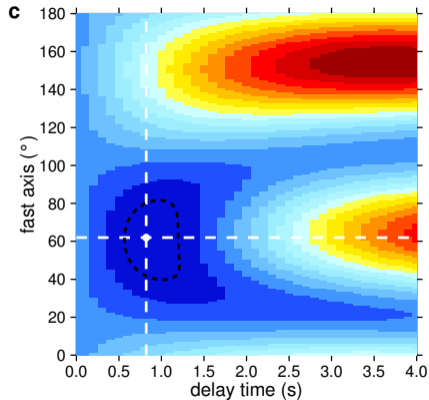

Supplement: Supplementary file 10 — Supplementary Data 8 [file 41467_2023_38296_MOESM10_ESM.zip › TP_CUQ_25-Apr-2021_22_28_01_SKS_average.pdf]

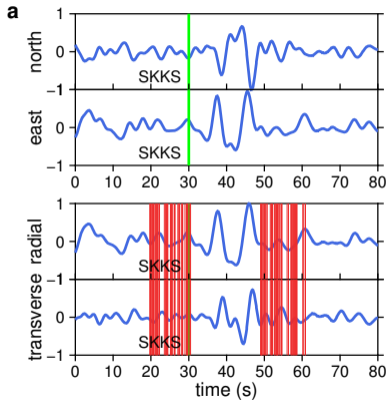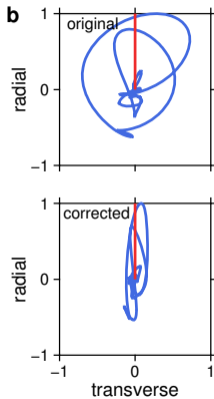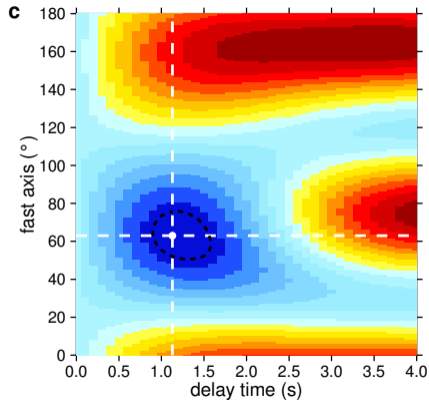

Supplement: Supplementary file 10 — Supplementary Data 8 [file 41467_2023_38296_MOESM10_ESM.zip › TP_CUQ_29-Apr-2021_06_50_29_SKKS_average.pdf]

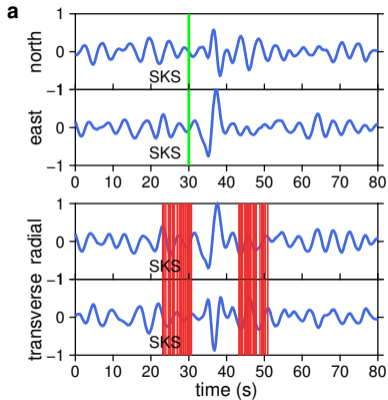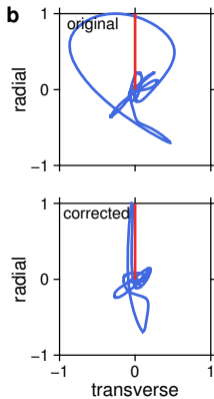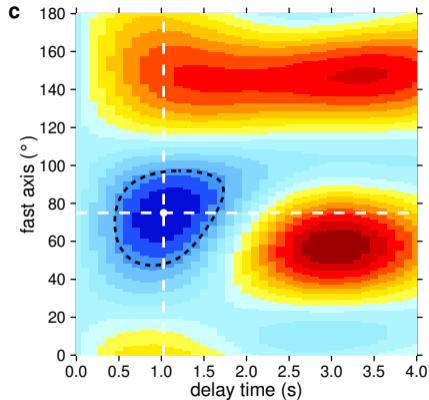

Supplement: Supplementary file 10 — Supplementary Data 8 [file 41467_2023_38296_MOESM10_ESM.zip › TP_CUQ_30-Nov-2019_19_52_40_SKS_average.pdf]

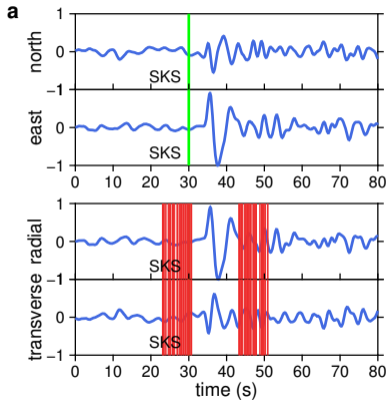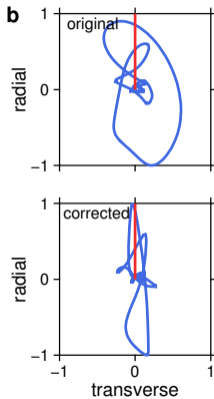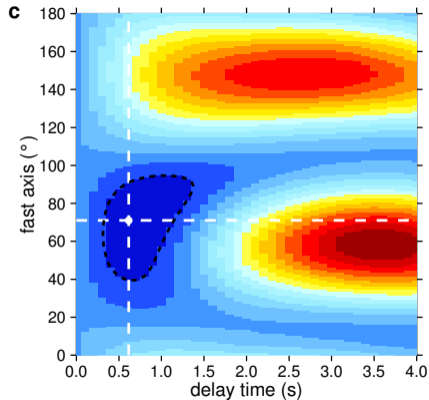

Supplement: Supplementary file 10 — Supplementary Data 8 [file 41467_2023_38296_MOESM10_ESM.zip › TP_DAX_01-Apr-2021_15_11_18_SKS_average.pdf]

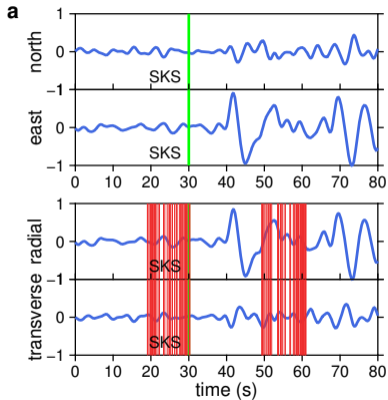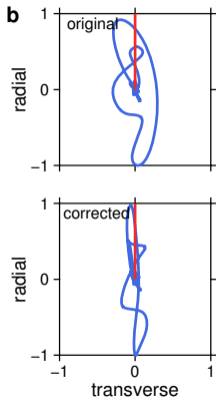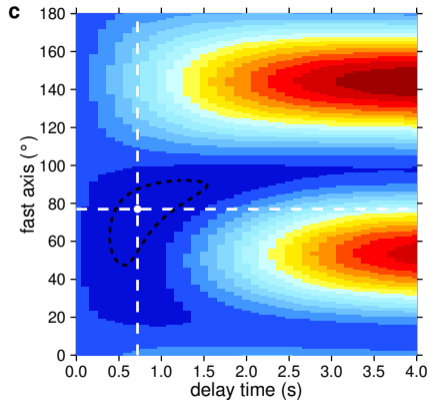

Supplement: Supplementary file 10 — Supplementary Data 8 [file 41467_2023_38296_MOESM10_ESM.zip › TP_DAX_01-Oct-2020_01_13_41_SKS_average.pdf]

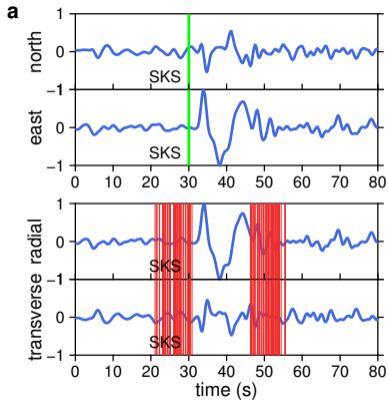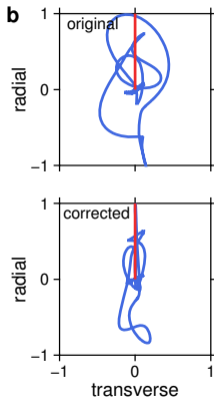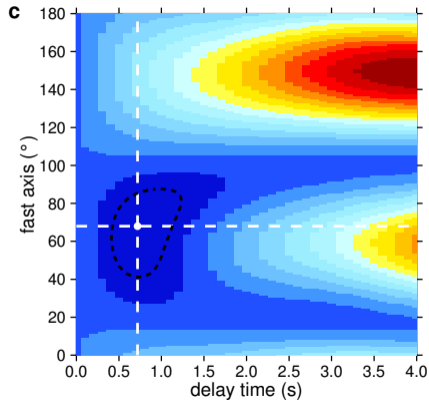

Supplement: Supplementary file 10 — Supplementary Data 8 [file 41467_2023_38296_MOESM10_ESM.zip › TP_DAX_01-Sep-2019_15_54_20_SKS_average.pdf]

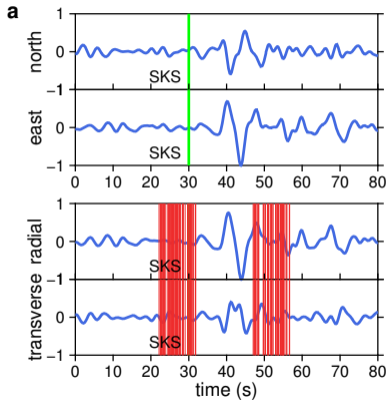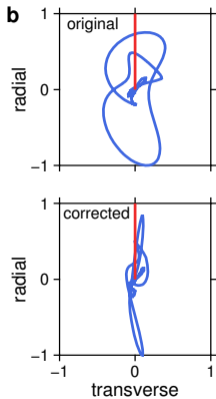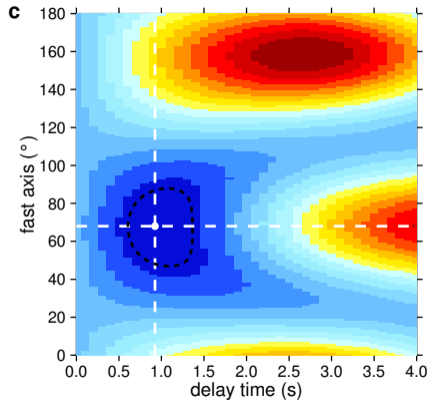

Supplement: Supplementary file 10 — Supplementary Data 8 [file 41467_2023_38296_MOESM10_ESM.zip › TP_DAX_03-Dec-2020_17_07_30_SKS_good.pdf]

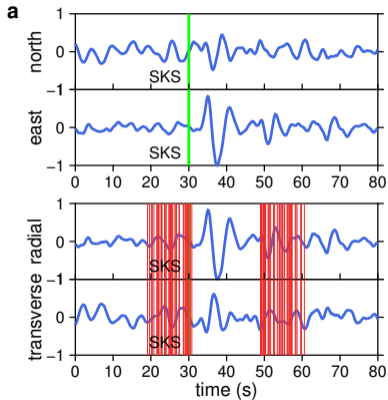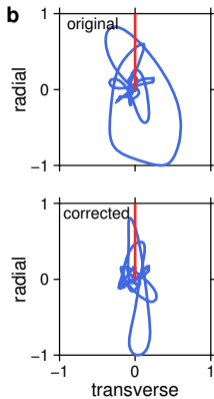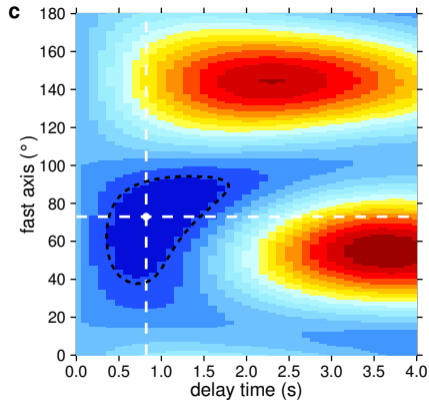

Supplement: Supplementary file 10 — Supplementary Data 8 [file 41467_2023_38296_MOESM10_ESM.zip › TP_DAX_03-Nov-2020_08_18_56_SKS_average.pdf]

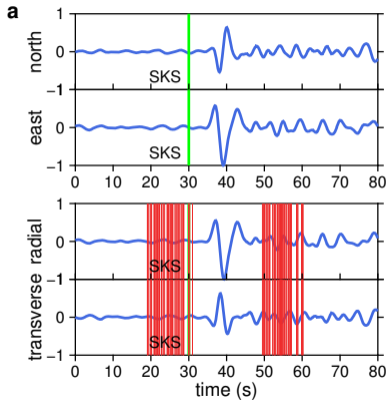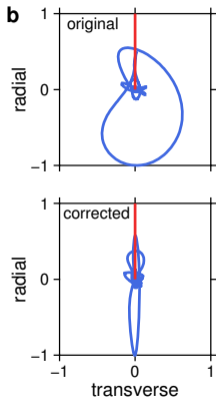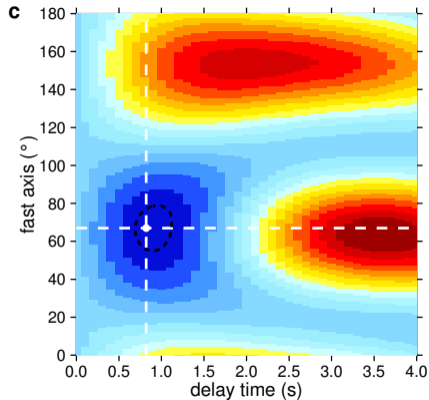

Supplement: Supplementary file 10 — Supplementary Data 8 [file 41467_2023_38296_MOESM10_ESM.zip › TP_DAX_04-Dec-2019_20_10_03_SKS_good.pdf]

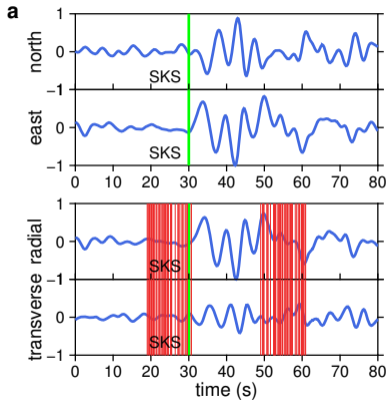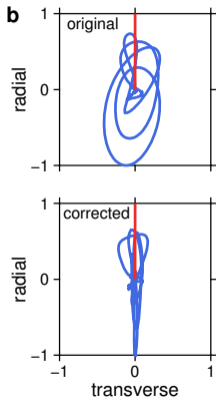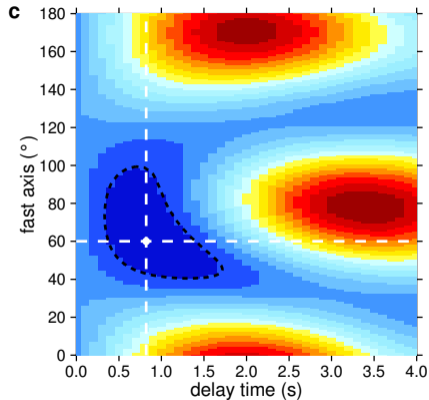

Supplement: Supplementary file 10 — Supplementary Data 8 [file 41467_2023_38296_MOESM10_ESM.zip › TP_DAX_04-Mar-2021_13_27_33_SKS_average.pdf]

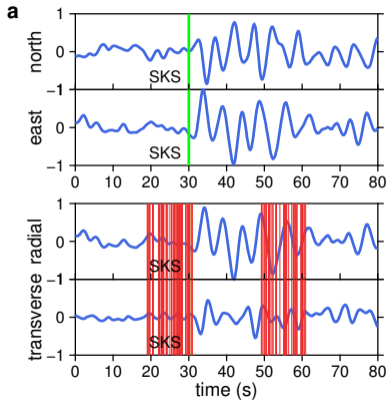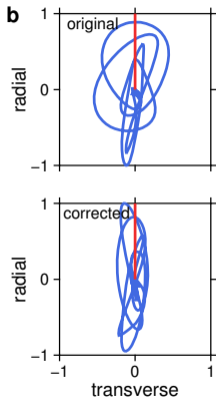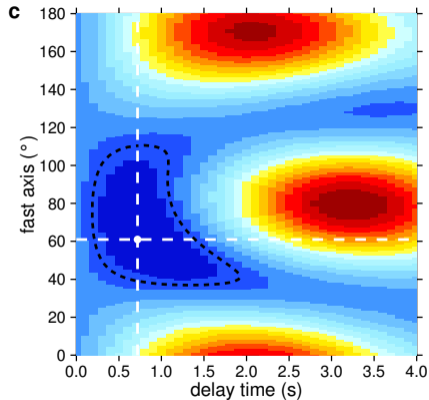

Supplement: Supplementary file 10 — Supplementary Data 8 [file 41467_2023_38296_MOESM10_ESM.zip › TP_DAX_05-Apr-2021_07_37_50_SKS_average.pdf]

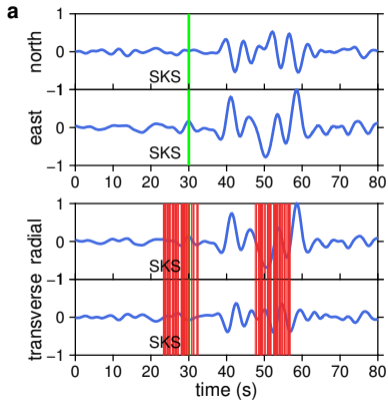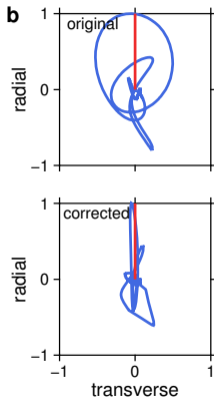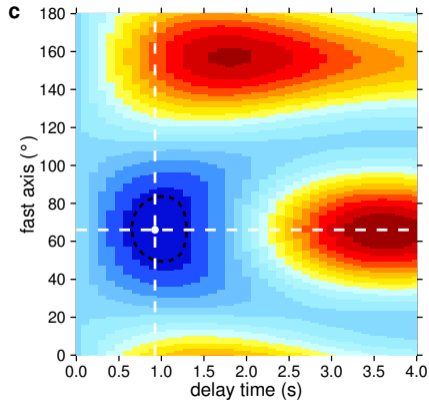

Supplement: Supplementary file 10 — Supplementary Data 8 [file 41467_2023_38296_MOESM10_ESM.zip › TP_DAX_05-Mar-2021_14_24_54_SKS_average.pdf]

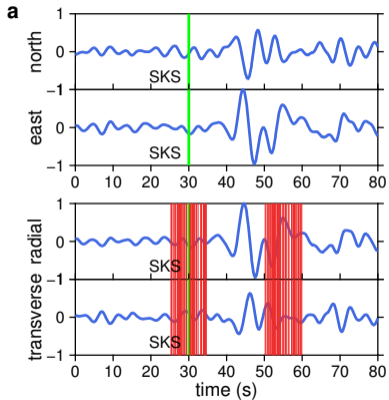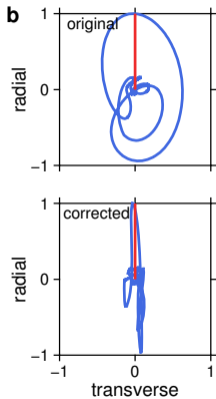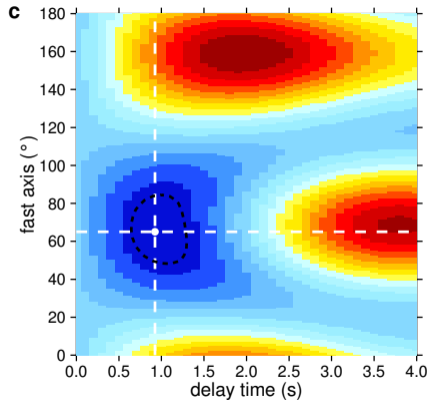

Supplement: Supplementary file 10 — Supplementary Data 8 [file 41467_2023_38296_MOESM10_ESM.zip › TP_DAX_05-Mar-2021_20_26_39_SKS_good.pdf]

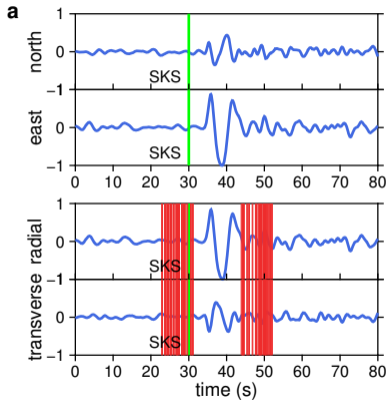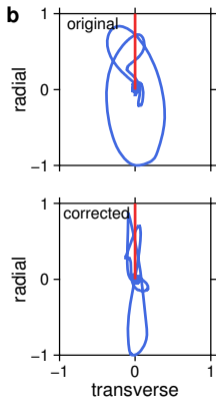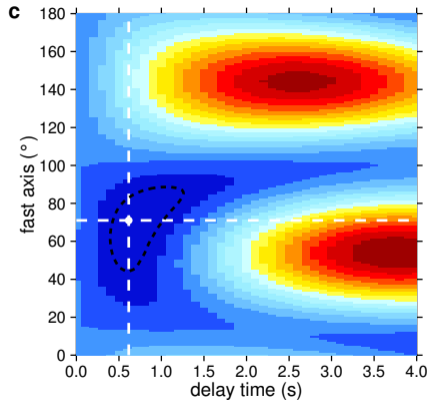

Supplement: Supplementary file 10 — Supplementary Data 8 [file 41467_2023_38296_MOESM10_ESM.zip › TP_DAX_06-Oct-2020_10_11_45_SKS_average.pdf]

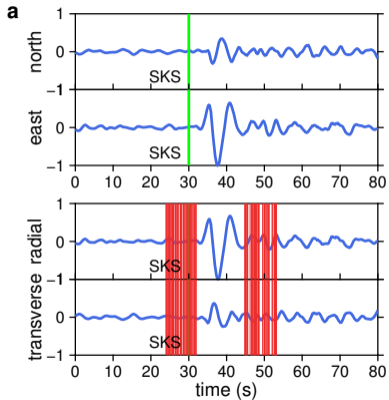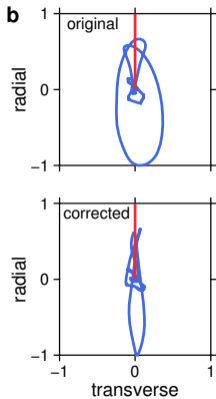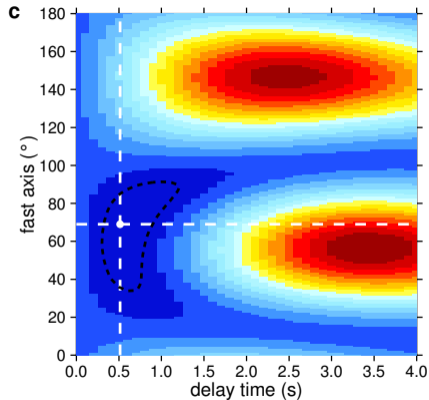

Supplement: Supplementary file 10 — Supplementary Data 8 [file 41467_2023_38296_MOESM10_ESM.zip › TP_DAX_07-May-2021_23_35_12_SKS_average.pdf]

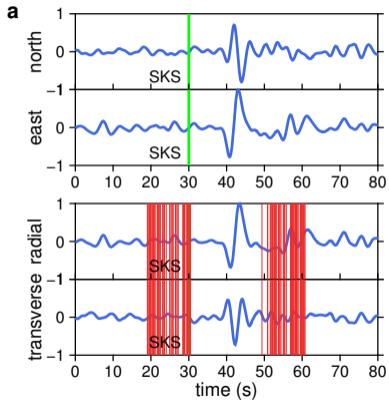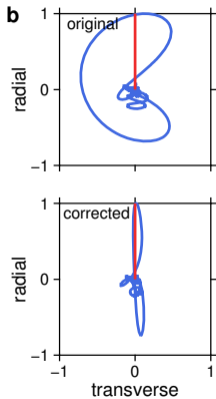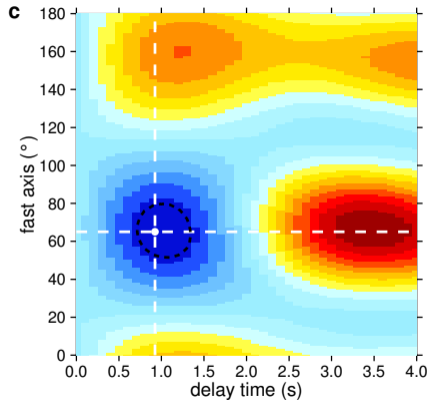

Supplement: Supplementary file 10 — Supplementary Data 8 [file 41467_2023_38296_MOESM10_ESM.zip › TP_DAX_08-Jan-2021_05_01_04_SKS_good.pdf]

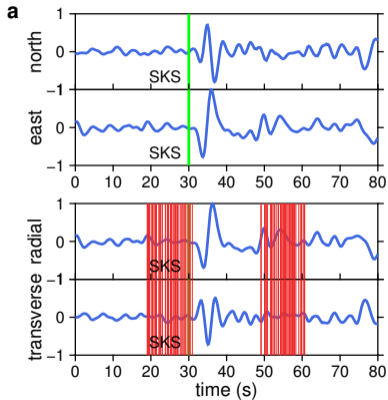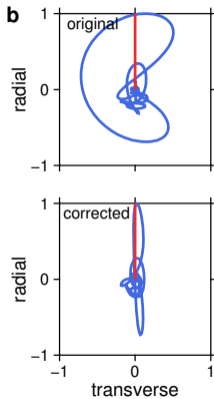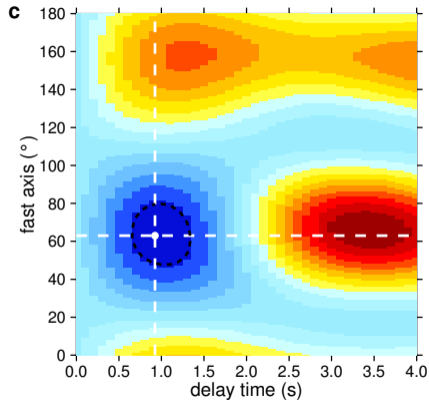

Supplement: Supplementary file 10 — Supplementary Data 8 [file 41467_2023_38296_MOESM10_ESM.zip › TP_DAX_08-Jan-2021_05_01_05_SKS_good.pdf]

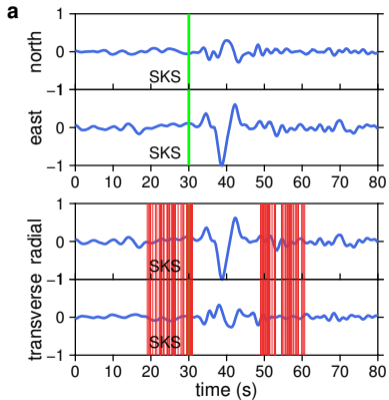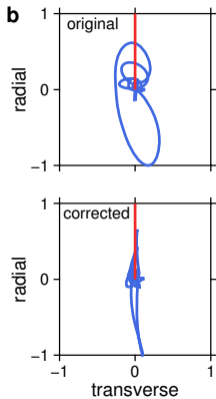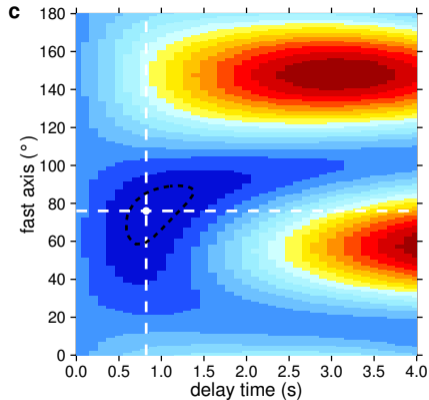

Supplement: Supplementary file 10 — Supplementary Data 8 [file 41467_2023_38296_MOESM10_ESM.zip › TP_DAX_08-Nov-2019_10_44_44_SKS_good.pdf]

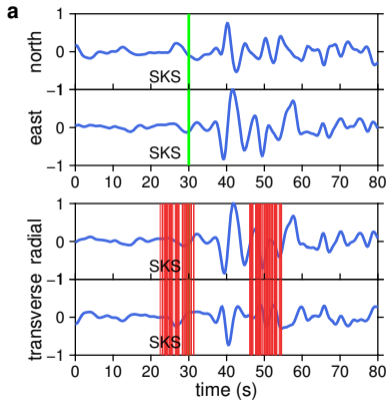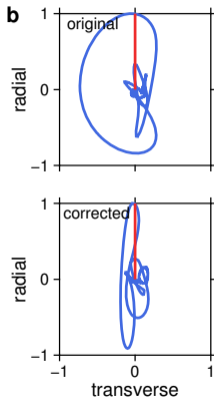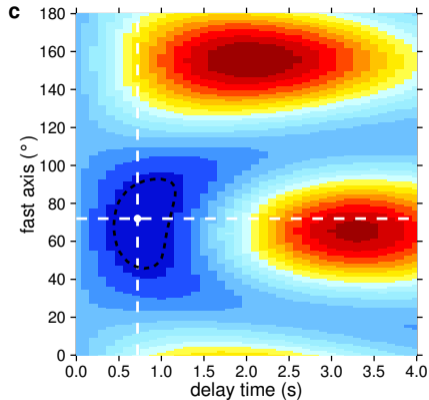

Supplement: Supplementary file 10 — Supplementary Data 8 [file 41467_2023_38296_MOESM10_ESM.zip › TP_DAX_10-Feb-2021_16_35_26_SKS_average.pdf]

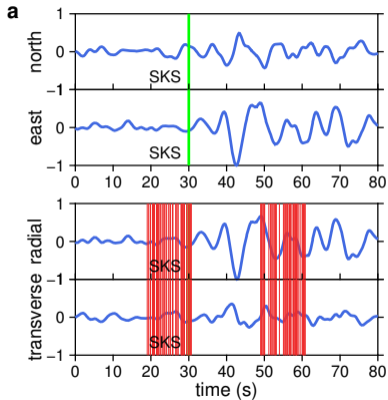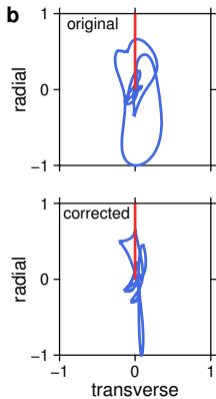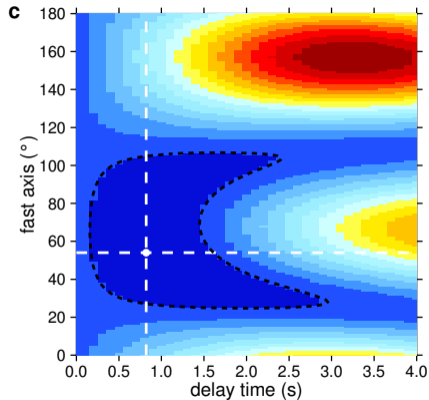

Supplement: Supplementary file 10 — Supplementary Data 8 [file 41467_2023_38296_MOESM10_ESM.zip › TP_DAX_10-Feb-2021_18_36_46_SKS_average.pdf]

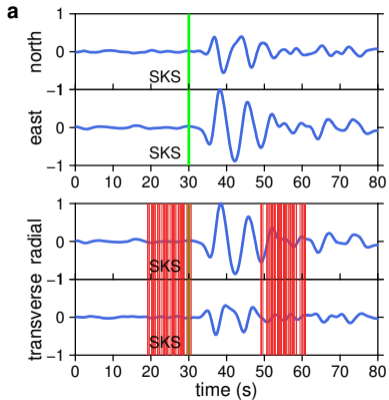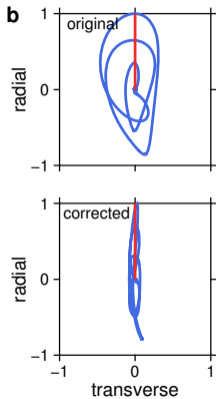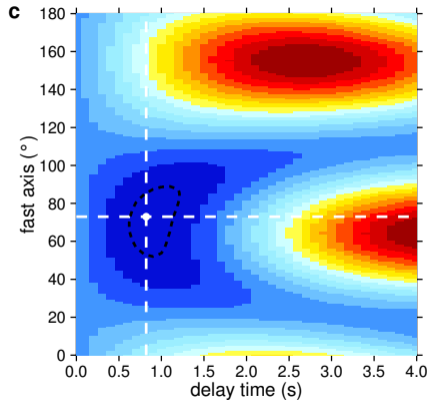

Supplement: Supplementary file 10 — Supplementary Data 8 [file 41467_2023_38296_MOESM10_ESM.zip › TP_DAX_10-Feb-2021_21_23_58_SKS_good.pdf]

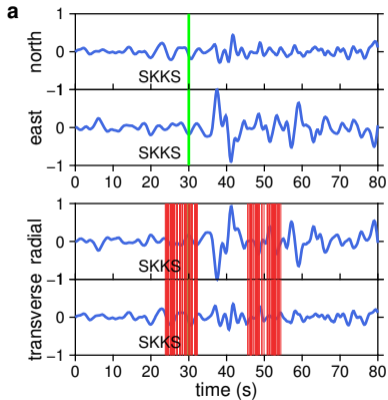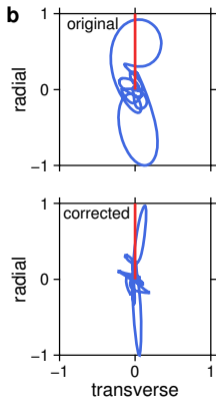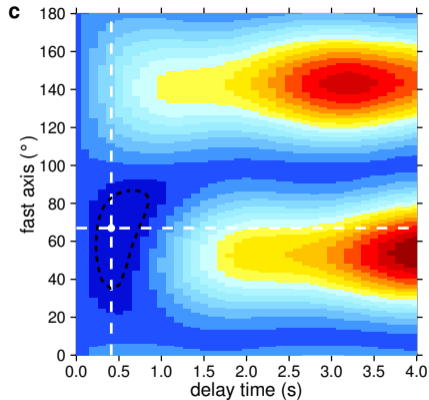

Supplement: Supplementary file 10 — Supplementary Data 8 [file 41467_2023_38296_MOESM10_ESM.zip › TP_DAX_10-Jan-2021_03_54_14_SKKS_average.pdf]

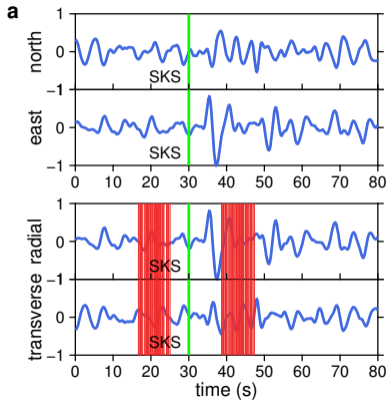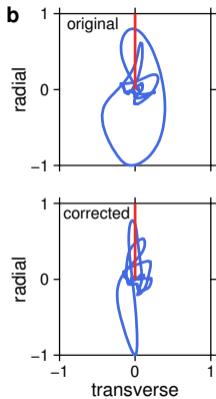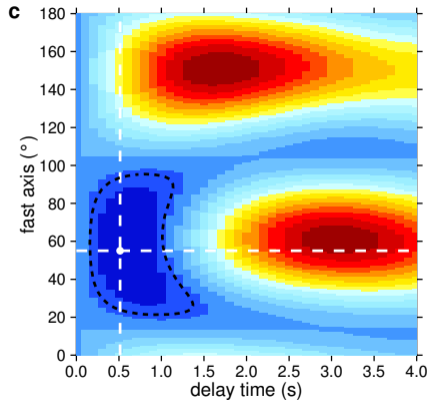

Supplement: Supplementary file 10 — Supplementary Data 8 [file 41467_2023_38296_MOESM10_ESM.zip › TP_DAX_10-Mar-2021_20_12_39_SKS_average.pdf]

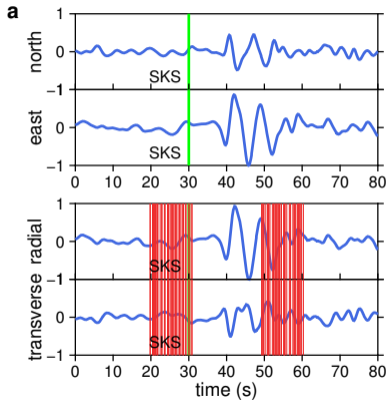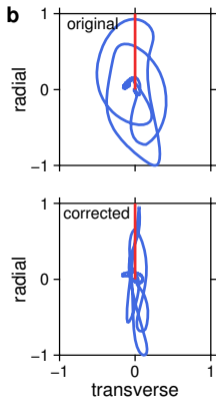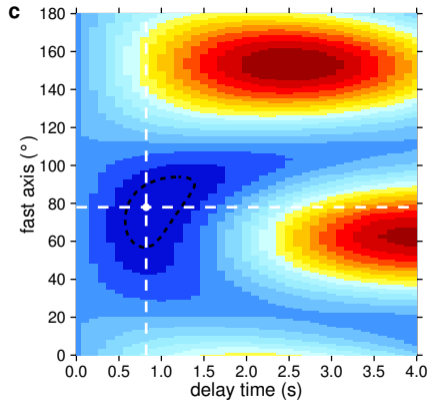

Supplement: Supplementary file 10 — Supplementary Data 8 [file 41467_2023_38296_MOESM10_ESM.zip › TP_DAX_11-Feb-2021_00_14_52_SKS_average.pdf]

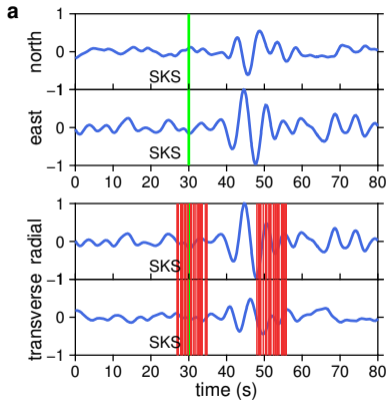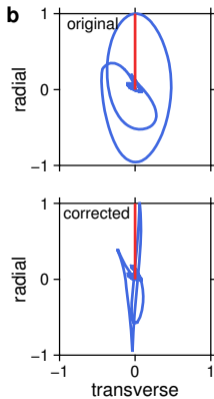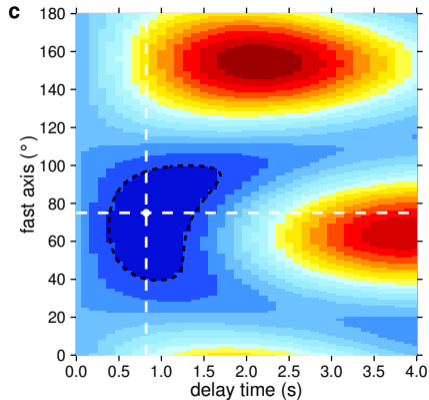

Supplement: Supplementary file 10 — Supplementary Data 8 [file 41467_2023_38296_MOESM10_ESM.zip › TP_DAX_11-Feb-2021_08_39_14_SKS_good.pdf]

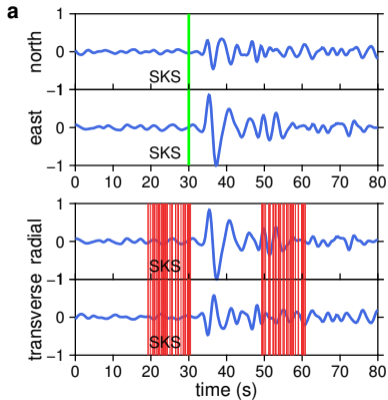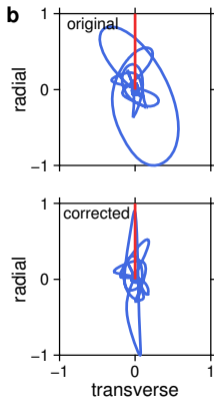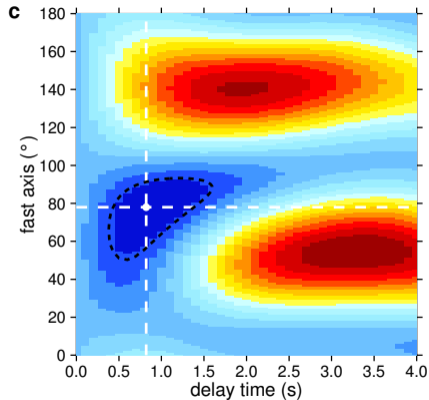

Supplement: Supplementary file 10 — Supplementary Data 8 [file 41467_2023_38296_MOESM10_ESM.zip › TP_DAX_11-Nov-2020_00_48_43_SKS_average.pdf]

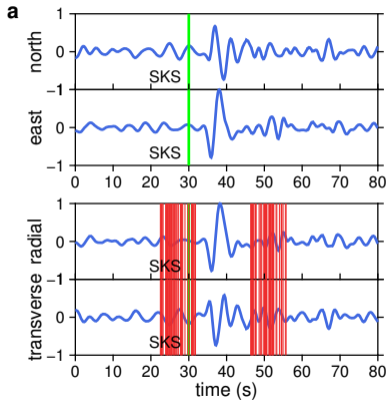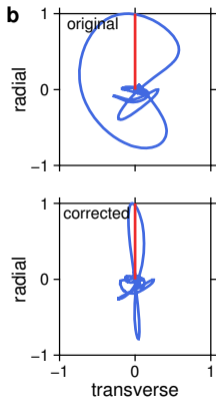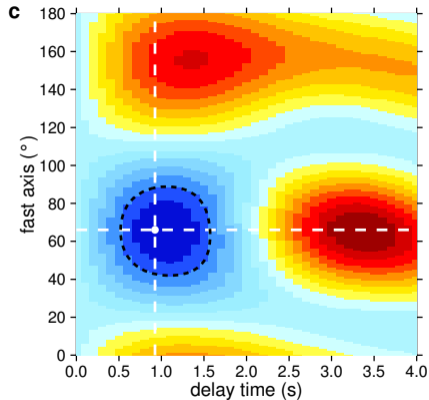

Supplement: Supplementary file 10 — Supplementary Data 8 [file 41467_2023_38296_MOESM10_ESM.zip › TP_DAX_12-Mar-2021_00_03_21_SKS_good.pdf]

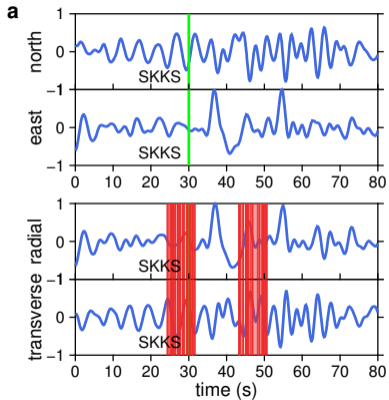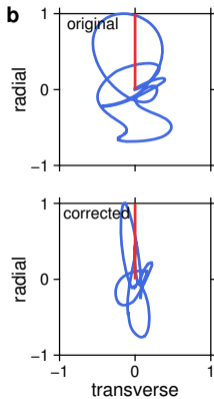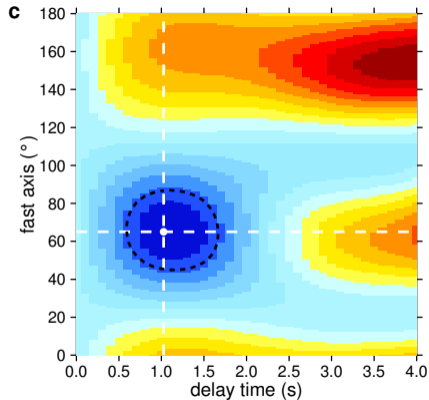

Supplement: Supplementary file 10 — Supplementary Data 8 [file 41467_2023_38296_MOESM10_ESM.zip › TP_DAX_14-Apr-2020_01_06_47_SKKS_average.pdf]

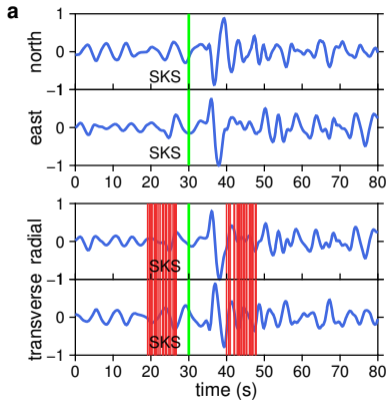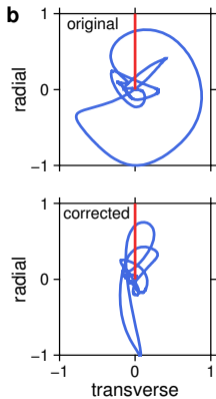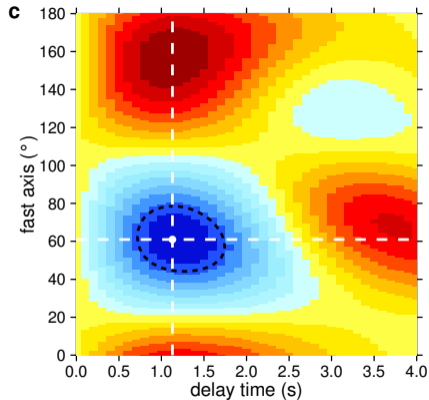

Supplement: Supplementary file 10 — Supplementary Data 8 [file 41467_2023_38296_MOESM10_ESM.zip › TP_DAX_14-Dec-2020_01_57_10_SKS_average.pdf]

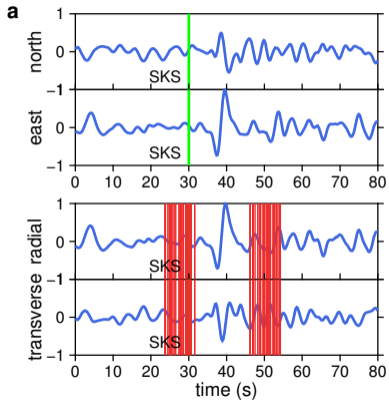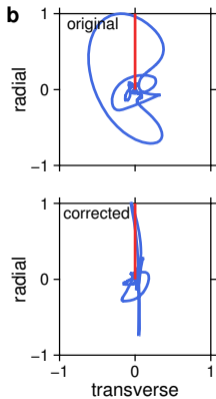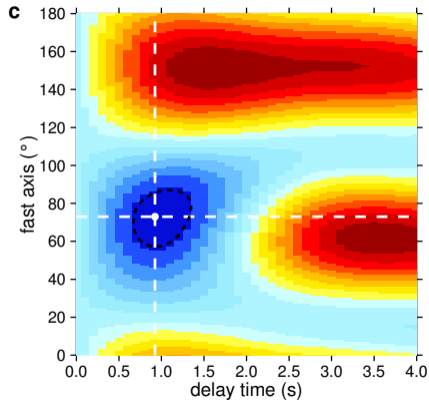

Supplement: Supplementary file 10 — Supplementary Data 8 [file 41467_2023_38296_MOESM10_ESM.zip › TP_DAX_16-Sep-2020_08_44_29_SKS_good.pdf]

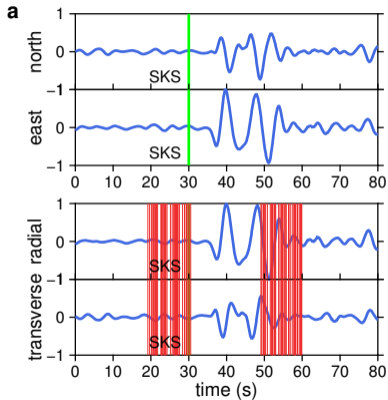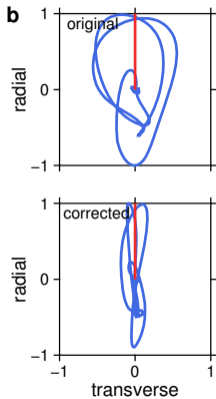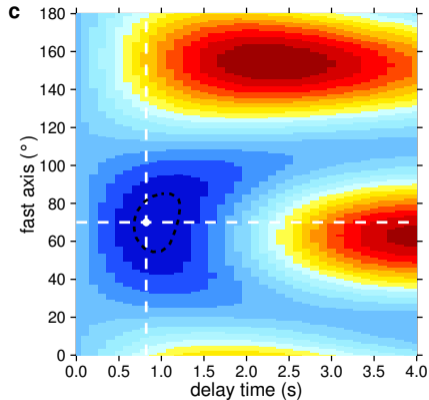

Supplement: Supplementary file 10 — Supplementary Data 8 [file 41467_2023_38296_MOESM10_ESM.zip › TP_DAX_17-Feb-2021_22_49_39_SKS_good.pdf]

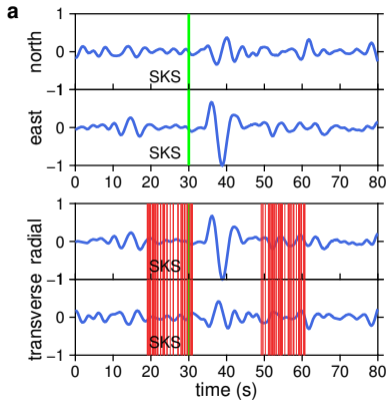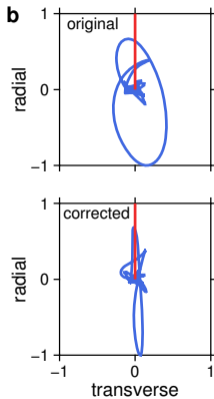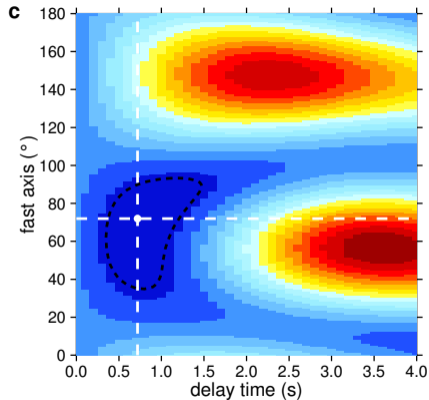

Supplement: Supplementary file 10 — Supplementary Data 8 [file 41467_2023_38296_MOESM10_ESM.zip › TP_DAX_17-Nov-2019_12_13_27_SKS_average.pdf]

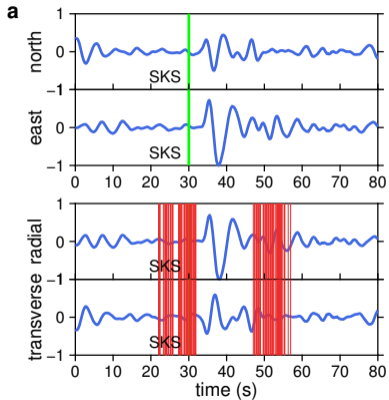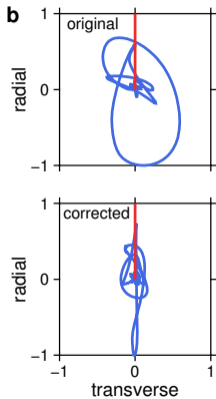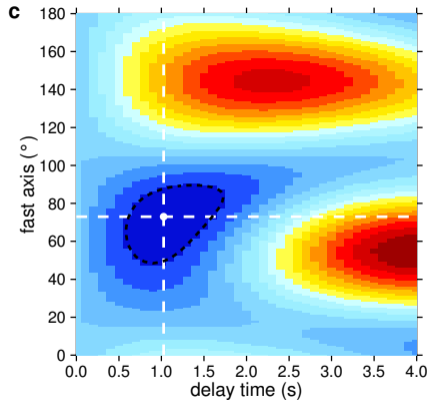

Supplement: Supplementary file 10 — Supplementary Data 8 [file 41467_2023_38296_MOESM10_ESM.zip › TP_DAX_21-Jul-2020_20_56_26_SKS_good.pdf]

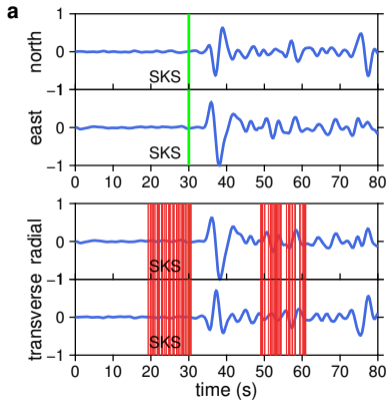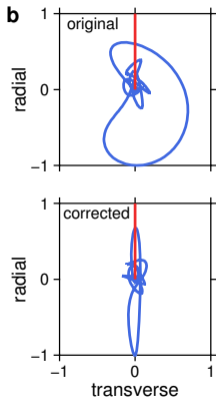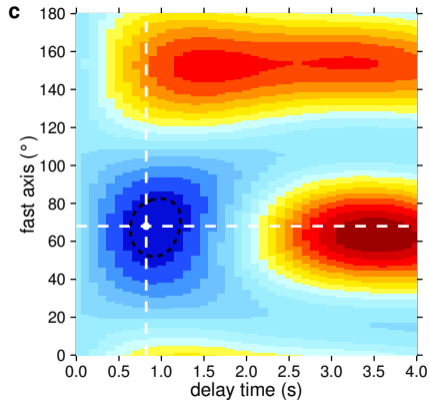

Supplement: Supplementary file 10 — Supplementary Data 8 [file 41467_2023_38296_MOESM10_ESM.zip › TP_DAX_21-Oct-2019_02_52_29_SKS_good.pdf]

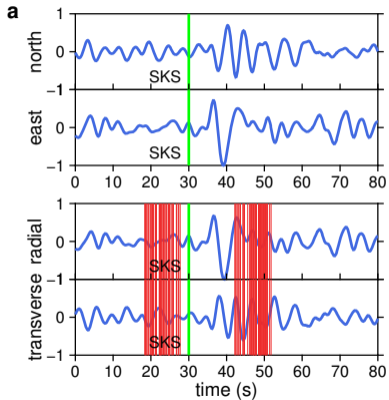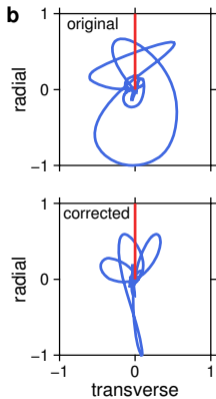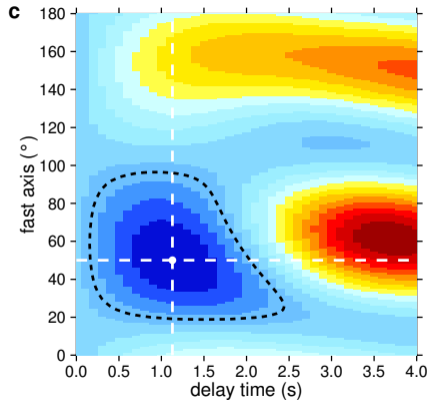

Supplement: Supplementary file 10 — Supplementary Data 8 [file 41467_2023_38296_MOESM10_ESM.zip › TP_DAX_22-Apr-2020_22_31_25_SKS_average.pdf]

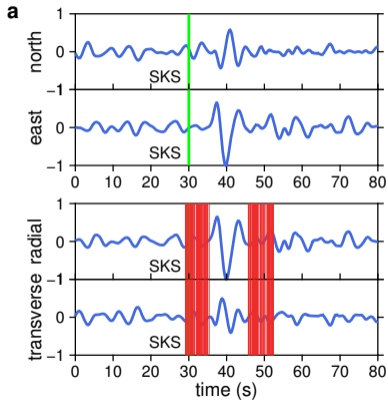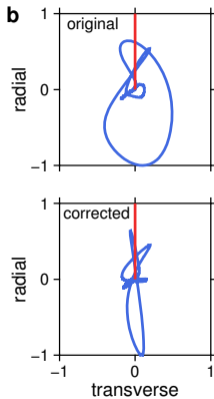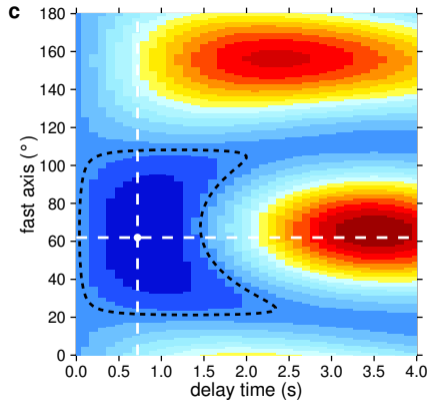

Supplement: Supplementary file 10 — Supplementary Data 8 [file 41467_2023_38296_MOESM10_ESM.zip › TP_DAX_23-Mar-2020_20_33_39_SKS_average.pdf]

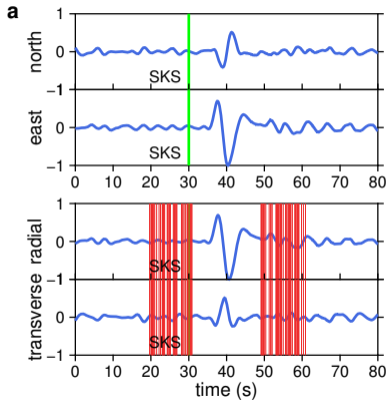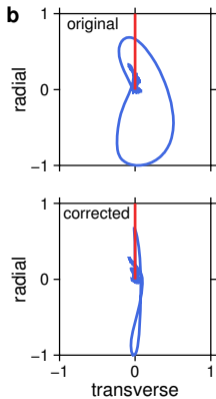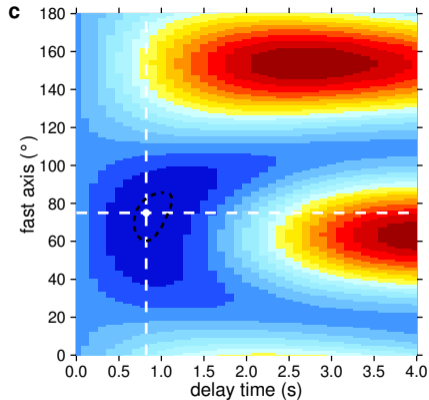

Supplement: Supplementary file 10 — Supplementary Data 8 [file 41467_2023_38296_MOESM10_ESM.zip › TP_DAX_23-Oct-2020_07_04_31_SKS_good.pdf]

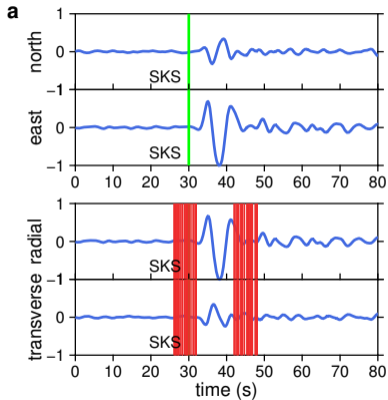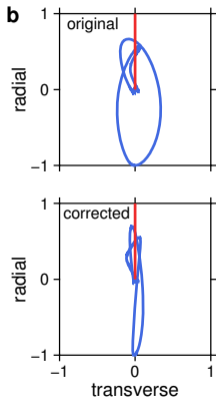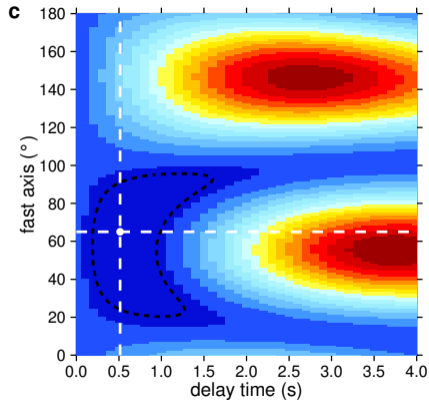

Supplement: Supplementary file 10 — Supplementary Data 8 [file 41467_2023_38296_MOESM10_ESM.zip › TP_DAX_24-Apr-2021_00_23_35_SKS_good.pdf]

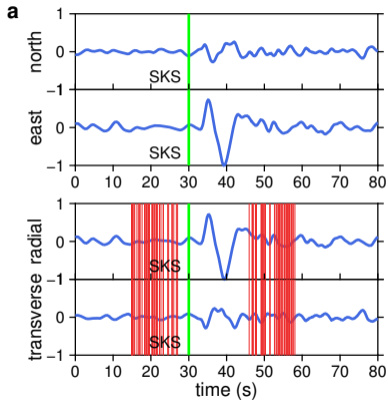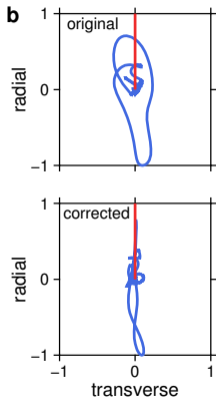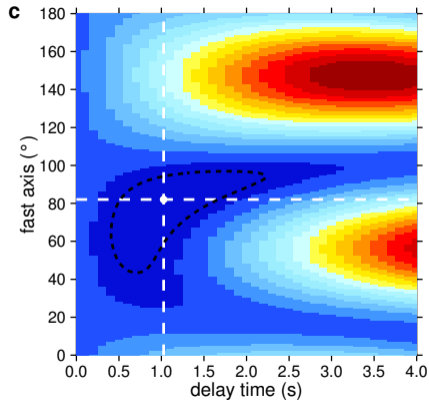

Supplement: Supplementary file 10 — Supplementary Data 8 [file 41467_2023_38296_MOESM10_ESM.zip › TP_DAX_25-Apr-2021_22_28_01_SKS_average.pdf]

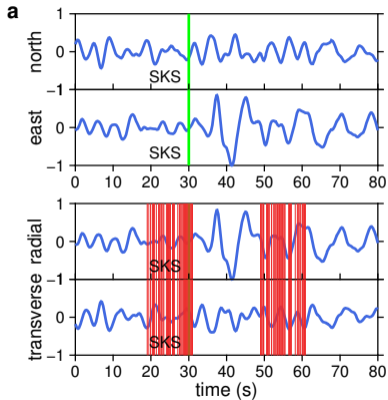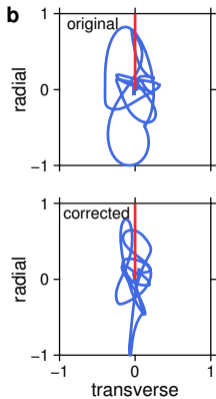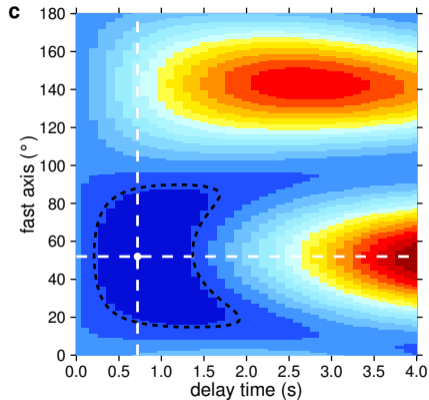

Supplement: Supplementary file 10 — Supplementary Data 8 [file 41467_2023_38296_MOESM10_ESM.zip › TP_DAX_25-Oct-2020_11_47_38_SKS_average.pdf]

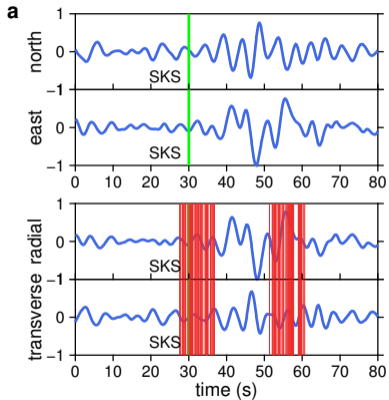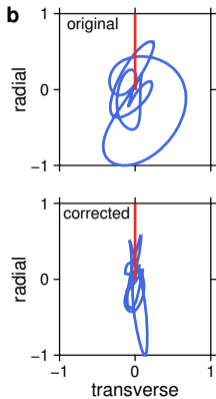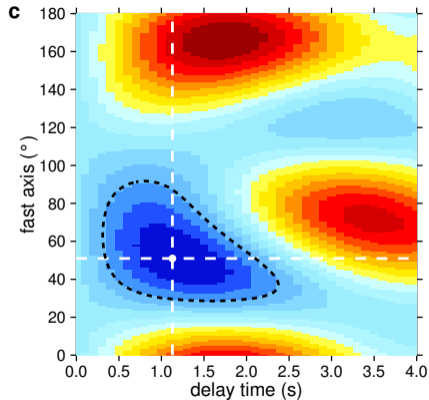

Supplement: Supplementary file 10 — Supplementary Data 8 [file 41467_2023_38296_MOESM10_ESM.zip › TP_DAX_26-Apr-2021_21_07_25_SKS_average.pdf]

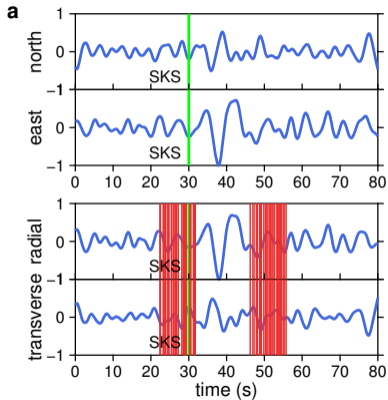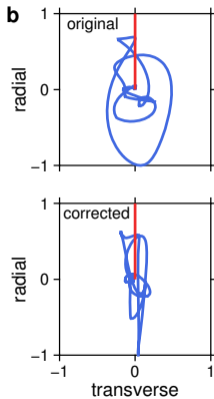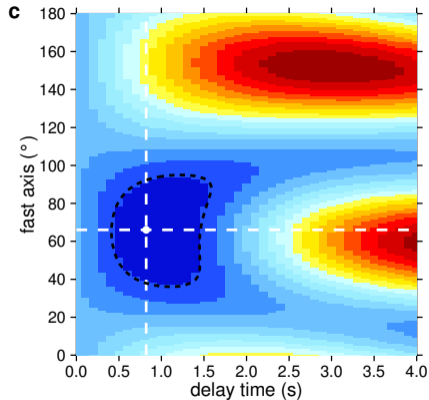

Supplement: Supplementary file 10 — Supplementary Data 8 [file 41467_2023_38296_MOESM10_ESM.zip › TP_DAX_26-Dec-2019_02_21_28_SKS_average.pdf]

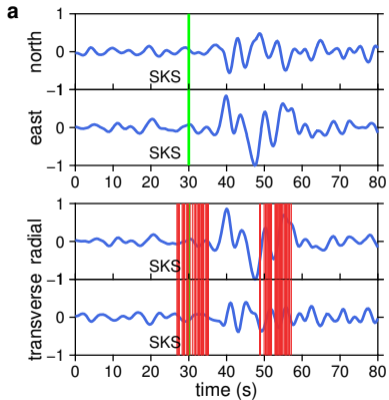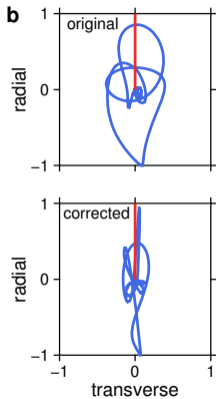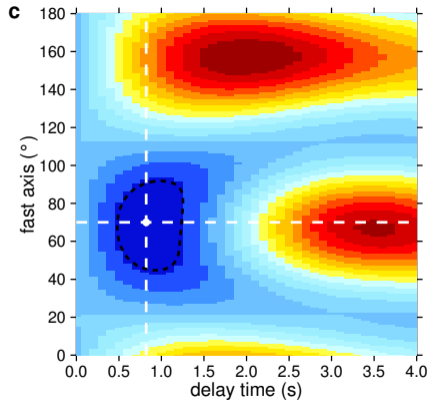

Supplement: Supplementary file 10 — Supplementary Data 8 [file 41467_2023_38296_MOESM10_ESM.zip › TP_DAX_27-Apr-2021_16_33_31_SKS_average.pdf]

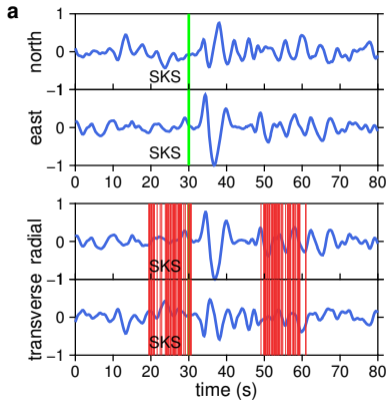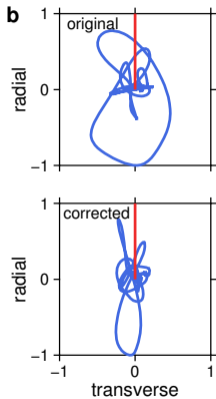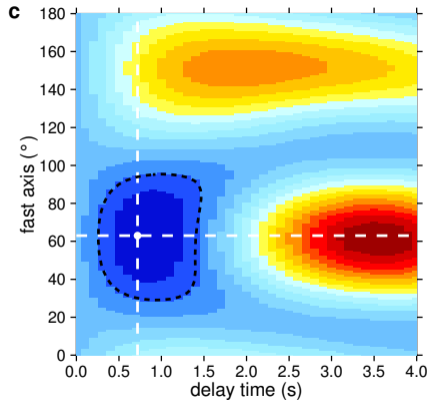

Supplement: Supplementary file 10 — Supplementary Data 8 [file 41467_2023_38296_MOESM10_ESM.zip › TP_DAX_27-Nov-2019_10_00_39_SKS_average.pdf]

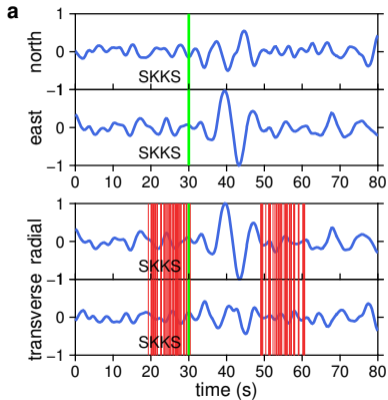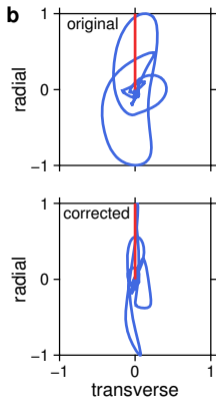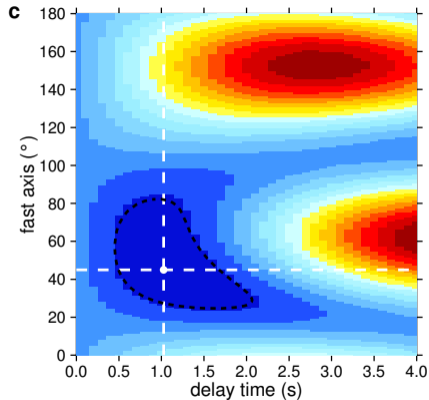

Supplement: Supplementary file 10 — Supplementary Data 8 [file 41467_2023_38296_MOESM10_ESM.zip › TP_DAX_28-May-2020_07_18_42_SKKS_average.pdf]

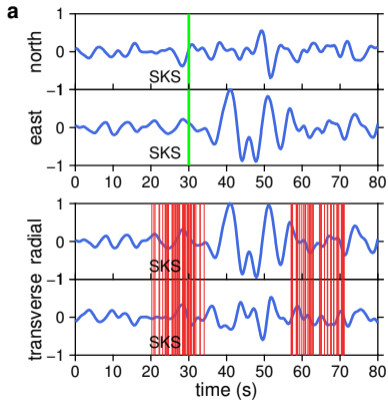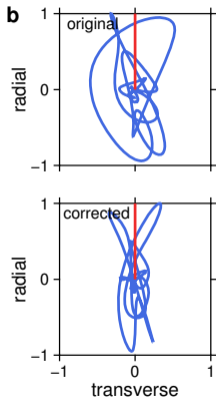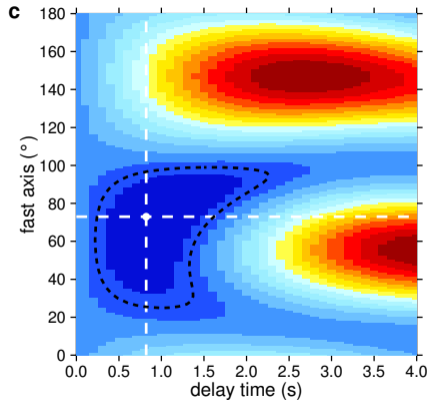

Supplement: Supplementary file 10 — Supplementary Data 8 [file 41467_2023_38296_MOESM10_ESM.zip › TP_DAX_28-May-2020_07_18_42_SKS_average.pdf]

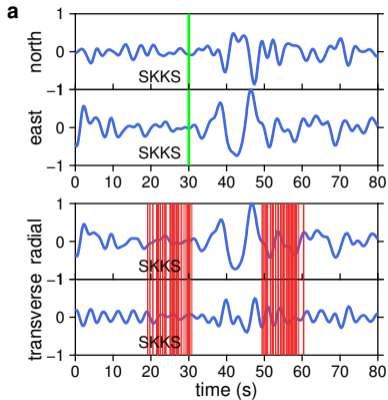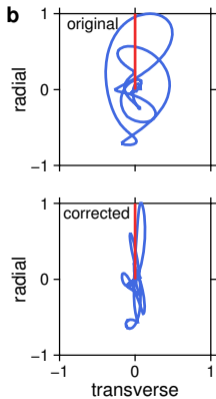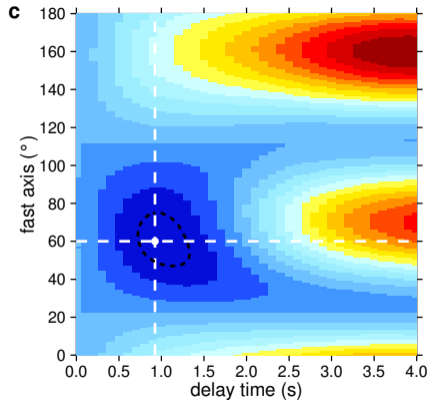

Supplement: Supplementary file 10 — Supplementary Data 8 [file 41467_2023_38296_MOESM10_ESM.zip › TP_DAX_29-Apr-2021_06_50_29_SKKS_good.pdf]

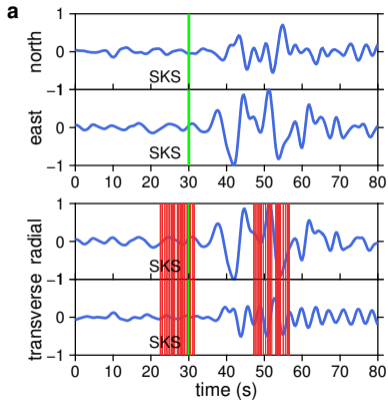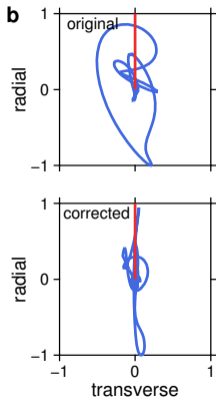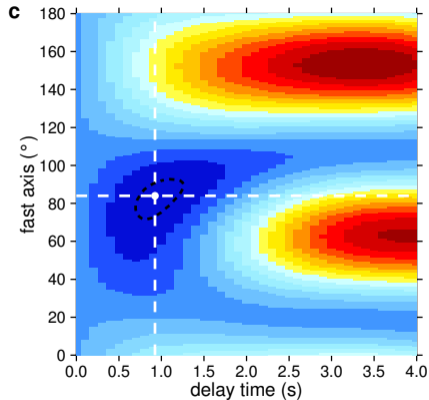

Supplement: Supplementary file 10 — Supplementary Data 8 [file 41467_2023_38296_MOESM10_ESM.zip › TP_DAX_29-Apr-2021_06_50_29_SKS_average.pdf]

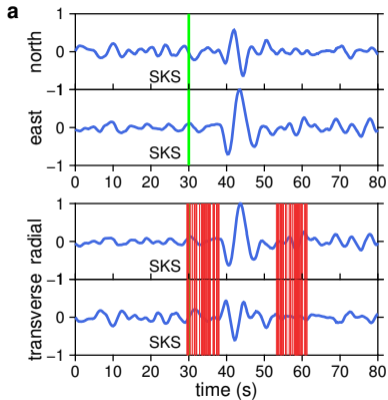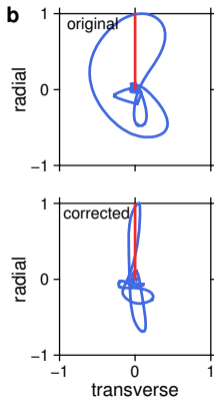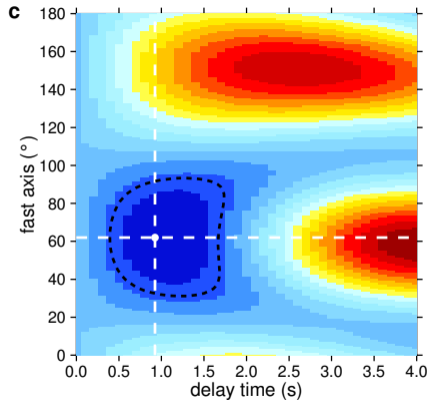

Supplement: Supplementary file 10 — Supplementary Data 8 [file 41467_2023_38296_MOESM10_ESM.zip › TP_DAX_31-May-2020_23_25_43_SKS_average.pdf]

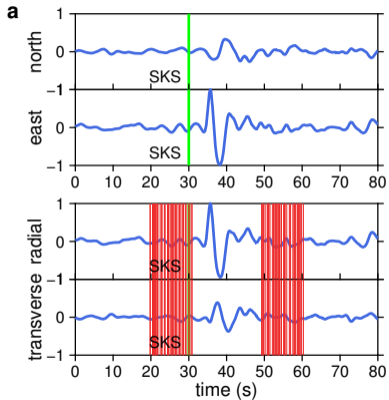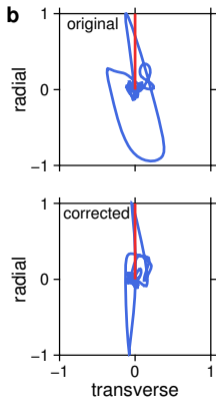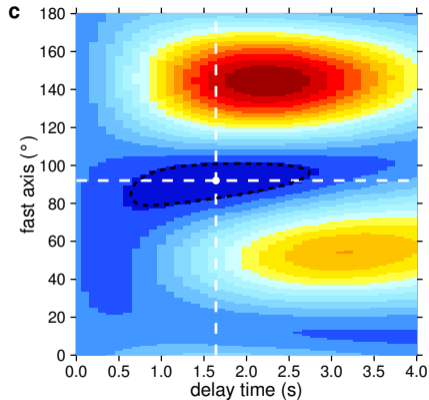

Supplement: Supplementary file 10 — Supplementary Data 8 [file 41467_2023_38296_MOESM10_ESM.zip › TP_DOC_01-Apr-2021_15_11_18_SKS_average.pdf]

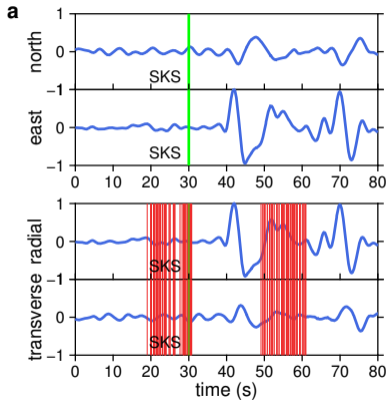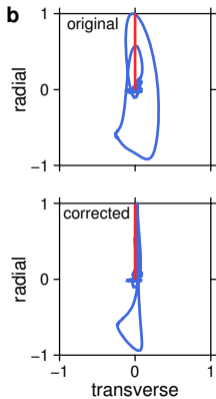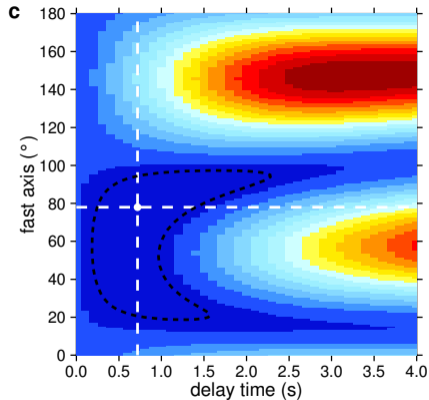

Supplement: Supplementary file 10 — Supplementary Data 8 [file 41467_2023_38296_MOESM10_ESM.zip › TP_DOC_01-Oct-2020_01_13_41_SKS_average.pdf]

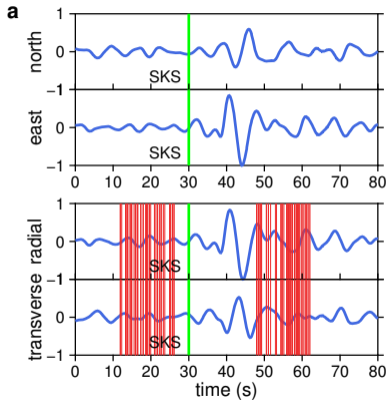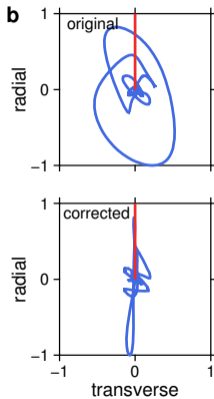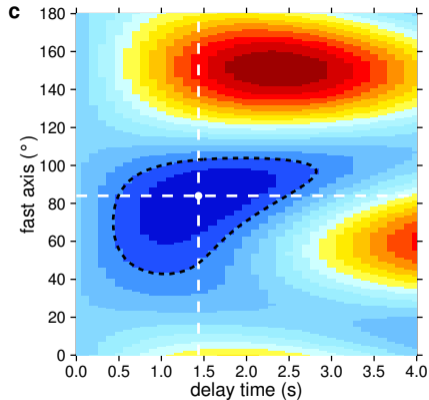

Supplement: Supplementary file 10 — Supplementary Data 8 [file 41467_2023_38296_MOESM10_ESM.zip › TP_DOC_03-Dec-2020_17_07_30_SKS_average.pdf]

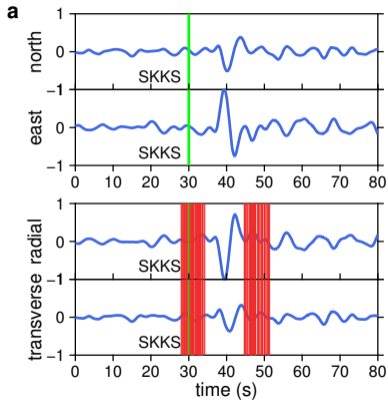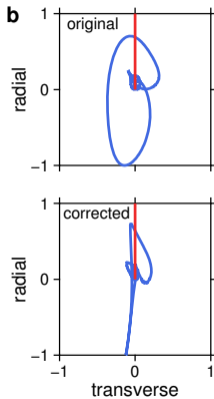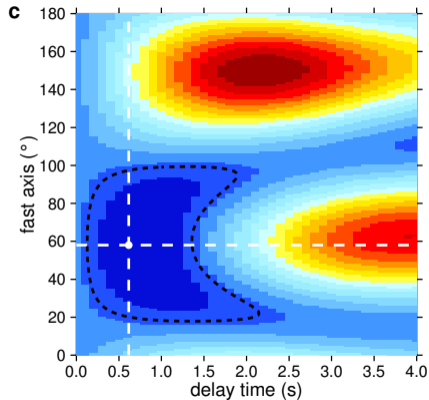

Supplement: Supplementary file 10 — Supplementary Data 8 [file 41467_2023_38296_MOESM10_ESM.zip › TP_DOC_03-Jun-2020_07_35_36_SKKS_average.pdf]

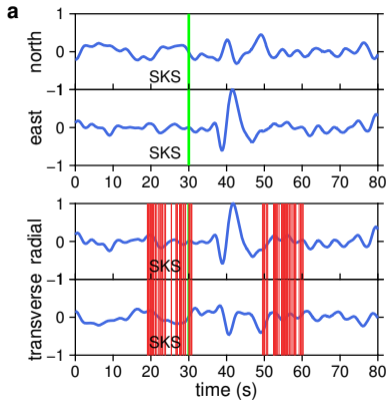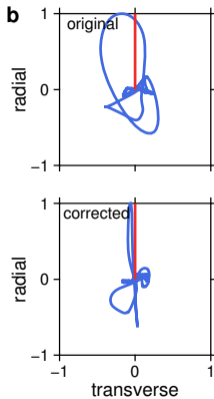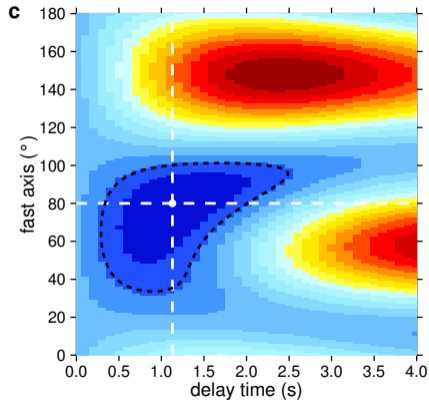

Supplement: Supplementary file 10 — Supplementary Data 8 [file 41467_2023_38296_MOESM10_ESM.zip › TP_DOC_03-Jun-2020_09_31_39_SKS_average.pdf]

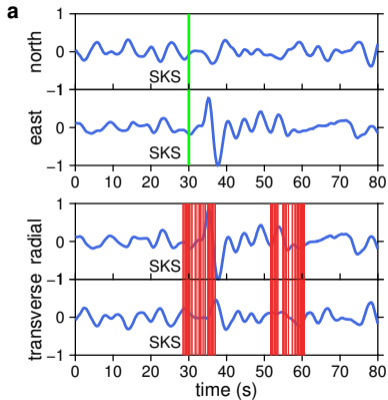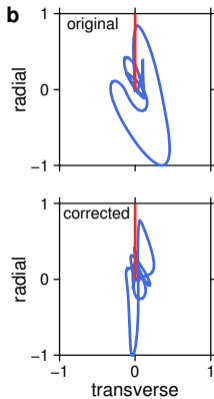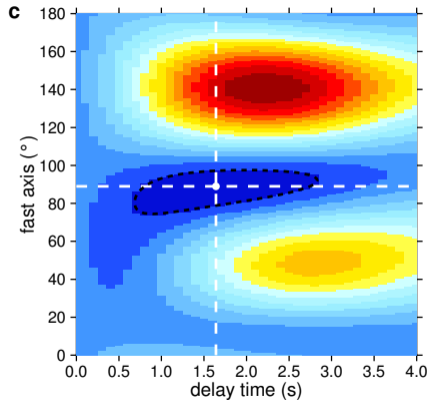

Supplement: Supplementary file 10 — Supplementary Data 8 [file 41467_2023_38296_MOESM10_ESM.zip › TP_DOC_03-Nov-2020_08_18_56_SKS_average.pdf]

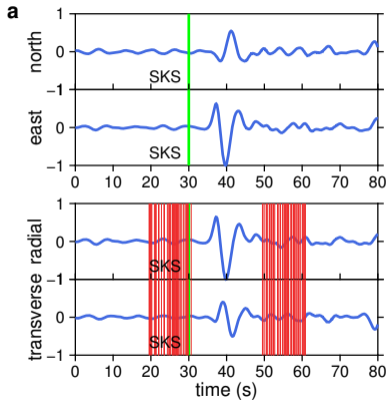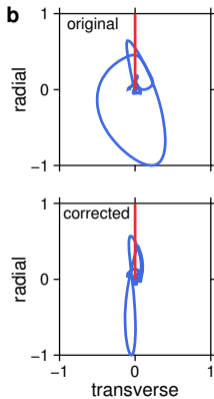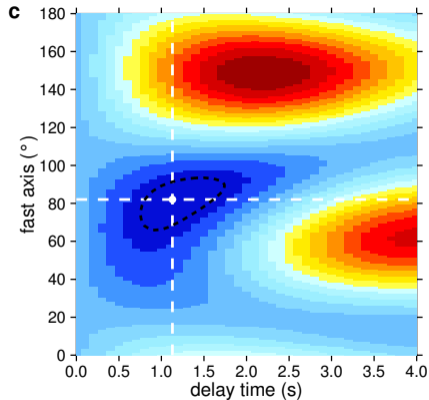

Supplement: Supplementary file 10 — Supplementary Data 8 [file 41467_2023_38296_MOESM10_ESM.zip › TP_DOC_04-Dec-2019_20_10_03_SKS_good.pdf]

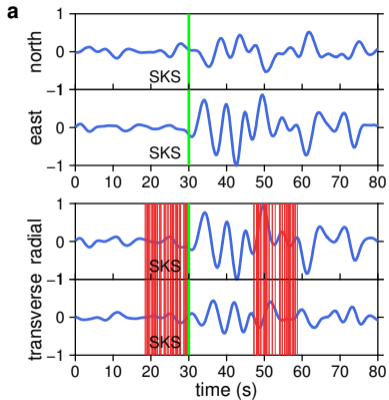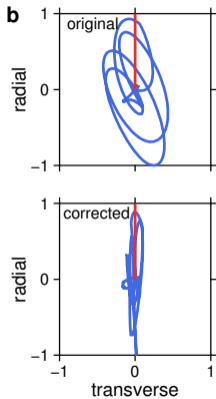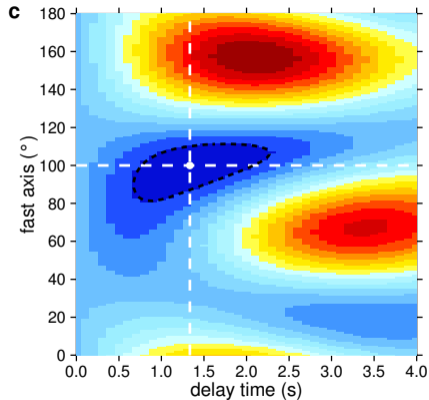

Supplement: Supplementary file 10 — Supplementary Data 8 [file 41467_2023_38296_MOESM10_ESM.zip › TP_DOC_04-Mar-2021_13_27_33_SKS_good.pdf]

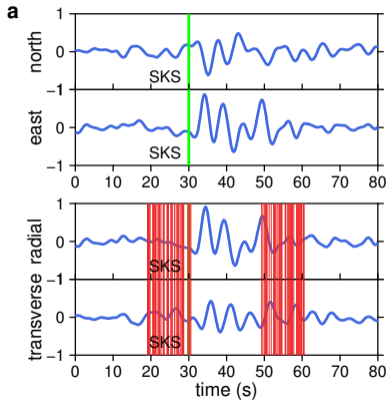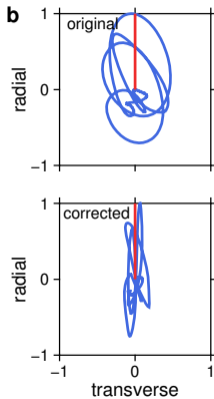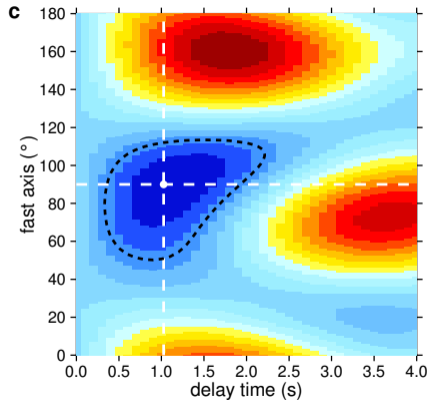

Supplement: Supplementary file 10 — Supplementary Data 8 [file 41467_2023_38296_MOESM10_ESM.zip › TP_DOC_05-Apr-2021_07_37_50_SKS_average.pdf]

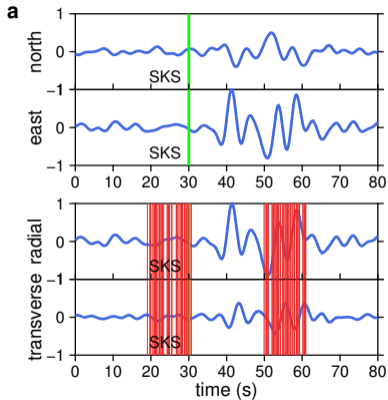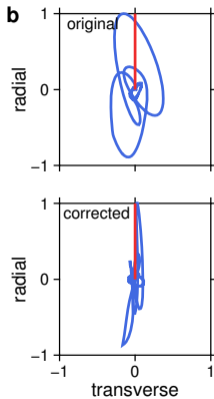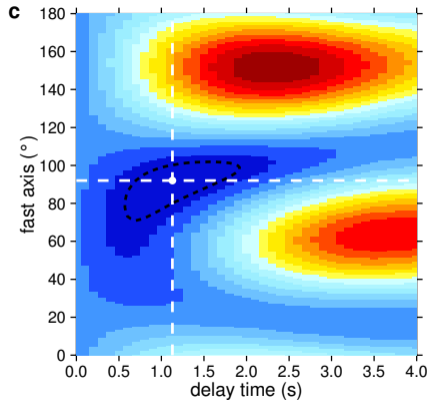

Supplement: Supplementary file 10 — Supplementary Data 8 [file 41467_2023_38296_MOESM10_ESM.zip › TP_DOC_05-Mar-2021_14_24_54_SKS_average.pdf]

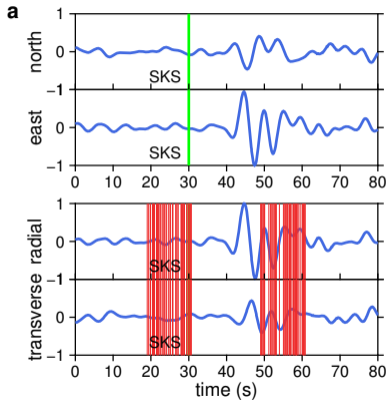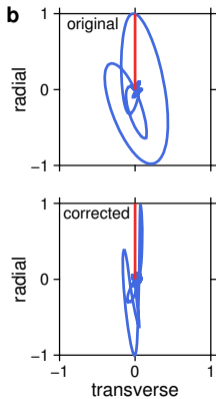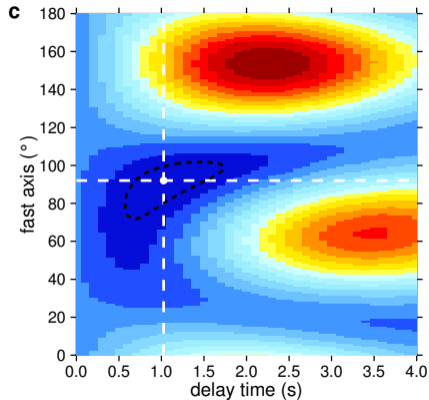

Supplement: Supplementary file 10 — Supplementary Data 8 [file 41467_2023_38296_MOESM10_ESM.zip › TP_DOC_05-Mar-2021_20_26_39_SKS_average.pdf]

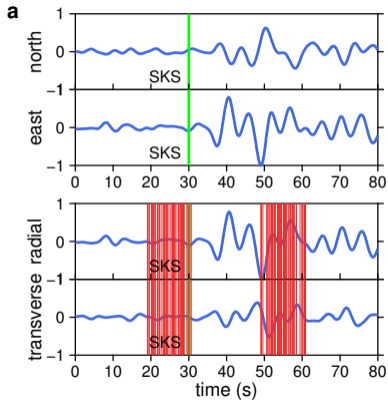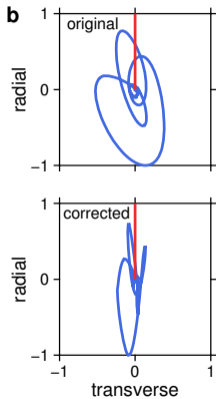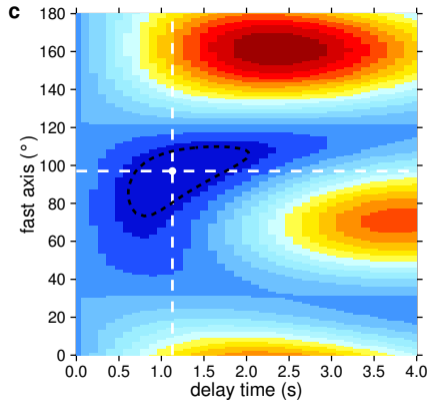

Supplement: Supplementary file 10 — Supplementary Data 8 [file 41467_2023_38296_MOESM10_ESM.zip › TP_DOC_06-Mar-2021_00_16_14_SKS_average.pdf]

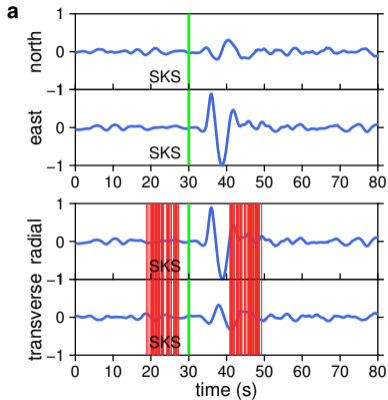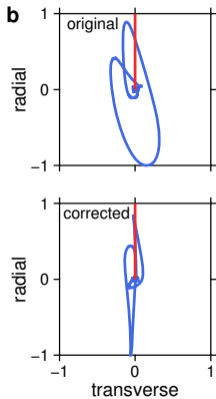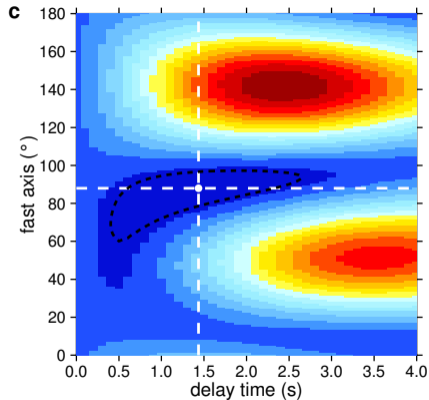

Supplement: Supplementary file 10 — Supplementary Data 8 [file 41467_2023_38296_MOESM10_ESM.zip › TP_DOC_06-Oct-2020_10_11_45_SKS_average.pdf]

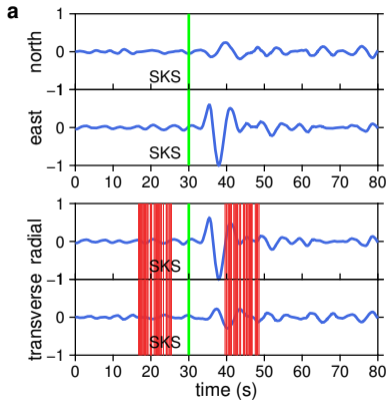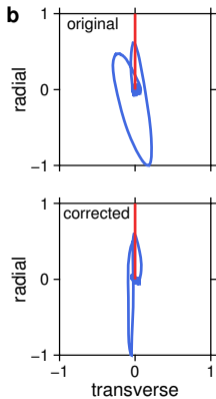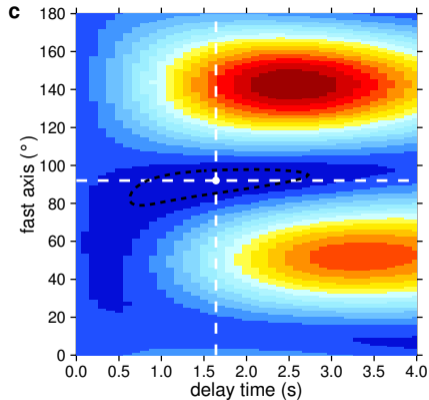

Supplement: Supplementary file 10 — Supplementary Data 8 [file 41467_2023_38296_MOESM10_ESM.zip › TP_DOC_07-May-2021_23_35_12_SKS_average.pdf]

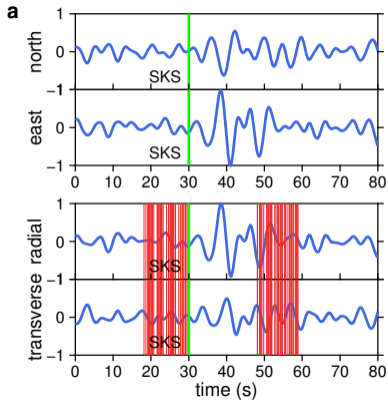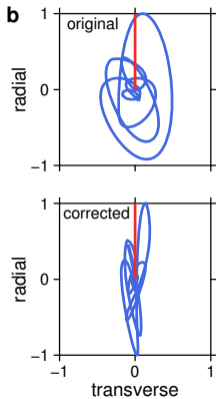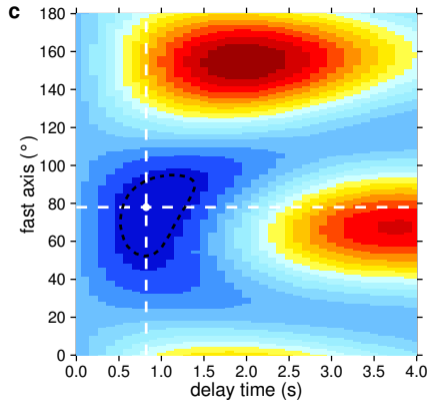

Supplement: Supplementary file 10 — Supplementary Data 8 [file 41467_2023_38296_MOESM10_ESM.zip › TP_DOC_08-Apr-2021_01_00_38_SKS_average.pdf]

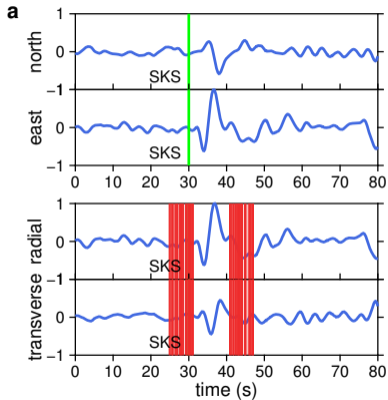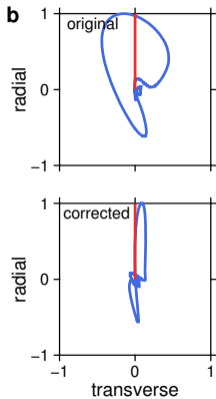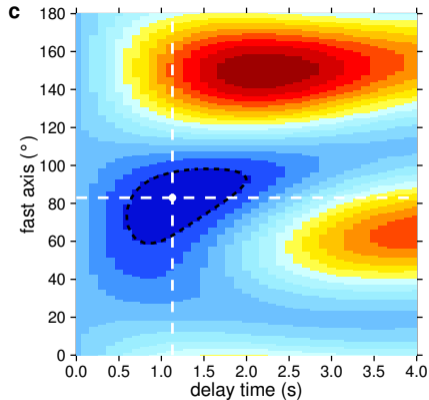

Supplement: Supplementary file 10 — Supplementary Data 8 [file 41467_2023_38296_MOESM10_ESM.zip › TP_DOC_08-Jan-2021_05_01_05_SKS_good.pdf]

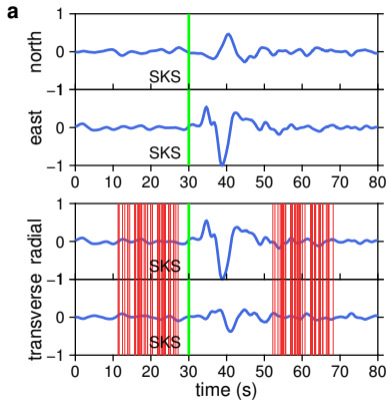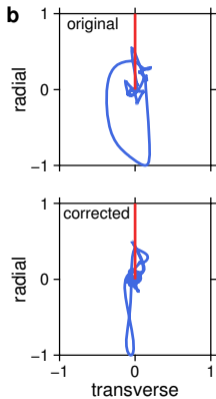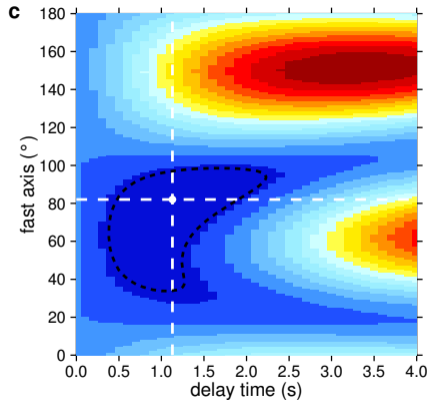

Supplement: Supplementary file 10 — Supplementary Data 8 [file 41467_2023_38296_MOESM10_ESM.zip › TP_DOC_08-Nov-2019_10_44_44_SKS_average.pdf]

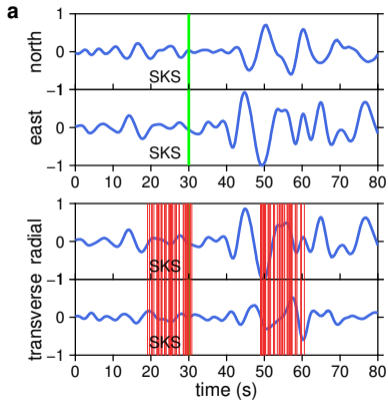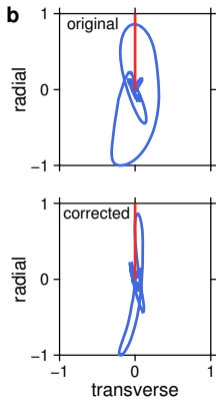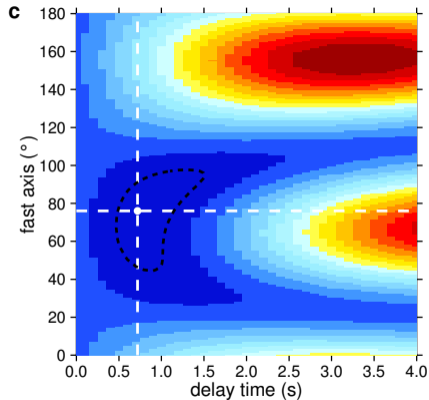

Supplement: Supplementary file 10 — Supplementary Data 8 [file 41467_2023_38296_MOESM10_ESM.zip › TP_DOC_10-Feb-2021_12_24_28_SKS_average.pdf]
