# Supplementary figures and images for: Southern Tibetan rifting since late Miocene enabled by basal shear of the underthrusting Indian lithosphere (part 3 of 4)
Source: Nat Commun. 2023 May 4;14:2565. doi: 10.1038/s41467-023-38296-w (PMC10160080; doi:10.1038/s41467-023-38296-w)

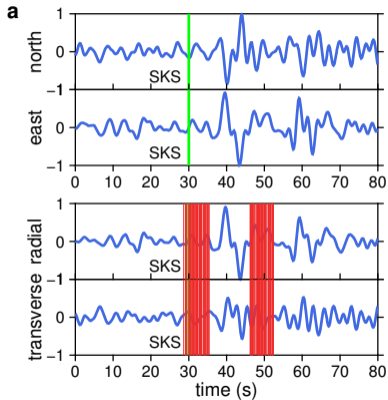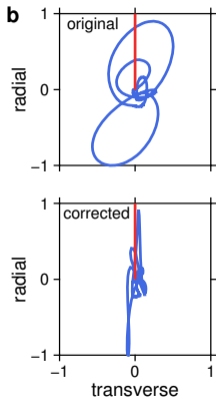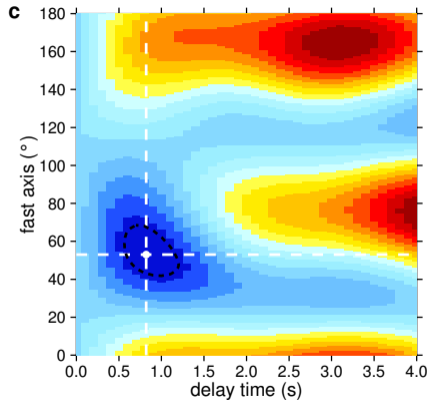

Supplement: Supplementary file 10 — Supplementary Data 8 [file 41467_2023_38296_MOESM10_ESM.zip › TP_CIS_03-Dec-2020_17_07_30_SKS_good.pdf]

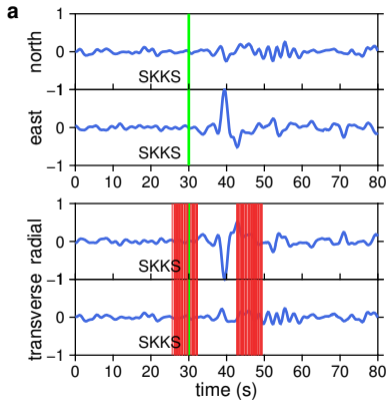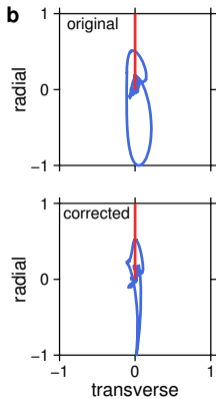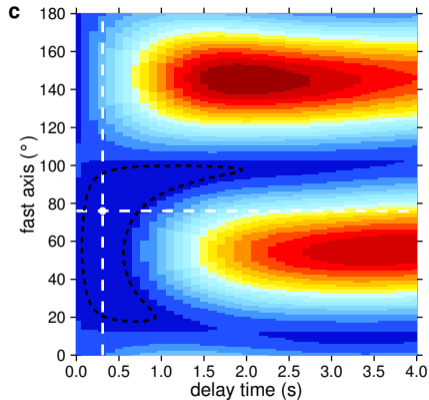

Supplement: Supplementary file 10 — Supplementary Data 8 [file 41467_2023_38296_MOESM10_ESM.zip › TP_CIS_03-Jun-2020_07_35_36_SKKS_average.pdf]

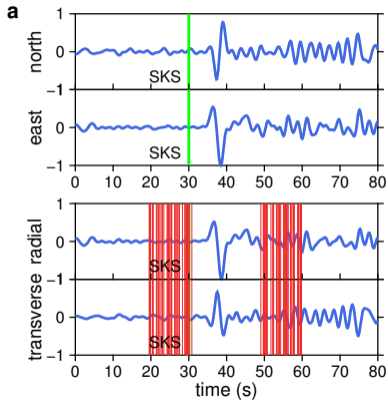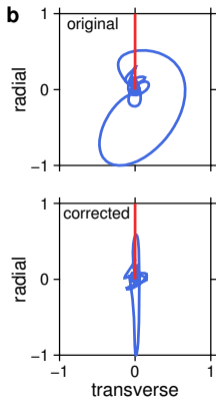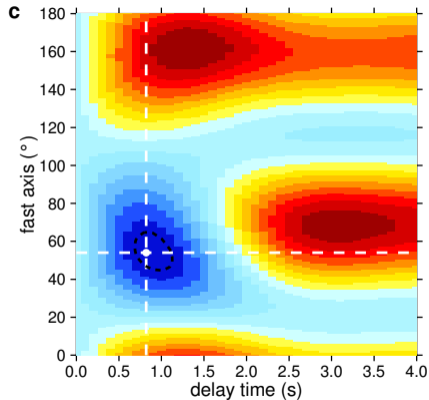

Supplement: Supplementary file 10 — Supplementary Data 8 [file 41467_2023_38296_MOESM10_ESM.zip › TP_CIS_04-Dec-2019_20_10_03_SKS_good.pdf]

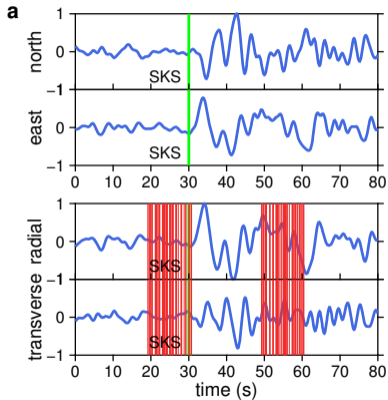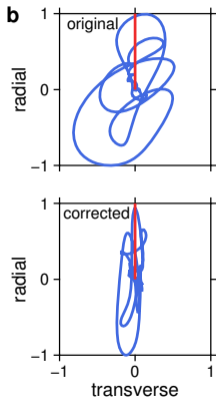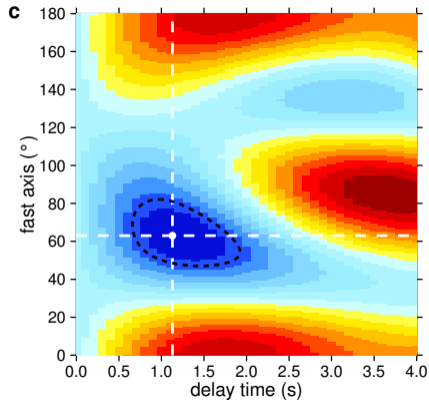

Supplement: Supplementary file 10 — Supplementary Data 8 [file 41467_2023_38296_MOESM10_ESM.zip › TP_CIS_04-Mar-2021_13_27_33_SKS_average.pdf]

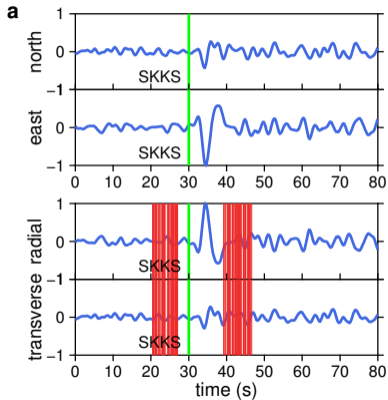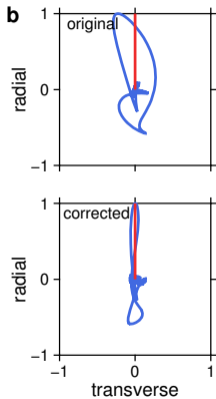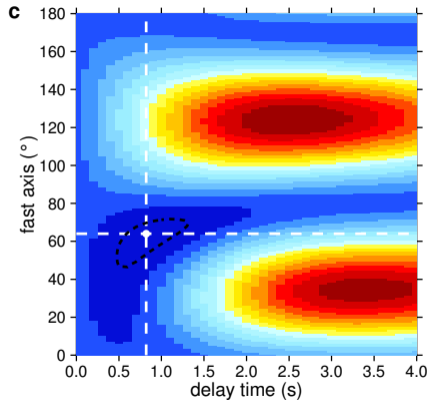

Supplement: Supplementary file 10 — Supplementary Data 8 [file 41467_2023_38296_MOESM10_ESM.zip › TP_CIS_04-Nov-2019_21_53_25_SKKS_average.pdf]

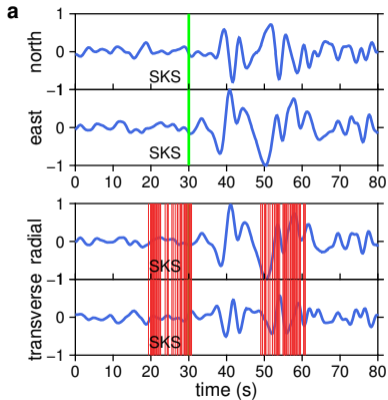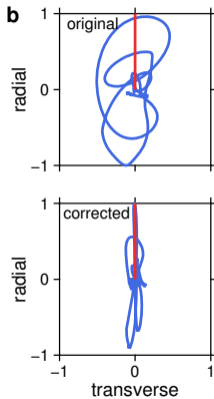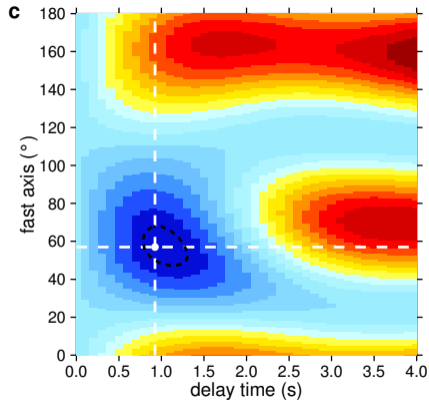

Supplement: Supplementary file 10 — Supplementary Data 8 [file 41467_2023_38296_MOESM10_ESM.zip › TP_CIS_05-Mar-2021_14_24_54_SKS_good.pdf]

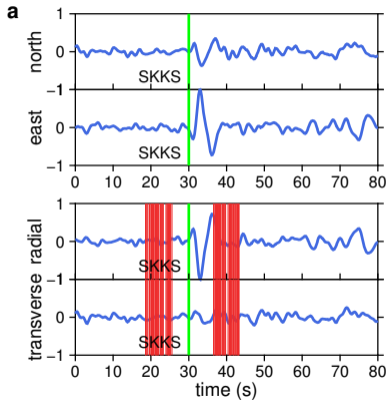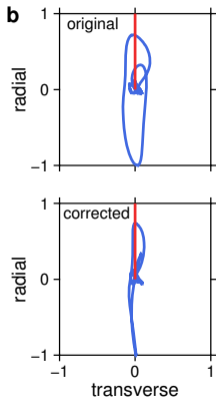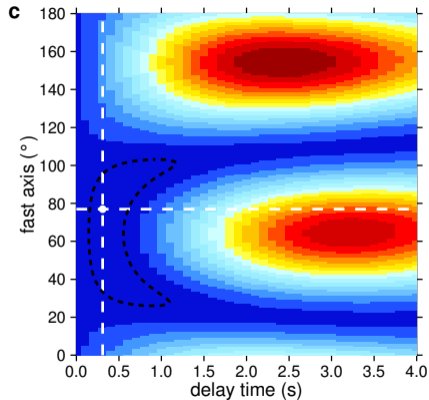

Supplement: Supplementary file 10 — Supplementary Data 8 [file 41467_2023_38296_MOESM10_ESM.zip › TP_CIS_06-Dec-2020_16_47_39_SKKS_average.pdf]

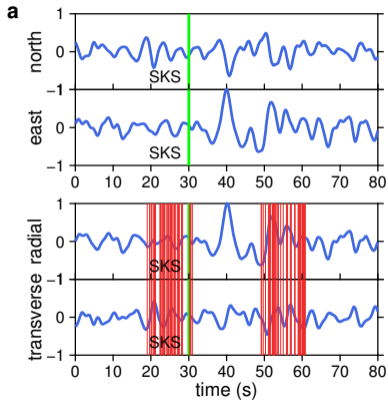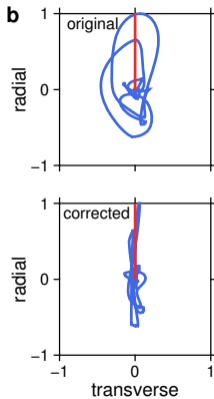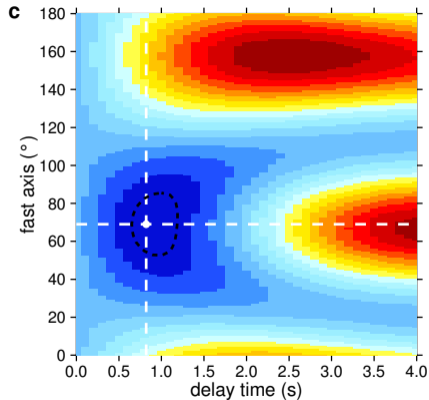

Supplement: Supplementary file 10 — Supplementary Data 8 [file 41467_2023_38296_MOESM10_ESM.zip › TP_CIS_06-Mar-2021_17_13_12_SKS_average.pdf]

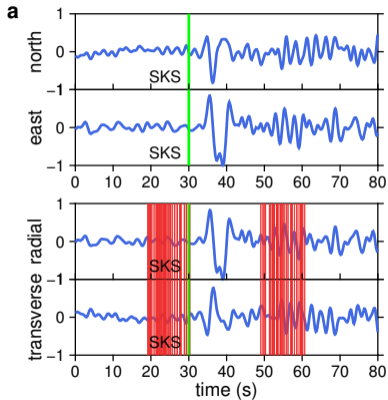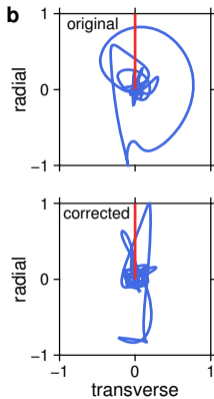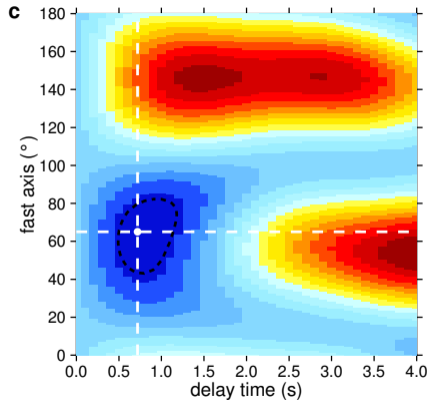

Supplement: Supplementary file 10 — Supplementary Data 8 [file 41467_2023_38296_MOESM10_ESM.zip › TP_CIS_06-Oct-2020_10_11_45_SKS_average.pdf]

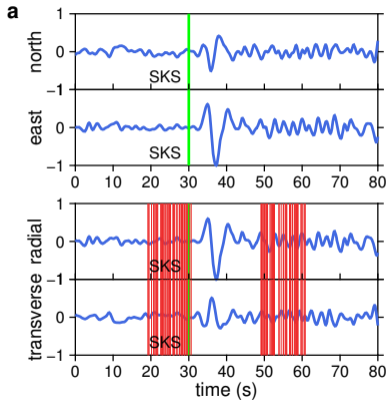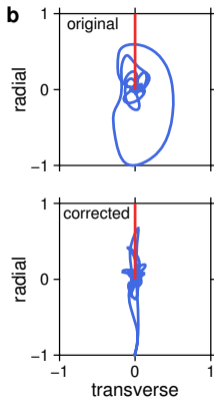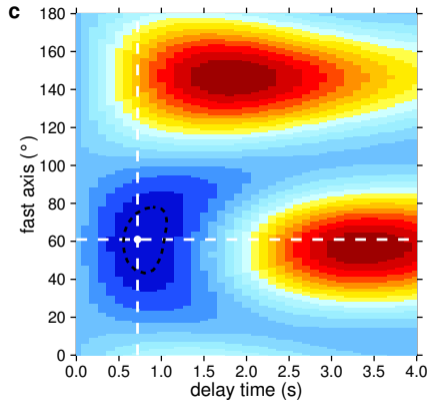

Supplement: Supplementary file 10 — Supplementary Data 8 [file 41467_2023_38296_MOESM10_ESM.zip › TP_CIS_07-May-2021_23_35_12_SKS_good.pdf]

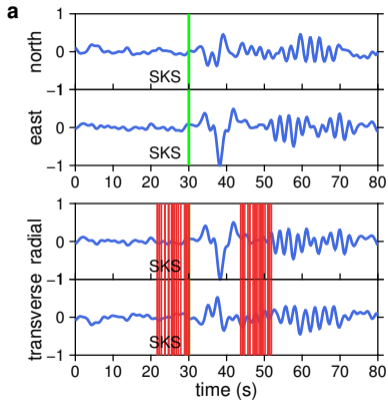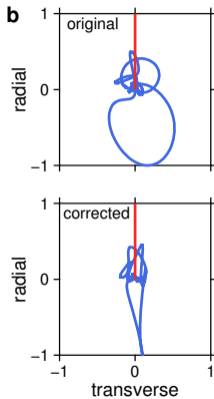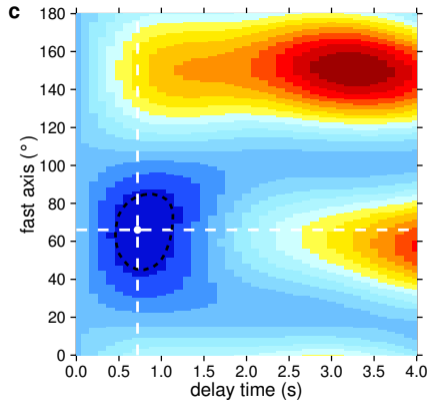

Supplement: Supplementary file 10 — Supplementary Data 8 [file 41467_2023_38296_MOESM10_ESM.zip › TP_CIS_08-Nov-2019_10_44_44_SKS_average.pdf]

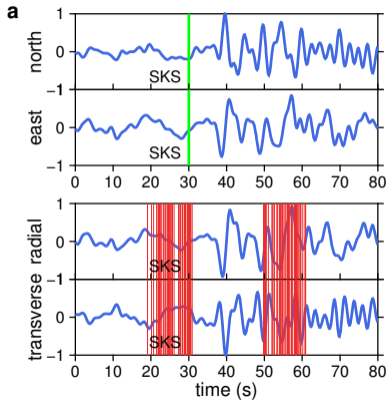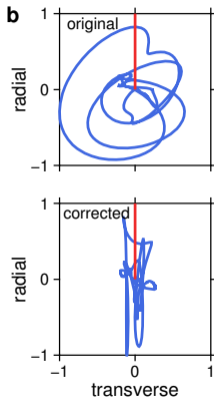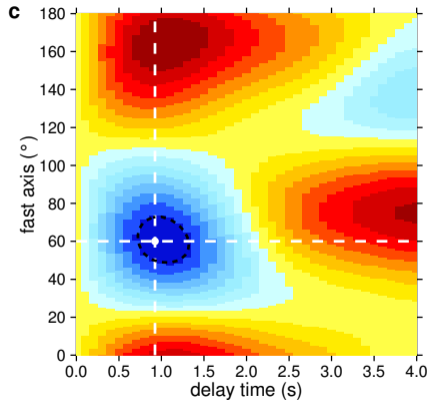

Supplement: Supplementary file 10 — Supplementary Data 8 [file 41467_2023_38296_MOESM10_ESM.zip › TP_CIS_10-Feb-2021_16_35_26_SKS_good.pdf]

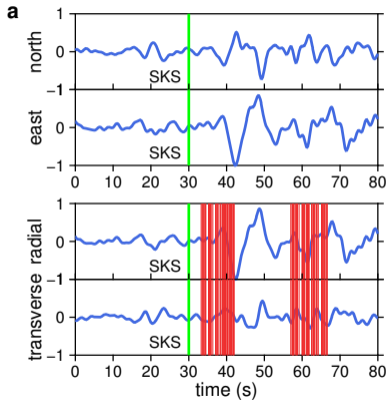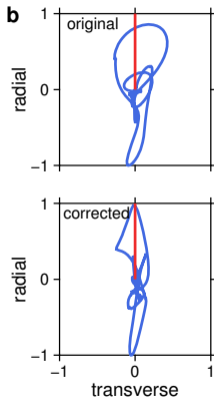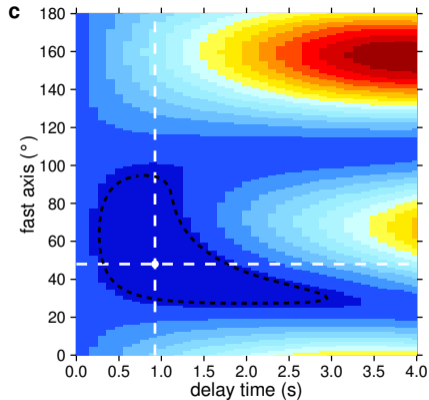

Supplement: Supplementary file 10 — Supplementary Data 8 [file 41467_2023_38296_MOESM10_ESM.zip › TP_CIS_10-Feb-2021_18_36_46_SKS_average.pdf]

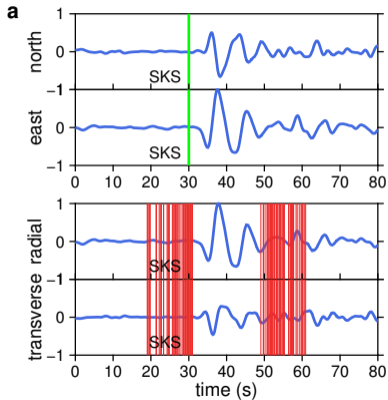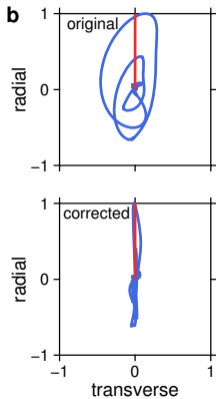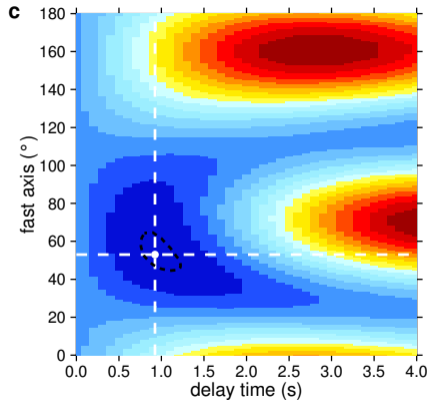

Supplement: Supplementary file 10 — Supplementary Data 8 [file 41467_2023_38296_MOESM10_ESM.zip › TP_CIS_10-Feb-2021_21_23_58_SKS_good.pdf]

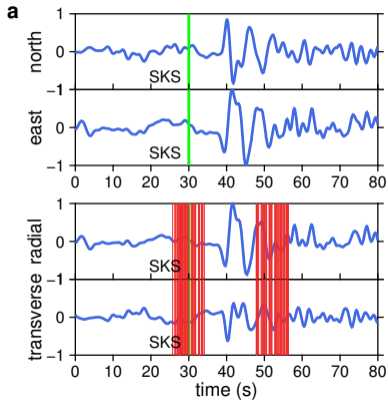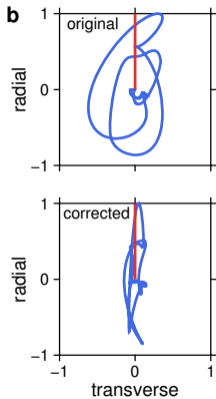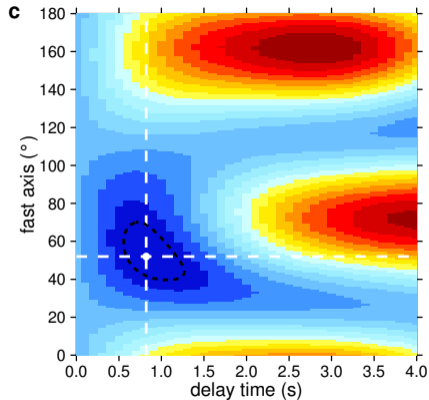

Supplement: Supplementary file 10 — Supplementary Data 8 [file 41467_2023_38296_MOESM10_ESM.zip › TP_CIS_11-Feb-2021_00_14_52_SKS_good.pdf]

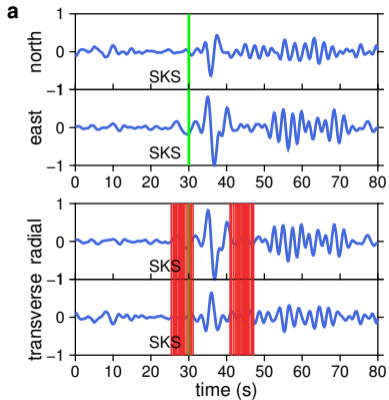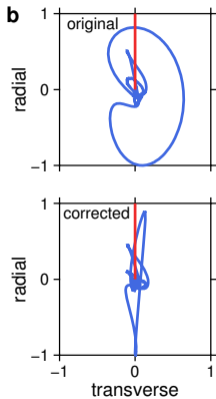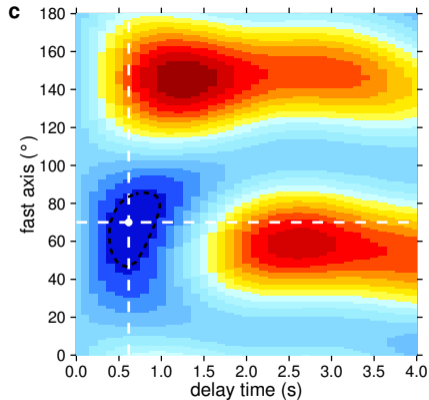

Supplement: Supplementary file 10 — Supplementary Data 8 [file 41467_2023_38296_MOESM10_ESM.zip › TP_CIS_11-Nov-2020_00_48_43_SKS_good.pdf]

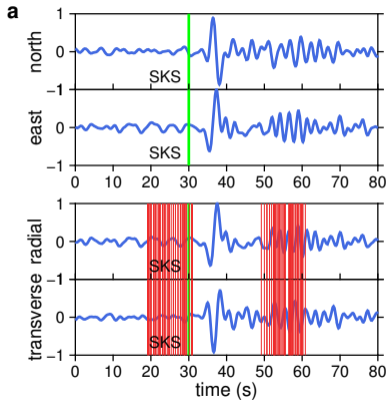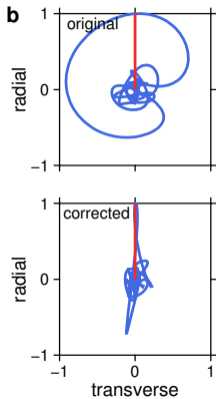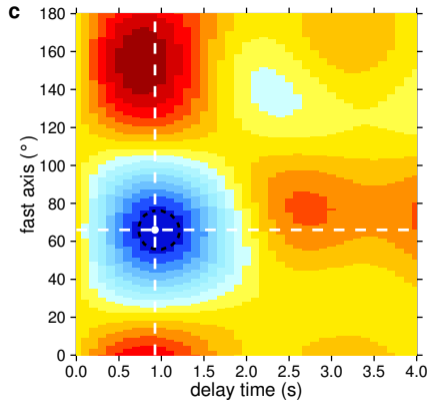

Supplement: Supplementary file 10 — Supplementary Data 8 [file 41467_2023_38296_MOESM10_ESM.zip › TP_CIS_12-Mar-2021_00_03_21_SKS_good.pdf]

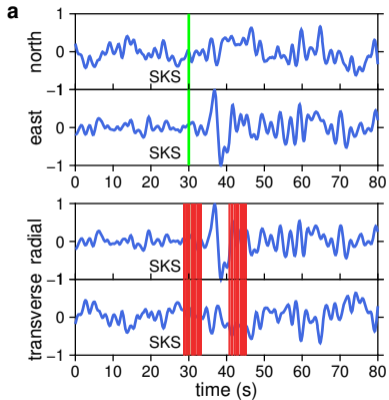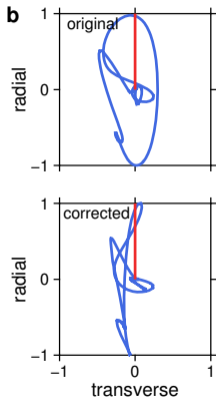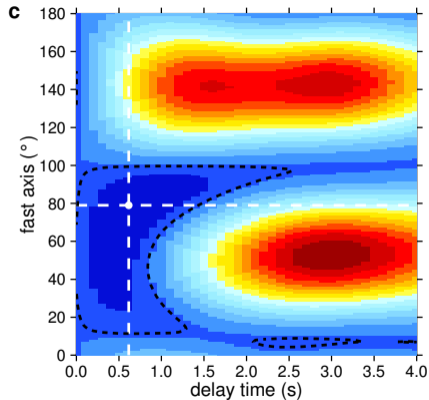

Supplement: Supplementary file 10 — Supplementary Data 8 [file 41467_2023_38296_MOESM10_ESM.zip › TP_CIS_13-May-2021_13_31_34_SKS_average.pdf]

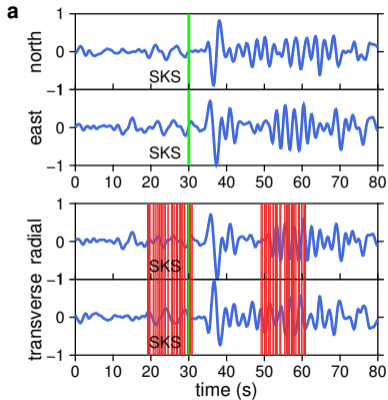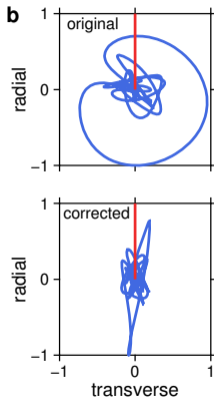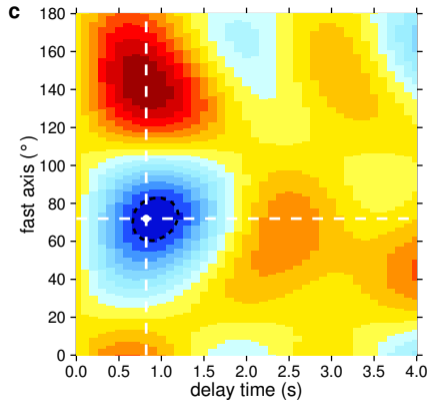

Supplement: Supplementary file 10 — Supplementary Data 8 [file 41467_2023_38296_MOESM10_ESM.zip › TP_CIS_14-Dec-2020_01_57_10_SKS_good.pdf]

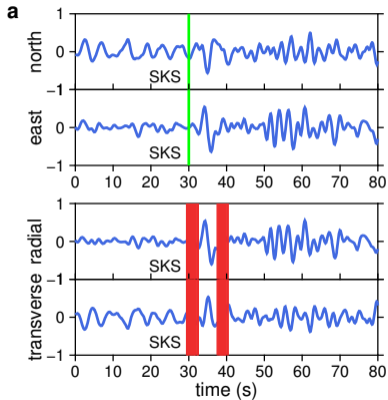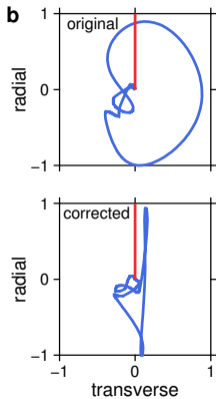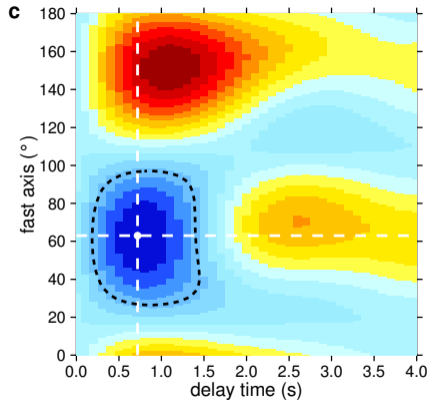

Supplement: Supplementary file 10 — Supplementary Data 8 [file 41467_2023_38296_MOESM10_ESM.zip › TP_CIS_16-Apr-2021_16_40_31_SKS_average.pdf]

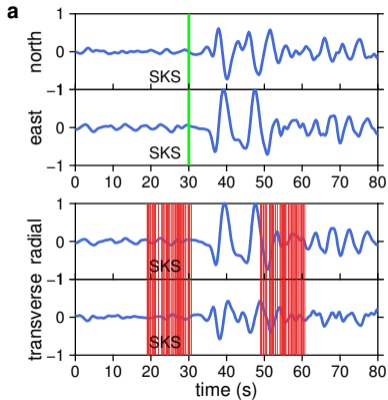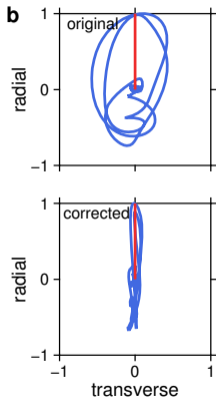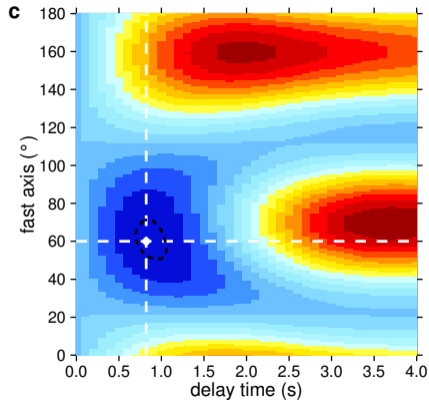

Supplement: Supplementary file 10 — Supplementary Data 8 [file 41467_2023_38296_MOESM10_ESM.zip › TP_CIS_17-Feb-2021_22_49_39_SKS_good.pdf]

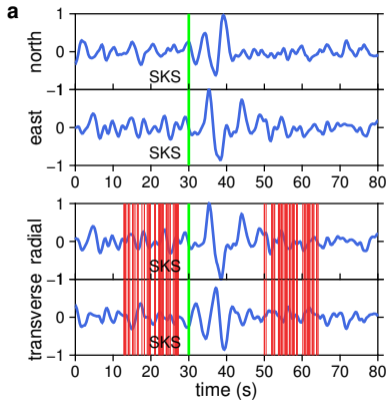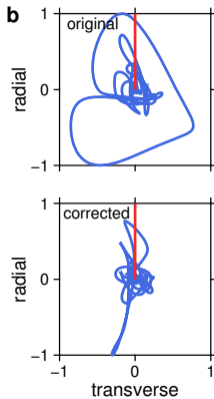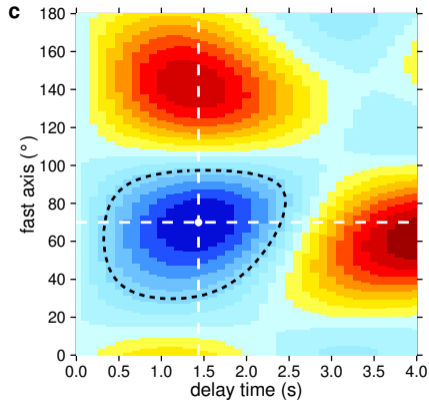

Supplement: Supplementary file 10 — Supplementary Data 8 [file 41467_2023_38296_MOESM10_ESM.zip › TP_CIS_17-Nov-2019_12_13_27_SKS_average.pdf]

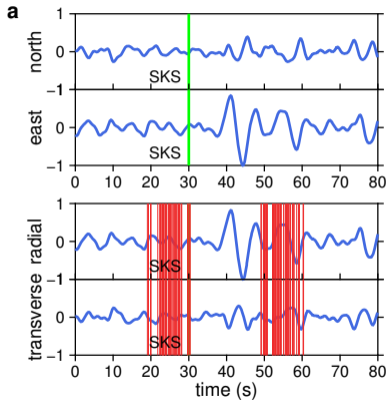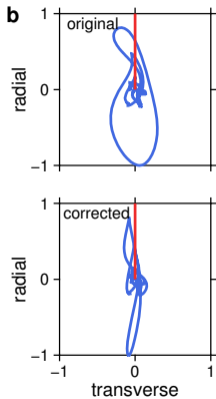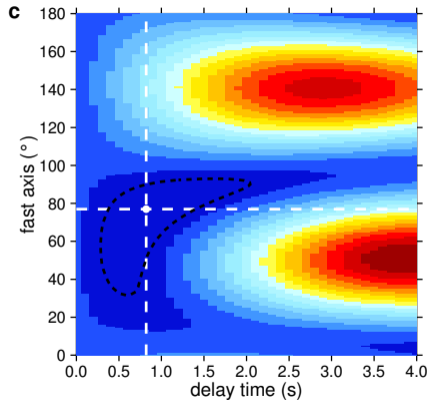

Supplement: Supplementary file 10 — Supplementary Data 8 [file 41467_2023_38296_MOESM10_ESM.zip › TP_CIS_18-Feb-2021_15_30_52_SKS_average.pdf]

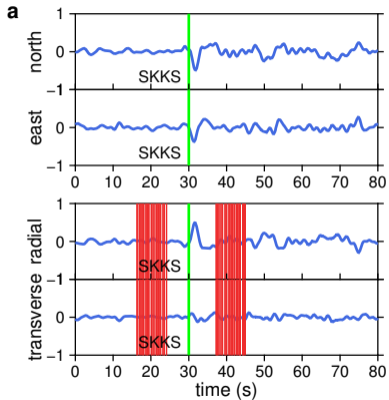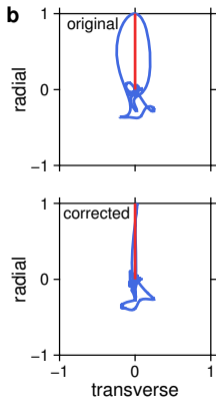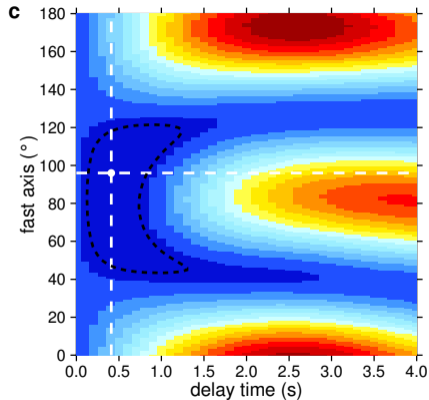

Supplement: Supplementary file 10 — Supplementary Data 8 [file 41467_2023_38296_MOESM10_ESM.zip › TP_CIS_20-Jan-2020_06_51_37_SKKS_average.pdf]

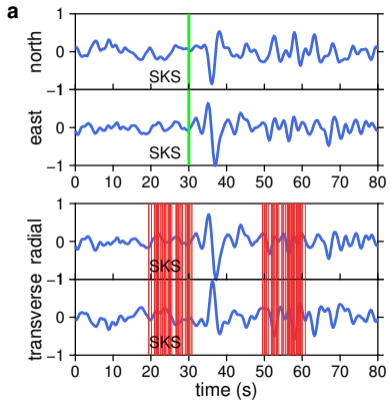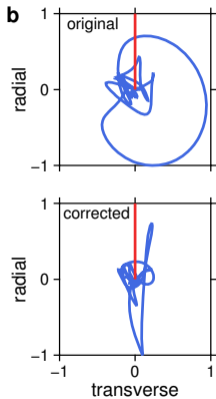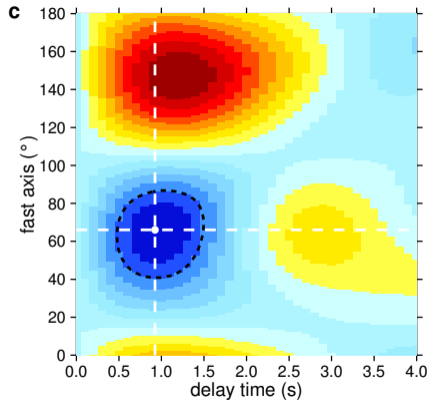

Supplement: Supplementary file 10 — Supplementary Data 8 [file 41467_2023_38296_MOESM10_ESM.zip › TP_CIS_21-Jul-2020_20_56_26_SKS_average.pdf]

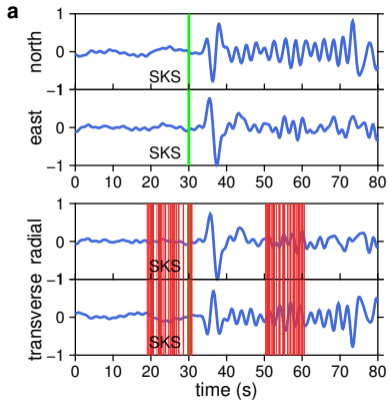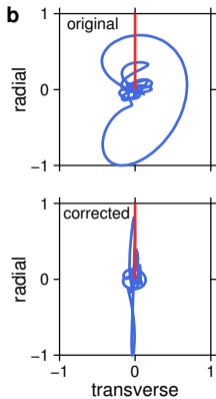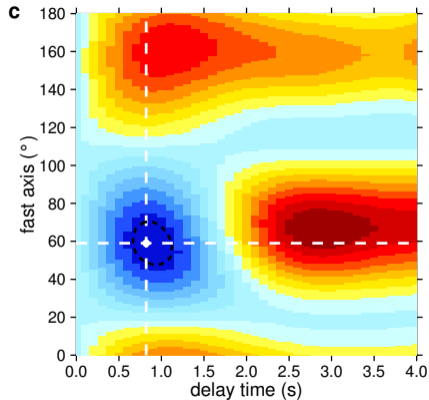

Supplement: Supplementary file 10 — Supplementary Data 8 [file 41467_2023_38296_MOESM10_ESM.zip › TP_CIS_21-Oct-2019_02_52_29_SKS_good.pdf]

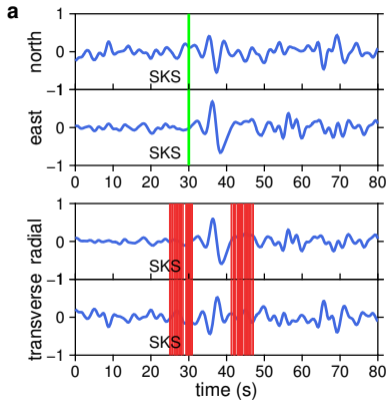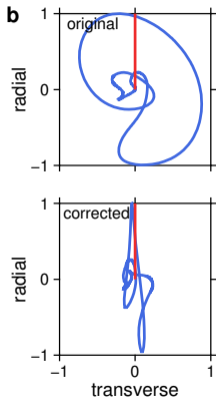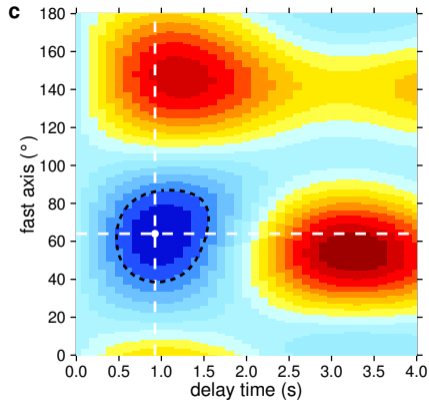

Supplement: Supplementary file 10 — Supplementary Data 8 [file 41467_2023_38296_MOESM10_ESM.zip › TP_CIS_22-Apr-2020_22_31_25_SKS_good.pdf]

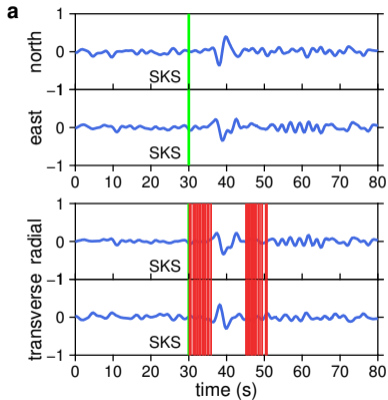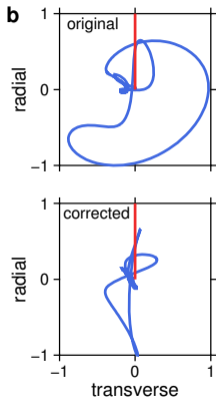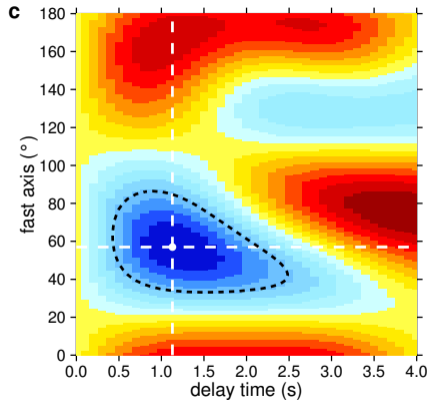

Supplement: Supplementary file 10 — Supplementary Data 8 [file 41467_2023_38296_MOESM10_ESM.zip › TP_CIS_23-Mar-2020_20_33_39_SKS_average.pdf]

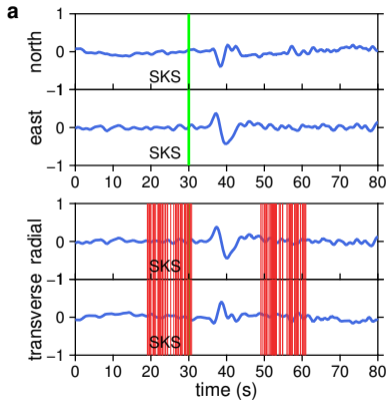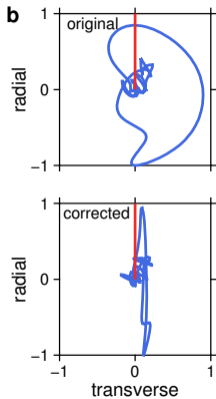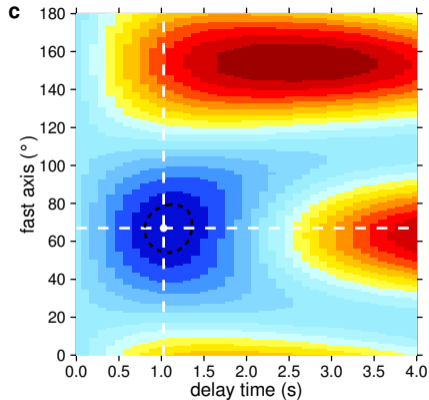

Supplement: Supplementary file 10 — Supplementary Data 8 [file 41467_2023_38296_MOESM10_ESM.zip › TP_CIS_23-Oct-2020_07_04_31_SKS_average.pdf]

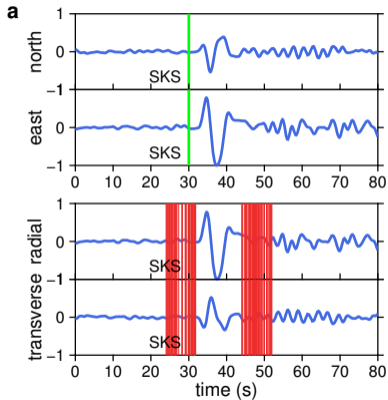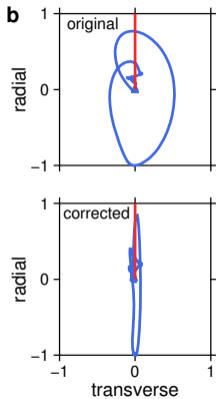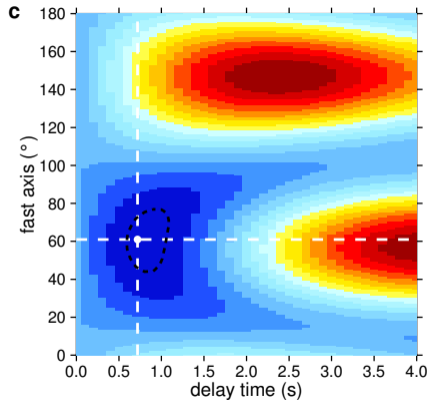

Supplement: Supplementary file 10 — Supplementary Data 8 [file 41467_2023_38296_MOESM10_ESM.zip › TP_CIS_24-Apr-2021_00_23_35_SKS_good.pdf]

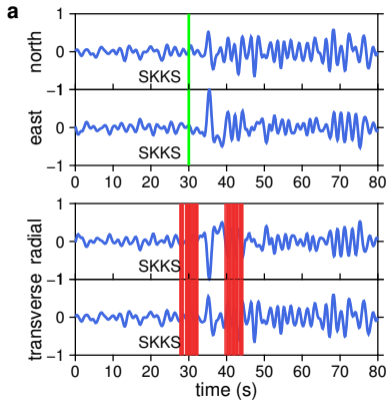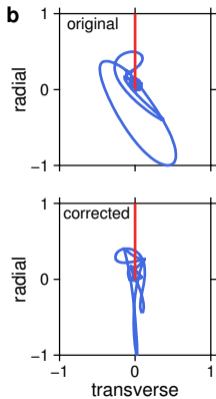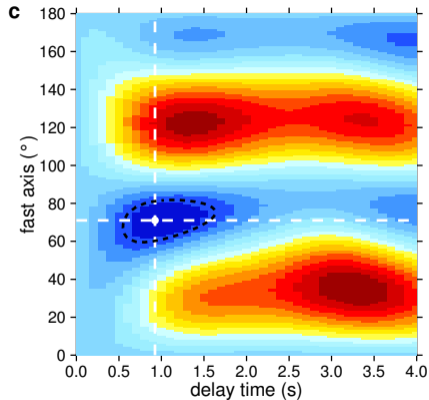

Supplement: Supplementary file 10 — Supplementary Data 8 [file 41467_2023_38296_MOESM10_ESM.zip › TP_CIS_24-Dec-2019_16_43_33_SKKS_average.pdf]

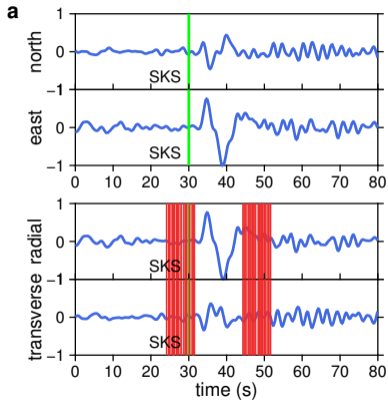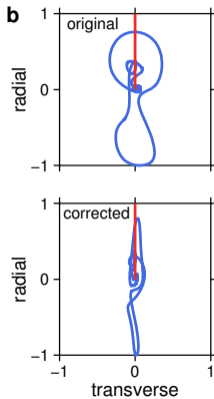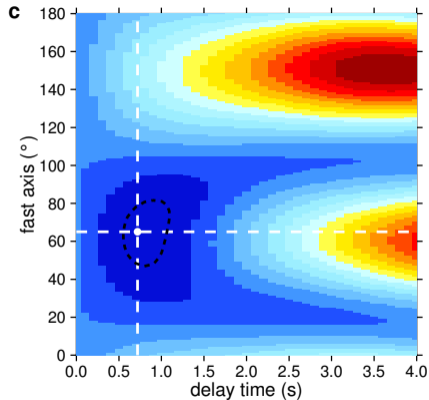

Supplement: Supplementary file 10 — Supplementary Data 8 [file 41467_2023_38296_MOESM10_ESM.zip › TP_CIS_25-Apr-2021_22_28_01_SKS_good.pdf]

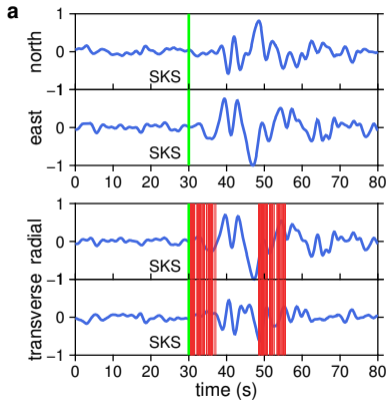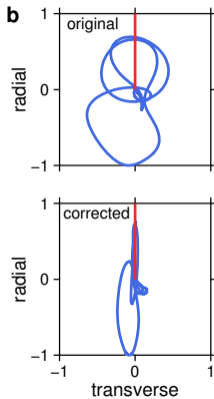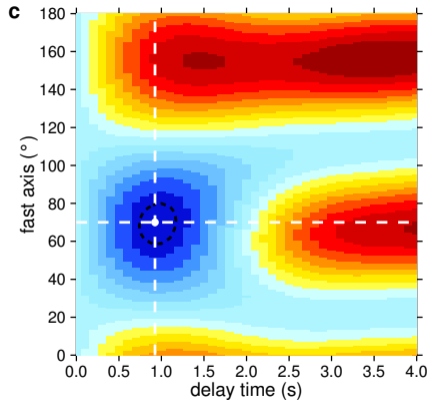

Supplement: Supplementary file 10 — Supplementary Data 8 [file 41467_2023_38296_MOESM10_ESM.zip › TP_CIS_27-Apr-2021_16_33_31_SKS_good.pdf]

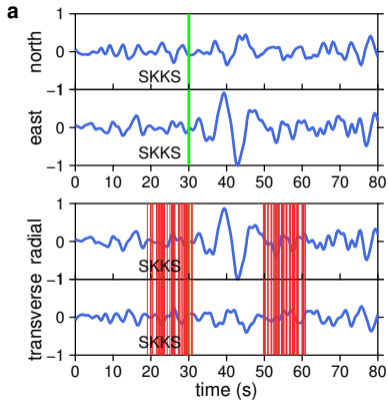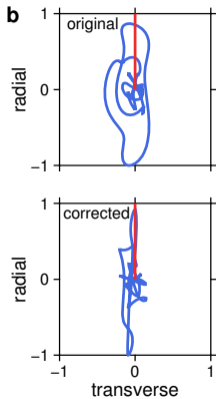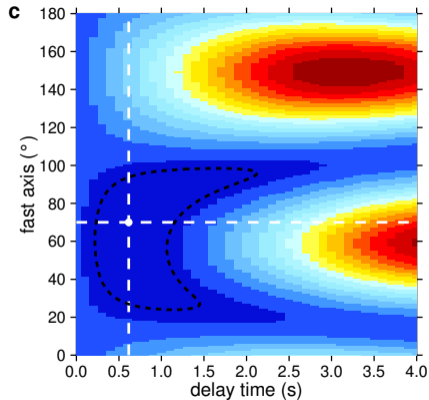

Supplement: Supplementary file 10 — Supplementary Data 8 [file 41467_2023_38296_MOESM10_ESM.zip › TP_CIS_28-May-2020_07_18_42_SKKS_average.pdf]

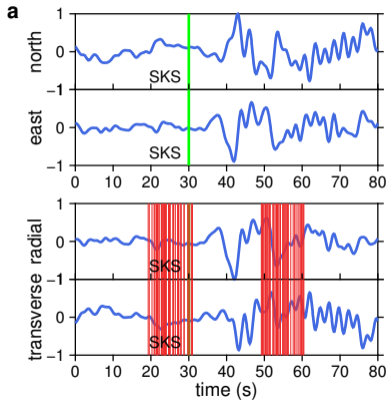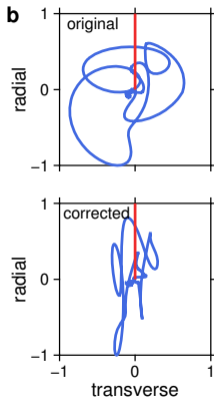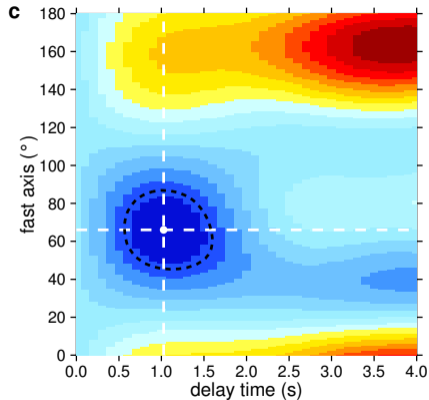

Supplement: Supplementary file 10 — Supplementary Data 8 [file 41467_2023_38296_MOESM10_ESM.zip › TP_CIS_29-Apr-2021_06_50_29_SKS_average.pdf]

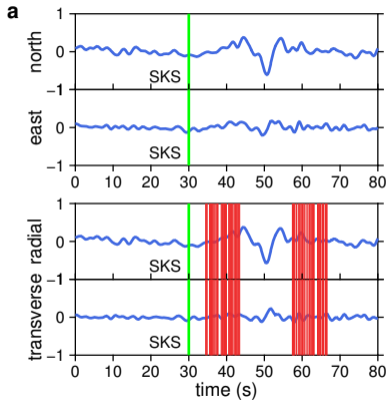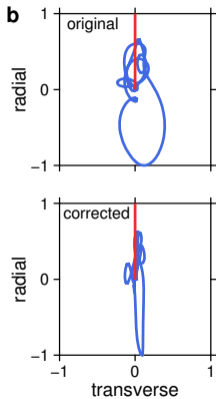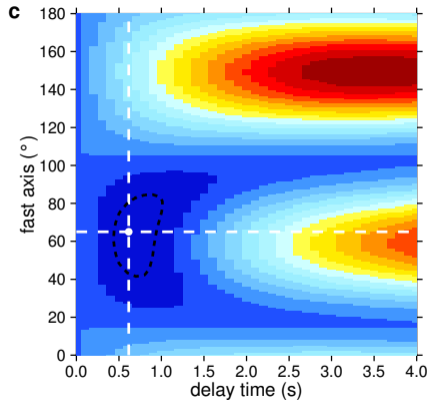

Supplement: Supplementary file 10 — Supplementary Data 8 [file 41467_2023_38296_MOESM10_ESM.zip › TP_CIS_31-Mar-2020_23_52_30_SKS_average.pdf]

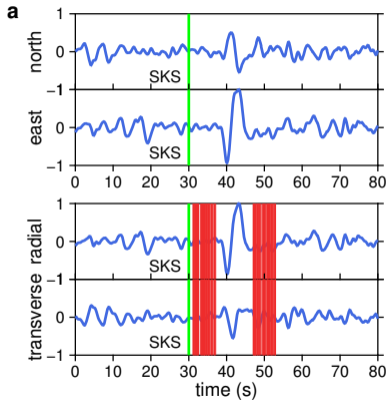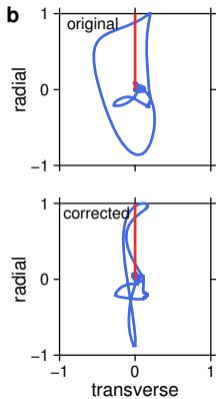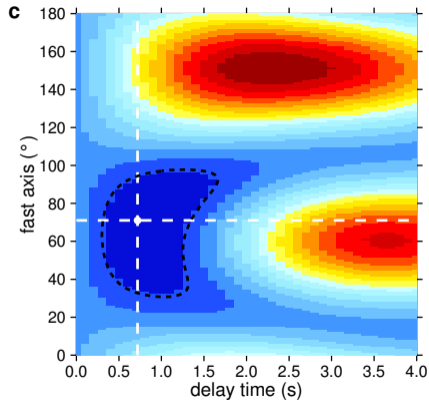

Supplement: Supplementary file 10 — Supplementary Data 8 [file 41467_2023_38296_MOESM10_ESM.zip › TP_CIS_31-May-2020_23_25_43_SKS_average.pdf]

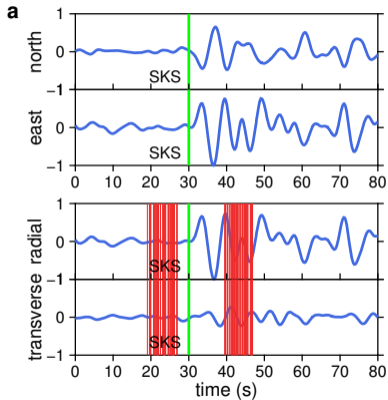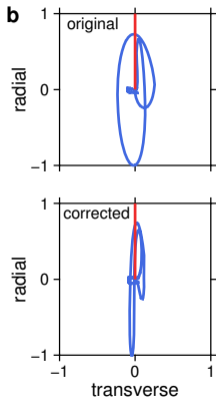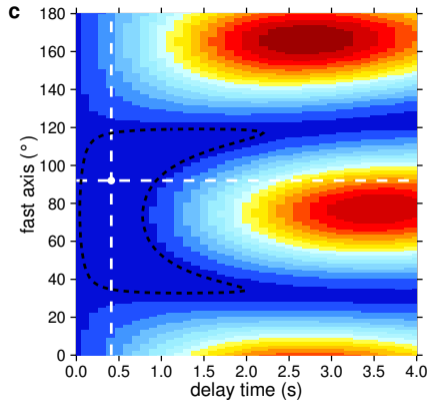

Supplement: Supplementary file 10 — Supplementary Data 8 [file 41467_2023_38296_MOESM10_ESM.zip › TP_CIW_04-Mar-2021_13_27_33_SKS_average.pdf]

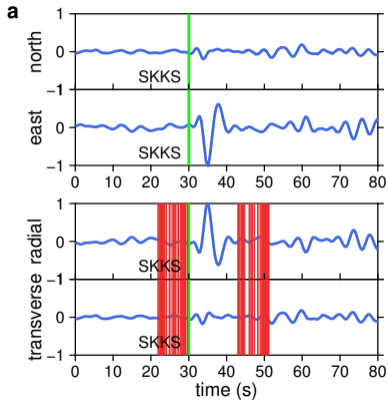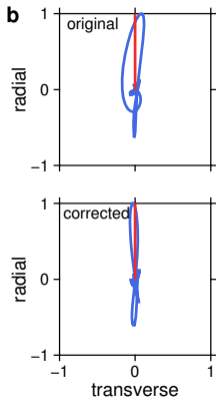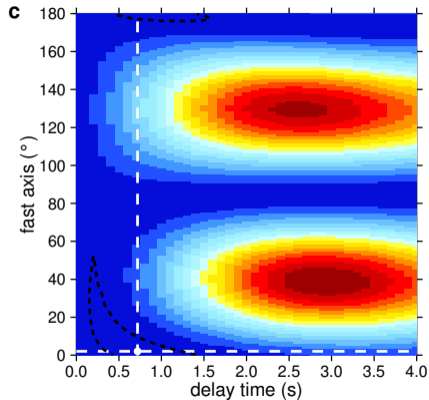

Supplement: Supplementary file 10 — Supplementary Data 8 [file 41467_2023_38296_MOESM10_ESM.zip › TP_CIW_04-Nov-2019_21_53_25_SKKS_average.pdf]

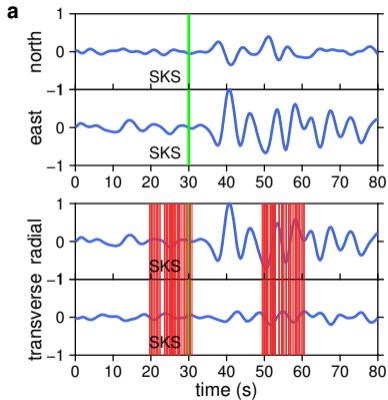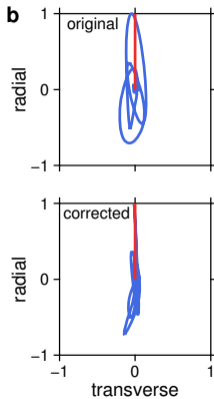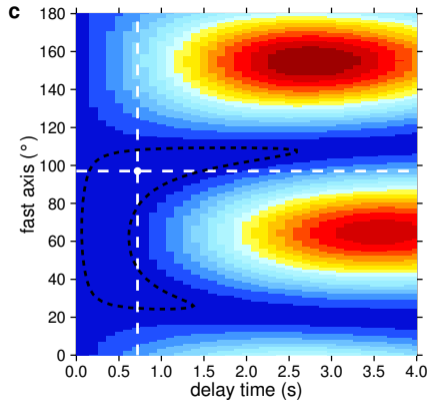

Supplement: Supplementary file 10 — Supplementary Data 8 [file 41467_2023_38296_MOESM10_ESM.zip › TP_CIW_05-Mar-2021_14_24_54_SKS_average.pdf]

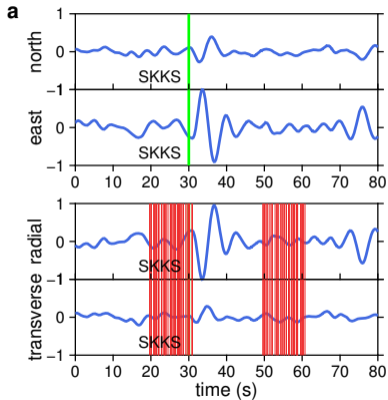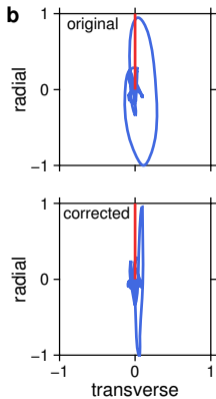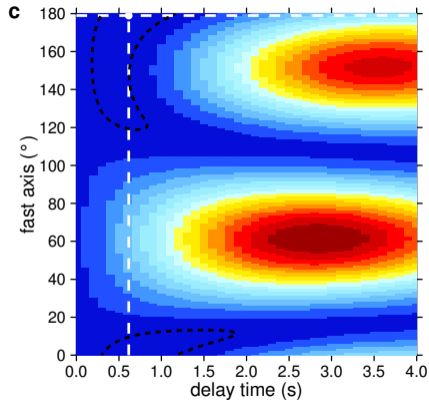

Supplement: Supplementary file 10 — Supplementary Data 8 [file 41467_2023_38296_MOESM10_ESM.zip › TP_CIW_06-Dec-2020_16_47_39_SKKS_average.pdf]

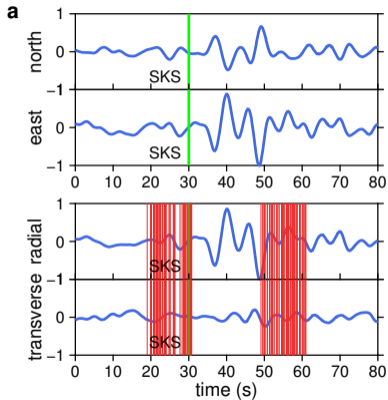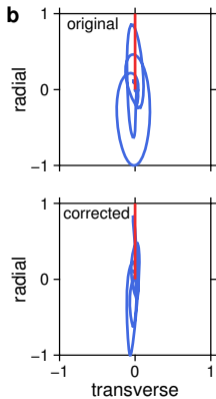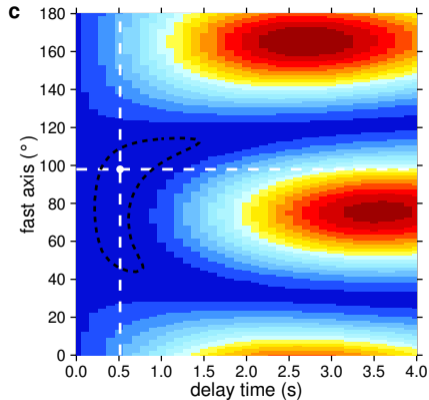

Supplement: Supplementary file 10 — Supplementary Data 8 [file 41467_2023_38296_MOESM10_ESM.zip › TP_CIW_06-Mar-2021_00_16_14_SKS_average.pdf]

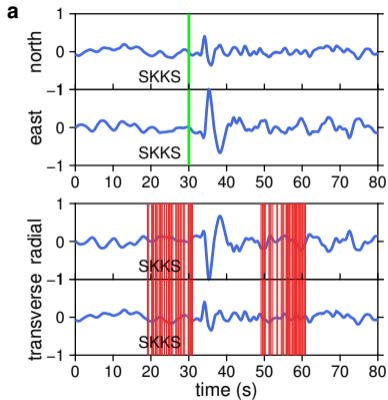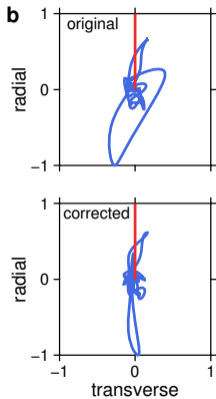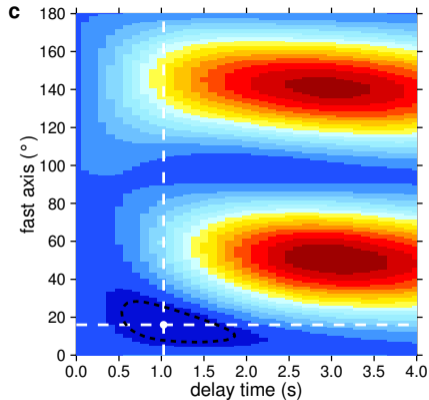

Supplement: Supplementary file 10 — Supplementary Data 8 [file 41467_2023_38296_MOESM10_ESM.zip › TP_CIW_24-Dec-2019_16_43_33_SKKS_average.pdf]

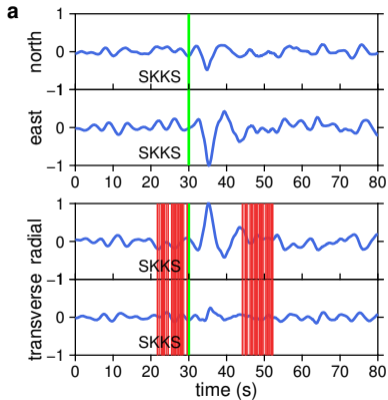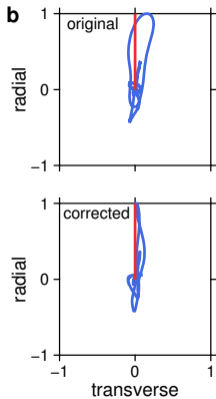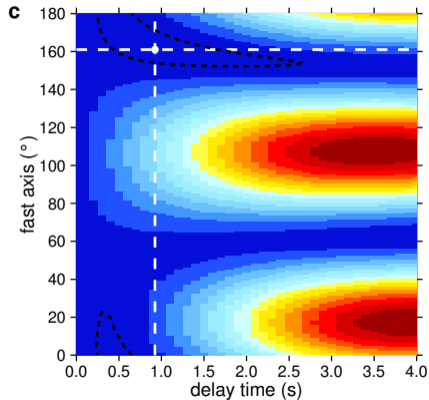

Supplement: Supplementary file 10 — Supplementary Data 8 [file 41467_2023_38296_MOESM10_ESM.zip › TP_CIW_26-Sep-2019_16_36_18_SKKS_average.pdf]

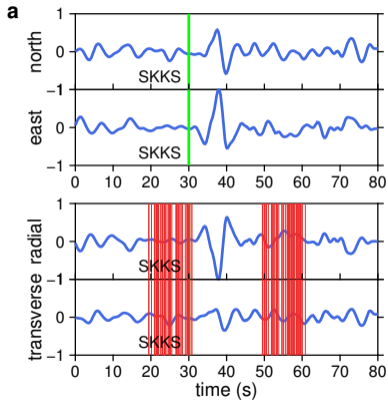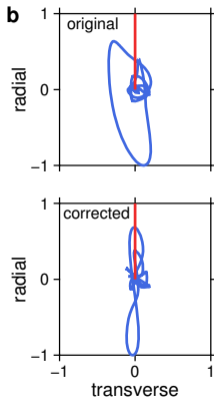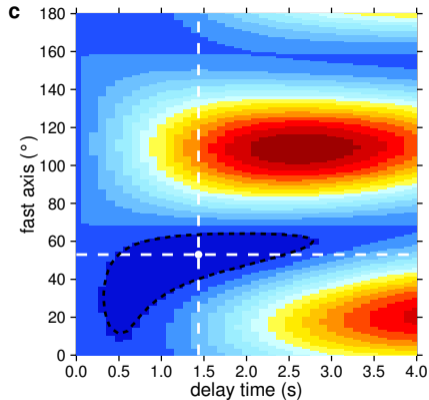

Supplement: Supplementary file 10 — Supplementary Data 8 [file 41467_2023_38296_MOESM10_ESM.zip › TP_CIW_29-Apr-2021_02_17_16_SKKS_average.pdf]

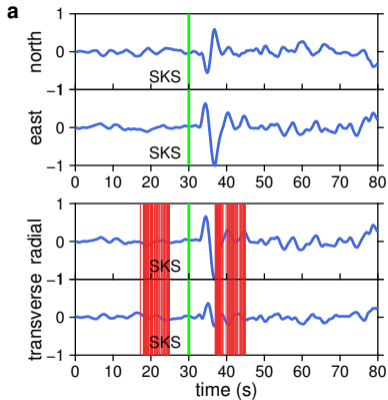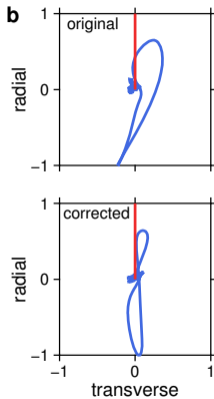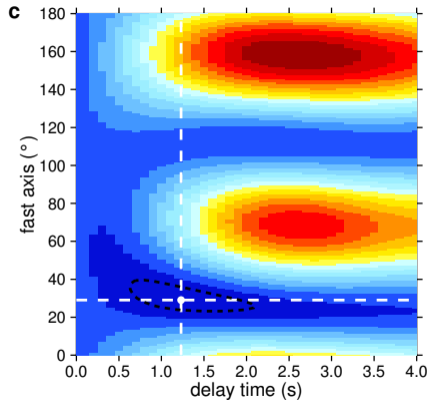

Supplement: Supplementary file 10 — Supplementary Data 8 [file 41467_2023_38296_MOESM10_ESM.zip › TP_CUM_01-Apr-2021_15_11_18_SKS_average.pdf]

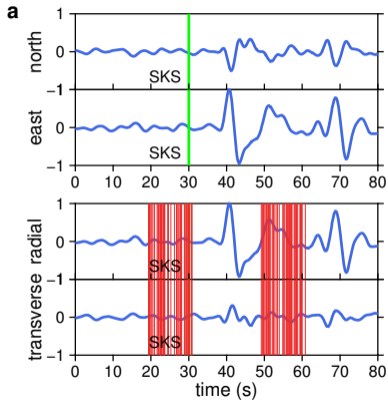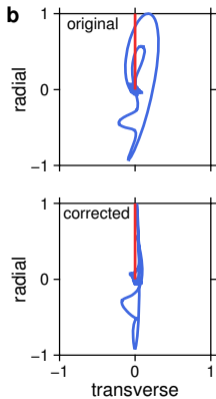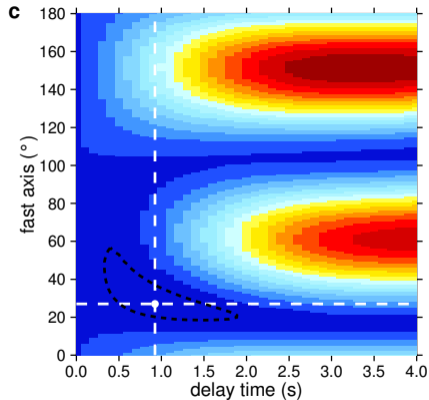

Supplement: Supplementary file 10 — Supplementary Data 8 [file 41467_2023_38296_MOESM10_ESM.zip › TP_CUM_01-Oct-2020_01_13_41_SKS_average.pdf]

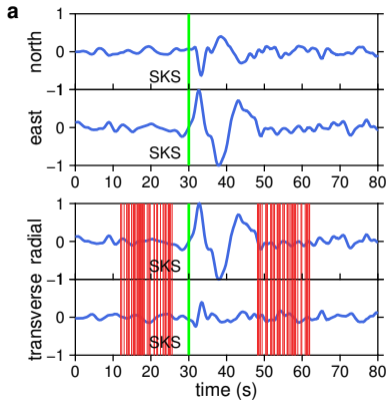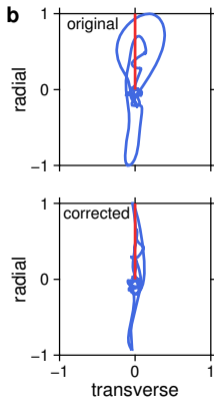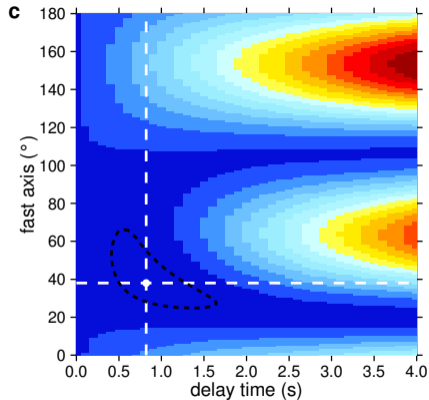

Supplement: Supplementary file 10 — Supplementary Data 8 [file 41467_2023_38296_MOESM10_ESM.zip › TP_CUM_01-Sep-2019_15_54_20_SKS_average.pdf]

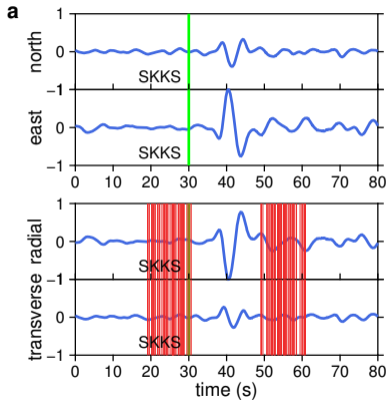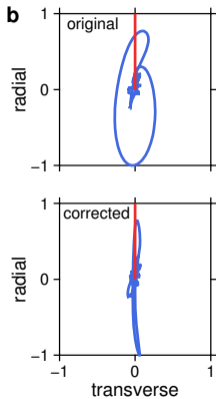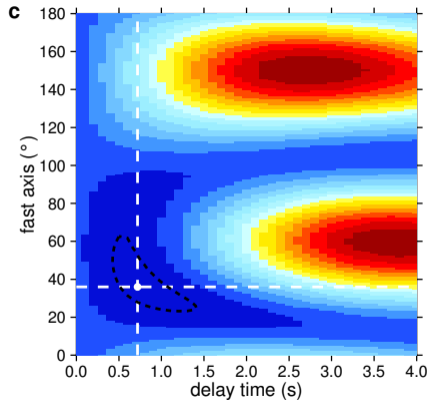

Supplement: Supplementary file 10 — Supplementary Data 8 [file 41467_2023_38296_MOESM10_ESM.zip › TP_CUM_03-Jun-2020_07_35_36_SKKS_good.pdf]

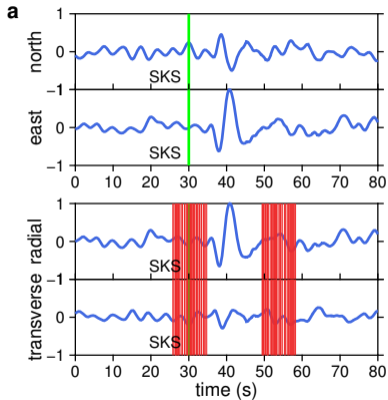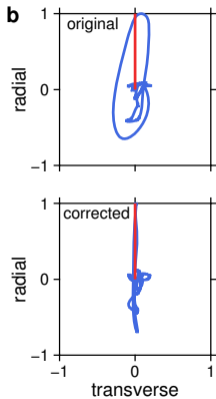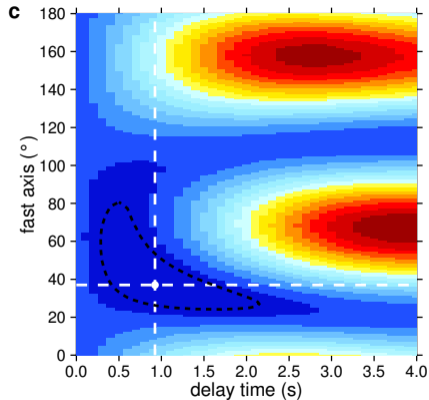

Supplement: Supplementary file 10 — Supplementary Data 8 [file 41467_2023_38296_MOESM10_ESM.zip › TP_CUM_03-Jun-2020_09_31_39_SKS_average.pdf]

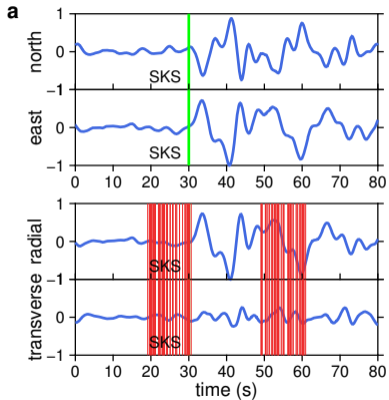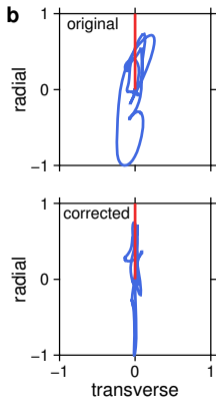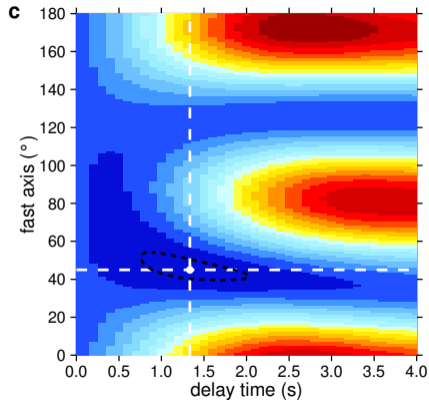

Supplement: Supplementary file 10 — Supplementary Data 8 [file 41467_2023_38296_MOESM10_ESM.zip › TP_CUM_04-Mar-2021_13_27_33_SKS_average.pdf]

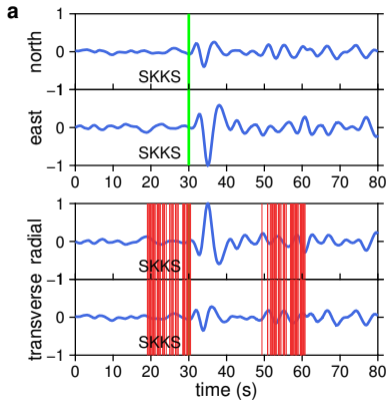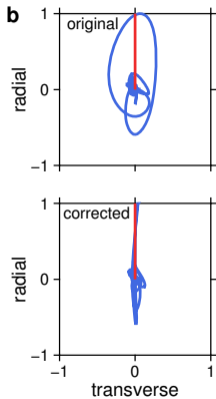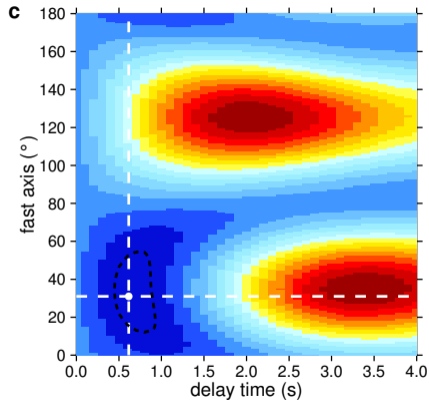

Supplement: Supplementary file 10 — Supplementary Data 8 [file 41467_2023_38296_MOESM10_ESM.zip › TP_CUM_04-Nov-2019_21_53_25_SKKS_good.pdf]

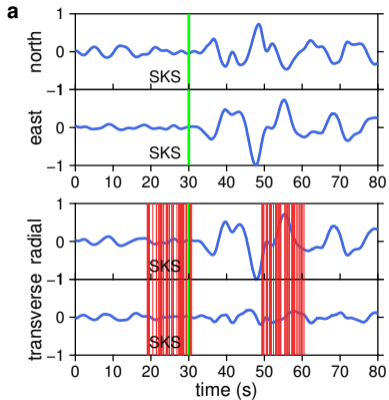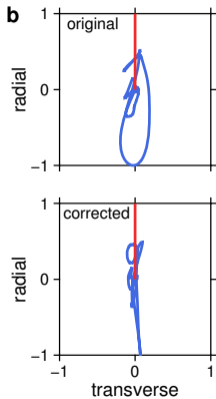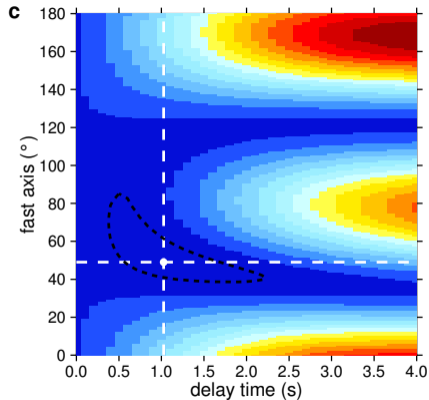

Supplement: Supplementary file 10 — Supplementary Data 8 [file 41467_2023_38296_MOESM10_ESM.zip › TP_CUM_06-Mar-2021_00_16_14_SKS_average.pdf]

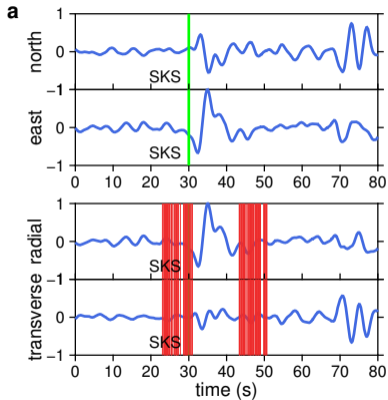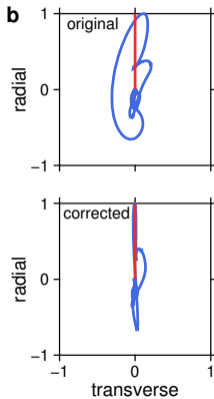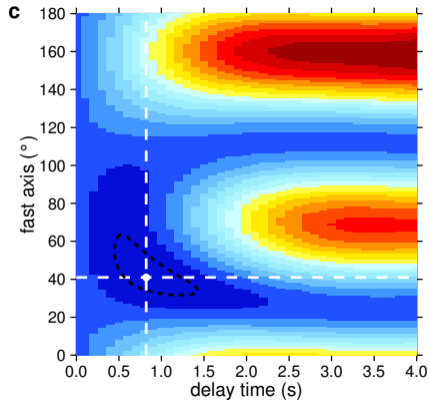

Supplement: Supplementary file 10 — Supplementary Data 8 [file 41467_2023_38296_MOESM10_ESM.zip › TP_CUM_08-Jan-2021_05_01_05_SKS_good.pdf]

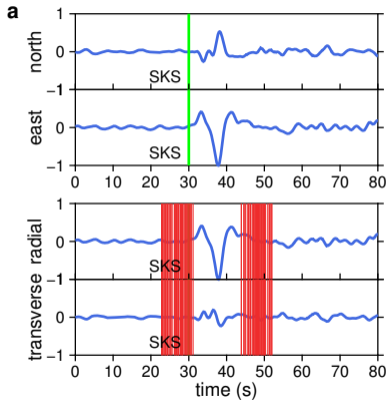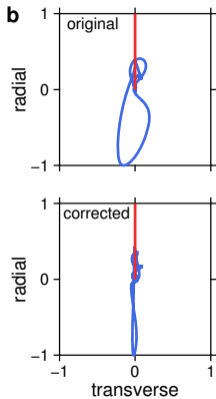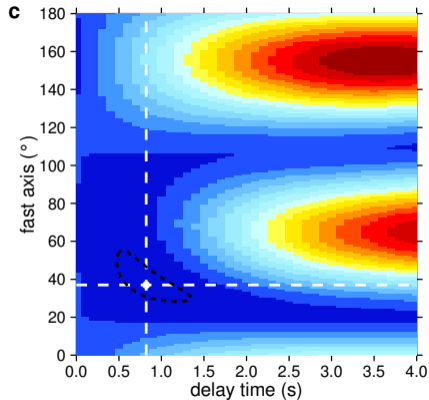

Supplement: Supplementary file 10 — Supplementary Data 8 [file 41467_2023_38296_MOESM10_ESM.zip › TP_CUM_08-Nov-2019_10_44_44_SKS_good.pdf]

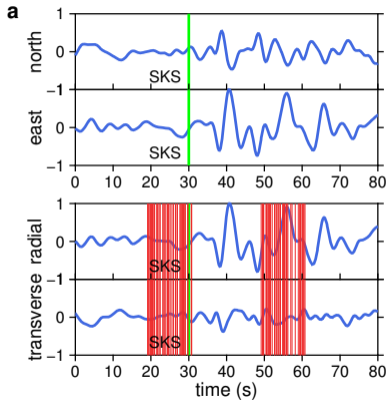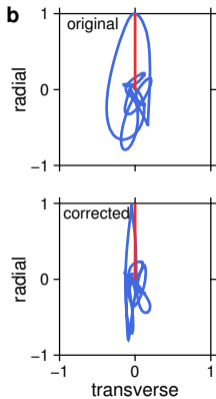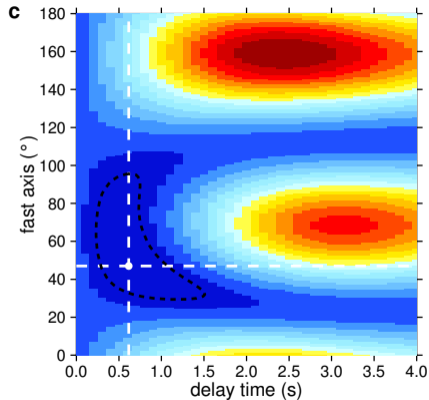

Supplement: Supplementary file 10 — Supplementary Data 8 [file 41467_2023_38296_MOESM10_ESM.zip › TP_CUM_10-Feb-2021_16_35_26_SKS_average.pdf]

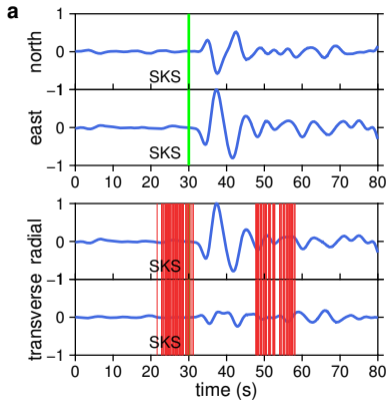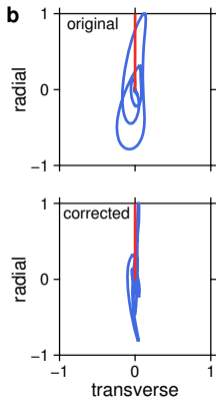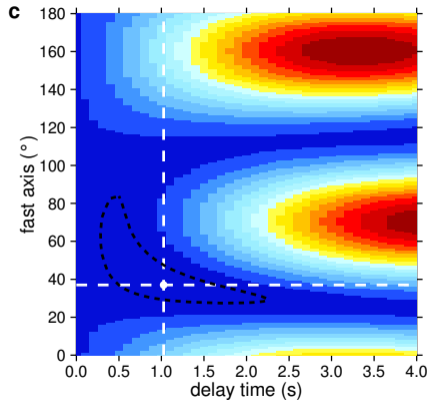

Supplement: Supplementary file 10 — Supplementary Data 8 [file 41467_2023_38296_MOESM10_ESM.zip › TP_CUM_10-Feb-2021_21_23_58_SKS_average.pdf]

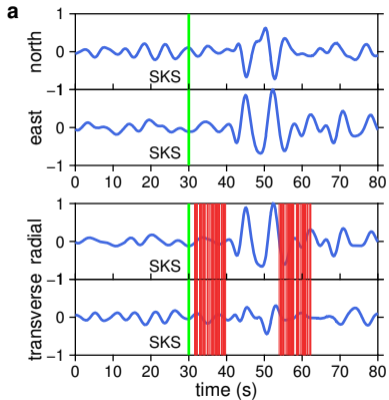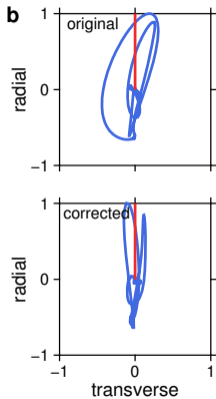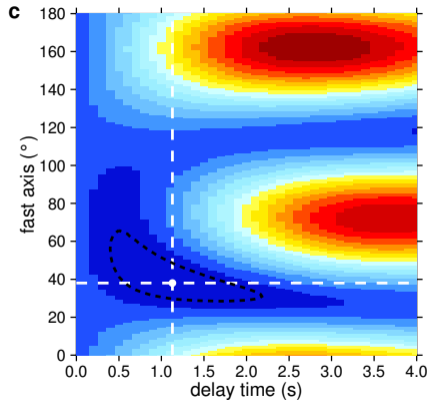

Supplement: Supplementary file 10 — Supplementary Data 8 [file 41467_2023_38296_MOESM10_ESM.zip › TP_CUM_10-Feb-2021_23_45_22_SKS_average.pdf]

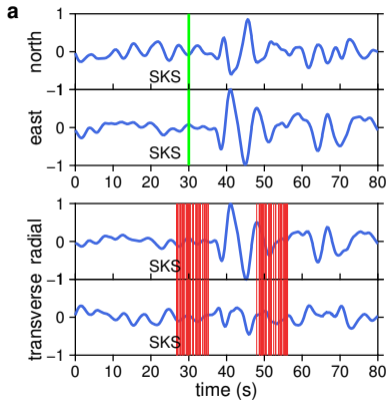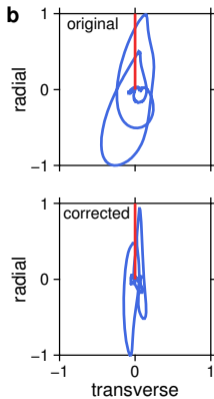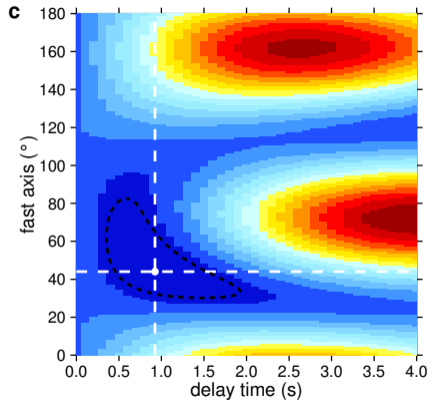

Supplement: Supplementary file 10 — Supplementary Data 8 [file 41467_2023_38296_MOESM10_ESM.zip › TP_CUM_11-Feb-2021_00_14_52_SKS_average.pdf]

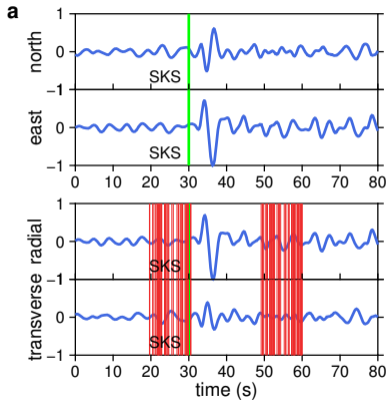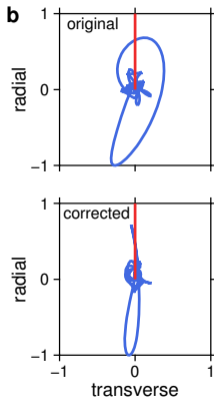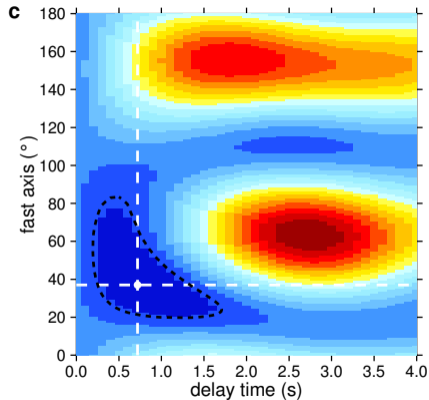

Supplement: Supplementary file 10 — Supplementary Data 8 [file 41467_2023_38296_MOESM10_ESM.zip › TP_CUM_11-Nov-2020_00_48_43_SKS_average.pdf]

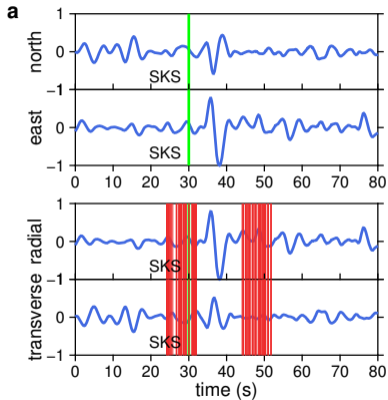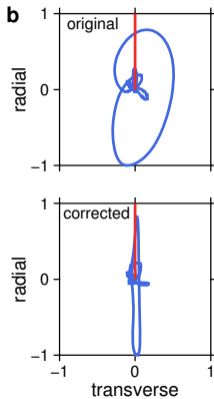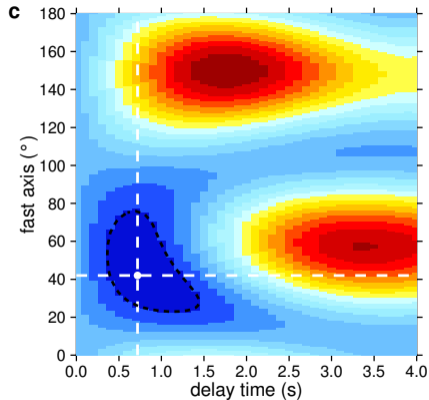

Supplement: Supplementary file 10 — Supplementary Data 8 [file 41467_2023_38296_MOESM10_ESM.zip › TP_CUM_13-May-2021_13_31_34_SKS_good.pdf]

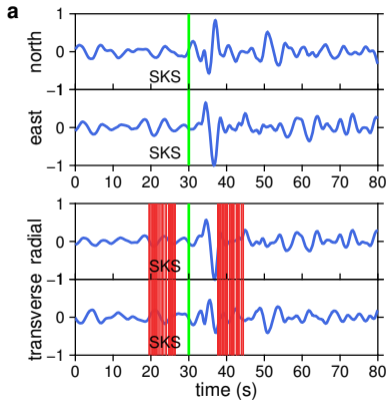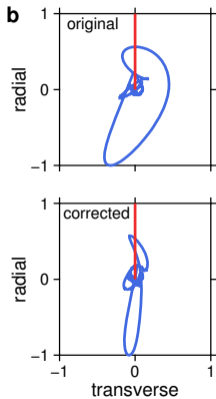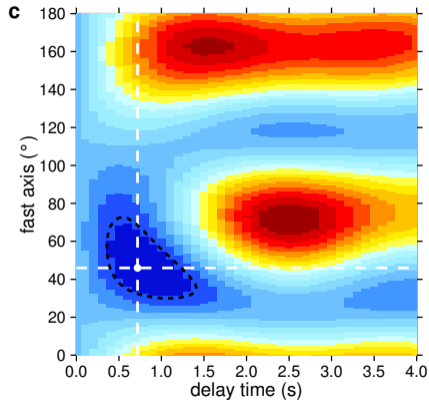

Supplement: Supplementary file 10 — Supplementary Data 8 [file 41467_2023_38296_MOESM10_ESM.zip › TP_CUM_14-Dec-2020_01_57_10_SKS_average.pdf]

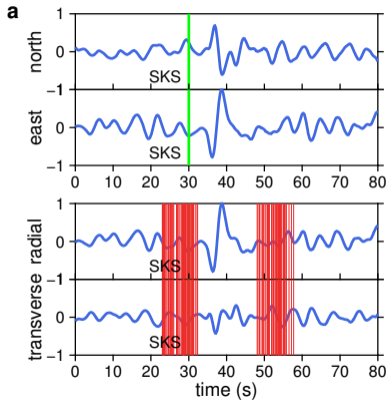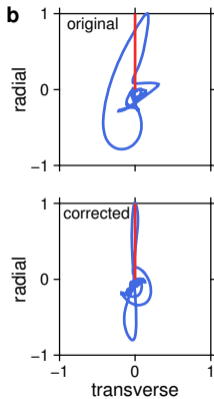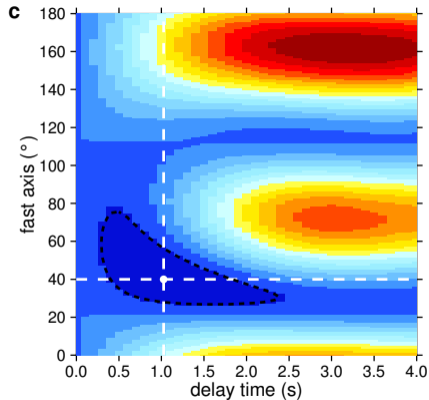

Supplement: Supplementary file 10 — Supplementary Data 8 [file 41467_2023_38296_MOESM10_ESM.zip › TP_CUM_16-Sep-2020_08_44_29_SKS_average.pdf]

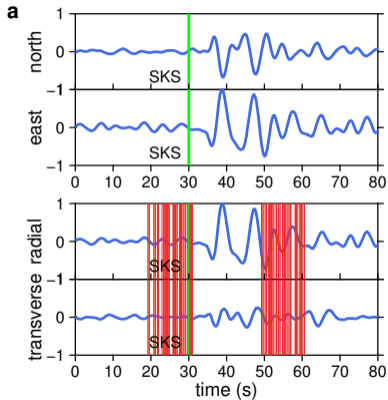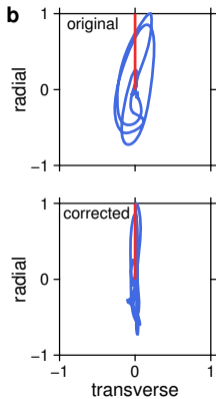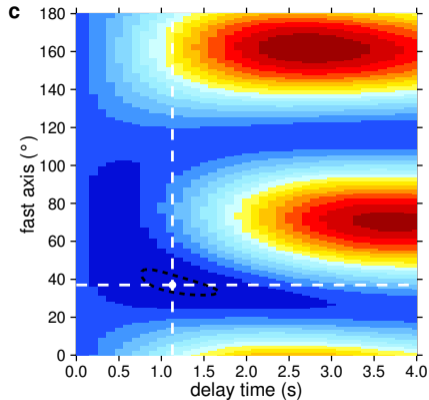

Supplement: Supplementary file 10 — Supplementary Data 8 [file 41467_2023_38296_MOESM10_ESM.zip › TP_CUM_17-Feb-2021_22_49_39_SKS_good.pdf]

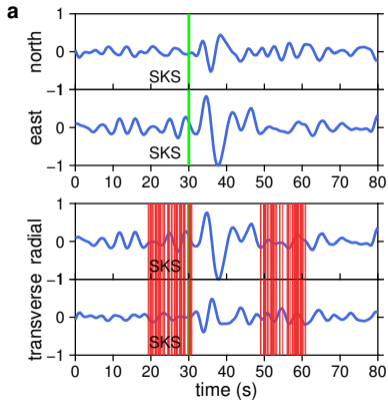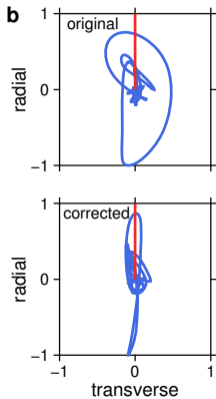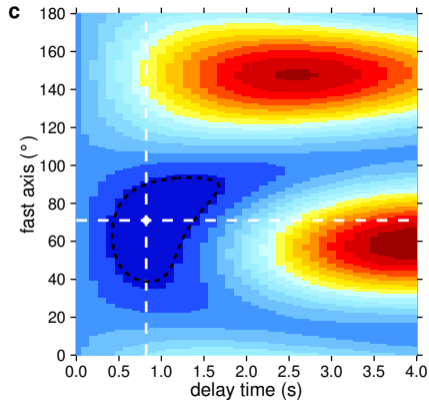

Supplement: Supplementary file 10 — Supplementary Data 8 [file 41467_2023_38296_MOESM10_ESM.zip › TP_CUM_17-Nov-2019_12_13_27_SKS_average.pdf]

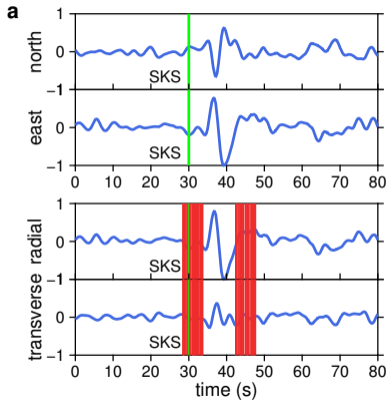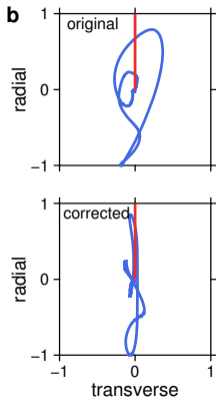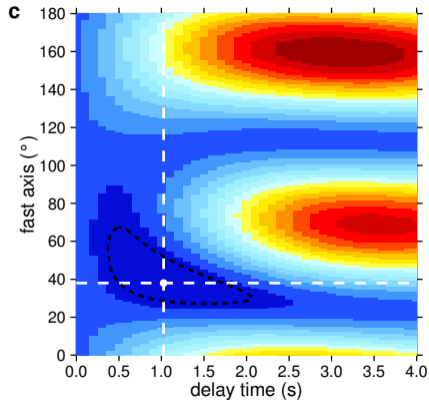

Supplement: Supplementary file 10 — Supplementary Data 8 [file 41467_2023_38296_MOESM10_ESM.zip › TP_CUM_23-Oct-2020_07_04_31_SKS_average.pdf]

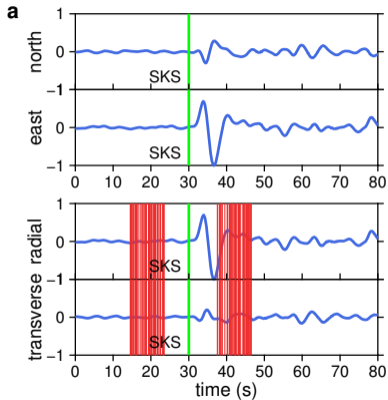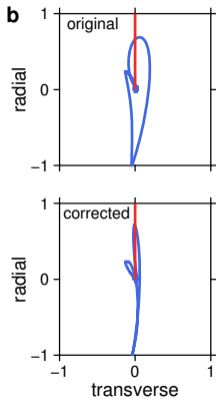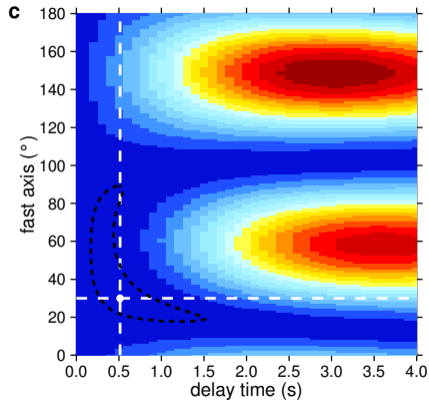

Supplement: Supplementary file 10 — Supplementary Data 8 [file 41467_2023_38296_MOESM10_ESM.zip › TP_CUM_24-Apr-2021_00_23_35_SKS_average.pdf]

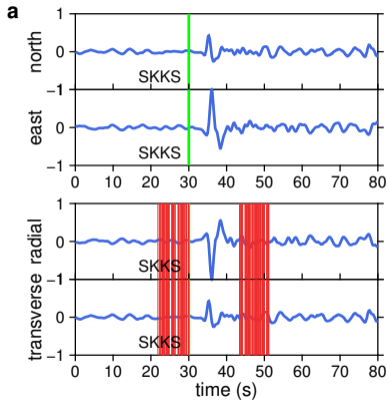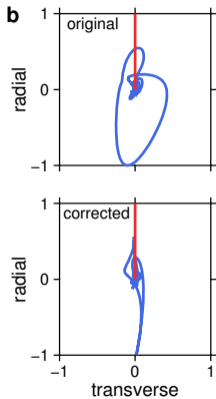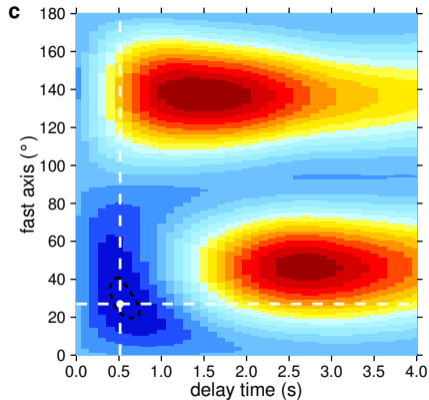

Supplement: Supplementary file 10 — Supplementary Data 8 [file 41467_2023_38296_MOESM10_ESM.zip › TP_CUM_24-Dec-2019_16_43_33_SKKS_good.pdf]

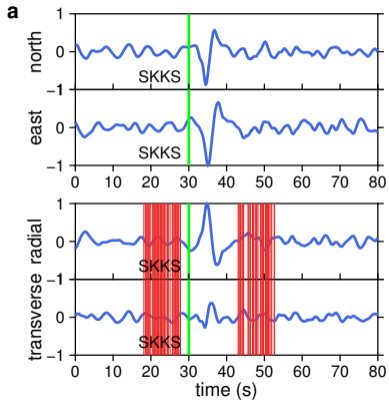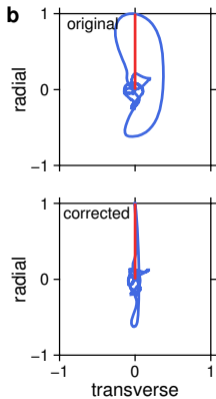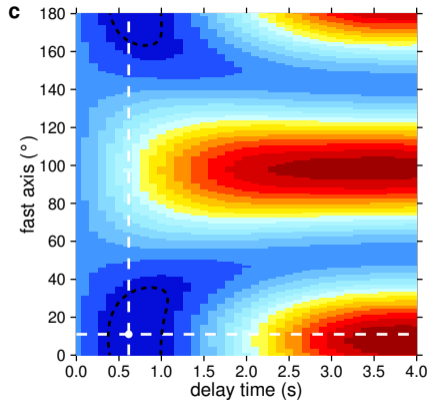

Supplement: Supplementary file 10 — Supplementary Data 8 [file 41467_2023_38296_MOESM10_ESM.zip › TP_CUM_26-Sep-2019_16_36_18_SKKS_average.pdf]

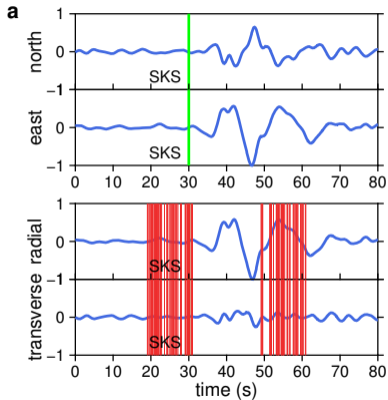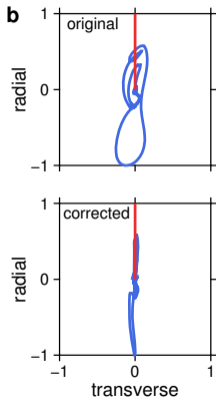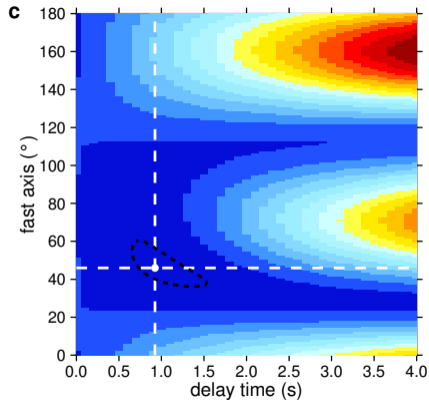

Supplement: Supplementary file 10 — Supplementary Data 8 [file 41467_2023_38296_MOESM10_ESM.zip › TP_CUM_27-Apr-2021_16_33_31_SKS_good.pdf]

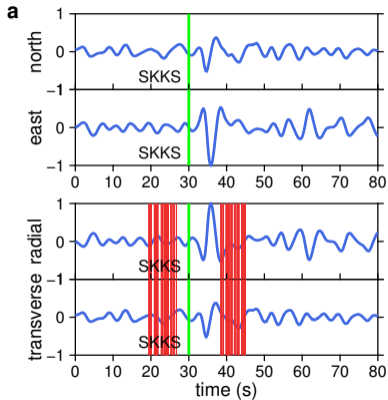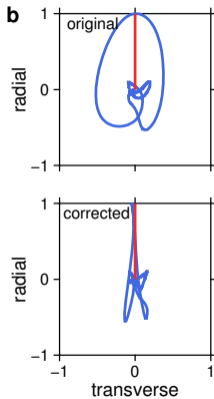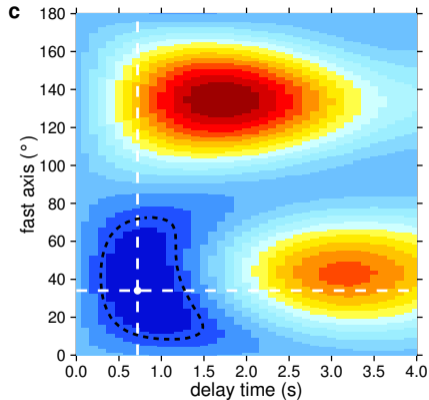

Supplement: Supplementary file 10 — Supplementary Data 8 [file 41467_2023_38296_MOESM10_ESM.zip › TP_CUM_28-Oct-2020_14_53_11_SKKS_average.pdf]

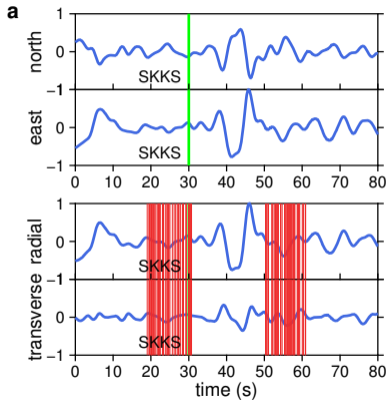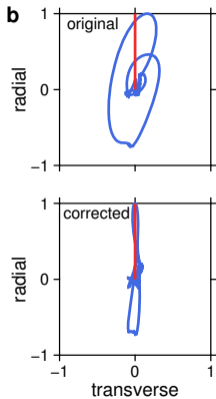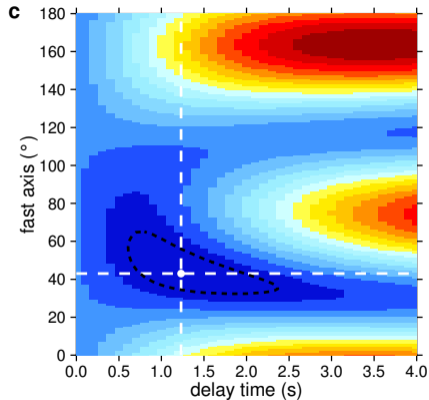

Supplement: Supplementary file 10 — Supplementary Data 8 [file 41467_2023_38296_MOESM10_ESM.zip › TP_CUM_29-Apr-2021_06_50_29_SKKS_average.pdf]

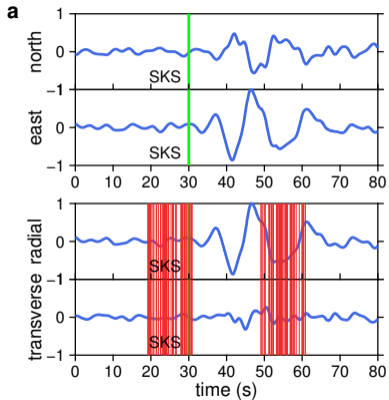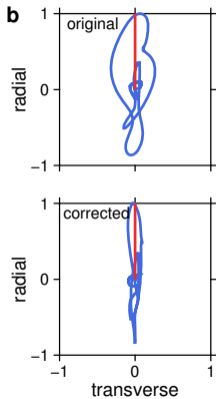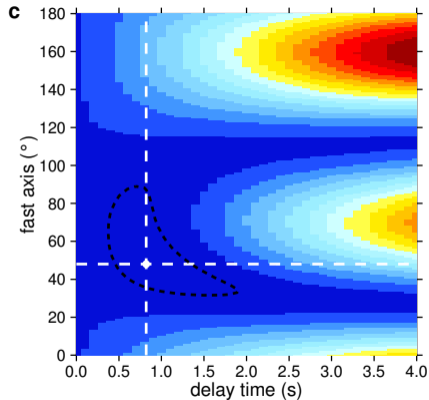

Supplement: Supplementary file 10 — Supplementary Data 8 [file 41467_2023_38296_MOESM10_ESM.zip › TP_CUM_29-Apr-2021_06_50_29_SKS_average.pdf]

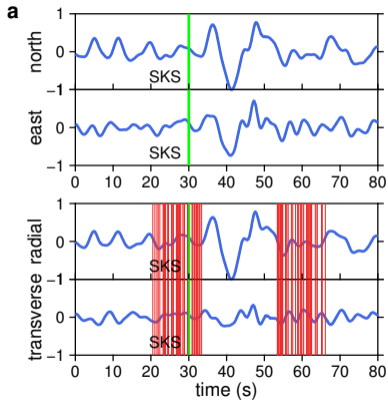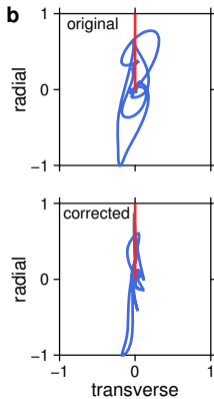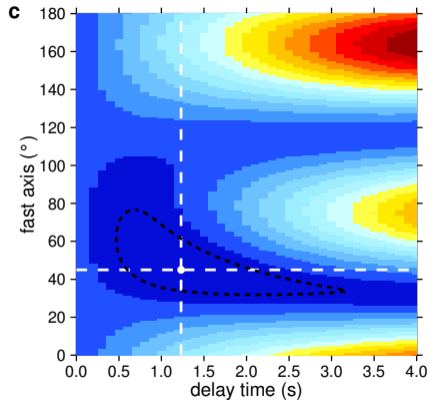

Supplement: Supplementary file 10 — Supplementary Data 8 [file 41467_2023_38296_MOESM10_ESM.zip › TP_CUM_29-Aug-2019_15_07_57_SKS_average.pdf]

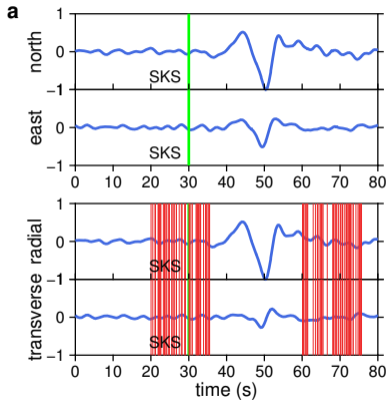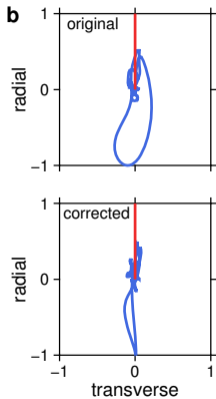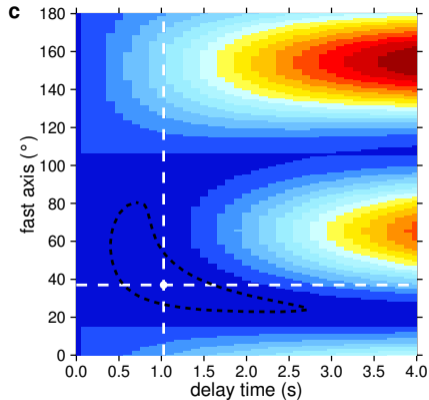

Supplement: Supplementary file 10 — Supplementary Data 8 [file 41467_2023_38296_MOESM10_ESM.zip › TP_CUM_31-Mar-2020_23_52_30_SKS_average.pdf]

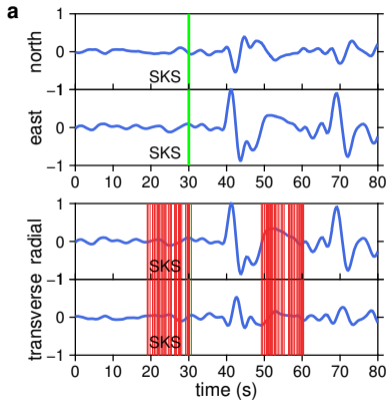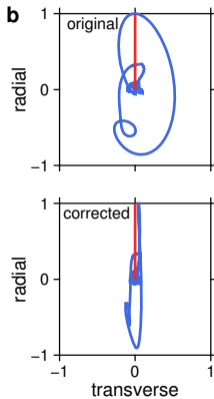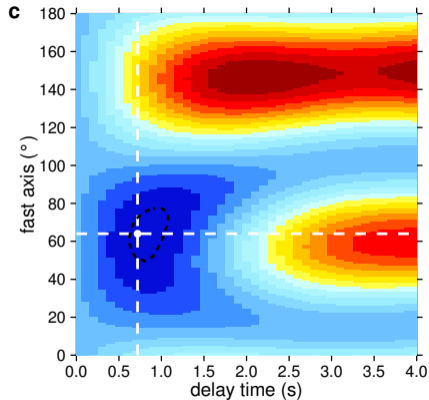

Supplement: Supplementary file 10 — Supplementary Data 8 [file 41467_2023_38296_MOESM10_ESM.zip › TP_CUQ_01-Oct-2020_01_13_41_SKS_good.pdf]

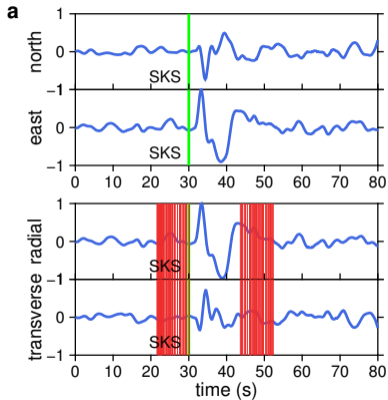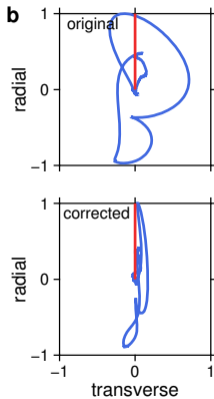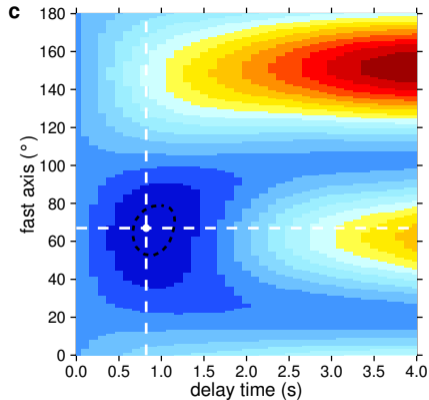

Supplement: Supplementary file 10 — Supplementary Data 8 [file 41467_2023_38296_MOESM10_ESM.zip › TP_CUQ_01-Sep-2019_15_54_20_SKS_good.pdf]

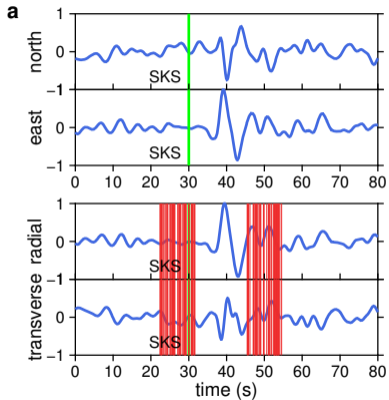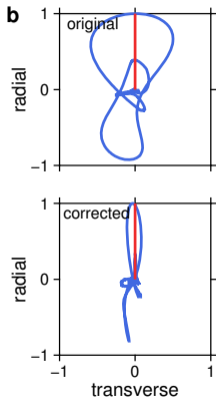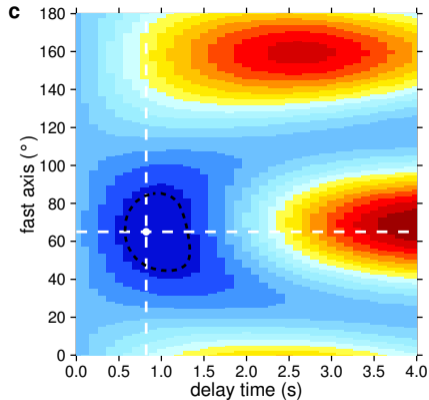

Supplement: Supplementary file 10 — Supplementary Data 8 [file 41467_2023_38296_MOESM10_ESM.zip › TP_CUQ_03-Dec-2020_17_07_30_SKS_average.pdf]

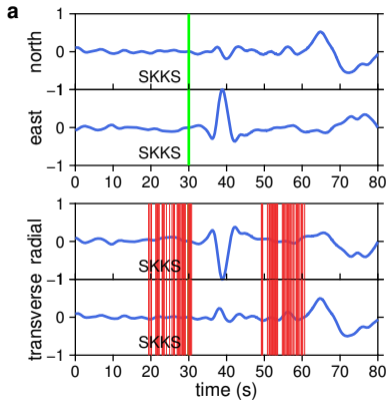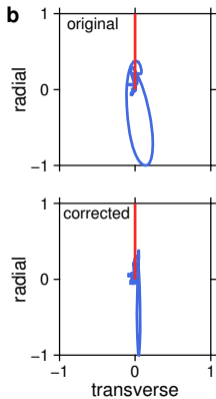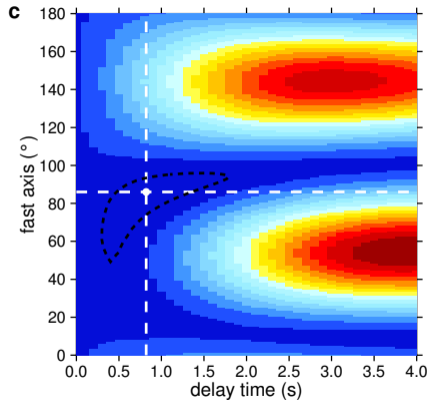

Supplement: Supplementary file 10 — Supplementary Data 8 [file 41467_2023_38296_MOESM10_ESM.zip › TP_CUQ_03-Jun-2020_07_35_36_SKKS_average.pdf]

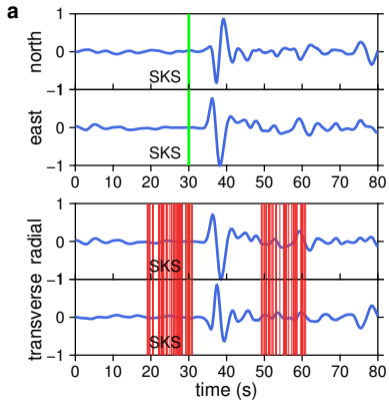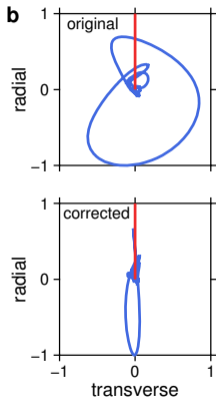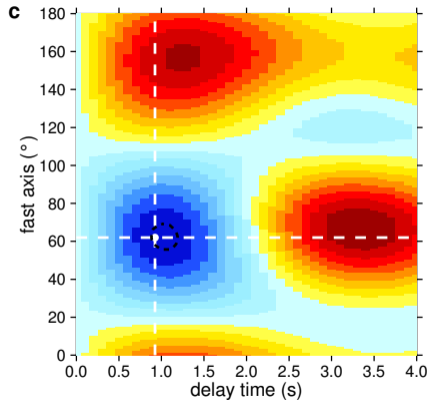

Supplement: Supplementary file 10 — Supplementary Data 8 [file 41467_2023_38296_MOESM10_ESM.zip › TP_CUQ_04-Dec-2019_20_10_03_SKS_good.pdf]

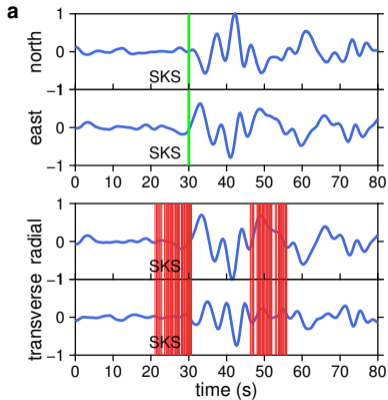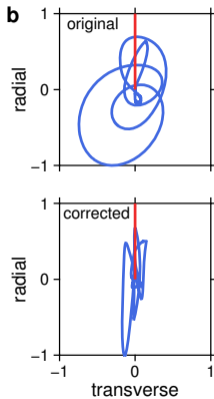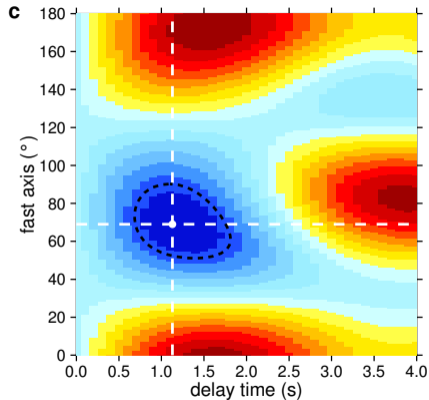

Supplement: Supplementary file 10 — Supplementary Data 8 [file 41467_2023_38296_MOESM10_ESM.zip › TP_CUQ_04-Mar-2021_13_27_33_SKS_good.pdf]

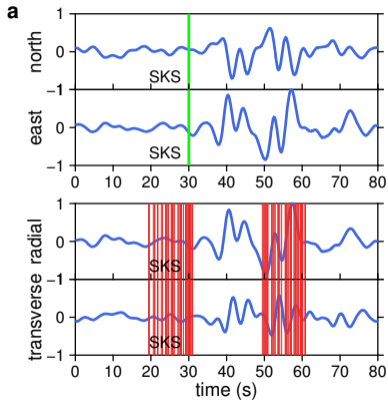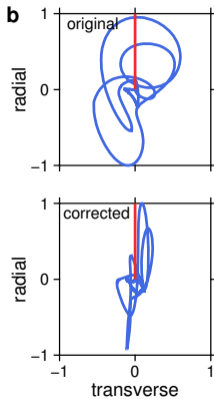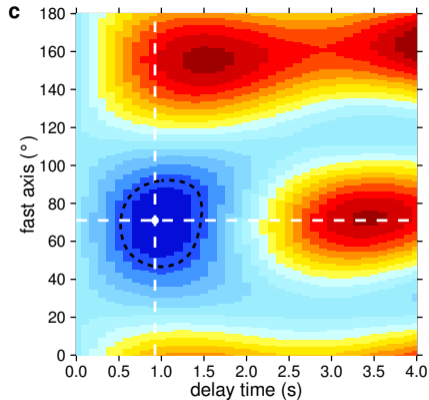

Supplement: Supplementary file 10 — Supplementary Data 8 [file 41467_2023_38296_MOESM10_ESM.zip › TP_CUQ_05-Mar-2021_14_24_54_SKS_average.pdf]

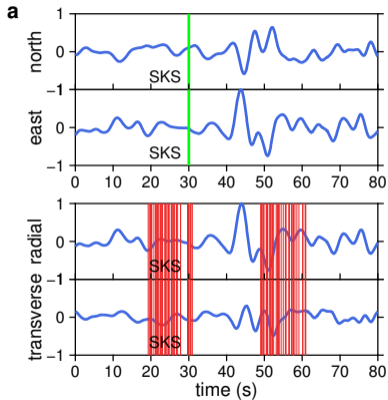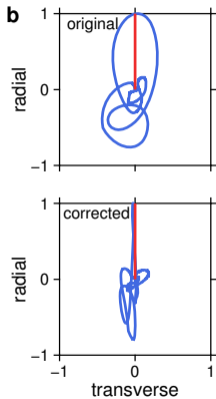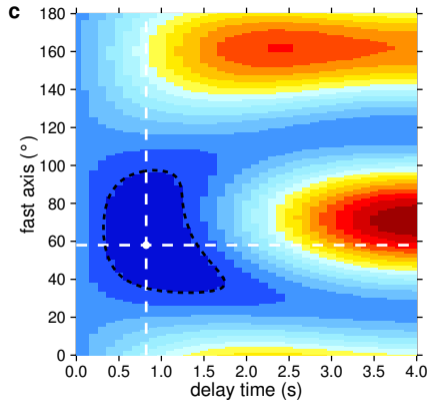

Supplement: Supplementary file 10 — Supplementary Data 8 [file 41467_2023_38296_MOESM10_ESM.zip › TP_CUQ_05-Mar-2021_20_26_39_SKS_average.pdf]

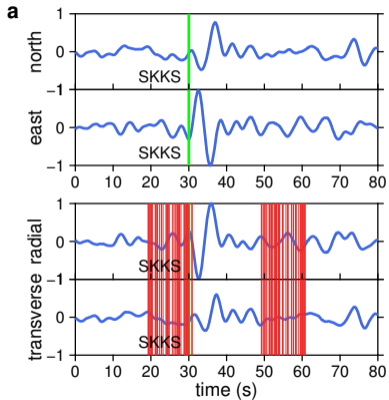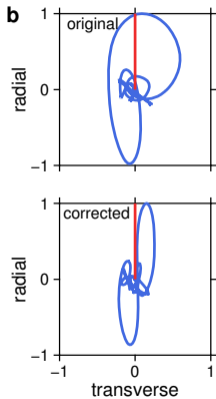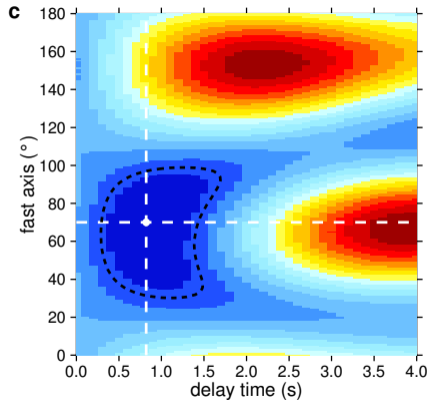

Supplement: Supplementary file 10 — Supplementary Data 8 [file 41467_2023_38296_MOESM10_ESM.zip › TP_CUQ_06-Dec-2020_16_47_39_SKKS_average.pdf]

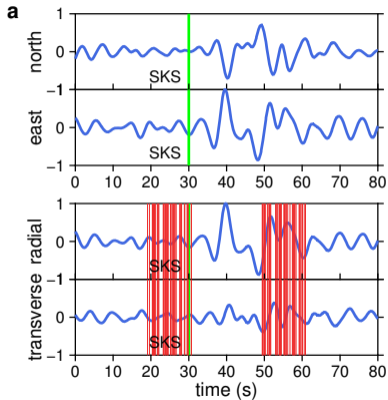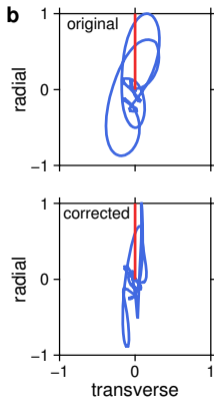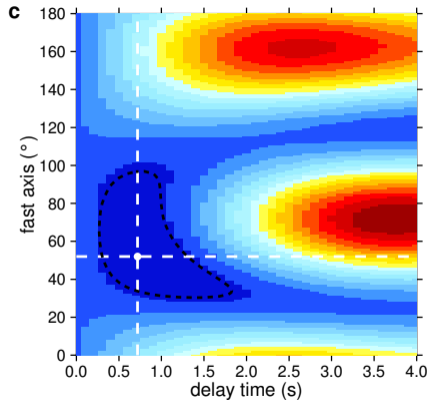

Supplement: Supplementary file 10 — Supplementary Data 8 [file 41467_2023_38296_MOESM10_ESM.zip › TP_CUQ_06-Mar-2021_17_13_12_SKS_average.pdf]

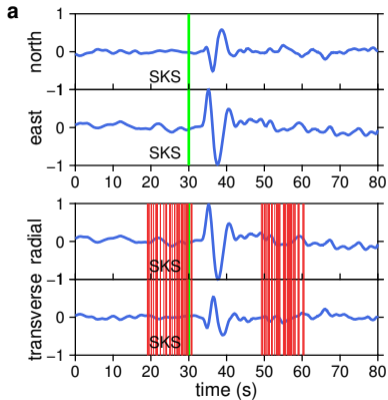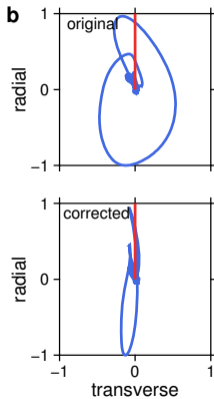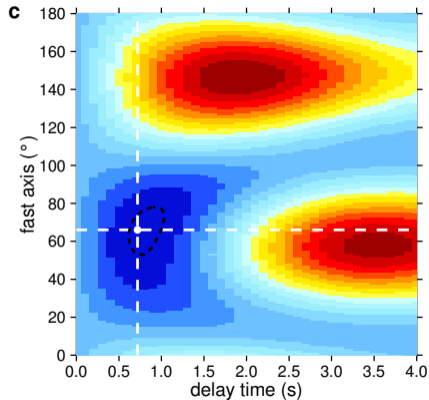

Supplement: Supplementary file 10 — Supplementary Data 8 [file 41467_2023_38296_MOESM10_ESM.zip › TP_CUQ_06-Oct-2020_10_11_45_SKS_good.pdf]

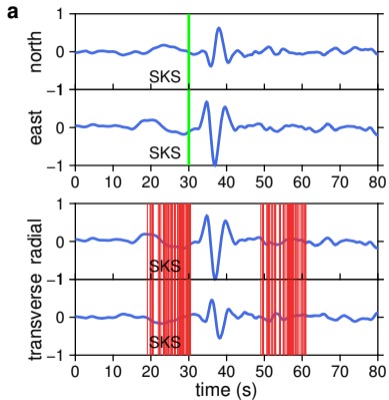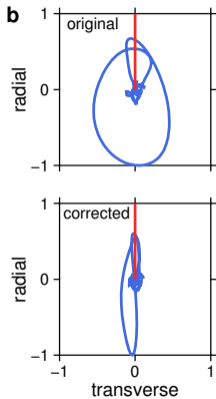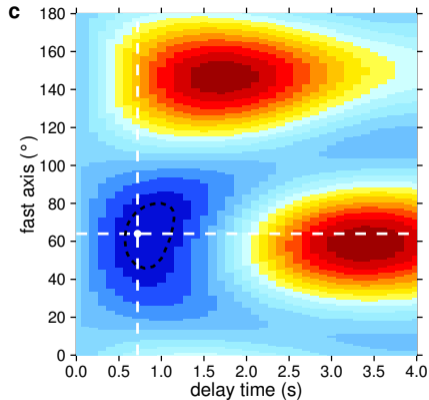

Supplement: Supplementary file 10 — Supplementary Data 8 [file 41467_2023_38296_MOESM10_ESM.zip › TP_CUQ_07-May-2021_23_35_12_SKS_good.pdf]

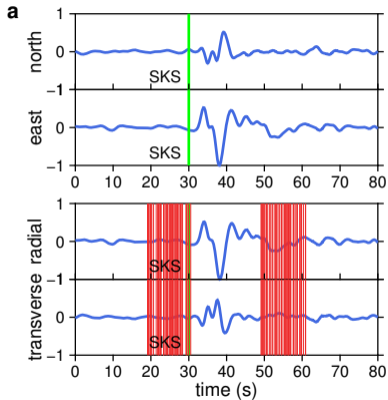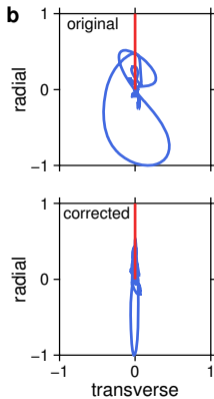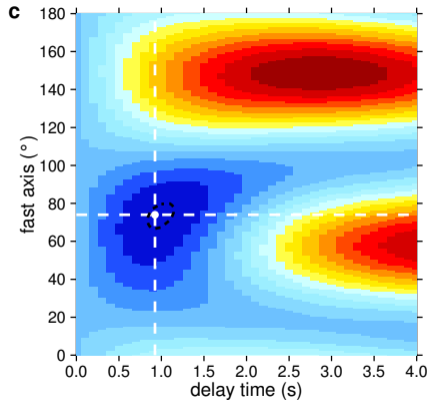

Supplement: Supplementary file 10 — Supplementary Data 8 [file 41467_2023_38296_MOESM10_ESM.zip › TP_CUQ_08-Nov-2019_10_44_44_SKS_good.pdf]

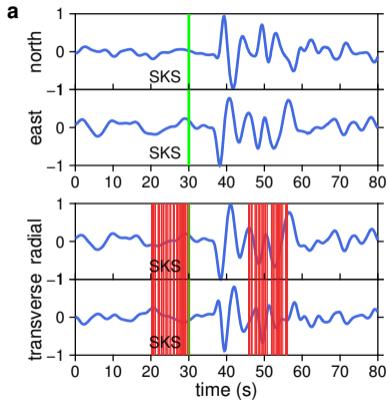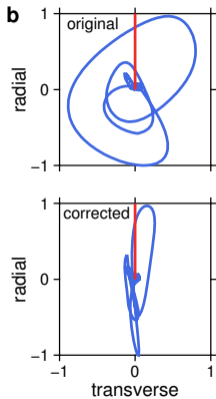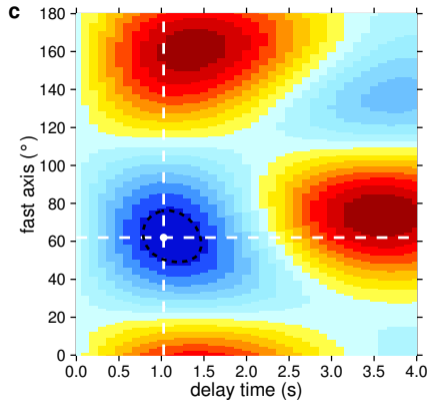

Supplement: Supplementary file 10 — Supplementary Data 8 [file 41467_2023_38296_MOESM10_ESM.zip › TP_CUQ_10-Feb-2021_16_35_26_SKS_average.pdf]

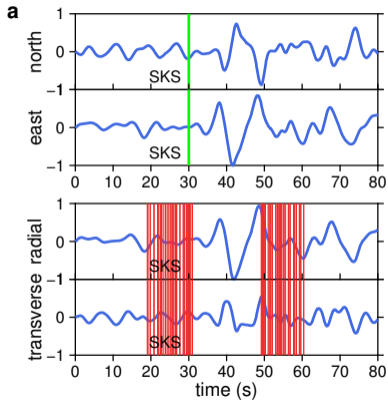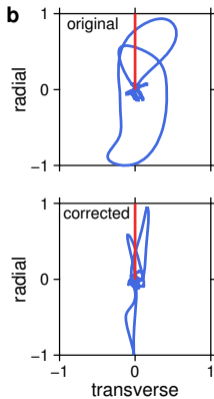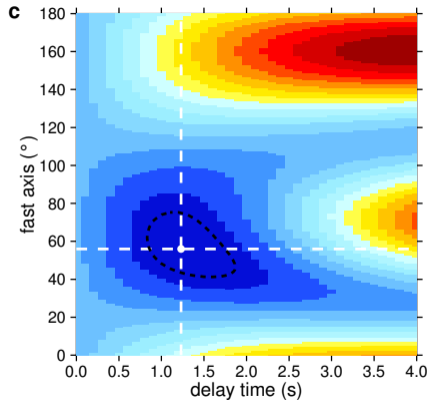

Supplement: Supplementary file 10 — Supplementary Data 8 [file 41467_2023_38296_MOESM10_ESM.zip › TP_CUQ_10-Feb-2021_18_36_46_SKS_average.pdf]

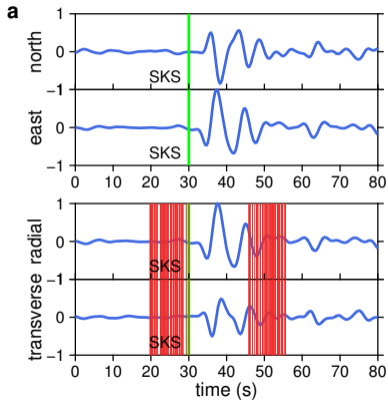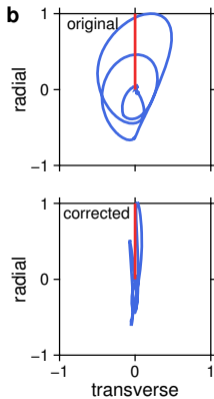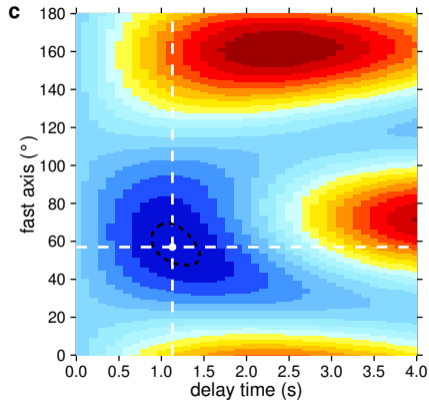

Supplement: Supplementary file 10 — Supplementary Data 8 [file 41467_2023_38296_MOESM10_ESM.zip › TP_CUQ_10-Feb-2021_21_23_58_SKS_average.pdf]

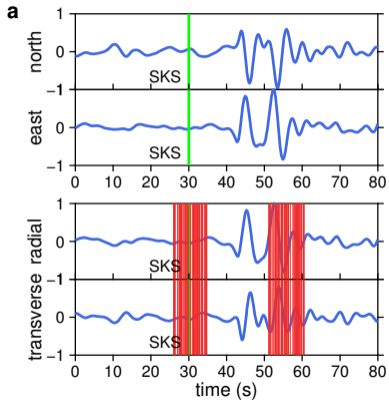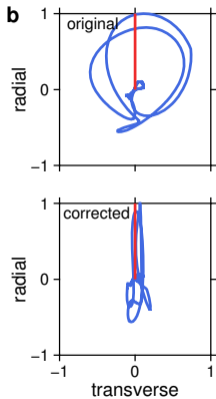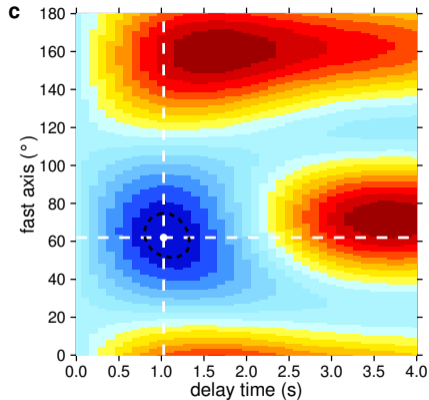

Supplement: Supplementary file 10 — Supplementary Data 8 [file 41467_2023_38296_MOESM10_ESM.zip › TP_CUQ_10-Feb-2021_23_45_22_SKS_good.pdf]

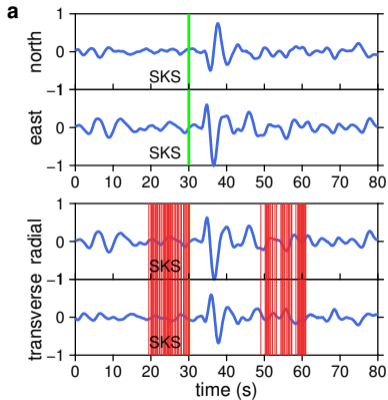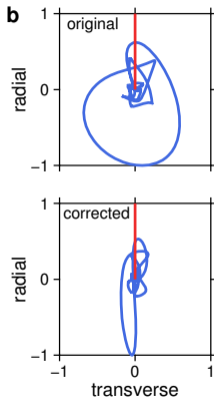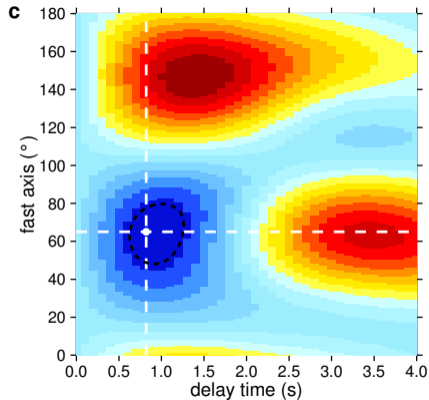

Supplement: Supplementary file 10 — Supplementary Data 8 [file 41467_2023_38296_MOESM10_ESM.zip › TP_CUQ_10-Mar-2021_20_12_39_SKS_good.pdf]

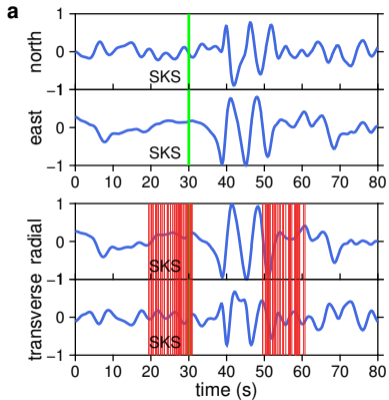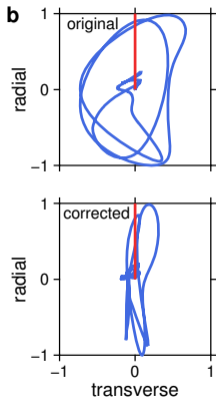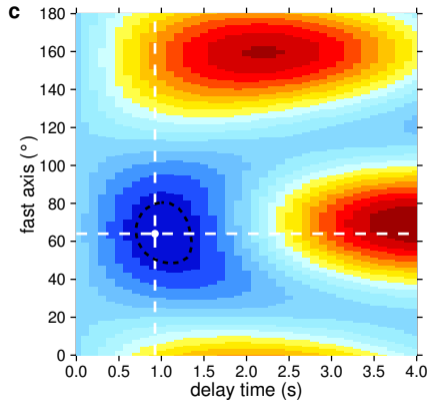

Supplement: Supplementary file 10 — Supplementary Data 8 [file 41467_2023_38296_MOESM10_ESM.zip › TP_CUQ_11-Feb-2021_00_14_52_SKS_average.pdf]

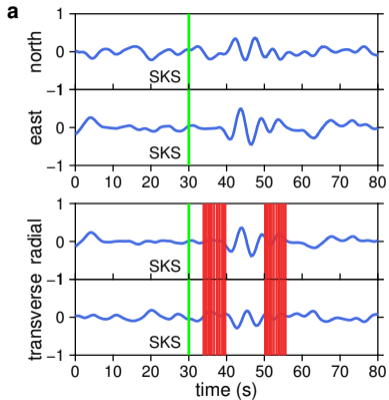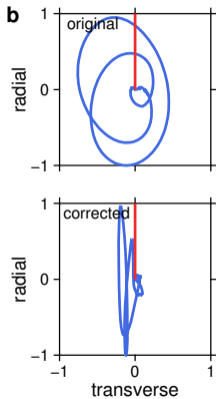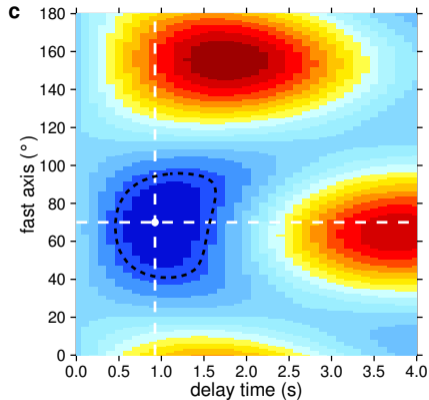

Supplement: Supplementary file 10 — Supplementary Data 8 [file 41467_2023_38296_MOESM10_ESM.zip › TP_CUQ_11-Feb-2021_08_39_14_SKS_average.pdf]

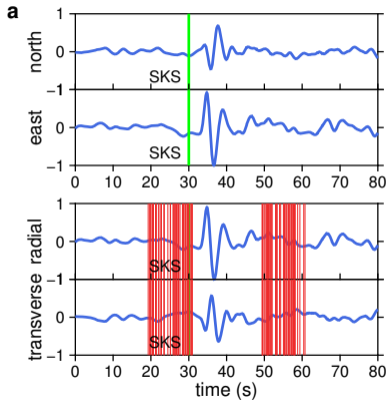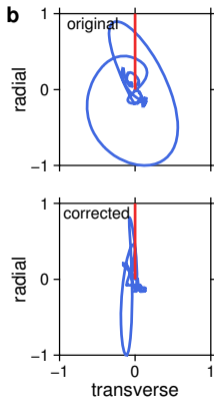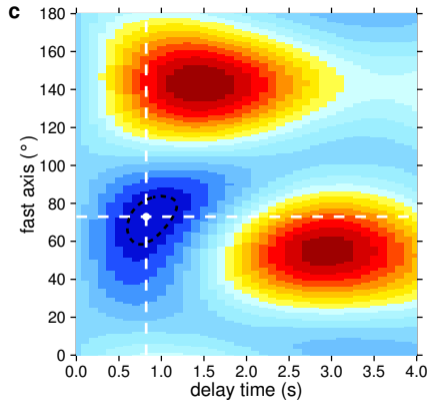

Supplement: Supplementary file 10 — Supplementary Data 8 [file 41467_2023_38296_MOESM10_ESM.zip › TP_CUQ_11-Nov-2020_00_48_43_SKS_good.pdf]

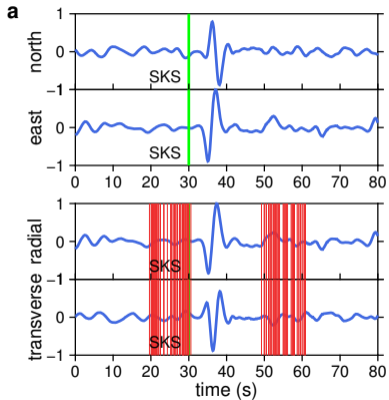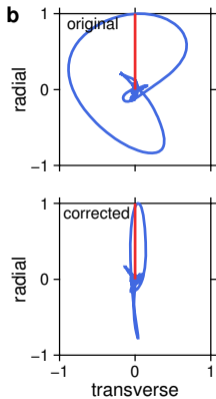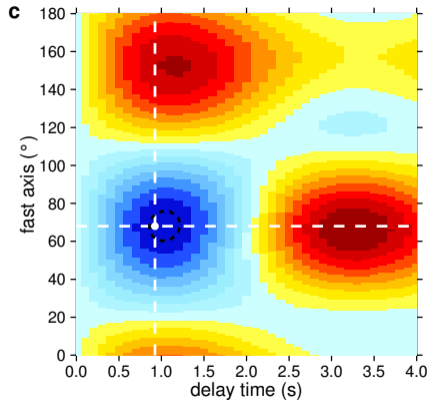

Supplement: Supplementary file 10 — Supplementary Data 8 [file 41467_2023_38296_MOESM10_ESM.zip › TP_CUQ_12-Mar-2021_00_03_21_SKS_good.pdf]

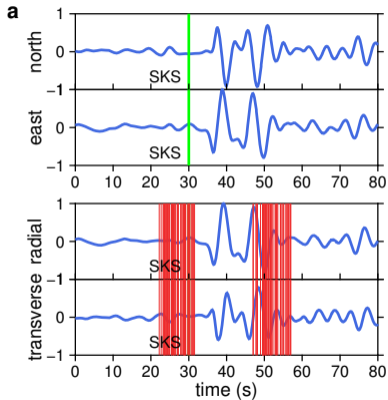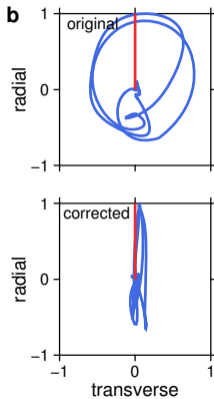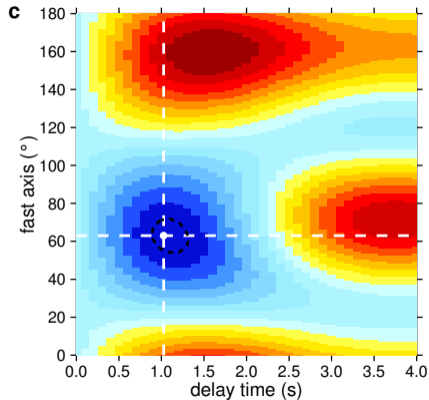

Supplement: Supplementary file 10 — Supplementary Data 8 [file 41467_2023_38296_MOESM10_ESM.zip › TP_CUQ_17-Feb-2021_22_49_39_SKS_good.pdf]

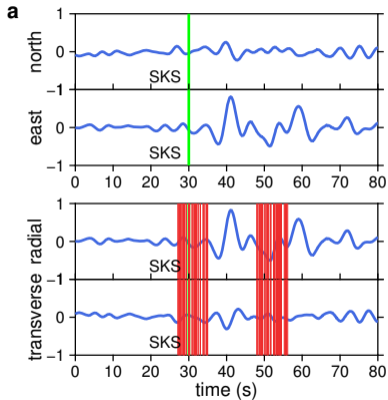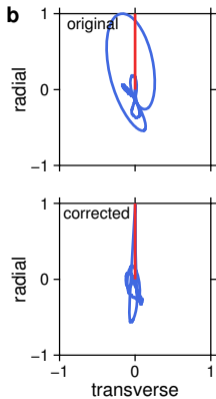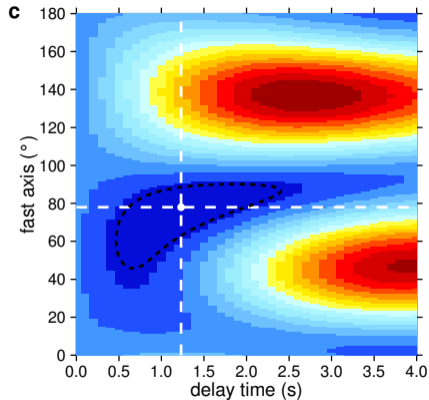

Supplement: Supplementary file 10 — Supplementary Data 8 [file 41467_2023_38296_MOESM10_ESM.zip › TP_CUQ_17-Mar-2020_16_06_22_SKS_average.pdf]

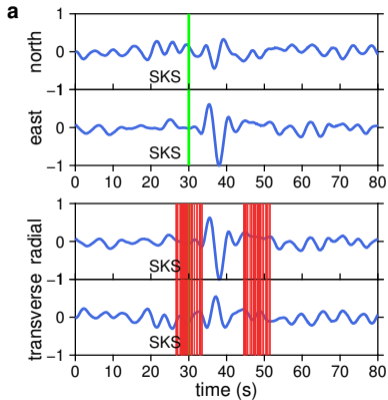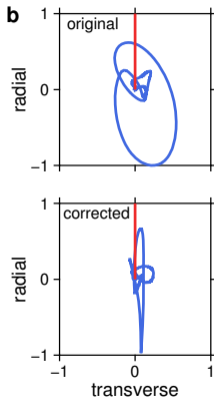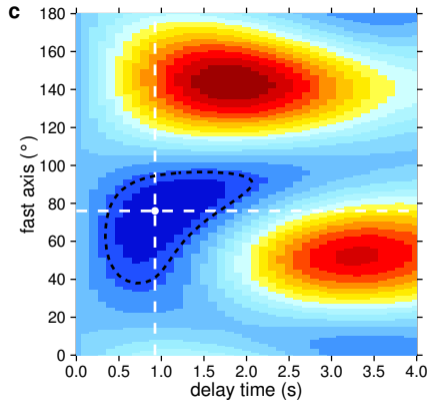

Supplement: Supplementary file 10 — Supplementary Data 8 [file 41467_2023_38296_MOESM10_ESM.zip › TP_CUQ_17-Nov-2019_12_13_27_SKS_average.pdf]
