# Supplementary material for: Southern Tibetan rifting since late Miocene enabled by basal shear of the underthrusting Indian lithosphere
Source: Nat Commun. 2023 May 4;14:2565. doi: 10.1038/s41467-023-38296-w (PMC10160080; doi:10.1038/s41467-023-38296-w)
Supplement: Supplementary file 6 — Supplementary Data 4 [file 41467_2023_38296_MOESM6_ESM.zip › unstable_Y2-WT18.pdf]

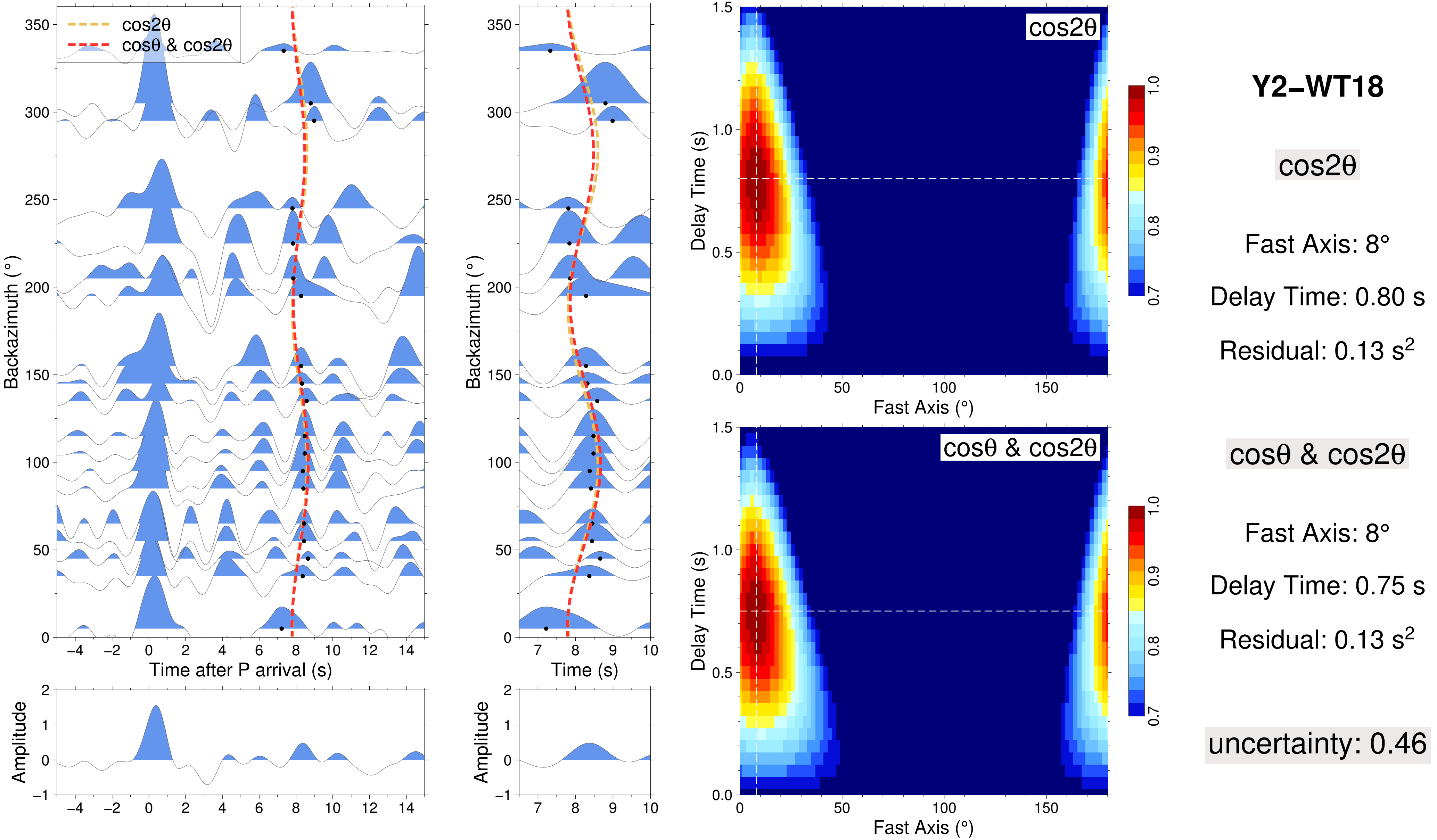

cos2θ
cosθ & cos2θ
Backazimuth (°)
Time after P arrival (s)
Amplitude
Time (s)
Delay Time (s)
Fast Axis (°)
Y2–WT18
cos2θ
Fast Axis: 8°
Delay Time: 0.80 s
Residual: 0.13 s2
cosθ & cos2θ
Fast Axis: 8°
Delay Time: 0.75 s
Residual: 0.13 s2
uncertainty: 0.46
